# Supplementary material for: DABCO-Catalyzed Mono-/Diallylation of N-Unsubstituted Isatin N,N′-Cyclic Azomethine Imine 1,3-Dipoles with Morita-Baylis-Hillman Carbonates
Source: Molecules. 2023 Mar 28;28(7):3002. doi: 10.3390/molecules28073002 (PMC10095907; doi:10.3390/molecules28073002)
Supplement: Supplementary file 1 [file molecules-28-03002-s001.zip › molecules-2290215-supplementary-final.pdf]

Supplementary Materials

DABCO-Catalyzed Mono-/Diallylation of *N*-Unsubstituted Isatin *N,N′*-Cyclic Azomethine Imine 1,3-Dipoles with Morita-Baylis-Hillman Carbonates

Qiumi Wang [1,†], Sicheng Li [2,†], Guosheng Yang [1], Xinyu Zou [1], Xi Yin [1], Juhua Feng [1], Huabao Chen [3], Chunping Yang [3], Li Zhang [1], Cuifen Lu [4] and Guizhou Yue [1,*]

1 College of Science, Sichuan Agricultural University, Ya'an 625014, China
2 The Yingjing County Emergency Management Agency, Ya'an 625200, China
3 College of Agronomy, Sichuan Agricultural University, Chengdu 611130, China
4 Hubei Collaborative Innovation Center for Advanced Organochemical Materials, Ministry-of-Education Key Laboratory for the Synthesis and Application of Organic Functional Molecules, Hubei University, Wuhan 430062, China
* Correspondence: yueguizhou@sicau.edu.cn
† These authors contributed equally to this work.

1. The phenomenon of the reaction and TLC.

The phenomenon of the reaction for **1a** and **2a** under DABCO in DCM at rt. (a) no DABCO (0 min, a yellow cloudy solution); (b) reaction finished (5 min, a yellow clear solution) (Figure 1). TLC of starting materials (**1a** and **2a**) and products (**3a** and **4a**) showed the result (Figure 2).

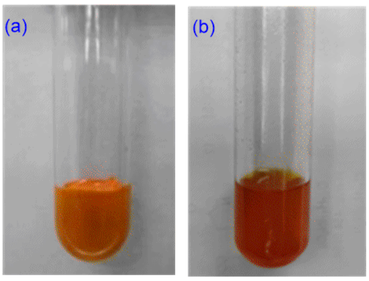

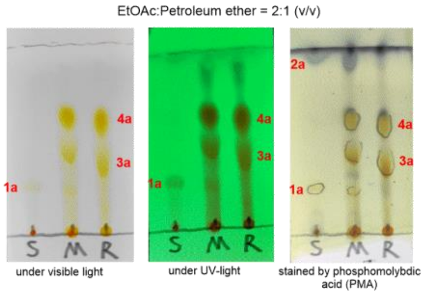

Fig. 1 (left) and Fig. 2 (right)

2. Data for all new compounds.

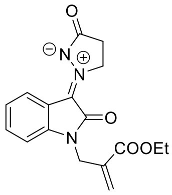

[Reaction time: 5 min]; **3a**: 297 mg, 91%, a yellow solid, m.p. 192.7-193.2 ºC; IR (thin film): ν_{max} 3684, 3987, 1710, 1694, 1573, 1383, 1267, 1128, 761 cm^{-1}; ^{1}H NMR (400 MHz, $CDCl_3$): δ 8.42 (d, *J*= 7.6 Hz, 1 H), 7.38 (td, *J*= 7.8, 0.8 Hz, 1 H), 7.15 (ψt, *J*= 7.6 Hz, 1 H), 6.85 (d, *J*= 8.0 Hz, 1 H), 6.34 (s, 1 H), 5.59 (s, 1 H), 5.00 (t, *J*= 7.6 Hz, 2 H), 4.64 (s, 2 H), 4.27 (q, *J*= 7.2 Hz, 2 H), 2.88 (t, *J*= 7.6 Hz, 2 H), 1.33 (t, *J*= 7.2 Hz, 3 H); ^{13}C NMR (100 MHz, $CDCl_3$): δ 186.7, 165.4, 160.9, 141.6, 133.8, 132.6, 127.8, 126.5, 124.5,123.5, 116.9, 109.2, 61.4, 57.4, 40.6, 28.4, 14.2; HRMS (ESI): *m/z* calcd for $C_{17}H_{17}N_3O_4Na$ $[M+Na]^+$ 350.1117, found 350.1116.

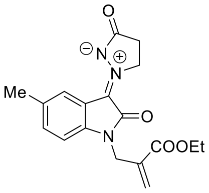

[Reaction time: 5 min]; **3b**: 266 mg, 78%, a red solid, m.p. 184.2-186.0 ºC; IR (thin film): ν_{max} 3671, 2996, 1720, 1699, 1578, 1368, 1149, 1132, 734 cm^{-1}; ^{1}H NMR (400 MHz, $CDCl_3$): δ 8.28 (s, 1 H), 7.19 (d, *J*= 8.0 Hz, 1 H), 7.74 (d, *J*= 8.0 Hz, 1 H), 6.33 (s, 1 H), 5.57 (s, 1 H), 5.00 (ψt, *J*= 7.4 Hz, 2 H), 4.62 (s, 2 H), 4.27 (q, *J*= 7.2 Hz, 2 H), 2.89 (ψt, *J*= 7.6 Hz, 2 H), 2.36 (s, 3 H), 1.33 (t, *J*= 7.2 Hz, 3 H); ^{13}C NMR (100 MHz, $CDCl_3$): δ 186.8, 165.4, 160.8, 139.5, 133.8, 133.4, 133.2, 128.2, 126.4, 124.4, 116.8, 108.9, 61.3, 57.4, 40.5, 28.5, 21.0, 14.2; HRMS (ESI): *m/z* calcd for $C_{18}H_{20}N_3O_4$ $[M+H]^+$ 342.1454, found 342.1428.

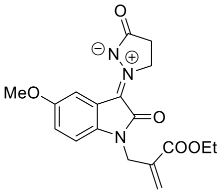

[Reaction time: 3 min]; **3c**: 303 mg, 86%, a red solid, m.p. 172.2-174.7 ºC; IR (thin film): ν_{max} 3684, 2986, 1725, 1701, 1570, 1367, 1264, 1014, 762 cm^{-1}; ^{1}H NMR (400 MHz, $CDCl_3$): δ 8.05 (d, *J*= 2.8 Hz, 1 H), 6.95 (dd, *J*= 8.8, 2.8 Hz, 1 H), 6.77 (d, *J*= 7.2 Hz, 1 H), 6.34 (s, 1 H), 5.59 (s, 1 H), 5.02 (ψt, *J*= 7.6 Hz, 2 H), 4.62 (s, 2 H), 4.28 (q, *J*= 7.2 Hz, 2 H), 3.84 (s, 3 H), 2.91-2.87 (m, 2 H), 1.33 (t, *J*= 7.2 Hz, 3 H); ^{13}C NMR (100 MHz, $CDCl_3$): δ 186.7, 165.4, 160.9, 156.3, 135.5, 133.9, 126.5, 125.0, 119.4, 117.4, 112.3, 110.0, 61.3, 57.5, 56.2, 40.6, 28.4, 14.2; HRMS (ESI): *m/z* calcd for $C_{18}H_{20}N_3O_5$ $[M+H]^+$ 358.1403, found 358.1377.

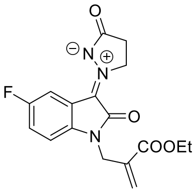

[Reaction time: 8 min]; **3d**: 169 mg, 49%, a yellow solid, m.p. 135.9-138.2 ºC; IR (thin film): ν_{max} 3671, 2984, 1705, 1615, 1578, 1262, 1115, 659 cm^{-1}; ^{1}H NMR (400 MHz, $CDCl_3$): δ 8.17 (dd, *J*= 8.2, 2.6 Hz, 1 H), 7.08 (m, 1 H), 6.82 (dd, *J*= 8.4, 4.0 Hz, 1 H), 6.36 (s, 1 H), 5.62 (s, 1 H), 5.03 (ψt, *J*= 7.6 Hz, 2 H), 4.64 (s, 2 H), 4.27 (q, *J*= 7.2 Hz, 2 H), 2.90 (t, *J*= 7.4 Hz, 2 H), 1.33 (t, *J*= 7.2 Hz, 3 H); ^{13}C NMR (100 MHz, $CDCl_3$): δ 186.7, 165.3, 160.8, 157.9, 137.5 (d), 133.8, 126.9, 123.7, 119.9 (d), 117.7 (d), 114.8 (d),

110.0 (d), 61.4, 57.7, 40.8, 28.3, 14.2; ^{19}F NMR (376 MHz, $CDCl_3$): δ -118.7; HRMS (ESI): *m/z* calcd for $C_{17}H_{17}FN_3O_4$ $[M+H]^+$ 346.1203, found 346.1131.

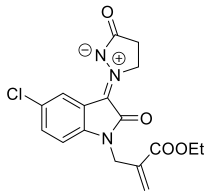

[Reaction time: 14 min]; **3e**: 173 mg, 50%, a yellow solid, m.p. 164.1-165.9 °C; IR (thin film): ν_{max} 3410, 3101, 2989, 1710, 1643, 1592, 1265, 1148, 776, 694 cm^{-1}; ^{1}H NMR (400 MHz, $CDCl_3$): δ 8.39 (d, *J*= 2.0 Hz, 1 H), 7.32 (dd, *J*= 8.4, 2.0 Hz, 1 H), 6.81 (d, *J*= 8.4 Hz, 1 H), 6.36 (s, 1 H), 5.62 (s, 1 H), 5.03 (ψt, *J*= 7.6 Hz, 2 H), 4.64 (s, 2 H), 4.26 (q, *J*= 7.2 Hz, 2 H), 2.90 (t, *J*= 7.4 Hz, 2 H), 1.33 (t, *J*= 7.2 Hz, 3 H); ^{13}C NMR (100 MHz, $CDCl_3$): δ 186.6, 165.3, 160.8, 160.2, 137.5, 133.8, 126.9, 123.7, 119.9, 117.7, 114.8, 110.0, 61.4, 57.7, 40.8, 28.3, 14.2; ^{13}C NMR (100 MHz, $CDCl_3$): δ 186.7, 165.3, 160.6, 139.8, 133.7, 132.0, 129.0, 127.1, 127.0, 123.1, 118.0, 110.2, 61.4, 57.8, 40.8, 28.3, 14.2; HRMS (ESI): *m/z* calcd for $C_{17}H_{17}ClN_3O_4$ $[M+H]^+$ 362.0908, found 362.0905.

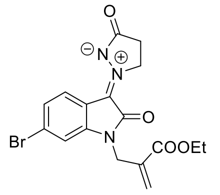

[Reaction time: 12 min]; **3f**: 329 mg, 81%, a yellow solid, m.p. 166.7-168.3 °C; IR (thin film): ν_{max} 3406, 2930, 1709, 1644, 1599, 1374, 1153, 1025, 783, 511 cm^{-1}; ^{1}H NMR (400 MHz, $CDCl_3$): δ 8.29 (d, *J*= 8.0 Hz, 1 H), 7.30 (dd, *J*= 8.0, 1.6 Hz, 1 H), 7.04 (d, *J*= 1.2 Hz, 1 H), 6.38 (s, 1 H), 5.60 (s, 1 H), 4.98 (ψt, *J*= 7.6 Hz, 2 H), 4.64 (s, 2 H), 4.28 (q, *J*= 7.2 Hz, 2 H), 2.89 (t, *J*= 7.4 Hz, 2 H), 1.34 (t, *J*= 7.2 Hz, 3 H); ^{13}C NMR (100 MHz, $CDCl_3$): δ 186.6, 165.2, 160.7, 142.3, 133.5, 128.4, 127.0, 126.7, 126.5, 123.3, 115.8, 112.7, 61.5, 57.7, 40.8, 28.3, 14.2; HRMS (ESI): *m/z* calcd for $C_{17}H_{17}BrN_3O_4$ $[M+H]^+$ 406.0402, found 406.0427.

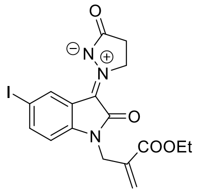

[Reaction time: 12 min]; **3g**: 353 mg, 78%, a yellow solid, m.p. 204.1-206.6 °C; IR (thin film): ν_{max} 3425, 1705, 1694, 1600, 1384, 1163, 1127, 778, 518 cm^{-1}; ^{1}H NMR (400 MHz, $CDCl_3$): δ 8.74 (s, 1 H), 7.70 (d, *J*= 8.0 Hz, 1 H), 6.68 (d, *J*= 8.4 Hz, 1 H), 6.35 (s, 1 H), 5.60 (s, 1 H), 5.03 (ψt, *J*= 2.8 Hz, 2 H), 4.64 (s, 2 H), 4.26 (q, *J*= 6.8 Hz, 2 H), 2.90 (t, *J*= 6.4 Hz, 2 H), 1.34 (t, *J*= 6.8 Hz, 3 H); ^{13}C NMR (100 MHz, $CDCl_3$): δ 186.6, 165.3, 160.4, 140.9, 140.8, 135.3, 133.7, 126.9, 122.8, 118.6, 111.2, 86.1, 61.4, 57.8, 40.7, 28.3, 14.2; HRMS (ESI): *m/z* calcd for $C_{17}H_{17}IN_3O_4$ $[M+H]^+$ 454.0264, found 454.0259.

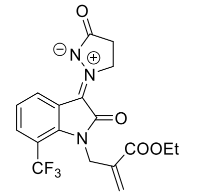

[Reaction time: 20 min]; **3h**: 229 mg, 58%, a yellow solid, m.p. 127.8-128.4 °C; IR (thin film): ν_{max} 3355, 2984, 1716, 1599, 1369, 1330, 1124, 750 cm^{-1}; ^{1}H NMR (400 MHz, $CDCl_3$): δ 8.17 (dd, *J*= 8.2, 2.6 Hz, 1 H), 7.08 (m, 1 H), 6.82 (dd, *J*= 8.4, 4.0 Hz, 1 H), 6.36 (s, 1 H), 5.62 (s, 1 H), 5.03 (ψt, *J*= 7.6 Hz, 2 H), 4.64 (s, 2 H), 4.27 (q, *J*= 7.2 Hz, 2 H), 2.90 (t, *J*= 7.4 Hz, 2 H), 1.33 (t, *J*= 7.2 Hz, 3 H); ^{19}F NMR (376 MHz, $CDCl_3$): δ -55.7; ^{13}C NMR (100 MHz, $CDCl_3$): δ 186.7, 165.1, 161.6, 138.8, 134.6, 130.9, 129.8 (q), 124.3, 123.0, 122.9, 121.6, 119.4, 112.9 (q), 61.2, 58.5, 42.8 (d), 28.0, 14.2; ^{19}F NMR (376 MHz, $CDCl_3$): δ -55.7; HRMS (ESI): *m/z* calcd for $C_{18}H_{17}F_3N_3O_4$ $[M+H]^+$ 396.1171, found 396.1164.

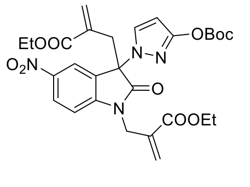

[Reaction time: 1 min]; **3'i**: 275 mg, 47%, a white solid, m.p. 159.0-161.7 °C; IR (thin film): ν_{max} 3164, 1772, 1751, 1709, 1617, 1375, 1150, 759 cm^{-1}; ^{1}H NMR (400 MHz, DMSO-*d*$_6$): δ 8.33 (dd, *J*= 8.8, 2.4 Hz, 1 H), 8.18 (dd, *J*= 7.8, 2.6 Hz, 2 H), 7.34 (dd, *J*= 8.8 Hz, 1 H), 6.27 (d, *J*= 2.8 Hz, 1 H), 6.20 (s, 1 H), 6.02 (s, 1 H), 5.63 (s, 1 H), 5.55 (s, 1 H), 4.63 (s, 2 H), 4.22-4.13 (m, 2 H), 3.91-3.75 (m, 3 H), 3.43 (d, *J*= 12.8 Hz, 1 H), 1.45 (s, 9 H), 1.23 (t, *J*= 7.0 Hz, 3 H), 1.02 (d, *J*= 7.0 Hz, 3 H); ^{13}C NMR (100 MHz, DMSO-*d*$_6$): δ 172.9, 166.1, 165.1, 155.7, 150.1, 148.6, 143.3, 133.7, 133.3, 131.8, 131.4, 127.7, 127.5, 126.4, 121.7, 111.1, 97.6, 84.5, 68.7, 61.3, 61.2, 41.4, 35.2, 27.5, 14.4, 14.1; HRMS (ESI): *m/z* calcd for $C_{28}H_{32}N_4NaO_{10}$ $[M+Na]^+$ 607.2016, found 607.1944.

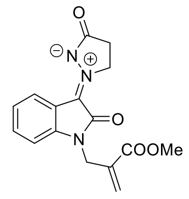

[Reaction time: 5 min]; **3j**: 250 mg, 80%, a yellow solid, m.p. 199.1-201.2 °C; IR (thin film): ν_{max} 3417, 3103, 2950, 1730, 1713, 1573, 1350, 1267, 1113, 761 cm^{-1}; ^{1}H NMR (400 MHz, $CDCl_3$): δ 8.44 (d, *J*= 7.6 Hz, 1 H), 7.39 (m, 1 H), 7.16 (ψt, *J*= 7.6 Hz, 1 H), 6.86 (d, *J*= 8.0 Hz, 1 H), 6.35 (s, 1 H), 5.61 (s, 1 H), 5.01 (ψt, *J*= 7.4 Hz, 2 H), 4.65 (s, 2 H), 3.82 (s, 3 H), 2.89 (ψt, *J*= 7.6 Hz, 2 H); ^{13}C NMR (100 MHz, $CDCl_3$): δ 186.7, 165.8, 160.9, 141.5, 133.5, 132.7, 127.9, 126.8, 124.5, 123.6, 116.9, 109.2, 57.4, 52.3, 40.6, 28.4; ^{13}C NMR (100 MHz, $CDCl_3$): δ 186.7, 165.8, 160.9, 141.5, 133.5, 132.7, 127.9, 126.8, 124.5, 123.6, 116.9, 109.2, 57.4, 52.3, 40.6, 28.4; HRMS (ESI): *m*/*z* calcd for $C_{16}H_{16}N_3O_4$ $[M+H]^+$ 314.1141, found 314.1138.

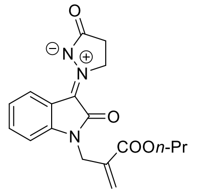

[Reaction time: 9 min]; **3k**: 239 mg, 70%, a white solid, m.p. 159.4-162.4 °C; IR (thin film): ν_{max} 3410, 3118, 2964, 2885, 1708, 1604, 1694, 1265, 1139, 762 cm^{-1}; ^{1}H NMR (400 MHz, $CDCl_3$): δ 8.43 (d, *J*=7.6 Hz, 1 H), 7.39 (m, 1 H), 7.15 (ψt, *J*=7.6 Hz, 1 H), 6.85 (d, *J*= 8.0 Hz, 1 H), 6.34 (s, 1 H), 5.59 (s, 1 H), 5.01 (t, *J*= 7.4 Hz, 2 H), 4.65 (s, 2H), 4.17 (t, *J*= 6.8 Hz, 2 H), 2.89 (t, *J*= 7.4 Hz, 2 H), 1.76-1.69 (m, 2 H), 0.89 (t, *J*= 7.4 Hz, 3 H); ^{13}C NMR (100 MHz, $CDCl_3$): δ 186.7, 165.4, 160.9, 141.6, 133.8, 132.6, 127.8, 126.4, 124.5, 123.6, 116.9, 109.2, 66.9, 57.4, 40.6, 28.4, 21.9, 10.4; HRMS (ESI): *m*/*z* calcd for $C_{18}H_{19}N_3O_4Na$ $[M+Na]^+$ 364.1273, found 364.1266.

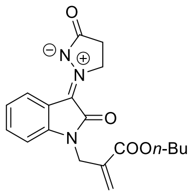

[Reaction time: 15 min]; **3l**: 142 mg, 40%, a yellow solid, m.p. 144.6-146.2 °C; IR (thin film): ν_{max} 3437, 2962, 2937, 2873, 1730, 1693, 1604, 1344, 1151, 755 cm^{-1}; ^{1}H NMR (400 MHz, $CDCl_3$): δ 8.44 (d, *J*= 7.6 Hz, 1 H), 7.38 (d, *J*= 8.0 Hz, 1 H), 7.15 (ψt, *J*= 7.4 Hz, 1 H), 6.86 (d, 1 H), 6.33 (s, 1 H), 5.59 (s, 1 H), 5.01 (t, *J*= 7.4 Hz, 2 H), 4.65 (s, 2 H), 4.22 (t, *J*= 6.6 Hz, 2 H), 2.89 (t, *J*= 7.6 Hz, 2 H), 1.71-1.64 (m, 2 H), 1.46-1.37 (m, 2H), 0.95 (t, *J*= 7.4 Hz, 3 H); ^{13}C NMR (100 MHz, $CDCl_3$): δ 186.7, 165.4, 160.9, 141.6, 133.8, 132.7, 127.9, 126.4, 124.6, 123.6, 116.9, 109.2, 65.2, 57.4, 40.6, 30.6, 28.5, 19.2, 13.7; HRMS (ESI): *m*/*z* calcd for $C_{19}H_{22}N_3O_4$ $[M+H]^+$ 356.1610, found 356.1601.

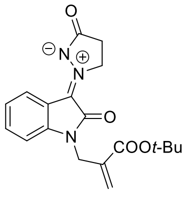

[Reaction time: 23 min]; **3m**: 199 mg, 56%, a yellow solid, m.p. 177.1-185.4 °C; IR (thin film): ν_{max} 3381, 3110, 3008, 2977, 2930, 1698, 1605, 1372, 1257, 1143, 758 cm^{-1}; ^{1}H NMR (400 MHz, $CDCl_3$): δ 8.44 (d, *J*= 7.6Hz, 1 H), 7.39 (td, *J*= 7.8, 1.2 Hz, 1 H), 7.15 (m, 1 H), 6.85 (d, *J*= 7.6 Hz, 1 H), 6.22 (s, 1 H), 5.45 (s, 1 H), 5.01 (t, *J*= 7.6 Hz, 2 H), 4.61 (s, 2 H), 2.89 (t, *J*= 7.6 Hz, 2 H), 1.52 (s, 9 H); ^{13}C NMR (100 MHz, $CDCl_3$): δ 186.7, 164.6, 160.9, 141.6, 135.1, 132.7, 127.9, 125.1, 124.6, 123.6, 116.9, 109.2, 81.9, 57.4, 40.6, 28.5, 28.1 (3 C). HRMS (ESI): *m*/*z* calcd for $C_{19}H_{22}N_3O_4$ $[M+H]^+$ 356.1610, found 356.1602.

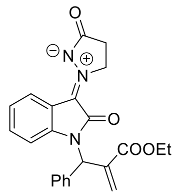

[Reaction time: 5 min]; **6a**: 169 mg, 42%, a yellow solid, m.p. 103.1-106.6 °C; IR (thin film): ν_{max} 3424, 2980, 1708, 1602, 1368, 1261, 1149, 750 cm^{-1}; ^{1}H NMR (400 MHz, $CDCl_3$): δ 8.49 (d, *J*= 7.2 Hz, 1 H), 7.39-7.36 (m, 1 H), 7.35-7.32 (m, 2 H), 7.30 (s, 1 H), 7.25 (dd, *J*= 7.8, 1.0 Hz, 1 H), 7.11 (m, 1 H), 6.81 (d, *J*= 8.0 Hz, 1 H), 6.62 (s, 1 H), 6.59 (d, *J*= 1.2 Hz, 1 H), 6.64 (d, *J*= 2.0 Hz, 1 H), 5.01 (ddd, *J*= 32.0, 16.4, 7.2 Hz, 2 H), 4.20-4.13 (m, 2 H), 2.88 (t, *J*= 7.6 Hz, 2 H), 1.16 (t, *J*= 7.2 Hz, 3 H); ^{13}C NMR (100 MHz, $CDCl_3$): δ 186.7, 165.4, 161.0, 141.9, 138.4, 136.0, 132.4, 129.3, 128.9, 128.3, 128.0, 127.9, 124.5, 123.3, 117.2, 110.9, 61.4, 57.5, 56.1, 28.4, 14.0; HRMS (ESI): *m*/*z* calcd for $C_{23}H_{22}N_3O_4$ $[M+H]^+$ 404.1610, found 404.1566.

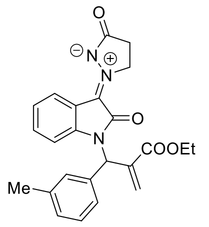

[Reaction time: 5 min]; **6c**: 213 mg, 56%, a yellow solid, m.p. 173.4-175.2 °C; IR (thin film): ν_{max} 3426, 2928, 1714, 1634, 1346, 1368, 1600, 1255, 745, 668 cm^{-1}; ^{1}H NMR (400 MHz, $CDCl_3$): δ 8.48 (d, *J*= 7.2 Hz, 1 H), 7.28-7.22 (m, 2 H), 7.14-7.08 (m, 4 H), 6.83 (d, *J*= 8.0 Hz, 1 H), 6.57 (s, 2 H), 5.62 (d, *J*= 1.6 Hz, 1 H), 5.09-4.95 (m, 2 H), 4.21-4.12 (m, 2 H), 2.88 (t, *J*= 7.6 Hz, 2 H), 2.23(s, 3 H) , 1.16 (t, *J*= 7.0 Hz, 3 H); ^{13}C NMR (100 MHz, $CDCl_3$): δ 186.7, 165.5, 161.0, 142.0, 138.7, 138.5, 136.0, 132.4, 129.1, 128.8, 128.6, 127.9, 124.9, 124.6, 123.3, 117.2, 111.0, 61.3, 57.4, 56.2, 29.7, 28.5, 21.5, 14.0; HRMS (ESI): *m*/*z* calcd for $C_{24}H_{24}N_3O_4$ $[M+H]^+$ 418.1767, found 418.1698.

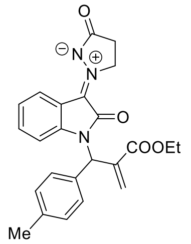

[Reaction time: 5 min]; **6d**: 154 mg, 37%, a yellow solid, m.p. 162.7-165.4 °C; IR (thin film): ν_{max} 3405, 2982, 1715, 1688, 1604, 1368, 1152, 746, 700 cm^{-1}; ^{1}H NMR (400 MHz, $CDCl_3$) δ 8.48 (d, *J*= 7.2 Hz, 1 H), 7.28-7.22 (m, 2 H), 7.14-7.08 (m, 4 H), 6.83 (d, *J*= 8.0 Hz, 1 H), 6.57 (s, 2 H), 5.62 (d, *J*= 1.6 Hz, 1 H), 5.09-4.95 (m, 2 H), 4.21-4.12 (m, 2 H), 2.88 (t, *J*= 7.6 Hz, 2 H), 2.32 (s, 3 H),1.16 (t, *J*= 7.0 Hz, 3 H); ^{13}C NMR (1 00 MHz, $CDCl_3$): δ 186.7, 165.5, 161.0, 142.0, 138.7, 138.5, 136.0, 132.4, 129.1, 128.8, 128.6, 127.9, 124.9, 124.6, 123.2, 117.2, 111.0, 61.3, 57.4, 56.2, 28.4, 28.1, 21.5, 14.0; HRMS (ESI): *m*/*z* calcd for $C_{24}H_{24}N_3O_4$ $[M+H]^+$ 418.1767, found 418.1744.

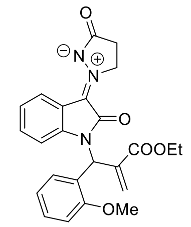

[Reaction time: 2h]; **6e**: 71 mg, 16%, a yellow solid, m.p. 153.4-155.7 °C; IR (thin film): ν_{max} 3408, 2978, 1709, 1567, 1461, 1258, 1154, 754 cm^{-1}; ^{1}H NMR (400 MHz, $CDCl_3$) δ 8.48 (dd, *J*= 7.6, 0.8 Hz, 1 H), 7.34-7.28 (m, 2 H), 7.25 (m, 1 H), 7.08 (td, *J*= 7.8, 0.8 Hz, 1 H), 6.94 (m, 1 H), 6.90 (d, *J*= 8.0 Hz, 1 H), 6.85 (d, *J*= 8.0 Hz, 1 H), 6.77 (s, 1 H), 6.51 (d, *J*= 1.2 Hz, 1 H), 5.56 (d, *J*= 1.2 Hz, 1 H), 5.66 (s, 1 H), 5.07-4.93 (m, 2 H), 4.19-4.09 (m, 2 H), 3.79 (s, 3 H), 2.87 (t, *J*= 7.2 Hz, 2 H), 1.15 (t, *J*= 7.2 Hz, 3 H); ^{13}C NMR (100 MHz, $CDCl_3$): δ 186.7, 165.5, 161.0, 156.9, 142.5, 137.6, 132.5, 129.8, 128.6, 127.9, 124.9, 124.2, 123.1, 120.6, 117.1, 110.8, 1110.7, 111.0, 61.2, 57.3, 55.6, 51.7, 28.5, 14.0; HRMS (ESI): *m*/*z* calcd for $C_{24}H_{23}N_3O_5Na$ $[M+Na]^+$ 456.1535, found 456.1527.

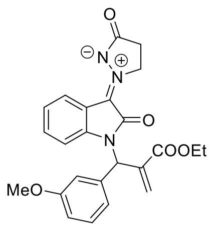

[Reaction time: 5 min]; **6f**: 277 mg, 64%, a yellow solid, m.p. 92.8-93.9 °C; IR (thin film): ν_{max} 3418, 2979, 2837, 1711, 1602, 1368, 1263, 750, 670 cm^{-1}; ^{1}H NMR (400 MHz, $CDCl_3$) δ 8.48 (dd, *J*= 8.0, 0.8 Hz, 1 H), 7.30-7.24 (m, 2 H), 7.10 (td, *J*= 7.8, 0.6 Hz, 1 H), 6.91-6.82 (m, 4 H), 6.59 (s, 2 H), 5.66 (s, 1 H), 5.08-4.94 (m, 2 H), 4.21-4.12 (m, 2 H), 3.76 (s, 3 H), 2.87 (t, *J*= 7.6 Hz, 1 H), 1.17 (t, *J*= 7.0 Hz, 3 H); ^{13}C NMR (100 MHz, $CDCl_3$): δ 186.7, 165.4, 161.0, 160.0, 141.9, 138.3, 137.6, 132.4, 130.0, 129.3, 127.9, 124.5, 123.3, 120.2, 117.2, 114.2, 113.2, 111.0, 61.3, 57.5, 56.0, 55.3, 28.4, 14.0; HRMS (ESI): *m*/*z* calcd for $C_{24}H_{24}N_3O_5$ $[M+H]^+$ 434.1716, found 434.1710.

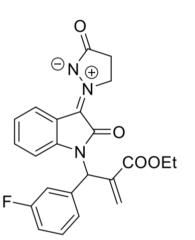

[Reaction time: 5 min]; **6i**: 328 mg, 71%, a yellow solid, m.p. 106.3-109.1 °C; IR (thin film): ν_{max} 3398, 2979, 2943, 1712, 1593, 1388, 1145, 751, 689 cm^{-1}; ^{1}H NMR (400 MHz, $CDCl_3$) δ 8.49 (d, *J*= 7.2 Hz, 1 H), 7.35-7.33 (m, 1 H), 7.29-7.25 (m, 1 H), 7.15-7.10 (m, 2 H), 7.05-7.01 (m, 2 H), 6.81 (d, *J*= 8.0 Hz, 1 H), 6.62 (s, 2 H), 5.67 (s, 1 H), 5.09-5.50 (m, 2 H), 4.23-4.12 (m, 2 H), 2.88 (t, *J*= 7.6 Hz, 2 H), 1.17 (t, *J*= 7.0 Hz, 3 H); ^{13}C NMR (100 MHz, $CDCl_3$): δ 186.7, 165.2, 161.0, 141.5, 138.7 (d), 137.8, 132.4, 130.6 (d), 129.5, 128.0, 124.2, 123.6 (d), 123.5, 117.3, 115.5, 115.3 (d), 115.0, 110.7, 61.5, 57.5, 55.6, 28.4, 14.0; ^{19}F NMR (376 MHz, DMSO-d_6): δ -111.5; HRMS (ESI): *m*/*z* calcd for $C_{23}H_{21}FN_3O_4$ $[M+H]^+$ 422.1516, found 422.1546.

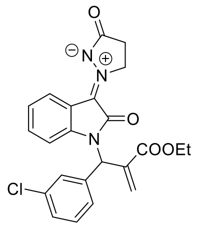

[Reaction time:8 min]; **6l**: 167 mg, 38%, a yellow solid, m.p. 96.1-98.3 °C; IR (thin film): ν_{max} 3426, 2980, 1710, 1593, 1385, 1149, 1026, 750, 686 cm^{-1}; ^{1}H NMR (400 MHz, $CDCl_3$) δ 8.50 (d, *J*= 7.2 Hz, 1 H), 7.32-7.28 (m, 4 H), 7.22-7.19 (m, 1 H), 7.14 (m, 1 H), 6.81 (d, *J*= 8.0 Hz, 1 H), 6.62 (d, *J*= 1.6 Hz, 1 H), 6.59 (s, 1 H), 5.66 (d, *J*= 1.6 Hz, 1 H), 5.09-4.95 (m, 2 H), 4.23-4.11 (m, 2 H), 2.88 (t, *J*= 7.6 Hz,2 H), 1.17 (t, *J*= 7.2 Hz, 3 H); ^{13}C NMR (100 MHz, $CDCl_3$): δ 186.7, 165.2, 161.0, 141.4, 138.2, 137.7, 134.9, 132.4, 130.2, 129.6, 128.6, 128.1, 128.0, 126.1, 124.2, 123.5, 117.3, 110.7, 61.5, 57.5, 55.6, 28.4, 14.0; HRMS (ESI): *m/z* calcd for $C_{23}H_{21}ClN_3O_4$ $[M+H]^+$ 438.1221, found 438.1222.

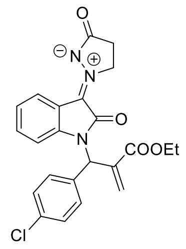

[Reaction time: 5 min]; **6m**: 188 mg, 43%, a yellow solid, m.p. 111.4-111.5 °C; IR (thin film): ν_{max} 3423, 2926, 1712, 1603, 1262, 1150, 1014, 750, 678 cm^{-1}; ^{1}H NMR (400 MHz, $CDCl_3$) δ 8.49 (d, *J*= 7.6 Hz, 1 H), 7.35-7.33 (m, 2 H), 7.29-7.24 (m, 4 H), 7.13 (ψt, *J*= 7.2 Hz, 1 H), 6.78 (d, *J*= 8.0 Hz, 1 H), 6.59 (s, 1 H), 5.65 (d, *J*= 1.6 Hz, 1 H), 5.06-4.96 (m, 2 H), 4.21-4.11 (m, 2 H), 2.88 (ψt, *J*= 7.4 Hz, 2 H), 1.17 (t, *J*= 7.2 Hz, 3 H); ^{13}C NMR (100 MHz, $CDCl_3$): δ 186.7, 165.2, 161.0, 141.5, 138.0, 134.6, 134.3, 132.4, 129.3, 129.2, 129.0, 128.0, 123.5, 117.3, 110.7, 61.5, 57.5, 55.5, 29.7, 28.4, 14.0; HRMS (ESI): *m/z* calcd for $C_{23}H_{20}ClN_3O_4Na$ $[M+Na]^+$ 460.1040, found 460.1024.

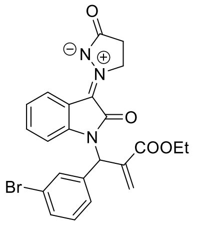

[Reaction time: 10 min]; **6o**: 265 mg, 55%, a yellow solid, m.p. 101.1-104.3 °C; IR (thin film): ν_{max} 3421, 2979, 1709, 1593, 1368, 1149, 771, 682 cm^{-1}; ^{1}H NMR (400 MHz, $CDCl_3$) δ 8.50 (d, *J*= 7.6 Hz, 1 H), 7.48-7.46 (m, 2 H), 7.31-7.22 (m, 4 H), 7.13 (ψt, *J*= 7.8 Hz, 1 H), 6.81 (d, *J*= 8.0 Hz, 1 H), 6.62 (s, 1 H), 6.58 (s, 1 H), 5.66 (d, *J*= 1.2 Hz,1 H), 5.09-4.95 (m, 2 H), 4.23-4.11 (m, 2 H), 2.88 (t, *J*= 7.4 Hz, 2 H), 1.17 (t, *J*= 7.0 Hz, 3 H); ^{13}C NMR (100 MHz, $CDCl_3$): δ 186.7, 165.2, 161.0, 141.4, 138.5, 137.7, 132.4, 131.6, 131.0, 130.4, 129.6, 128.1, 126.5, 124.2, 123.5, 123.0, 117.3, 110.7, 61.5, 57.6, 55.5, 28.4, 14.0; HRMS (ESI): *m/z* calcd for $C_{23}H_{21}BrN_3O_4$ $[M+H]^+$ 482.0715, found 482.0714.

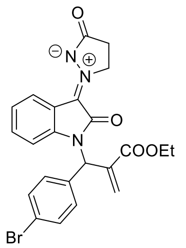

[Reaction time: 5 min]; **6p**: 216 mg, 45%, a yellow solid, m.p. 112.7-114.8 °C; IR (thin film): ν_{max} 3568, 3425, 2979, 1704, 1596, 1371, 1270, 1071, 749, 682 cm^{-1}; ^{1}H NMR (400 MHz, $CDCl_3$) δ 8.49 (d, *J*= 7.2 Hz, 1 H), 7.49 (d, *J*= 8.4 Hz, 2 H), 7.27 (m, 1 H), 7.19 (d, *J*= 8.4 Hz, 2 H), 7.12 (m, 1 H), 6.78 (d, *J*= 8.0 Hz, 1 H), 6.60 (d, *J*= 1.2 Hz, 1 H), 6.58 (s, 1 H), 5.56 (d, *J*= 1.6 Hz, 1 H), 5.08-4.94 (m, 2 H), 4.23-4.11 (m, 2 H), 2.88 (t, *J*= 7.6 Hz, 2 H), 1.17 (t, *J*= 27.2 Hz, 3 H);^{13}C NMR (100 MHz, $CDCl_3$): δ 186.7, 165.2, 161.0, 141.5, 137.9, 135.2, 132.4, 132.1, 129.6, 129.4, 128.0, 124.2, 123.5, 122.4, 117.3, 110.7, 61.5, 57.5, 55.6, 28.4, 14.0; HRMS (ESI): *m/z* calcd for $C_{23}H_{21}BrN_3O_4$ $[M+H]^+$ 482.0715, found 482.0715.

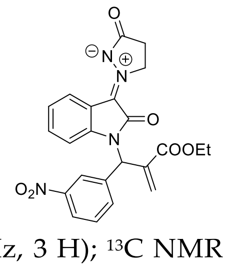

[Reaction time: 2 min]; **6r**: 211 mg, 47%, a yellow solid, m.p. 99.6-101.7 °C; IR (thin film): ν_{max} 3400, 2979, 2927, 1709, 1567, 1349, 1150, 748 cm^{-1}; ^{1}H NMR (400 MHz, $CDCl_3$) δ 8.51 (d, *J*= 7.6 Hz, 1 H), 8.21 (d, *J*= 8.8 Hz, 1 H), 8.20 (s, 1 H), 7.68 (d, *J*= 8.0 Hz, 1 H), 7.58 (ψt, *J*= 7.6 Hz, 1 H), 7.30 (m, 1 H), 7.15 (t, *J*= 7.6 Hz, 1 H), 6.80 (d, *J*= 8.0 Hz, 1 H), 6.72 (s, 1 H), 6.68 (d, *J*= 1.2 Hz, 1 H), 5.70 (d, *J*= 1.6 Hz, 1 H), 5.04-4.99 (m, 2 H), 4.24-4.15 (m, 2 H), 2.89 (t, *J*= 7.6 Hz, 2 H), 1.2 (t, *J*= 7.2 Hz, 3 H); ^{13}C NMR (100 MHz, $CDCl_3$): δ 186.6, 165.0, 161.0, 148.6, 140.9, 138.5, 137.1, 134.0, 132.4, 130.0, 129.9, 128.1, 123.8, 123.4, 123.0, 117.4, 110.3, 61.7, 57.7, 55.4, 28.3, 14.0; HRMS (ESI): *m/z* calcd for $C_{23}H_{21}N_4O_6$ $[M+H]^+$449.1461, found 449.1450.

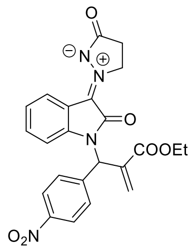

[Reaction time: 2 min]; **6s**: 242 mg, 54%, a yellow solid, m.p. 199.7-122.4 ^{o}C; IR (thin film): ν_{max} 3418, 2982, 1716, 1633, 1606, 1299, 1147, 750, 696 cm^{-1}; ^{1}H NMR (400 MHz, $CDCl_3$): δ 8.51 (d, *J*= 7.6 Hz, 1 H), 8.23 (d, *J*= 8.8 Hz, 2 H), 7.51 (d, *J*= 8.8 Hz, 2 H), 7.32-7.27 (m, 1 H), 7.16 (t, *J*= 7.6 Hz, 1 H), 6.79 (d, *J*= 7.6 Hz, 1 H), 6.73 (s, 1 H), 6.68 (s, 1 H), 5.69 (d, *J*= 1.6 Hz, 1 H), 5.08-4.95 (m, 2 H), 4.24-4.15 (m, 2 H), 2.89 (t, *J*= 7.6 Hz, 2 H), 1.20 (t, *J*= 7.2 Hz, 3 H); ^{13}C NMR (100 MHz, $CDCl_3$): δ 186.6, 165.0, 161.0, 147.7, 143.5, 141.0, 137.1, 132.4, 130.0, 129.0, 128.9, 128.1, 124.1, 123.8, 123.7, 117.4, 110.4, 61.7, 57.7, 55.4, 29.7, 28.3, 14.0; HRMS (ESI): *m*/*z* calcd for $C_{23}H_{21}N_4O_6$ [M+H]$^{+}$449.1461, found 449.1450

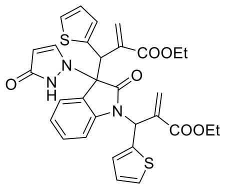

[Reaction time: 5 min]; **6't**: 156 mg, 34%, a white solid, m.p. 199.7-200.9 ^{o}C, *dr> 20:1*; IR (thin film): ν_{max} 3410, 3126, 3923, 2851, 1734,1713, 1610, 1300, 1157, 760 cm^{-1}; ^{1}H NMR (400 MHz, DMSO-*d*$_6$): δ 9.84 (s, 1H), 7.63 (d, *J*= 7.2 Hz, 1 H), 7.46 (dd, *J*= 4.8, 2.8 Hz, 1 H), 7.40 (ψs, 1 H), 7.30 (dd, *J*= 4.8, 2.8 Hz, 1 H), 7.25 (d, *J*= 7.2 Hz, 1 H), 7.15-7.11 (m, 2 H), 7.00 (ψt, *J*= 7.6-7.2 Hz, 1 H), 6.89 (dd, *J*= 5.2, 0.8 Hz, 1 H), 6.86 (dd, *J*= 4.8, 0.8 Hz, 1 H), 6.57-6.53 (m, 3 H), 6.46 (s, 1 H), 5.78 (s, 1 H), 5.57 (d, *J*= 2.4 Hz, 1 H), 4.12-4.01 (m, 2 H), 3.83 (d, *J*= 12.8 Hz, A of AB, 1 H), 3.80-3.72 (m, 1 H), 3.55-3.47 (m, 1 H), 3.42 (d, *J*= 12.8 Hz, B of AB, 1H), 1.06 (t, *J*= 7.2 Hz, 3H), 0.90 (t, *J*= 7.2 Hz, 3H); ^{13}C NMR (100 MHz, DMSO-*d*$_6$): δ 172.5, 168.3, 165.5, 162.0, 142.5, 137.6, 136.2, 136.1, 133.2, 130.2, 129.9, 129.5, 128.2, 127.9, 127.8, 127.5, 127.1, 126.7, 125.7, 124.3, 124.1, 122.6, 122.0, 92.3, 79.7,68.2, 61.5, 61.0, 51.9, 14.1, 13.7; HRMS (ESI): *m*/*z* calcd for $C_{31}H_{30}N_3O_6S_2$ [M+H]$^{+}$ 604.1576, found 604.1461.

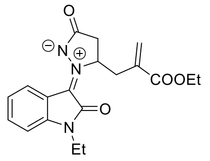

[Reaction time: 12 h]; **8b**: 171 mg, 48%, a yellow solid, m.p. 202.1-204.0 ^{o}C; IR (thin film): ν_{max} 3404, 2984, 2941, 1705, 1687, 1604, 1373, 1156, 1127, 779, 667 cm^{-1}; ^{1}H NMR (400 MHz, $CDCl_3$): δ 8.46 (d, *J*= 7.6 Hz, 1 H), 7.42 (ψt, *J*= 7.6 Hz, 1 H), 7.13 (ψt, *J*= 7.6 Hz, 1 H), 6.88 (d, *J*= 8.0 Hz, 1 H), 6.36 (s, 1 H), 6.07 (dd, *J*= 15.6, 6.4 Hz, 1 H), 5.74 (s, 1 H), 4.23-4.15 (m, 2 H), 3.89-3.79 (m, 2 H), 2.96-2.90 (m, 3 H), 2.68 (dd, *J*= 16.8, 1.2 Hz, 1 H), 1.33-1.27 (m, 6 H); ^{13}C NMR (100 MHz, $CDCl_3$): δ 185.3, 166.1, 160.0, 142.0, 135.2, 132.8, 129.1, 128.6, 123.3, 122.5, 117.3, 68.2, 61.3, 37.3, 35.0, 34.6, 14.1, 13.0 ; HRMS (ESI): *m*/*z* calcd for $C_{19}H_{22}N_3O_4$ [M+H]$^{+}$ 356.1610, found 356.1612.

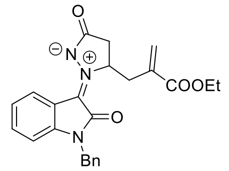

[Reaction time: 7 h]; **8c**: 313 mg, 74%, a red solid, m.p. 163.2-164.8 ^{o}C; IR (thin film): ν_{max} 3419, 2979, 2931, 1703, 1692, 1608, 1595, 1386, 1158, 1140, 756, 661 cm^{-1}; ^{1}H NMR (400 Hz, $CDCl_3$): δ 8.45 (d, *J*= 7.2 Hz, 1 H), 7.36-7.27(m, 6 H), 7.10 (td, *J*= 8.0-7.6 Hz, 1 H), 6.77 (d, *J*= 8.0 Hz, 1 H), 6.37 (s, 1 H), 6.15-6.09 (m, 1 H), 5.74 (s, 1 H), 5.02 (d, *J*= 16.0 Hz, A of AB, 1 H), 4.97 (d, *J*= 16.0 Hz, B of AB, 1 H), 4.22-4.14 (m, 2 H), 3.06-2.92 (m, 3 H), 2.70 (dd, *J*= 16.8, 1.6 Hz, 1 H), 1.27 (t, *J*= 7.2 Hz, 3 H); ^{13}C NMR (100 MHz, $CDCl_3$): δ 185.3, 166.1, 160.5, 142.1, 135.1, 132.8, 129.3, 129.0, 128.7, 128.5, 128.0, 127.2, 125.7, 123.5, 117.3, 109.2, 68.3, 61.3, 43.9, 37.4, 34.7, 14.1; HRMS (ESI): *m*/*z* calcd for $C_{24}H_{24}N_3O_4$ [M+H]$^{+}$ 418.1767, found 418.1760.

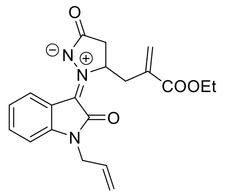

[Reaction time: 8 h]; **8d**: 323 mg, 88%, a yellow solid, m.p. 124.2-125.6 ^{o}C; IR (thin film): ν_{max} 3402, 2981, 2925, 1703, 1607, 1589, 1384, 1261, 1026, 759, 687 cm^{-1}; ^{1}H NMR (400 MHz, $CDCl_3$): δ 8.46 (d, *J*= 7.6 Hz, 1 H), 7.39 (ψt, *J*= 7.6 Hz, 1 H), 7.13 (ψt, *J*= 7.6 Hz, 1 H), 6.86 (d, *J*= 7.6 Hz, 1 H), 6.36 (s, 1 H), 6.07 (dd, *J*= 7.6, 6.8 Hz, 1 H), 5.91-5.81 (m, 1 H), 5.74 (s, 1 H), 5.28 (d, *J*= 2.8 Hz, 1 H), 5.25 (s, 1 H), 4.42 (d, *J*= 4.0 Hz, 2 H), 4.24-4.12 (m, 2 H), 3.02-2.91 (m, 3 H), 2.68 (d, *J*= 16.8 Hz, B of AB, 1 H), 1.28 (t, *J*= 7.2 Hz, 3 H); ^{13}C NMR (100 MHz, $CDCl_3$): δ 185.3, 166.1, 160.0, 142.1, 135.1, 132.8, 130.8,

129.2, 128.4, 123.4, 118.1, 117.7, 117.2, 109.1, 68.3, 61.3, 42.4, 37.3, 34.5, 14.1; HRMS (ESI): *m/z* calcd for $C_{20}H_{22}N_3O_4$ $[M+H]^+$ 368.1610, found 368.1596.

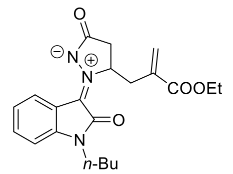

[Reaction time: 12 h]; **8e**: 71 mg, 20%, a yellow solid, m.p. 128.2-131.6 °C; IR (thin film): ν_{max} 3273, 2969, 2928, 1706, 1607, 1563, 1314, 1266, 1174, 749 cm^{-1}; ^{1}H NMR (400 MHz, $CDCl_3$): δ 8.47 (d, *J*= 7.4 Hz, 1 H), 7.46 (ψt, *J*= 7.6 Hz, 1 H), 7.18 (ψt, J= 7.6 Hz, 1 H), 7.08 (d, *J*= 8.0 Hz, 1 H), 6.37 (s, 1 H), 6.07-6.02 (m, 1 H), 5.75 (s, 1 H), 4.63 (dd, *J*= 18.0, 2.4 Hz, A of AB, 1 H), 4.58 (dd, *J*= 18.0, 2.4 Hz, B of AB, 1 H), 4.23-4.14 (m, 2 H), 3.01-2.89 (m, 3 H), 2.69 (d, *J*= 16.8 Hz, B′ of A′B′, 1 H), 2.30 (t, *J*= 2.4 Hz, 3 H), 1.29 (t, *J*= 7.2 Hz, 3 H); ^{13}C NMR (100 MHz, $CDCl_3$): δ 185.3, 166.1, 159.4, 140.9, 135.0, 132.7, 129.4, 128.4, 125.0, 123.8, 117.4, 109.2, 76.2, 72.9, 68.9, 61.4, 37.3, 34.4, 29.3 14.1; HRMS (ESI): m/z calcd for $C_{20}H_{20}N_3O_4$ $[M+H]^+$ 366.1454, found 366.1451.

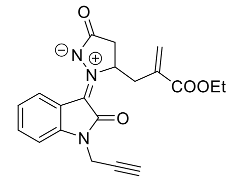

[Reaction time: 8 h]; **8f**: 277 mg, 72%, a yellow solid, m.p. 120.1-122.2 °C; IR (thin film): ν_{max} 3423, 2931, 2856, 1721, 1619, 1607, 1374, 1293, 1130, 754 cm^{-1}; ^{1}H NMR (400 MHz, $CDCl_3$): δ 8.46 (d, *J*= 7.6 Hz, 1 H), 7.41 (ψt, *J*= 7.4 Hz, 1 H), 7.12 (ψd, *J*= 7.6 Hz, 1 H), 6.87 (d, *J*= 8.0 Hz, 1 H), 6.36 (s, 1 H), 6.06 (dd, *J*= 13.2, 6.4 Hz, 1 H), 5.74 (s, 1 H), 4.22-4.14 (m, 2 H), 3.84-3.71 (m, 2 H), 2.96-2.90 (m, 3 H), 2.69 (dd, *J*= 16.4, 1.2 Hz, B of AB, 1 H), 1.73-1.66 (m, 2 H), 1.46-1.37 (m, 2 H), 1.38 (t, *J*= 7.2 Hz, 3 H), 0.97 (t, *J*= 7.4 Hz, 3 H); ^{13}C NMR (100 MHz, $CDCl_3$): δ 185.3, 166.1, 160.2, 142.4, 135.2, 132.8, 129.1, 128.6, 125.9, 123.2, 117.3, 108.5, 68.2, 61.3, 40.0, 37.3, 34.6, 29.7, 20.2, 14.1, 13.7; HRMS (ESI): *m/z* calcd for $C_{21}H_{26}N_3O_4$ $[M+H]^+$ 384.1923, found 384.1929.

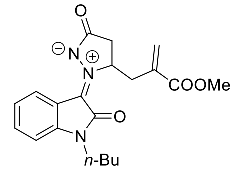

[Reaction time: 4 h]; **8g**: 217 mg, 55%, a yellow solid, m.p. 97.2-99.0 °C; IR (thin film): ν_{max} 3380, 2954, 2873, 1700, 1606, 1378, 1286, 1128, 754 cm^{-1}; ^{1}H NMR (600 MHz, $CDCl_3$): δ 8.45 (dd, *J*= 7.6, 0.4 Hz, 1 H), 7.41 (m, 1.2 Hz, 1 H), 7.12 (td, *J*= 7.6, 0.4 Hz, 1 H), 6.87 (d, *J*= 8.0 Hz, 1 H), 6.36 (s, 1 H), 6.10-6.04 (m, 1 H), 5.74 (s, 1 H), 3.84-3.73 (m, 2 H), 3.72 (s, 3 H), 3.01-2.91 (m, 3 H), 2.67 (dd, *J*= 16.4, 1.6 Hz, B of AB, 1 H), 1.75-1.66 (m, 2 H), 1.46-1.37 (m, 2 H), 0.98 (t, *J*= 7.4 Hz, 3 H); ^{13}C NMR (100 MHz, $CDCl_3$): δ 185.2, 166.5, 160.2, 142.4, 134.8, 132.8, 129.5, 128.6, 126.0, 123.2, 117.3, 108.5, 68.1, 52.3, 40.0, 37.4, 34.6, 29.7, 20.2, 13.7; HRMS (ESI): *m/z* calcd for $C_{20}H_{24}N_3O_4$ $[M+H]^+$ 370.1767, found 370.1755.

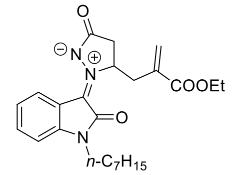

[Reaction time: 10 h]; **8h**: 349 mg, 82%, a red solid, m.p. 106.1-107.3 °C; IR (thin film): ν_{max} 3410, 2959, 2873, 1698, 1608, 1362, 1252, 1127, 753 cm^{-1}; ^{1}H NMR (400 MHz, $CDCl_3$): δ 8.45 (d, *J*= 7.6 Hz, 1 H), 7.41 (m, 1 H), 7.12 (m, 1 H), 6.87 (d, *J*= 8.0 Hz, 1 H), 6.36 (s, 1 H), 6.07 (dd, *J*= 13.0, 6.6 Hz, 1 H), 5.74 (d, *J*= 0.4 Hz, 1 H), 4.24-4.14 (m, 2 H), 3.83-3.70 (m, 2 H), 2.96-2.90 (m, 3 H), 2.68 (dd, *J*= 17.0, 1.4 Hz, B of AB, 1 H), 1.76-1.66 (m, 2 H), 1.36-1.27 (m, 8 H), 1.28 (t, *J*= 7.2 Hz, 3 H), 0.88 (t, *J*= 6.8 Hz, 3 H); ^{13}C NMR (100 MHz, $CDCl_3$): δ 185.3, 166.1, 160.2, 142.4, 135.1, 132.8, 129.1, 128.5, 125.9, 123.2, 117.3, 108.5, 68.2, 61.3, 40.3, 37.3, 34.5, 31.7, 28.9, 27.7, 26.9, 22.6, 14.1, 14.0; HRMS (ESI): *m/z* calcd for $C_{24}H_{32}N_3O_4$ $[M+H]^+$ 426.2393, found 426.2397.

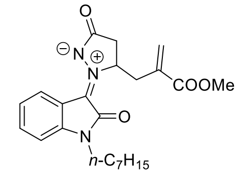

[Reaction time: 10 h]; **8i**: 305 mg, 74%, a yellow solid, m.p. 103.1-104.2 °C; IR (thin film): ν_{max} 3369, 2953, 2855, 1715, 1693, 1606, 1370, 1295, 1132, 752 cm^{-1}; ^{1}H NMR (400 MHz, $CDCl_3$): δ 8.45 (d, *J*= 7.2 Hz, 1 H), 7.41 (m, 1 H), 7.12 (ψt, *J*= 7.6 Hz, 1 H), 6.87 (d, *J*= 8.0 Hz, 1 H), 6.36 (s, 1 H), 6.10-6.04 (m, 1 H), 5.74 (s, 1 H), 3.83-7.32 (m, 2 H), 3.72 (s, 3 H), 3.01-2.91 (m, 3 H), 2.67 (dd, *J*= 16.4, 1.6 Hz, B of AB, 1 H), 1.76-1.67 (m, 2 H), 1.36-1.28 (m, 8 H), 0.88 (t, *J*= 6.8 Hz, 3 H); ^{13}C NMR (100 MHz, $CDCl_3$): δ 185.2, 166.5, 160.2, 142.4, 134.8, 132.8, 129.5, 128.6, 126.0, 123.2, 117.3, 108.5, 68.1, 52.3, 40.3, 37.4,

34.6, 31.7, 28.9, 27.7, 26.9, 22.6, 14.0; HRMS (ESI): *m/z* calcd for $C_{23}H_{30}N_3O_4$ [M+H]$^+$ 412.2236, found 412.2233.

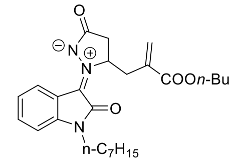

[Reaction time: 8 h]; **8j**: 349 mg, 77%, a yellow solid, m.p. 96.2-97.7 °C; IR (thin film): ν_{max} 3418, 2955, 2871, 1718, 1694, 1607, 1294, 1130, 754 cm^{-1}; ^{1}H NMR (400 MHz, CDCl$_3$): δ 8.46 (d, *J*= 7.6 Hz, 1 H), 7.41 (m, 1 H), 7.12 (m, 1 H), 6.87 (d, *J*= 8.0 Hz, 1 H), 6.36 (s, *J*= 7.2 Hz, 1 H), 6.09-6.03 (m, 1 H), 5.74 (s, 1 H), 4.18-4.08 (m, 2 H), 3.84-3.70 (m, 2 H), 2.96-2.90 (m, 3 H), 2.68 (dd, *J*= 16.8, 1.6 Hz, 1 H), 1.76-1.60 (m, 5 H), 1.43-1.26 (m, 10 H), 0.93 (t, *J*= 7.6-7.2 Hz, 3 H), 0.88 (t, *J*= 6.8 Hz, 3 H); ^{13}C NMR (1 00 MHz, CDCl$_3$): δ 185.2, 166.2, 160.2, 142.4, 135.2, 132.8, 129.1, 128.6, 125.9, 123.2, 117.3, 108.5, 68.2, 65.2, 40.3, 37.3, 34.5, 31.7, 30.5, 28.9, 27.7, 26.9, 22.6, 19.2, 14.0, 13.7; HRMS (ESI): *m/z* calcd for $C_{26}H_{36}N_3O_4$ [M+H]$^+$ 454.2706, found 454.2704.

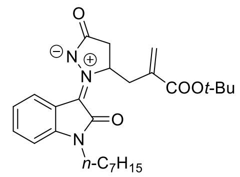

[Reaction time: 8 h]; **8k**: 358 mg, 79%, a yellow solid, m.p. 106.9-108.2 °C; IR (thin film): ν_{max} 3374, 3242,2955, 2859,1696, 1592, 1294, 1030, 757 cm^{-1}; ^{1}H NMR (400 MHz, CDCl$_3$): δ 8.45 (d, *J*= 8.0 Hz, 1 H), 7.40 (ψt, *J*= 7.6 Hz, 1 H), 7.11 (ψt, *J*= 7.6 Hz, 1 H), 6.86 (d, *J*= 8.0 Hz, 1 H), 6.26 (s, 1 H), 6.04-5.99 (m, 1 H), 5.67 (s, 1 H), 3.83-3.70 (m, 2 H), 3.01-2.84 (m, 3 H), 2.69 (d, *J*= 16.4 Hz, B of AB, 1 H), 1.72-1.60 (m, 2 H), 1.45 (s, 9 H), 1.35-1.28 (m, 8 H), 0.87 (t, *J*= 6.6 Hz, 3 H); ^{13}C NMR (100 MHz, CDCl$_3$): δ 185.3, 165.3, 160.2, 142.4, 136.4, 132.7, 128.6, 128.3, 125.9, 123.1, 117.3, 108.4, 81.5, 77.2, 68.4, 40.3, 36.8, 34.5, 31.7, 28.9, 28.0, 27.9, 27.7, 26.9, 22.6, 14.0; HRMS (ESI): *m/z* calcd for $C_{26}H_{36}N_3O_4$ [M+H]$^+$ 454.2706, found 454.2701.

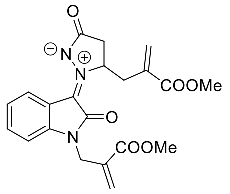

[Reaction time: 8 h]; **4a**: 175 mg, 44%, a yellow solid, m.p. 130.7-131.6 °C; IR (thin film): ν_{max} 2999, 2951, 1708, 1628, 1593, 1306, 1167, 685 cm^{-1}; ^{1}H NMR (400 MHz, CDCl$_3$): δ 8.47 (d, *J*= 7.2 Hz, 1 H), 7.39 (m, 1 H), 7.15 (m, 1 H), 6.84 (d, *J*= 7.6 Hz, 1 H), 6.35 (s, 2 H), 6.09-6.05 (m, 1 H), 5.75 (s, 1 H), 5.64 (s, 1 H), 4.67 (s, 2 H), 3.83 (s, 3 H), 3.69 (s, 3 H), 3.05 (dd, *J*= 14.0, 8.0 Hz, 1 H, A of AB), 2.98-2.88 (m, 2 H), 2.7.0 (dd, *J*= 16.8, 1.6 Hz, 1 H, B of AB); ^{13}C NMR (100 MHz, CDCl$_3$): δ 185.2, 166.5, 165.8, 160.4, 141.6, 134.6, 133.4, 132.9, 129.7, 128.4, 126.6, 125.4, 123.6, 117.3, 109.1, 68.3, 52.3 (2C), 40.5, 37.2, 34.5; HRMS (ESI): *m/z* calcd for $C_{21}H_{21}N_3NaO_6$ [M+Na]$^+$ 434.1328, found 434.1310.

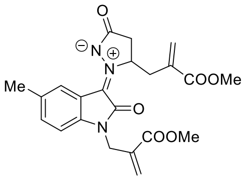

[Reaction time: 6 h]; **4b**: 172 mg, 41%, a yellow solid, m.p. 132.4-133.7 °C; IR (thin film): ν_{max} 3403, 2996, 2949, 1710, 1612, 1596, 1147, 686 cm^{-1}; ^{1}H NMR (400 MHz, CDCl$_3$): δ 8.32 (s, 1 H), 7.21 (d, *J*= 8.0 Hz, 1 H), 6.73 (d, *J*= 8.0 Hz, 1 H), 6.36 (s, 1 H), 6.35 (s, 1 H), 6.12-6.07 (m, 1 H), 5.76 (s, 1 H), 5.62 (s, 1 H), 4.65 (s, 2 H), 3.83 (s, 3 H), 3.71 (s, 3 H), 3.06-2.88 (s, 3 H), 2.69 (dd, *J*= 16.8, 1.2 Hz, 1 H, B of AB), 2.37 (s, 3 H); ^{13}C NMR (100 MHz, CDCl$_3$): δ 185.3, 166.5, 165.9, 160.4, 139.5, 134.7, 133.5 (2C), 133.4, 129.6, 128.9, 126.5, 125.9, 117.2, 108.9, 68.2, 52.3 (2C), 40.5, 37.3, 34.6, 21.0; HRMS (ESI): *m/z* calcd for $C_{22}H_{23}N_3NaO_6$ [M+Na]$^+$ 448.1485, found 448.4179.

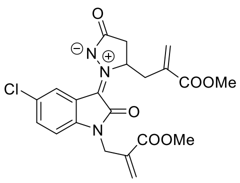

[Reaction time: 7 h]; **4c**: 163 mg, 37%, a yellow solid, m.p. 129.7-131.6 °C; IR (thin film): ν_{max} 3408, 2950, 1712, 1632, 1605, 1149, 1123, 679 cm^{-1}; ^{1}H NMR (400 MHz, CDCl$_3$): δ 8.48 (d, *J*= 2.0 Hz, 1 H), 7.36 (dd, *J*= 10.4, 8.4 Hz, 1 H), 6.82 (d, *J*= 8.4 Hz, 1 H), 6.38 (s, 1 H), 6.37 (s, 1 H), 6.12-6.07 (m, 1 H), 5.77 (s, 1 H), 5.66 (s, 1 H), 4.67 (s, 2 H), 3.83 (s, 3 H), 3.71 (s, 3 H), 3.08 (dd, *J*= 14.0, 7.6 Hz, 1 H, A of AB), 3.00-2.87(m, 2 H), 2.71 (dd, *J*= 16.8, 1.2 Hz, 1 H, B′ of A′B′); ^{13}C NMR (100 MHz, CDCl$_3$): δ 185.2, 166.5, 165.8, 160.1, 139.8, 134.4, 133.2, 132.2, 129.9, 129.2, 127.7, 127.0, 124.1, 118.4, 110.1, 68.7, 52.4 (2C), 40.7, 37.2, 34.3; HRMS (ESI): *m/z* calcd for $C_{21}H_{20}ClN_3NaO_6$ [M+Na]$^+$ 468.0938, found 468.0922.

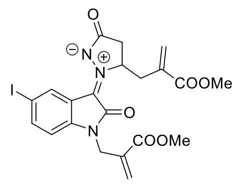

[Reaction time: 6 h]; **4d**: 263 mg, 49%, a red solid, m.p. 157.2-158.7 ^{o}C; IR (thin film): ν_{max} 3395, 3004, 2956, 1712, 1630, 1599, 1178, 1137, 675, 640 cm^{-1}; ^{1}H NMR (400 MHz, $CDCl_3$): δ 8.77 (d, *J*= 1.6 Hz, 1 H), 7.71 (dd, *J*= 8.0, 1.6 Hz, 1 H), 6.67 (d, *J*= 8.0 Hz, 1 H), 6.37 (s, 1 H), 6.36 (s, 1 H), 6.13-6.07 (m, 1 H), 5.77 (s, 1 H), 5.66 (s, 1 H), 4.66 (s, 2 H), 3.83 (s, 3 H), 3.72 (s, 3 H), 3.06-2.89 (s, 3 H),2.71 (dd, *J*= 16.8, 1.6 Hz, 1 H, B of AB); ^{13}C NMR (100 MHz, $CDCl_3$): δ 185.2, 166.5, 165.8, 159.8, 141.1, 140.9, 135.8, 134.4, 133.2, 129.9, 127.0, 123.7, 119.0, 111.1, 86.2, 68.7, 52.4, 40.6, 37.2, 34.4; HRMS (ESI): *m/z* calcd for $C_{21}H_{20}IN_3NaO_6$ [M+Na]$^+$ 560.0294, found 560.0277.

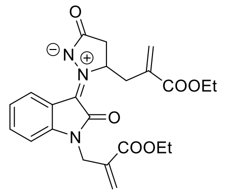

[Reaction time: 7 h]; **4e**: 338 mg, 77%, a yellow oil; IR (thin film): ν_{max} 2988, 2928, 1713, 1600, 1303, 1153, 683 cm^{-1}; ^{1}H NMR (400 MHz, $CDCl_3$): δ 8.47 (d, *J*= 7.2 Hz, 1 H), 7.39 (m, 1 H), 7.14 (ψt, *J*= 7.6 Hz, 1 H), 6.85 (d, *J*= 7.6 Hz, 1 H), 6.35 (s, 1 H), 6.34 (s, 1 H), 6.10-6.05 (m, 1 H), 5.74 (s, 1 H), 5.61 (s, 1 H), 4.67 (s, 2 H), 4.28 (q, *J*= 7.2 Hz, 2 H), 4.20-4.10 (m, 2 H), 3.04 (dd, *J*= 14.0, 8.0 Hz, A of AB, 1 H), 2.95 (dd, *J*= 16.8, 8.4 Hz, A′ of A′B′, 1 H), 2.91 (dd, *J*= 14.0, 4.4 Hz, B of AB, 1 H) , 2.69 (dd, *J*= 16.8, 1.2 Hz, B′ of A′B′, 1 H), 1.33 (t, *J*= 7.2 Hz, 3 H), 1.27 (t, *J*= 7.2 Hz, 3H); ^{13}C NMR (100 MHz, $CDCl_3$): δ 185.2, 166.1, 165.4, 160.4, 141.7, 135.0, 133.7, 132.8, 129.3, 128.4, 126.3, 125.3, 123.6, 117.3, 109.1, 77.2, 68.4, 61.3, 40.5, 37.2, 34.5, 14.2, 14.1; HRMS (ESI): *m/z* calcd for $C_{23}H_{26}N_3O_6$ [M+H]$^+$ 440.1822, found 440.1823.

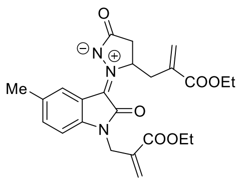

[Reaction time: 7 h]; **4f**: 225 mg, 50%, a yellow oil; IR (thin film): ν_{max} 2927, 2856, 1607, 1562, 1480, 1366, 1299, 1131, 696 cm^{-1}; ^{1}H NMR (400 MHz, $CDCl_3$): δ 8.32 (s, 1 H), 7.19 (d, *J*= 7.6 Hz, 1 H), 6.73 (d, *J*= 8.0 Hz, 1 H), 6.35 (s, 1 H), 6.33 (s, 1 H), 6.10-6.05 (m, 1 H), 5.74 (s, 1 H), 5.59 (s, 1 H), 4.64 (s, 2 H), 4.27 (q, *J*= 7.2 Hz, 2 H), 4.21-4.13 (m, 2 H), 3.01 (dd, *J*= 14.6, 8.2 Hz, A of AB, 1 H), 2.93 (dd, *J*= 17.0, 8.4 Hz, A′ of A′B′, 1 H), 2.91 (dd, *J*= 14.6, 4.0 Hz, B of AB, 1 H), 2.69 (dd, *J*= 17.2-16.8, 1.2-0.8 Hz, B′ of A′B′, 1 H), 2.36 (s, 3 H), 1.33 (t, *J*= 7.2 Hz, 3 H) , 1.27 (t, *J*= 7.2 Hz, 3 H); ^{13}C NMR (100 MHz, $CDCl_3$): δ 185.3, 166.1, 165.4, 160.4, 139.6, 135.1, 133.8, 133.5, 133.4, 129.2, 128.9, 126.1, 125.8, 117.3, 108.9, 68.3, 61.3, 40.5, 37.2, 34.6, 29.7, 21.0, 14.2, 14.1; HRMS (ESI): *m/z* calcd for $C_{24}H_{27}N_3O_6Na$ [M+Na]$^+$476.1798, found 476.1790.

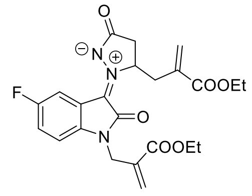

[Reaction time: 5 h]; **4g**: 146 mg, 31%, a yellow oil; IR (thin film): ν_{max} 3406, 2989, 2925, 1171, 1565, 1371, 1137, 754 cm^{-1}; ^{1}H NMR (400 MHz, $CDCl_3$): δ 8.23 (dd, *J*= 8.4, 2.4 Hz, 1 H), 7.09 (td, *J*= 8.4, 2.8 Hz, 1 H), 6.80 (dd, *J*= 8.4, 4.0 Hz, 1 H), 6.36 (s, 1 H), 6.35 (s, 1 H), 6.10-6.05 (m, 1 H), 5.75 (s, 1 H), 5.63 (s, 1 H), 4.66 (s, 2 H), 4.27 (q, *J*= 7.2 Hz, 2 H), 4.15 (qd, *J*= 7.2, 1.6 Hz, 2 H), 3.06 (dd, *J*= 14.0, 7.6 Hz, A of AB, 1 H), 2.94 (dd, *J*= 17.2, 8.4 Hz, A′ of A′B′, 1 H), 2.90 (dd, *J*= 14.0, 4.4 Hz, B of AB, 1 H), 2.70 (dd, *J*= 17.2, 1. Hz, B′ of A′B′, 1 H), 1.33 (t, *J*= 7.2 Hz, 3 H) , 1.27 (t, *J*= 7.2 Hz, 3 H); ^{13}C NMR (100 MHz, $CDCl_3$): δ 185.1, 166.1, 165.4, 160.4, 160.3, 137.6, 134.8, 133.7, 129.5, 126.6, 119.1, 118.9, 118.2 (d), 115.4 (d), 109.8 (d), 77.2, 68.7, 61.4, 40.7, 37.1, 34.3, 14.2, 14.1; ^{19}F NMR (376 MHz, $CDCl_3$): δ -118.7; HRMS (ESI): *m/z* calcd for $C_{23}H_{25}FN_3O_6$ [M+H]$^+$ 458.1727, found 458.1729.

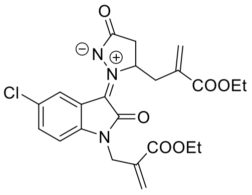

[Reaction time: 6 h]; **4h**: 256 mg, 54%, a yellow solid; m.p. 65.3-67.7 ^{o}C; IR (thin film): ν_{max} 3409, 2985, 2930, 1713, 1161, 1369, 1298, 1251, 1151, 697 cm^{-1}; ^{1}H NM R (400 MHz, $CDCl_3$): δ 8.48 (d, *J*= 2.0 Hz, 1 H), 7.35 (dd, *J*= 8.4, 2.0 Hz, 1 H), 6.81 (d, *J*= 8.4 Hz, 1 H), 6.36 (s, 1 H), 6.33 (s, 1 H), 6.28 (s, 1 H), 5.83 (ddd, *J*= 8.0, 4.8, 0.8 Hz, 1 H), 5.75 (s, 1 H), 5.64 (d, *J*= 8.0 Hz, 2 H), 4.69 (d, *J*= 17.6 Hz, A of AB, 1 H), 4.63 (d, *J*= 17.6 Hz, B of AB, 1 H), 4.28 (q, 7.2-6.8 Hz, 1 H), 4.19 (qd, *J*= 7.2, 1.2 Hz, 1 H), 4.15 (q, *J*= 7.2 Hz, 2 H), 3.05-2.97 (m, 2 H), 2.87 (td, *J*= 14.6, 4.6 Hz, 1 H), 2.48 (dd, *J*= 14.0, 9.6 Hz,

B′ of A′B′, 1 H), 1.33 (t, *J*= 7.2 Hz, 3H), 1.27 (t, *J*= 7.2 Hz, 3 H); ^{13}C NMR (100 MHz, DMSO-d_6): δ 187.1, 166.3, 165.9, 165.4, 160.0, 139.9, 136.7, 134.6, 132.2, 129.7, 128.6, 126.6, 124.2, 118.4, 110.1, 73.5, 61.3, 61.2, 40.6, 36.8, 33.9, 14.2, 14.1; HRMS (ESI): *m*/*z* calcd for $C_{23}H_{25}ClN_3O_6$ [M+H]$^+$ 474.1432, found 474.1441.

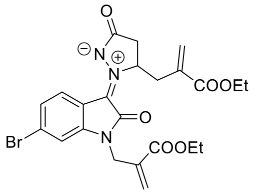

[Reaction time: 12 h]; **4i**: 352 mg, 68%, a yellow oil; IR (thin film): ν_{max}3420, 2982, 2933, 1721, 1603, 1370, 1300, 1062, 750 cm^{-1}; ^{1}H NMR (400 MHz, $CDCl_3$): δ 8.30 (d, *J*= 8.4 Hz, 1 H), 7.28 (dd, *J*= 8.0, 1.6 Hz, 1 H), 7.02 (d, *J*= 1.6 Hz, 1 H), 6.37 (s, 1 H), 6.35 (s, 1 H), 6.06-6.00 (m, 1 H), 5.74 (s, 1 H), 5.62 (s, 1 H), 4.65 (s, 2 H), 4.29 (q, *J*= 7.6 Hz, 2 H), 4.14 (qd, *J*= 7.2, 0.8 Hz, 2 H), 3.08 (dd, *J*= 14.0, 7.6 Hz, A of AB, 1 H), 2.95 (dd, *J*= 16.8, 8.4 Hz, A′ of A′B′, 1 H), 2.87 (dd, *J*= 14.2, 3.8 Hz, B of AB, 1 H), 2.69 (dd, *J*= 17.2, 1.6 Hz, B′ of A′B′ 1 H), 1.34 (t, *J*= 7.2 Hz, 3 H), 1.26 (t, *J*= 7.2 Hz, 3 H); ^{13}C NMR (100 MHz, $CDCl_3$): δ 185.1, 166.0, 165.2, 160.2, 142.4, 134.8, 133.4, 129.5, 129.1, 127.0, 126.6 (2C), 124.2, 116.2, 112.6, 77.2, 68.6, 61.4, 61.3, 40.7, 37.0, 34.3, 14.2 (d); HRMS (ESI): *m*/*z* calcd for $C_{23}H_{25}BrN_3O_6$ [M+H]$^+$ 518.0927, found 518.0936.

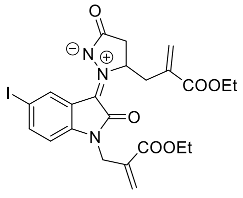

[Reaction time: 8 h]; **4j**: 311 mg, 55%, a yellow oil; IR (thin film): ν_{max} 3452, 3342, 1723, 1694, 1621, 1519, 1348, 1109, 977, 809, 749, 686 cm^{-1}; ^{1}H NMR (400 MHz, $CDCl_3$): δ 8.75 (d, *J*= 1.6 Hz, 1 H), 7.69 (dd, *J*= 8.4, 1.6 Hz, 1 H), 6.65 (d, *J*= 8.4 Hz, 1 H), 6.35 (s, 2 H), 6.10-6.05 (m, 1 H), 5.74 (s, 1 H), 5.62 (s, 1 H), 4.65 (s, 2 H), 4.27 (q, *J*= 7.2 Hz, 1 H), 4.19-4.12 (m, 2 H), 3.05 (dd, *J*= 14.0, 7.6 Hz, A of AB, 1 H), 2.95 (dd, *J*= 17.2, 8.4 Hz, A′ of A′B′, 1 H), 2.88 (dd, *J*= 14.2, 4.2 Hz, B of AB, 1 H), 2.70 (dd, *J*= 16.8, 1.6 Hz, 1 H), 1.33 (t, *J*= 7.2 Hz, 3 H), 1.27 (t, *J*= 7.2 Hz, 3 H); ^{13}C NMR(400 MHz, $CDCl_3$): δ 185.2, 166.0, 165.3, 159.8, 141.0, 140.9, 135.8, 134.7, 133.5, 129.5, 126.6, 123.6, 119.1, 111.1, 86.1, 77.2, 68.8, 61.4, 40.6, 37.1, 34.3, 14.2 (d); HRMS (ESI): *m*/*z* calcd for $C_{23}H_{25}IN_3O_6$ [M+H]$^+$ 566.0788, found 566.0744.

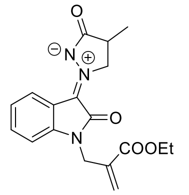

[Reaction time: 2 min]; **10**: 143 mg, 84%, a yellow solid, m.p. 157.2-158.7 °C; IR (thin film): ν_{max} 3684, 3987, 1710, 1694, 1573, 1383, 1267, 1128, 761 cm^{-1}; ^{1}H NMR (400 MHz, $CDCl_3$): δ 8.46 (d, *J*= 7.6 Hz, 1 H), 7.39 (m, 1 H), 7.16 (t, *J*= 7.6 Hz, 1 H), 6.87 (d, *J*= 8.0 Hz, 1 H), 6.35 (s, 1 H), 5.60 (s, 1 H), 5.27 (dd, *J*= 16.0, 7.2 Hz, 1 H), 4.63 (s, 2 H), 4.57 (dd, *J*= 15.6, 5.6 Hz, 1 H), 4.27 (q, *J*= 7.2 Hz, 2 H), 3.04-2.95 (m, 1 H), 1.49 (d, *J*= 7.2 Hz, 3 H), 1.33 (t, *J*= 7.2 Hz, 3 H); ^{13}C NMR (100 MHz, $CDCl_3$): δ 189.7, 165.4, 160.9, 141.5, 133.8, 132.6, 127.9, 126.5, 124.4, 123.6, 116.9, 109.2, 77.2, 64.2, 61.4, 40.6, 34.4, 16.1, 14.2; HRMS (ESI): *m*/*z* calcd for $C_{18}H_{20}N_3O_4$ [M+H]$^+$ 342.1454, found 1455.

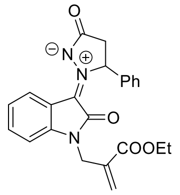

[Reaction time: 30 min]; **12**: 154 mg, 77%, a yellow solid, m.p. 175.3-177.2 °C; IR (thin film): ν_{max} 3384, 2935, 2934, 1604, 1182, 1162, 661, 648 cm^{-1}; ^{1}H NMR (400 MHz, $CDCl_3$): δ 8.60 (d, *J*= 7.6 Hz, 1 H), 7.38-7.29 (m, 6 H), 7.13 (ψt, *J*= 8.0 Hz, 1 H), 6.92-6.89 (m, 1 H), 6.52 (d, *J*= 1.2 Hz, 1 H), 5.93 (d, *J*= 1.6 Hz, 1 H), 5.36 (q, *J*= 7.2 Hz, 1 H), 3.89-3.74 (m, 2 H), 3.3.1 (dd, *J*= 17.2, 9.6 Hz, 1 H), 2.71 (dd, *J*= 16.8, 1.6 Hz, 1 H), 1.58 (t, *J*= 7.2 Hz, 1 H), 0.83 (t, *J*= 7.2 Hz, 3 H); ^{13}C NMR (100 MHz, $CDCl_3$): δ 185.4, 165.3, 159.7, 141.8, 138.7, 133.7, 133.0, 129.1, 128.8, 126.1, 125.7, 125.1, 123.5, 117.3, 109.0, 72.2, 61.3, 40.4, 38.9, 14.1, HRMS (ESI): *m*/*z* calcd for $C_{23}H_{21}N_3O_4Na$ [M+Na]$^+$ 426.1430, found 426.1423.

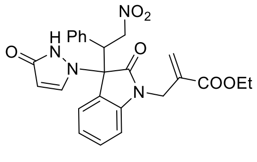

[Reaction time: 2 h]; **13**: 89 mg, 37%, a light yellow solid, m.p. 179.6-183.5 °C; IR (thin film): ν_{max} 2854, 1715, 1663, 1495, 1377, 1303, 1142, 755 cm^{-1}; ^{1}H NMR (400 MHz, DMSO-d_6): δ 10.16 (s, 1 H), 7.70 (d, *J*= 7.2 Hz, 1 H), 7.11 (d, *J*= 2.0 Hz, 1 H), 7.17-7.04 (m, 7 H), 6.71 (d, *J*= 8.0 Hz, 1 H), 6.01 (s, 1 H), 5.63 (d, *J*= 2.4 Hz, 1 H), 5.47 (dd, *J*= 13.2, 11.6 Hz, 1 H), 5.05 (dd, *J*= 11.2, 3.6 Hz, 1 H),

4.91 (s, 1 H), 4.86 (dd, *J*=13.6, 3.6 Hz,1 H), 4.45 (s, 2 H), 4.19-4.11 (m, 2 H), 1.21 (t, *J*= 3.2 Hz, 3 H); ^{13}C NMR (100 MHz, DMSO-d_6): δ 171.4, 165.2, 162.9, 141.3, 133.5, 133.2, 131.3, 130.2, 129.6, 128.7, 128.5, 127.5, 126.4, 125.1, 123.4, 110.2, 93.35, 79.6, 74.9, 70.1, 61.3, 48.2, 41.0, 14.4; HRMS (ESI): *m/z* calcd for $C_{25}H_{24}N_4O_6Na$ [M+Na]$^+$ 499.1594, found 499.1583.

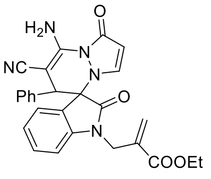

[Reaction time: 20 min]; **14**: 145 mg, 60%, a white solid m.p. 206.0-207.3 ^{o}C; IR (thin film): ν_{max} 3370, 2981, 2189, 1725, 1683, 1492, 1382, 1172, 1027, 754 cm^{-1} ; ^{1}H NMR (400 MHz, DMSO-d_6): δ 7.85 (d, *J*= 7.2 Hz, 1 H), 7.72 (s, 2 H), 7.40 (m, 1 H), 7.32 (d, *J*= 4.0 Hz, 1 H), 7.28 (ψt, *J*= 7.4 Hz 1 H), 7.20-7.30 (m, 3 H), 6.91 (*br* s, 2 H), 6.77 (d, *J*= 7.8 Hz, 1 H), 5.76 (s, 1 H), 5.67 (d, *J*= 4.0 Hz, 1 H), 4.77 (s, 1 H), 4.20 (d, *J*= 16.8 Hz, B of AB, 1 H), 4.14-4.04 (m, 2 H), 1.18 (t, *J*= 7.2 Hz, 3 H); ^{13}C NMR (100 MHz, DMSO-d_6): δ 169.3, 165.4, 165.0, 150.2, 143.3, 142.8, 134.4, 133.3, 132.0, 130.2, 128.6, 128.4, 126.0, 125.2, 124.1, 122.0, 119.5, 110.6, 101.4, 67.1, 61.2, 58.5, 46.4, 14.4; HRMS (ESI): *m/z* calcd for $C_{27}H_{24}N_5O_4$ [M+H]$^+$482.1828, found 482.1689.

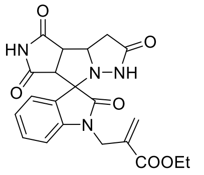

[Reaction time: 2 h]; **15**: 192 mg, 91%, a white solid, m.p. 245.7-247.9 ^{o}C; IR (thin film): ν_{max} 2982, 2924, 1726, 1702, 1611, 1376, 1152, 1024, 767 cm^{-1}; ^{1}H NMR (400 MHz, DMSO-d_6): δ 11.37 (s, 1 H), 9.10 (s, 1 H), 7.35 (t, *J*= 8.0 Hz, 1 H), 7.19 (d, *J*= 7.6 Hz, 1 H), 7.06 (t, *J*= 7.6 Hz, 1 H), 6.99 (d, *J*= 8.0 Hz, 1 H), 6.17 (s, 1 H), 5.50 (s, 1 H), 4.73 (td, *J*= 9.2, 4.8 Hz, 1 H), 4.52 (s, 2 H), 4.21 (q, *J*= 7.2 Hz, 2 H), 3.86 (t, *J*= 9.4 Hz, 1 H), 3.66 (d, *J*= 8.8 Hz, 1 H), 2.86 (dd, *J*= 17.2, 4.8 Hz, 1 H, A of AB), 2.63 (dd, *J*= 17.2, 9.2 Hz, 1 H, B of AB), 1.26 (t, *J*= 7.2 Hz, 3 H); ^{13}C NMR (100 MHz, DMSO-d_6): δ 178.2, 176.4, 175.1, 174.5, 165.4, 143.5, 134.2, 130.6, 128.7, 125.5, 122.6, 122.4, 109.8, 74.9, 63.1, 61.3, 53.6, 49.2, 40.5, 33.9, 14.5; HRMS (ESI): *m/z* calcd for $C_{21}H_{21}N_4O_6$ [M+H]$^+$ 425.1461, found 425.1451.

3. Copes of NMR for all new compounds.

^{1}H and ^{13}C NMR Spectra for Compound **3a**

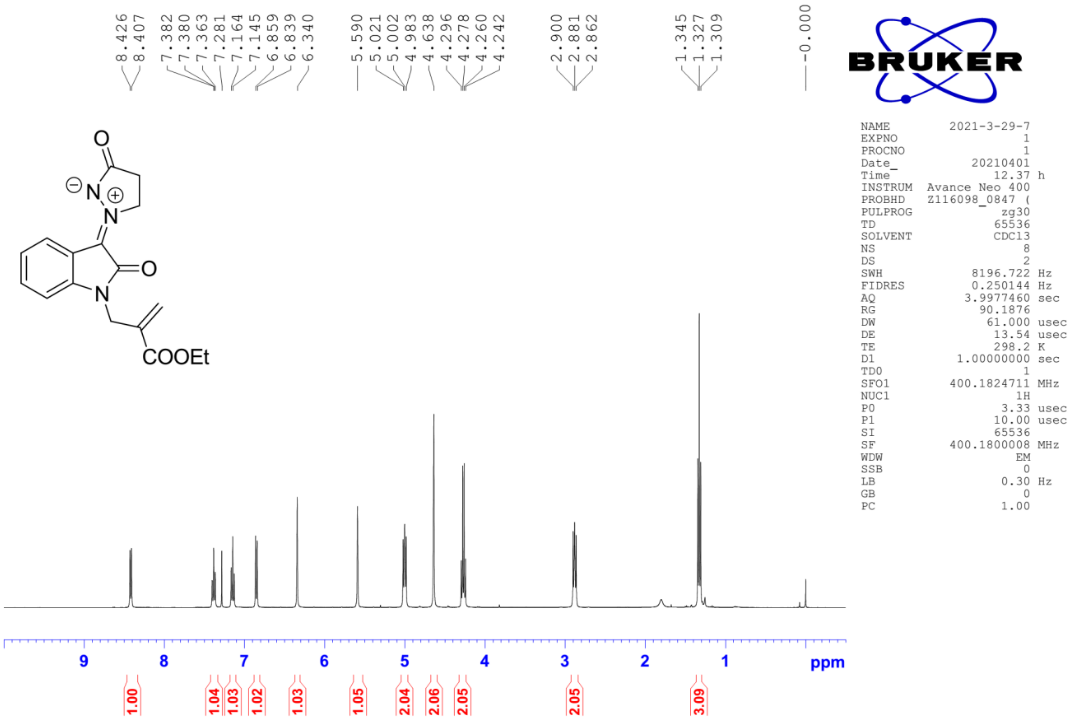

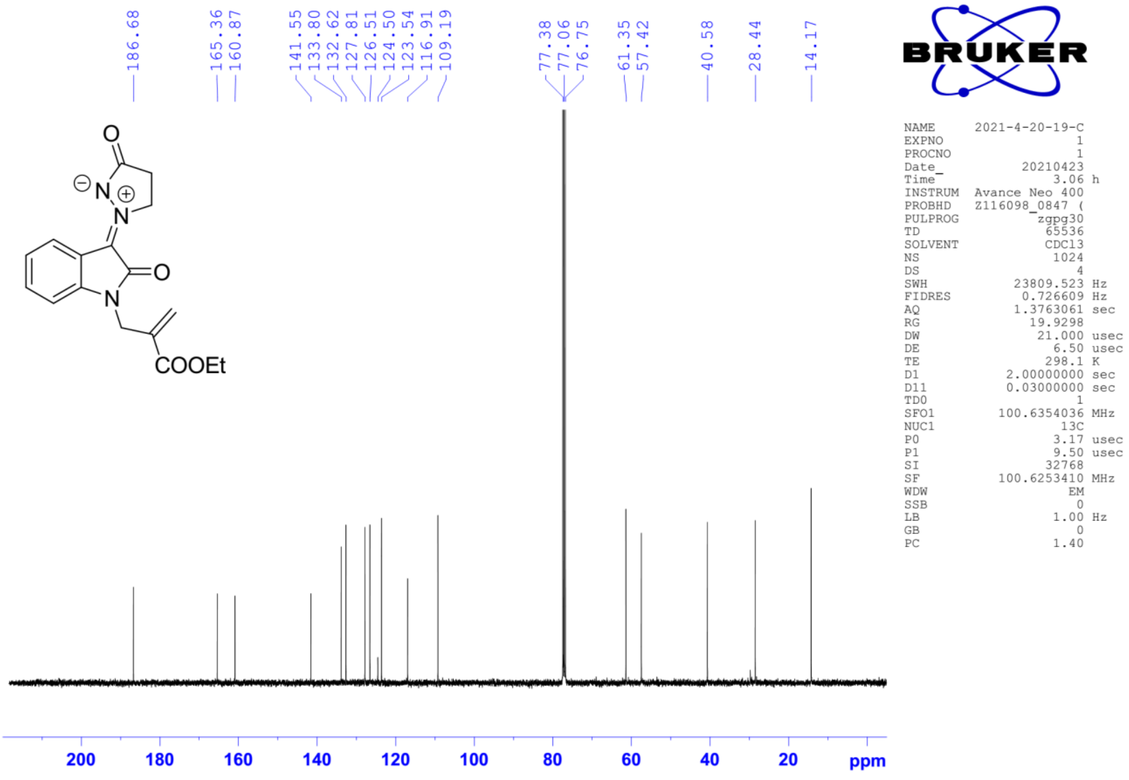

^{1}H and ^{13}C NMR Spectra for Compound **3b**

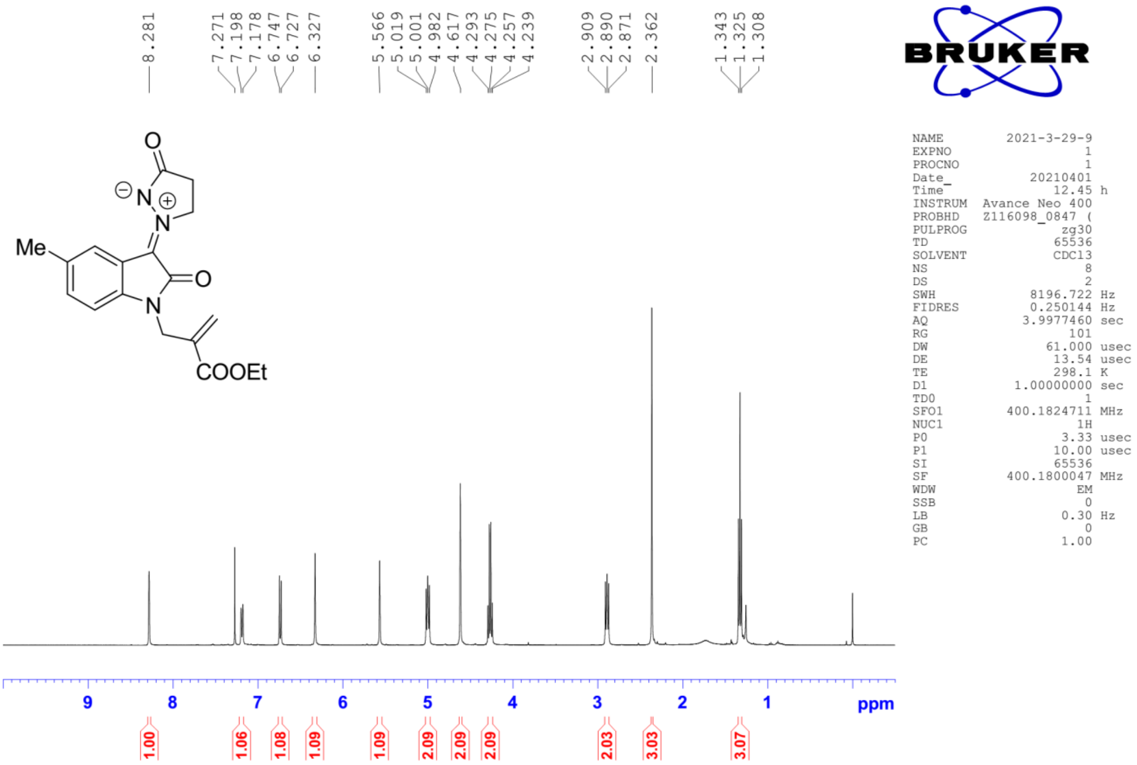

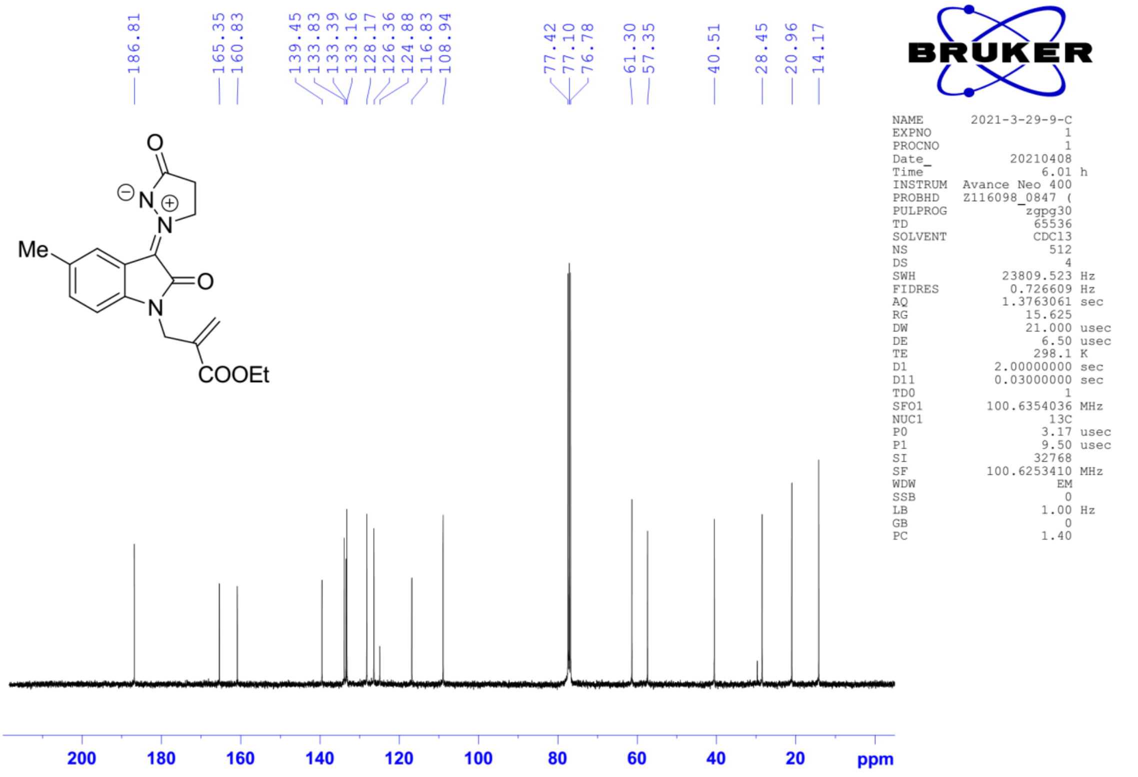

^{1}H and ^{13}C NMR Spectra for Compound **3c**

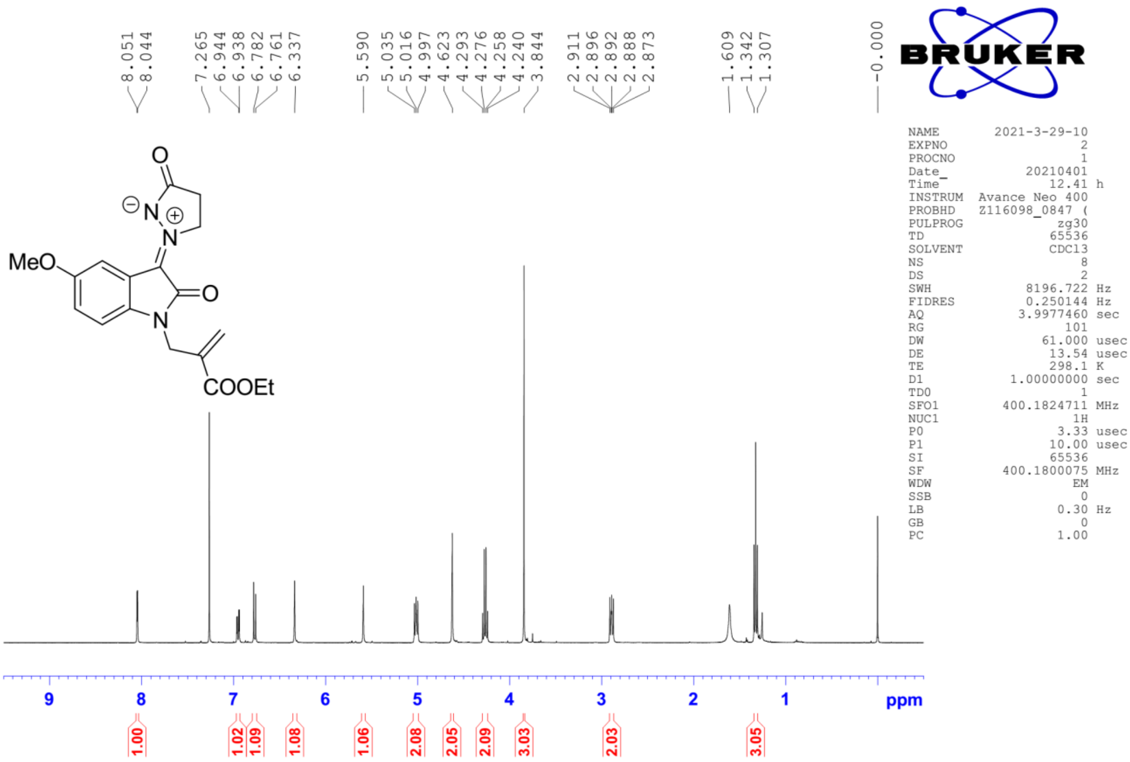

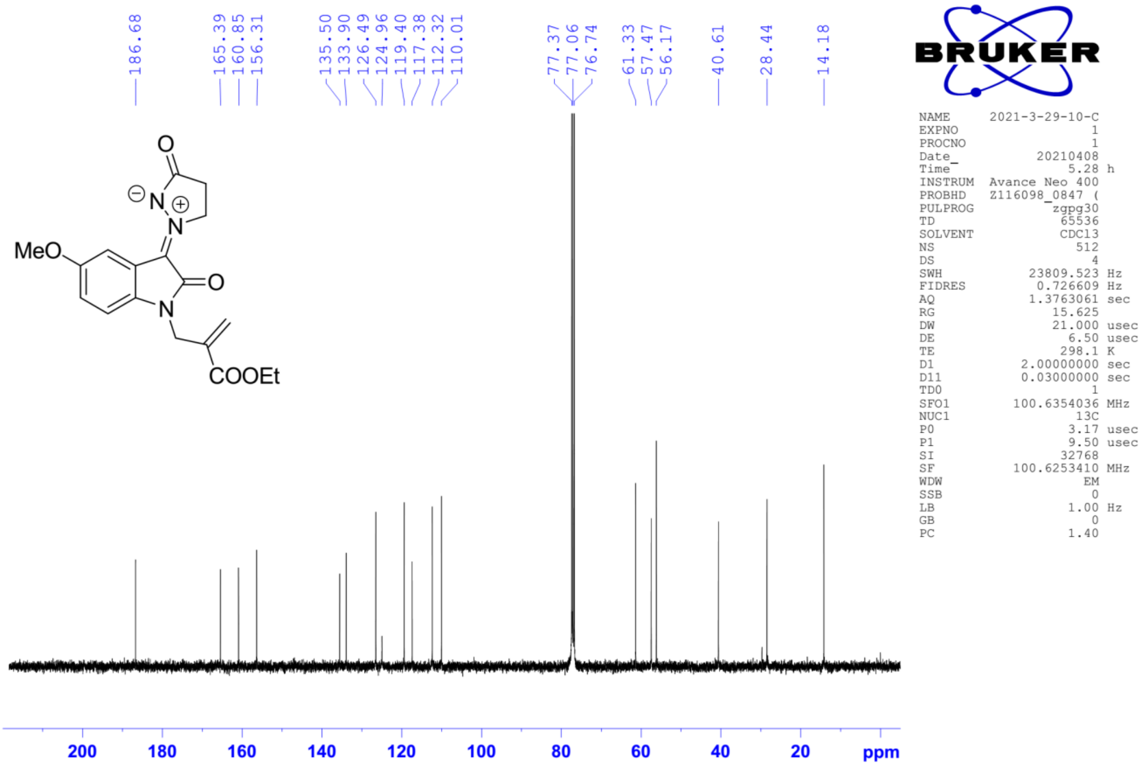

^{1}H, ^{13}C and ^{19}F NMR Spectra for Compound **3d**

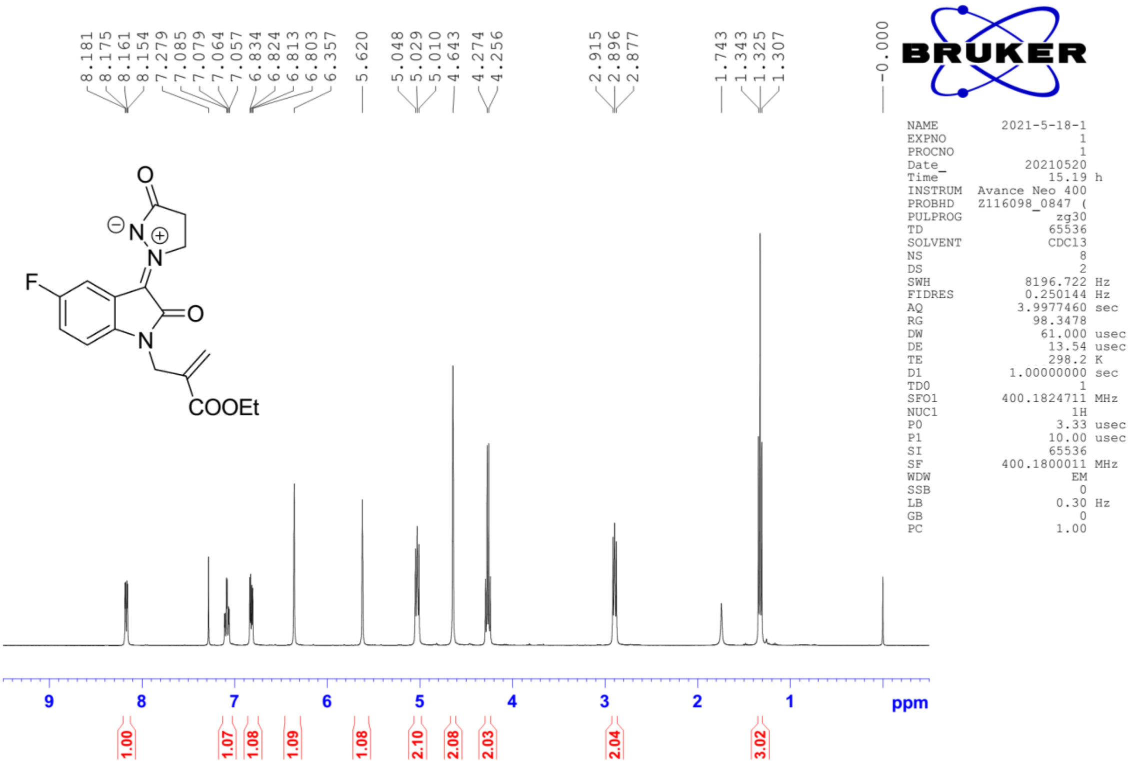

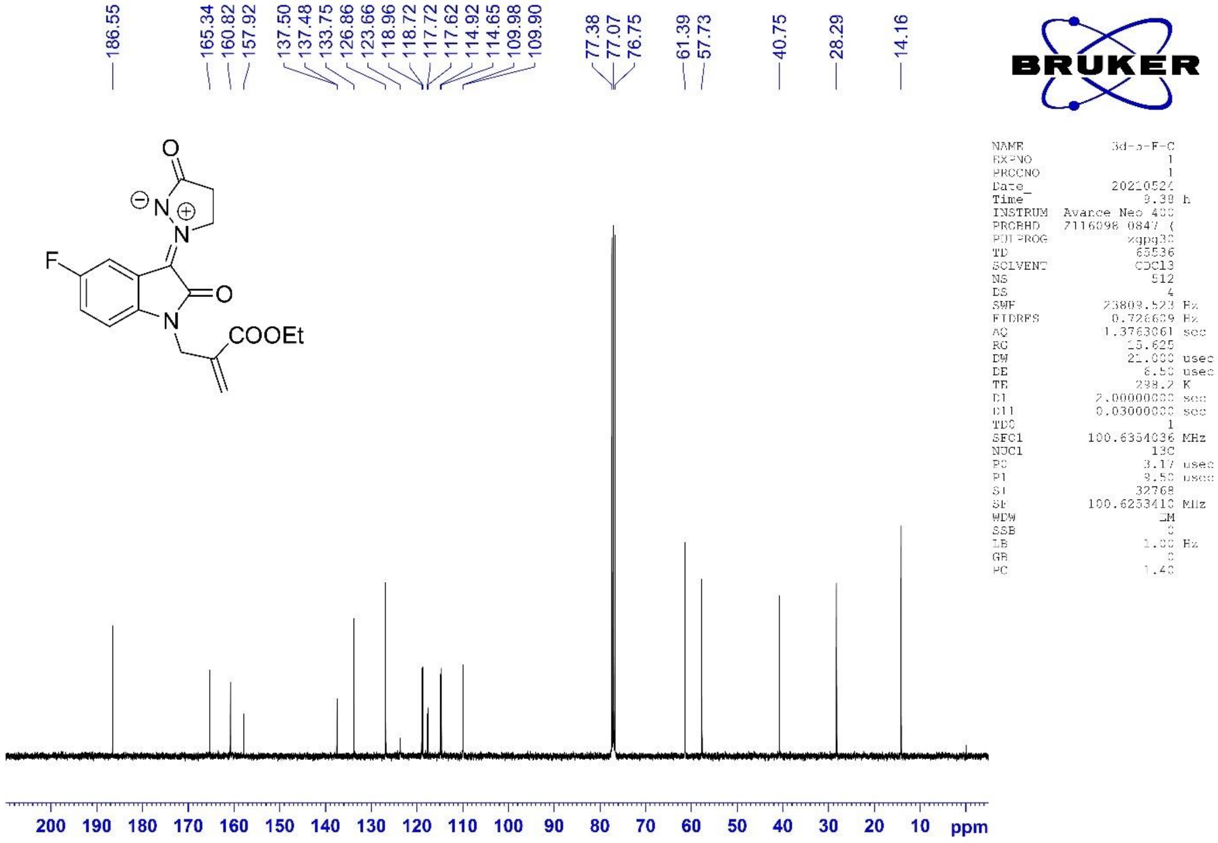

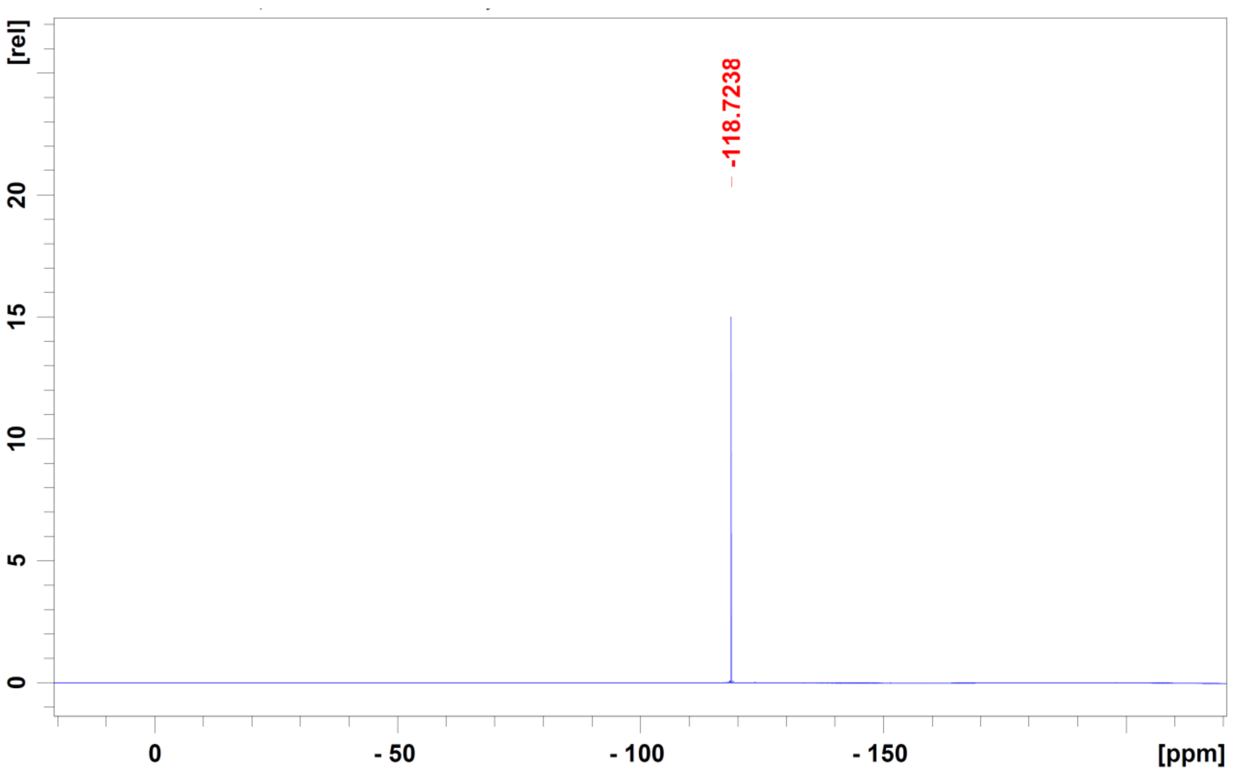

^{1}H and ^{13}C NMR Spectra for Compound **3e**

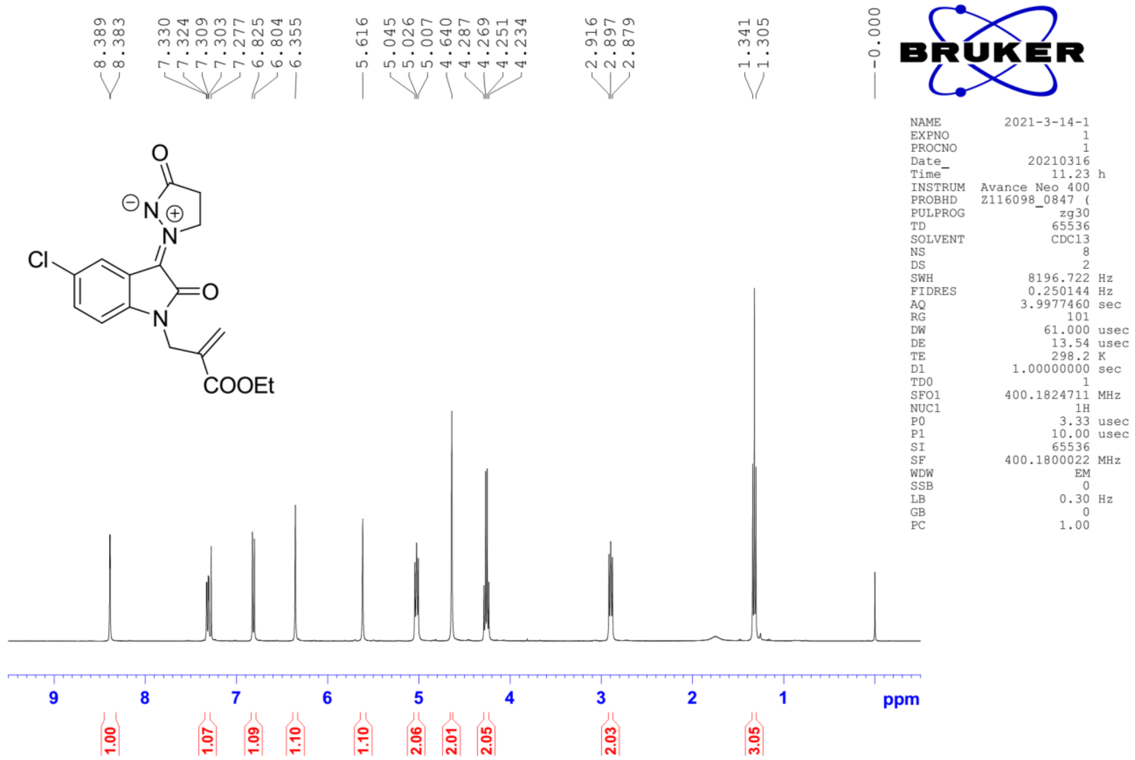

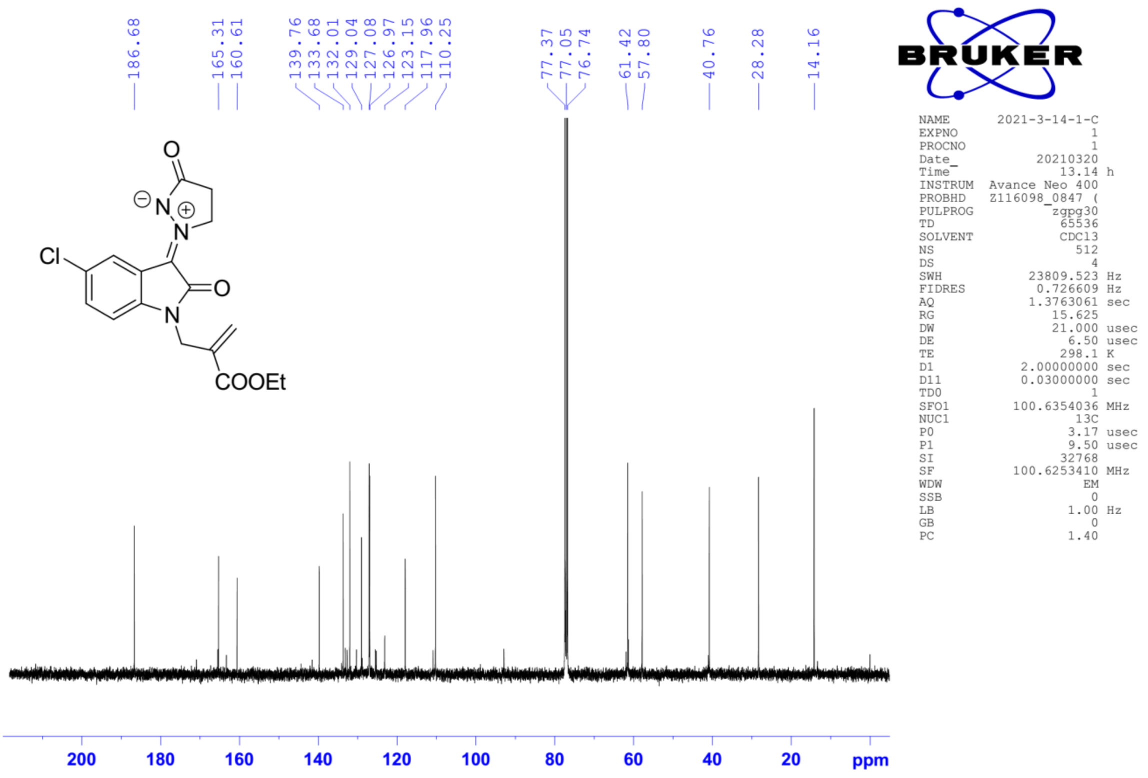

^1H and ^{13}C NMR Spectra for Compound **3f**

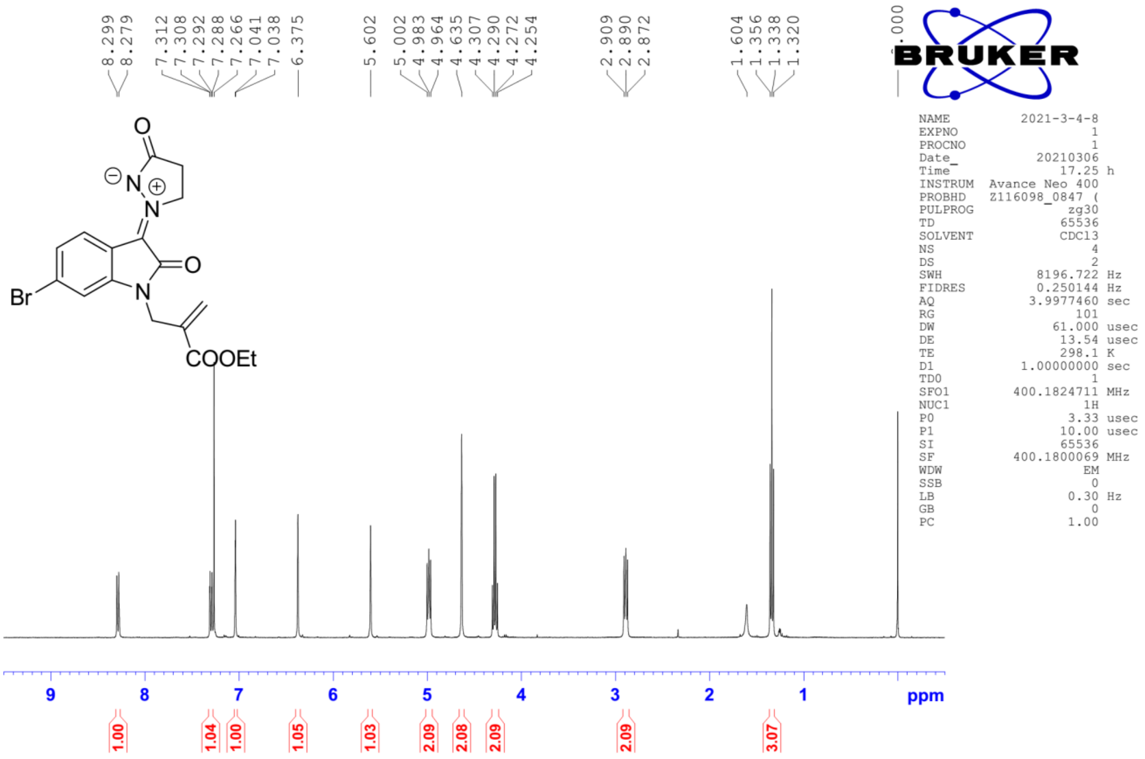

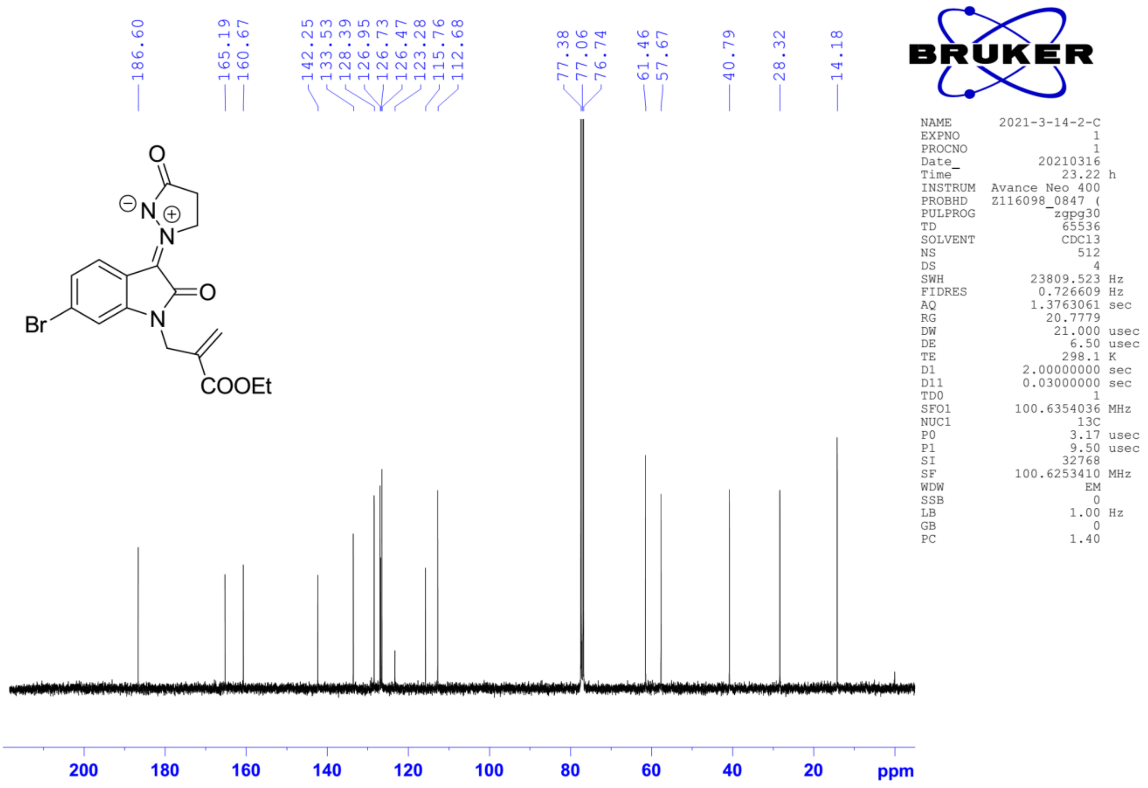

^{1}H and ^{13}C NMR Spectra for Compound **3g**

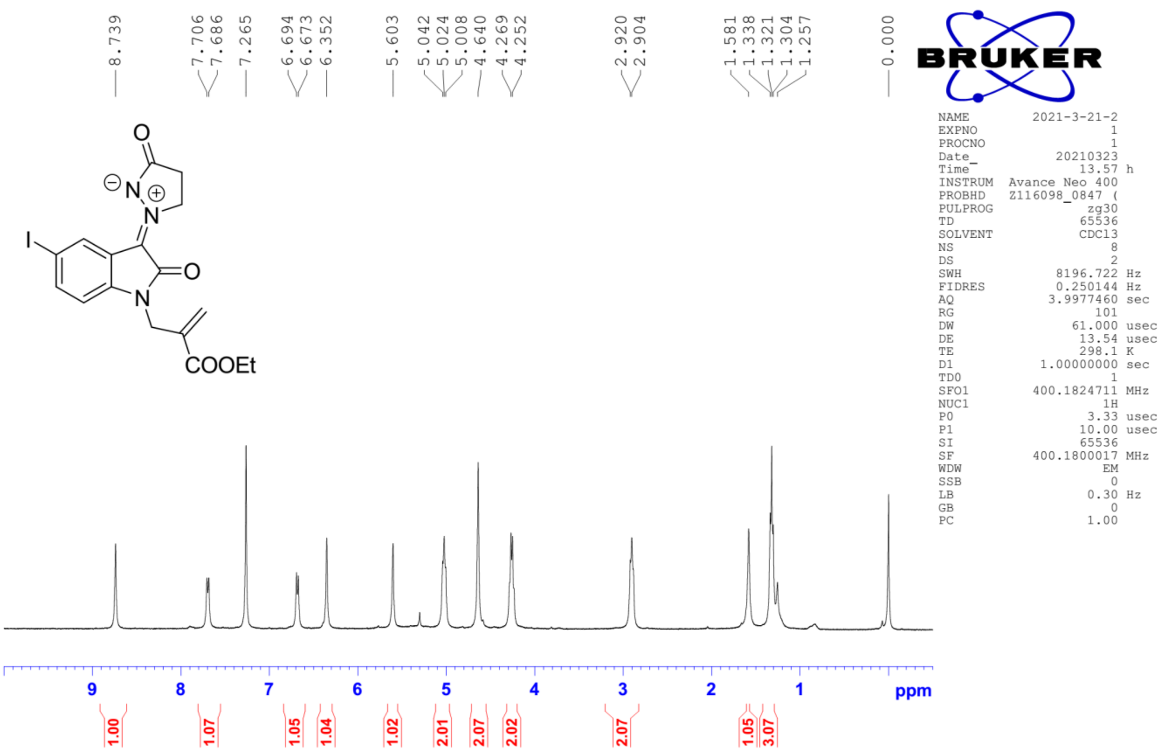

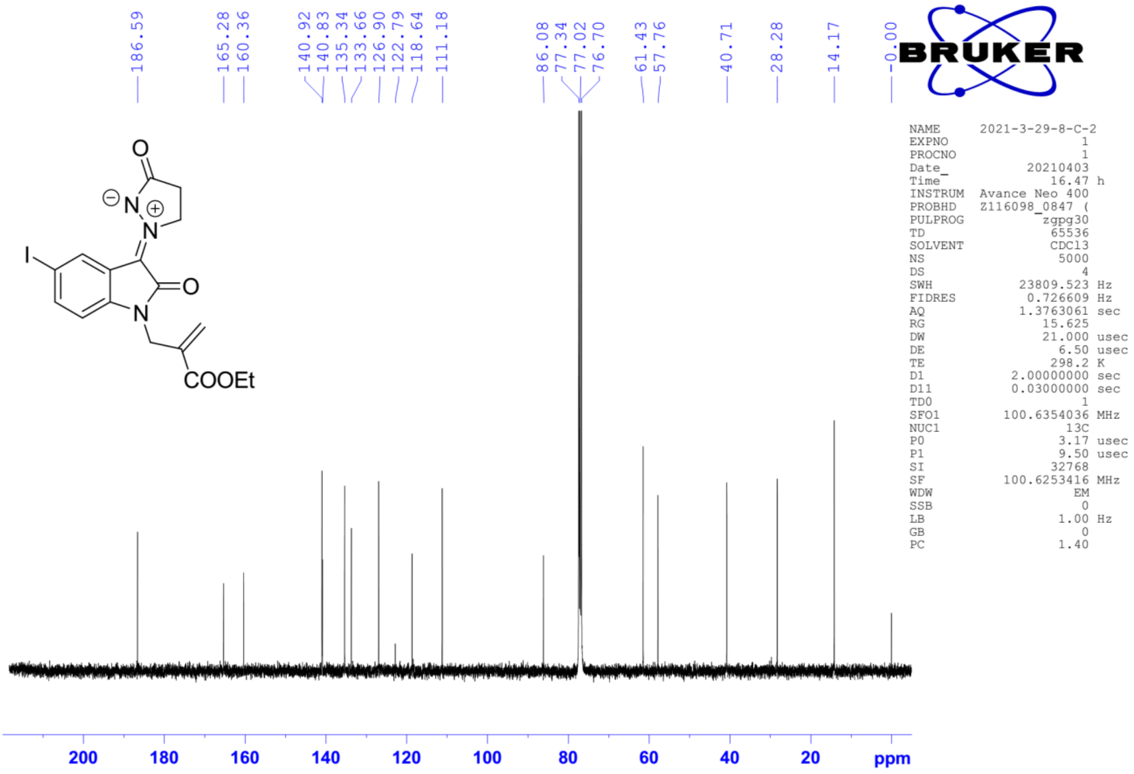

^{1}H, ^{13}C and ^{19}F NMR Spectra for Compound **3h**

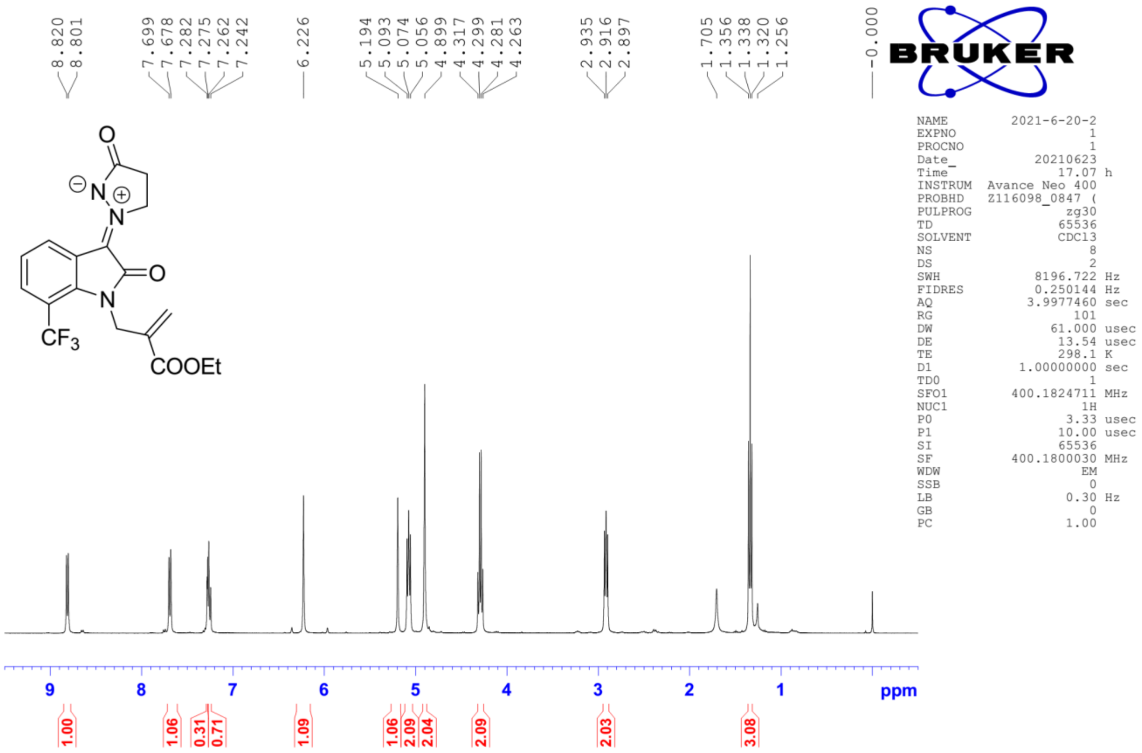

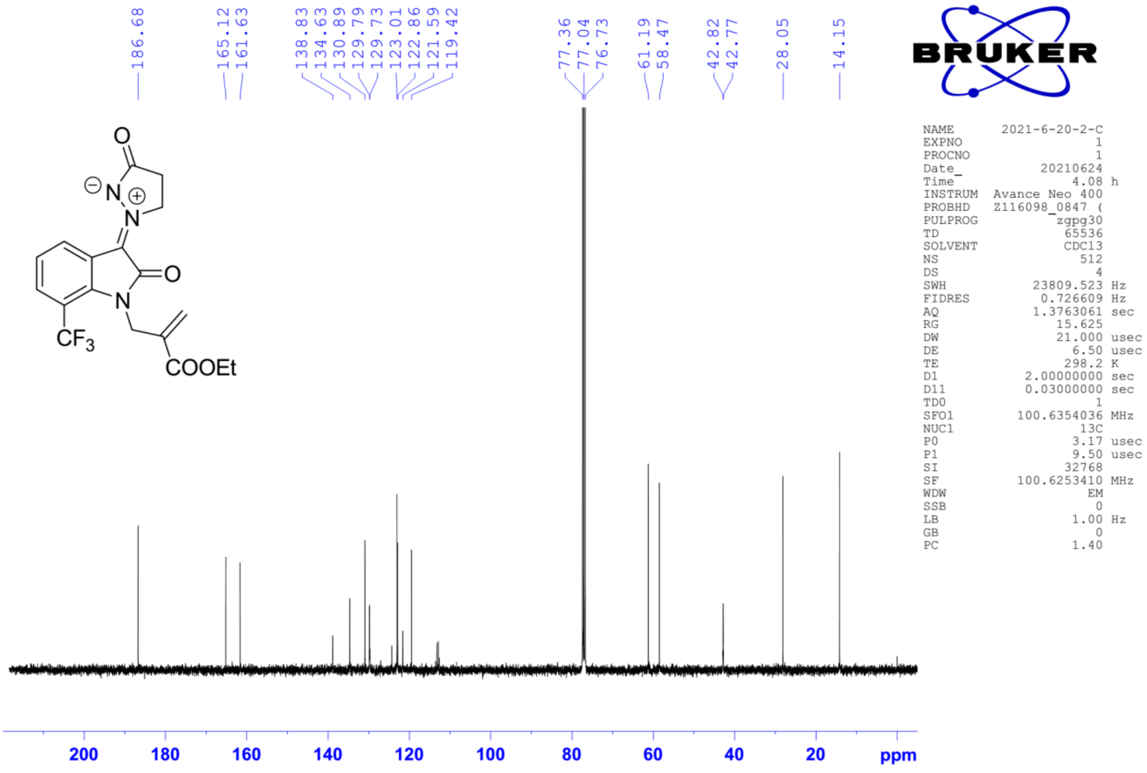

186.68
165.12
161.63
138.83
134.63
130.89
129.79
129.73
123.01
122.86
121.59
119.42
77.36
77.04
76.73
61.19
58.47
42.82
42.77
28.05
14.15
BRUKER
NAME 2021-6-20-2-C
EXPNO 1
PROCNO 1
Date_ 20210624
Time 4.08 h
INSTRUM Avance Neo 400
PROBHD Z116098_0847 (
PULPROG zgpg30
TD 65536
SOLVENT CDCl3
NS 512
DS 4
SWH 23809.523 Hz
FIDRES 0.726609 Hz
AQ 1.3763061 sec
RG 15.625
DW 21.000 usec
DE 6.50 usec
TE 298.2 K
D1 2.00000000 sec
D11 0.03000000 sec
TD0 1
SFO1 100.6354036 MHz
NUC1 13C
P0 3.17 usec
P1 9.50 usec
SI 32768
SF 100.6253410 MHz
WDW EM
SSB 0
LB 1.00 Hz
GB 0
PC 1.40
200
180
160
140
120
100
80
60
40
20
ppm

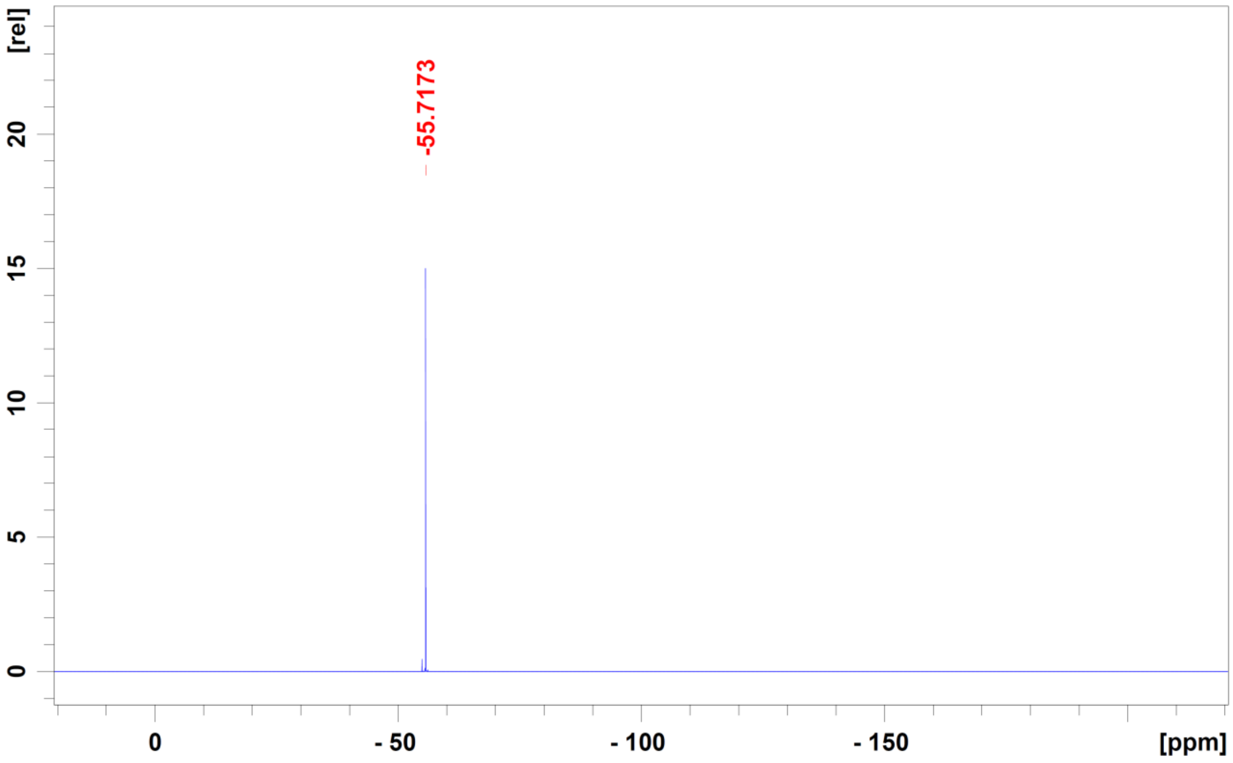

-55.7173
[rel]
20
15
10
5
0
0
- 50
- 100
- 150
[ppm]

^{1}H and ^{13}C NMR Spectra for Compound **3'i**

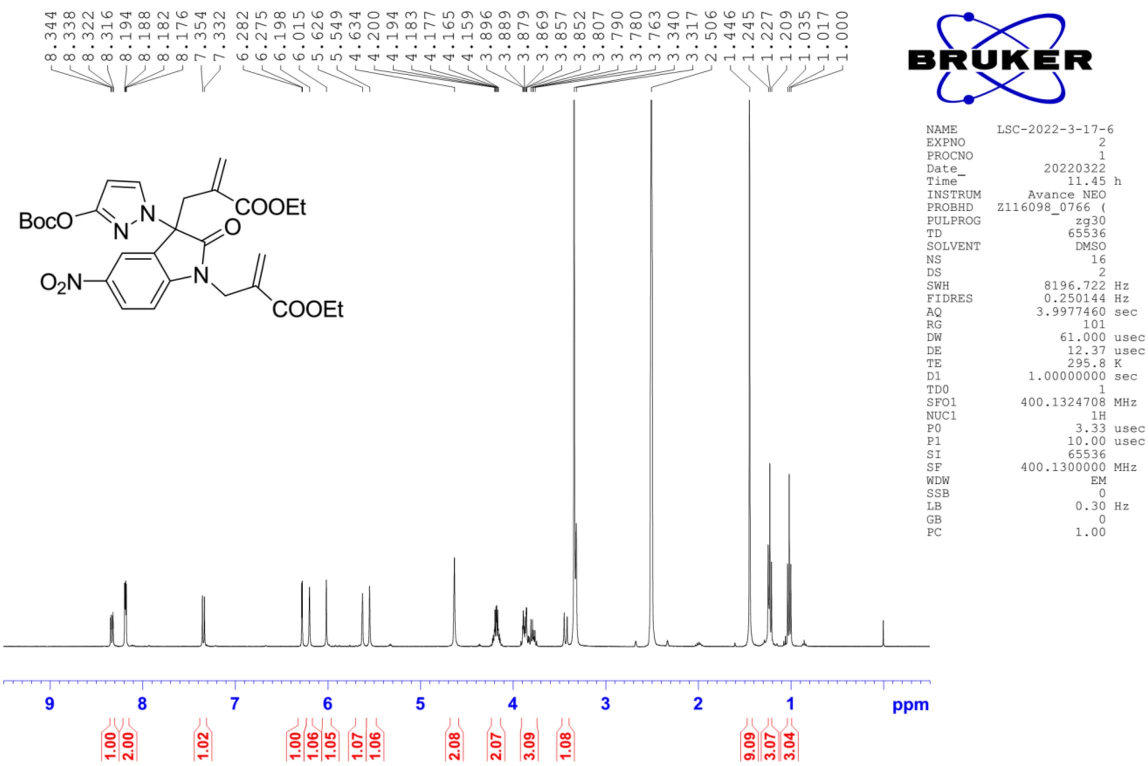

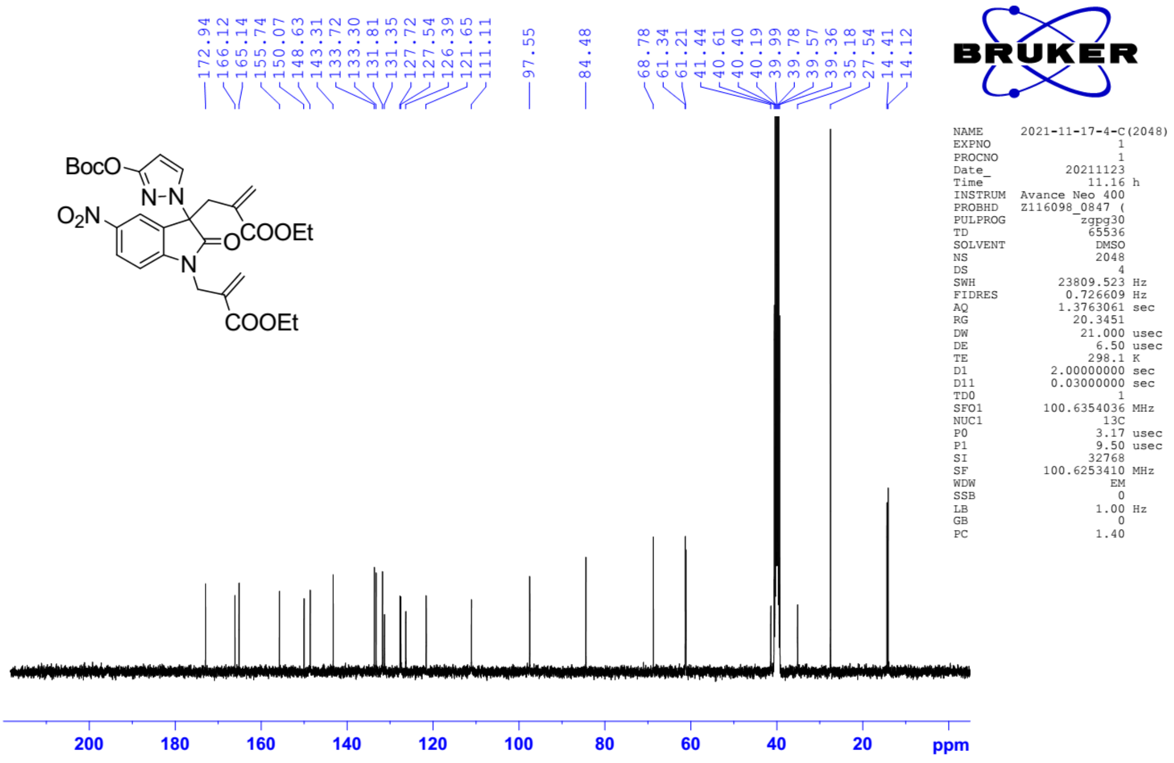

^{1}H and ^{13}C NMR Spectra for Compound **3j**

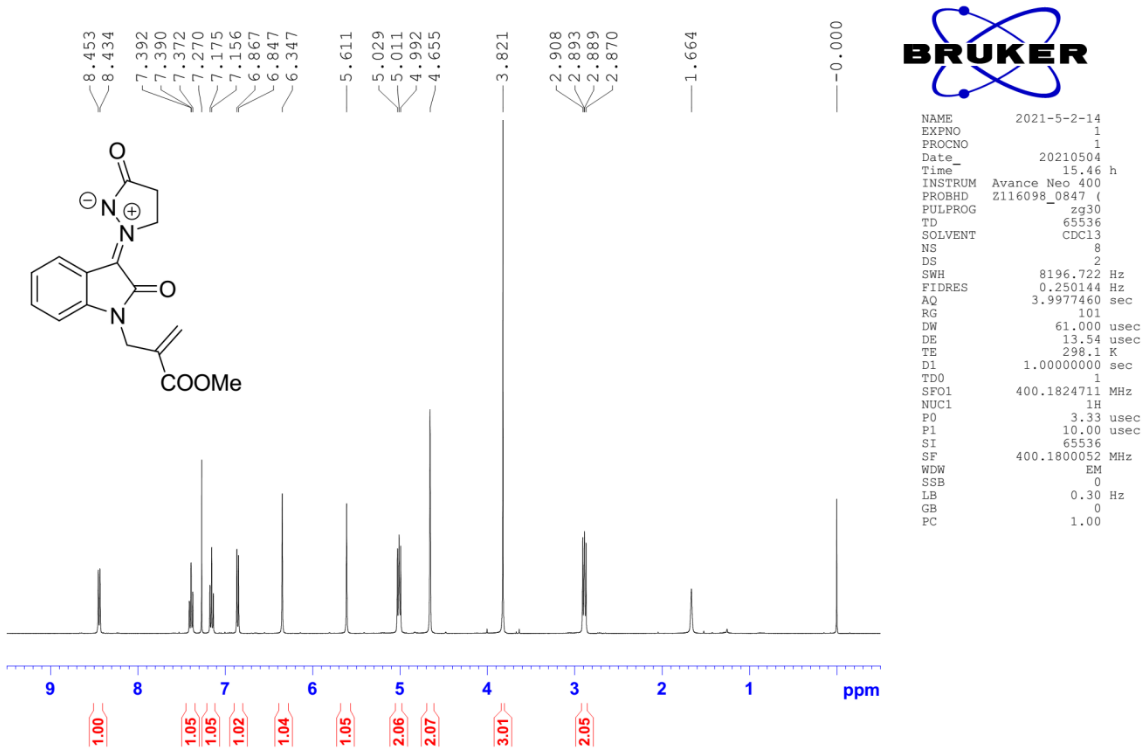

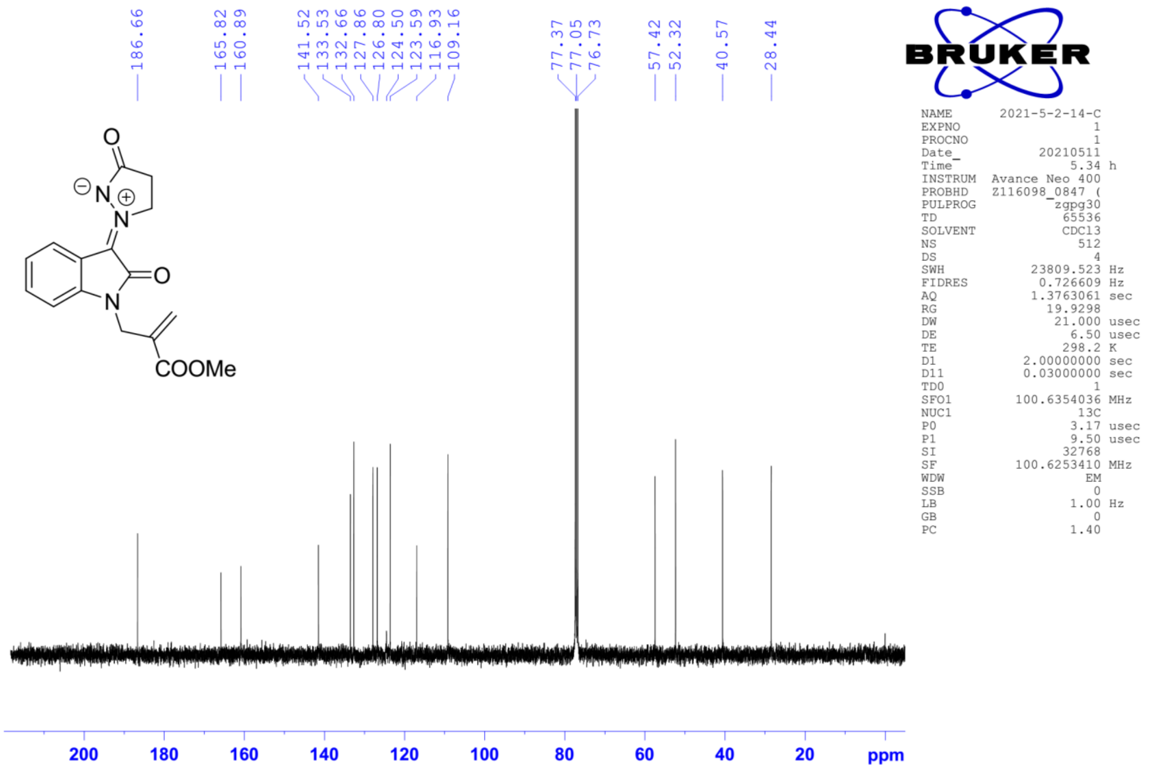

^{1}H and ^{13}C NMR Spectra for Compound **3k**

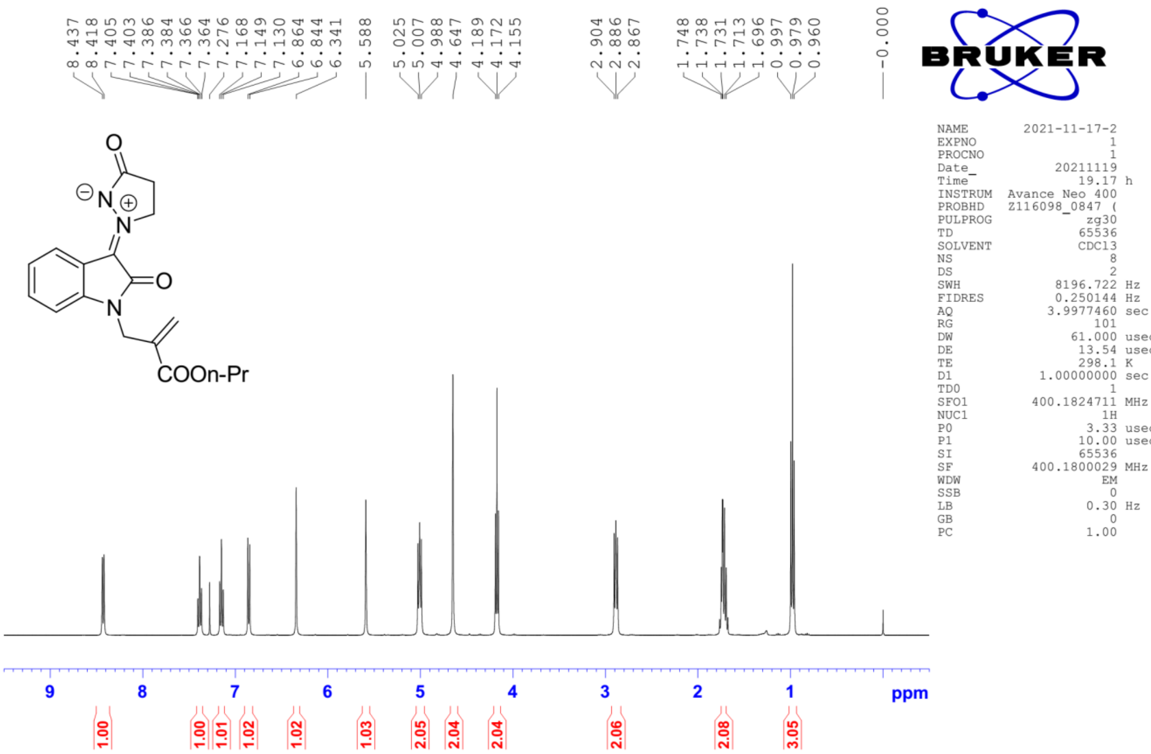

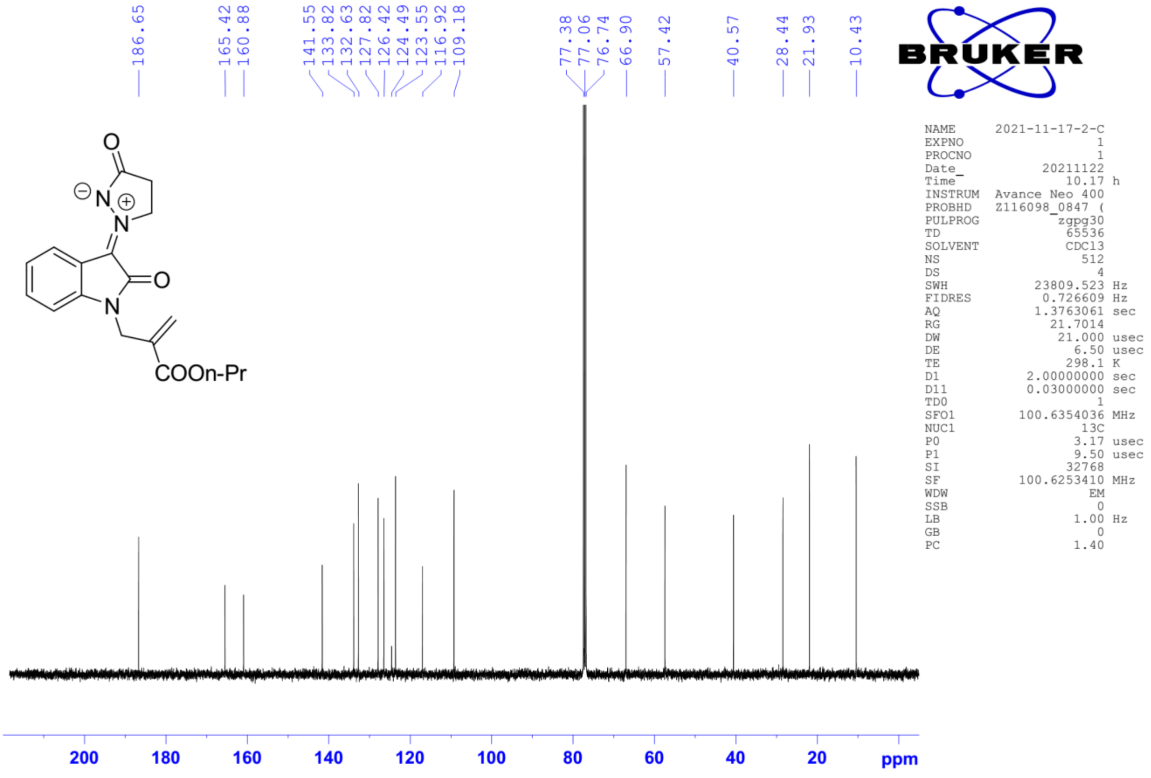

^{1}H and ^{13}C NMR Spectra for Compound **31**

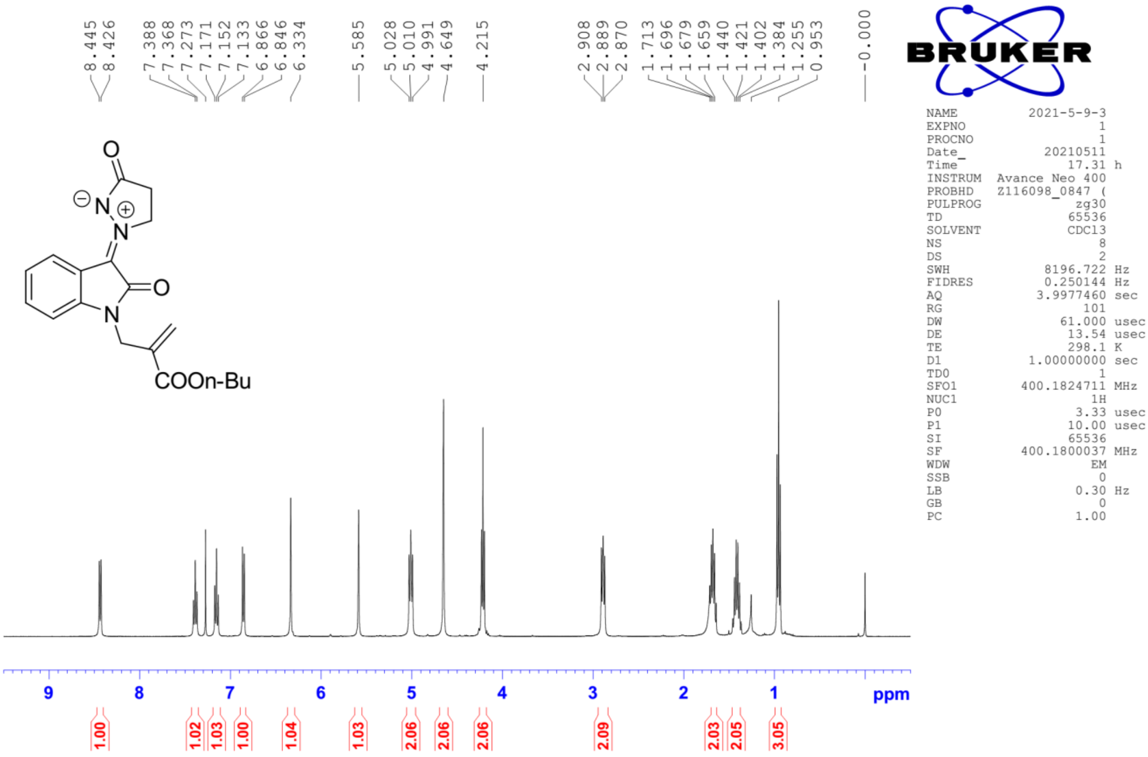

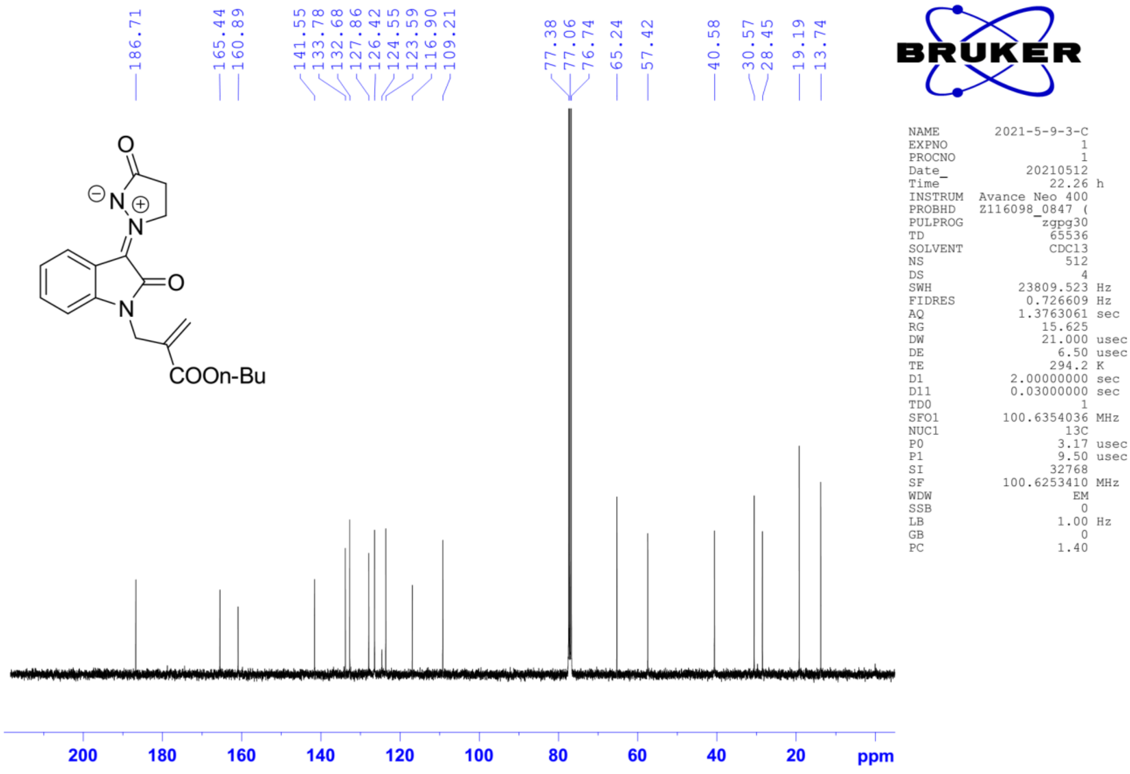

^{1}H and ^{13}C NMR Spectra for Compound **3m**

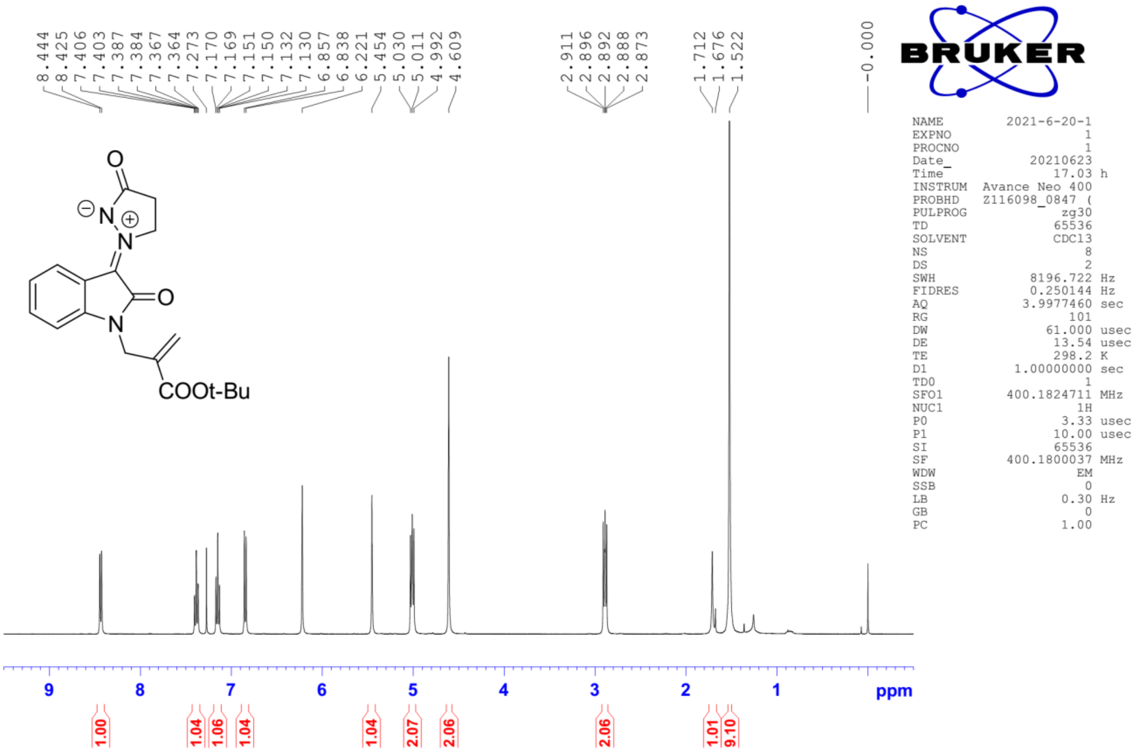

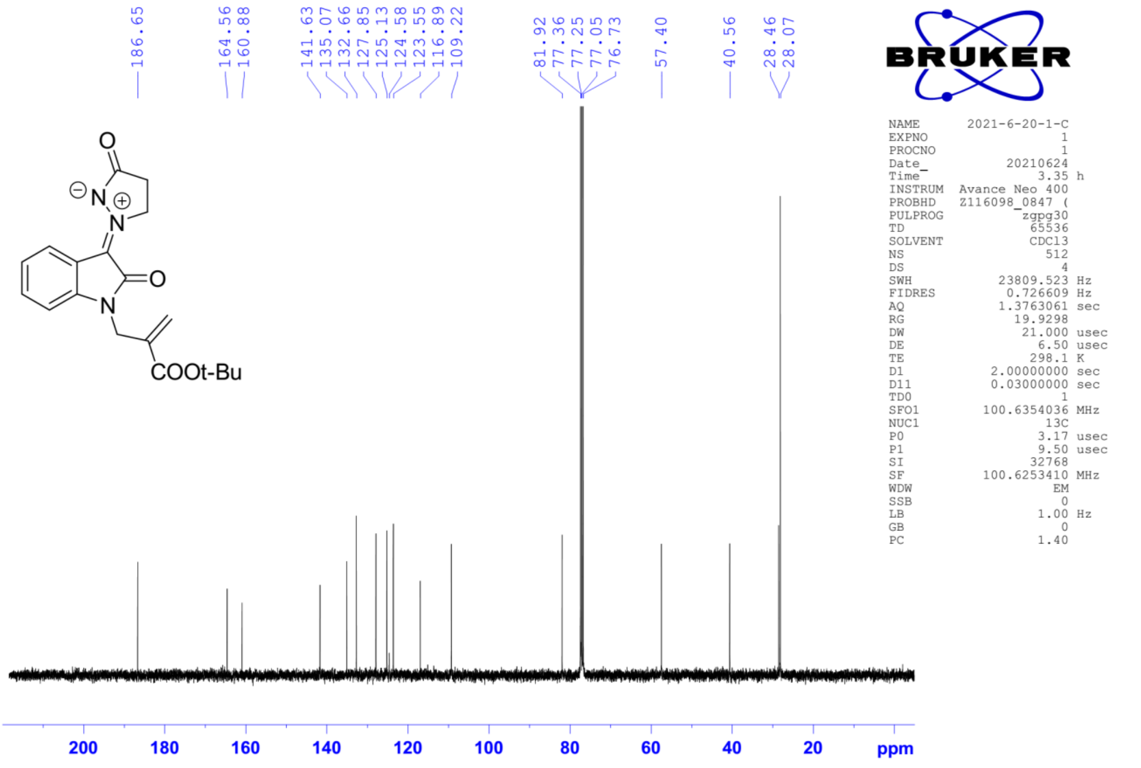

^{1}H and ^{13}C NMR Spectra for Compound **6a**

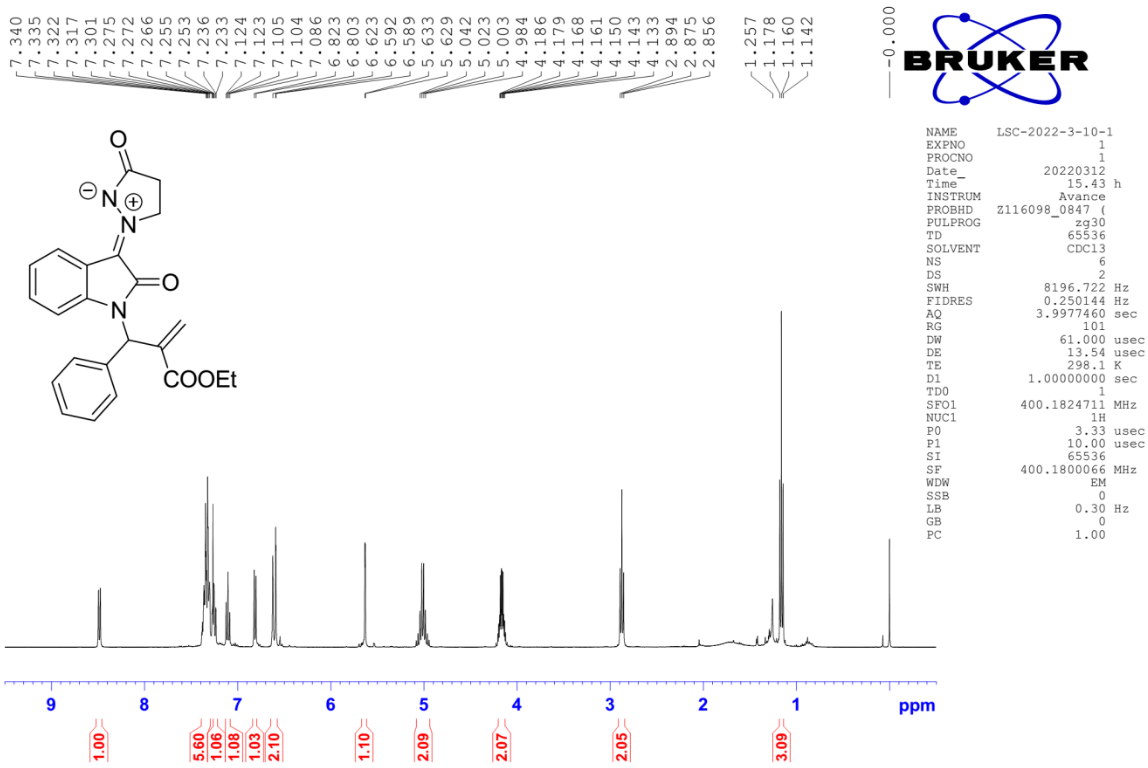

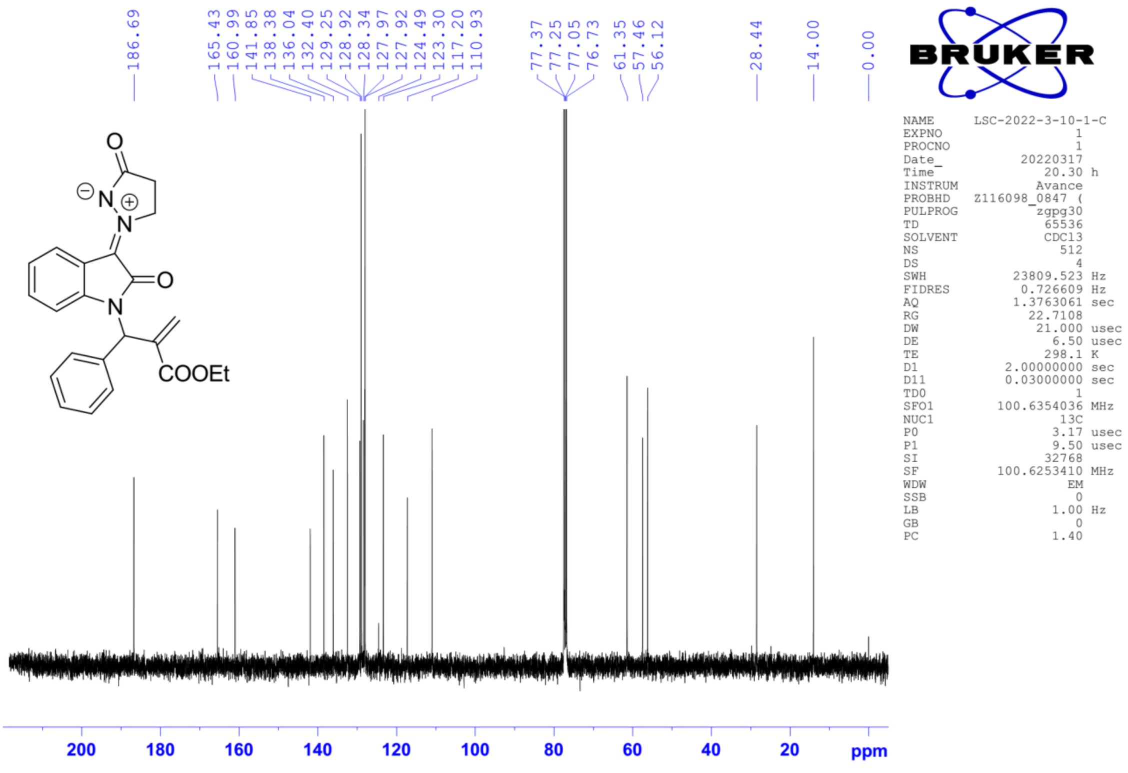

^{1}H NMR Spectra for Compound **6b** (mixed with an inseparable by-product)

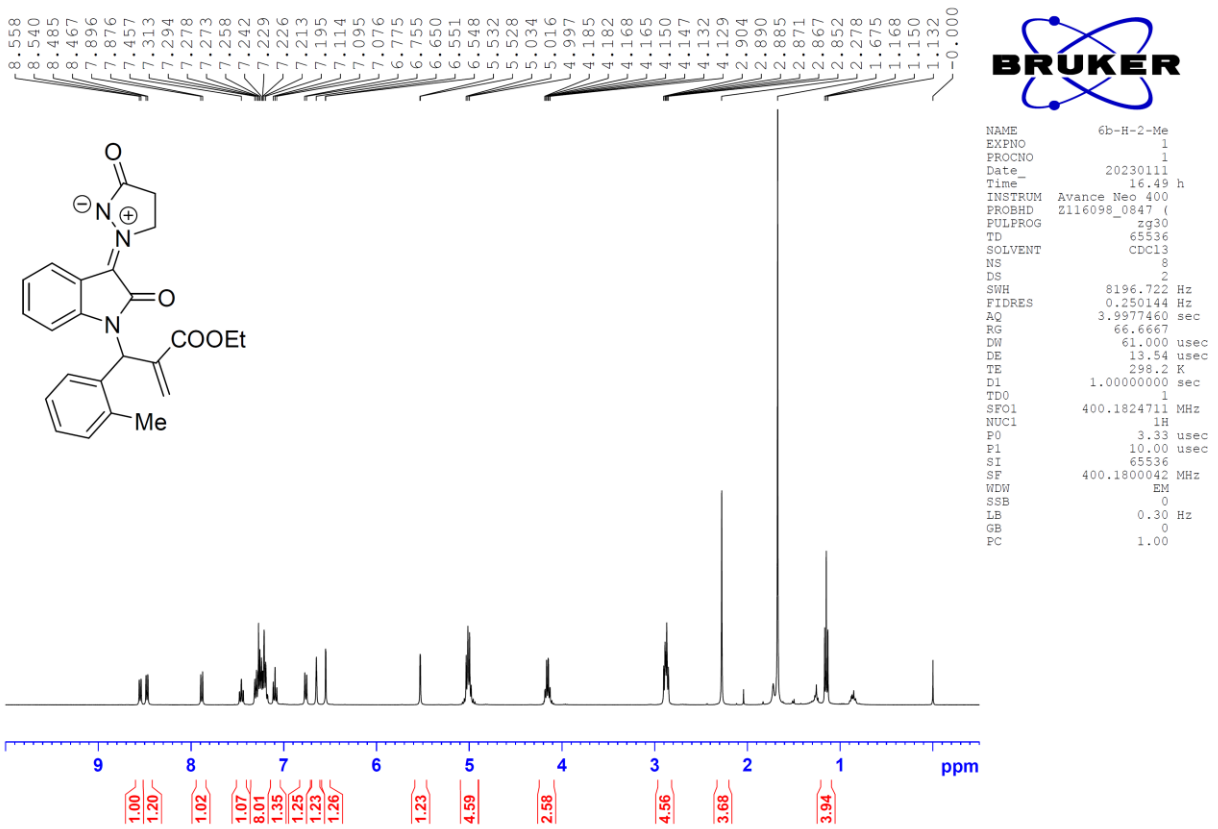

^{1}H and ^{13}C NMR Spectra for Compound **6c**

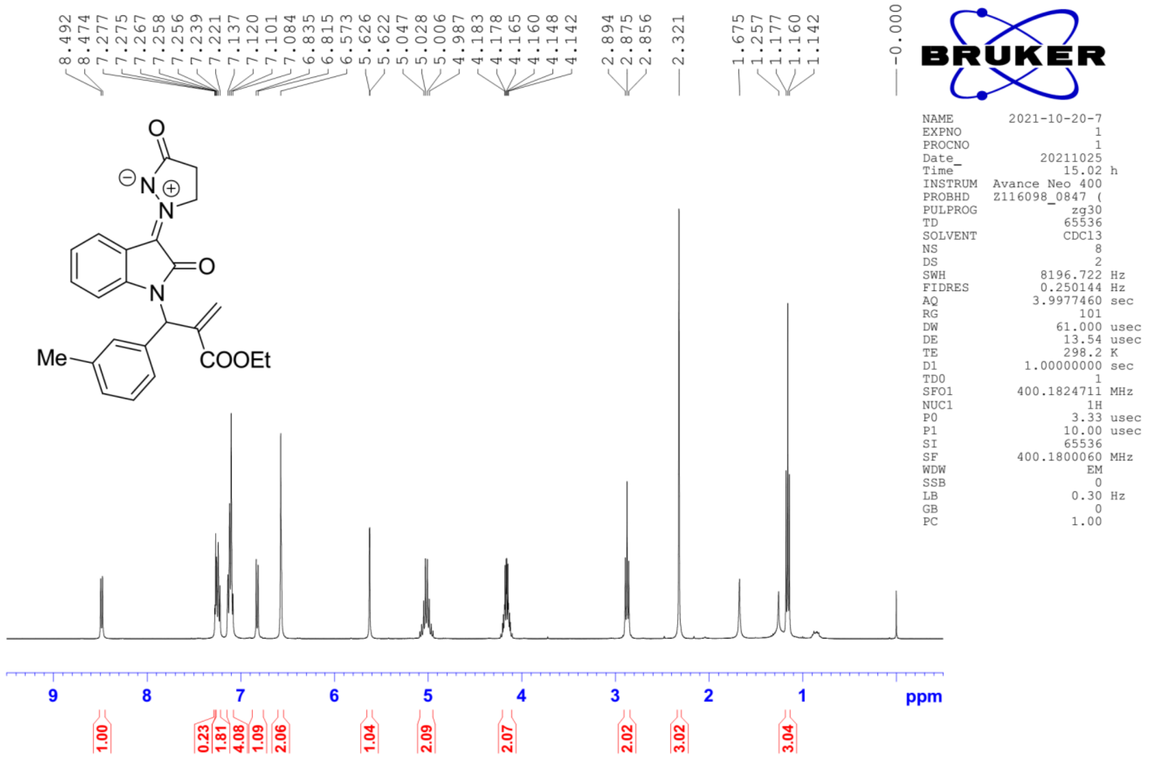

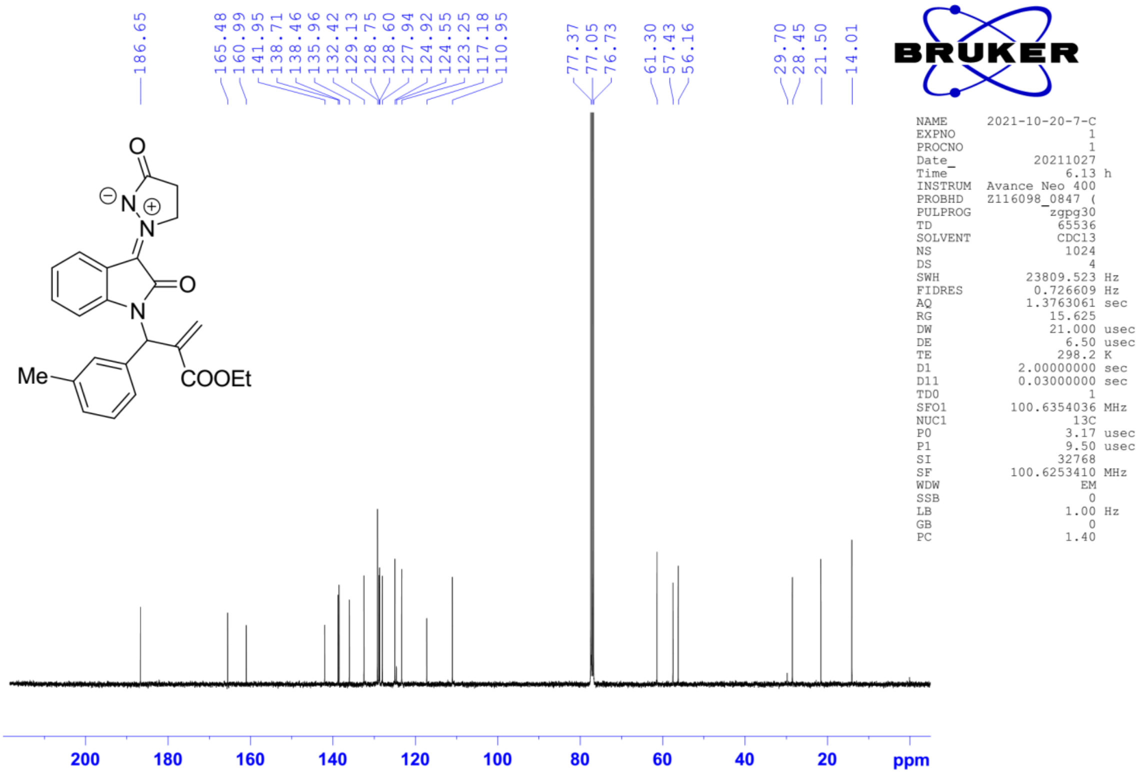

^{1}H and ^{13}C NMR Spectra for Compound **6d**

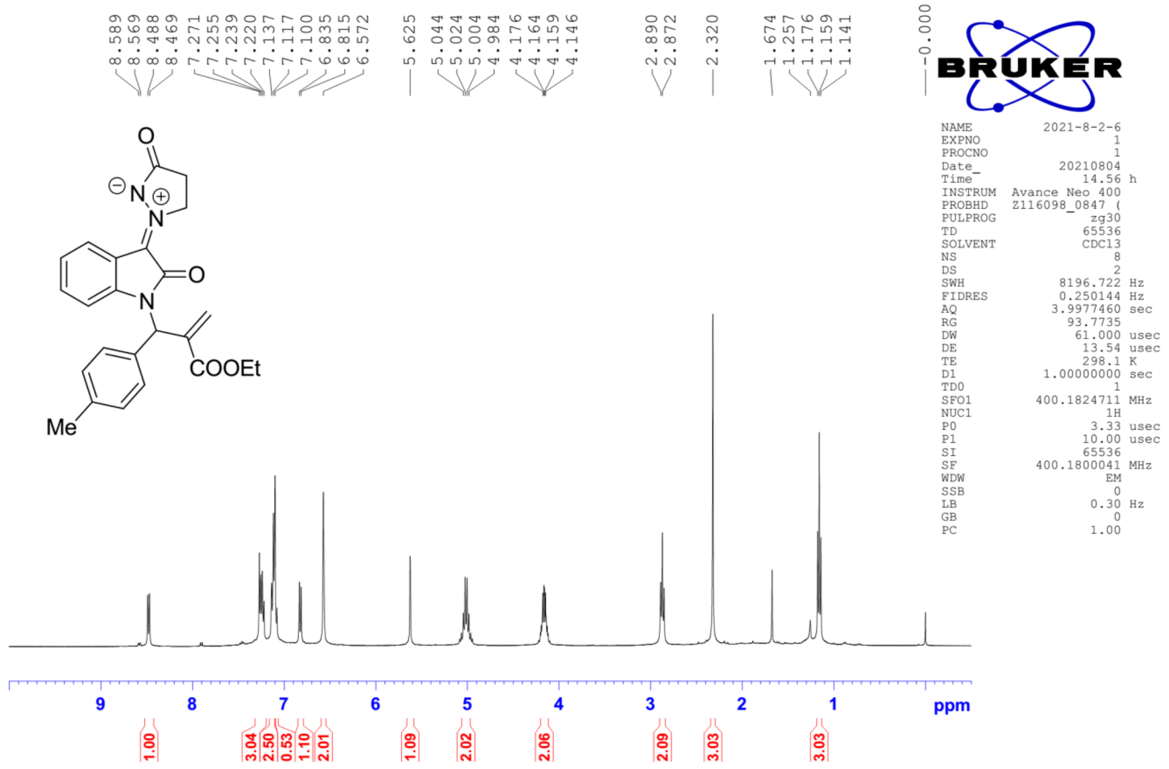

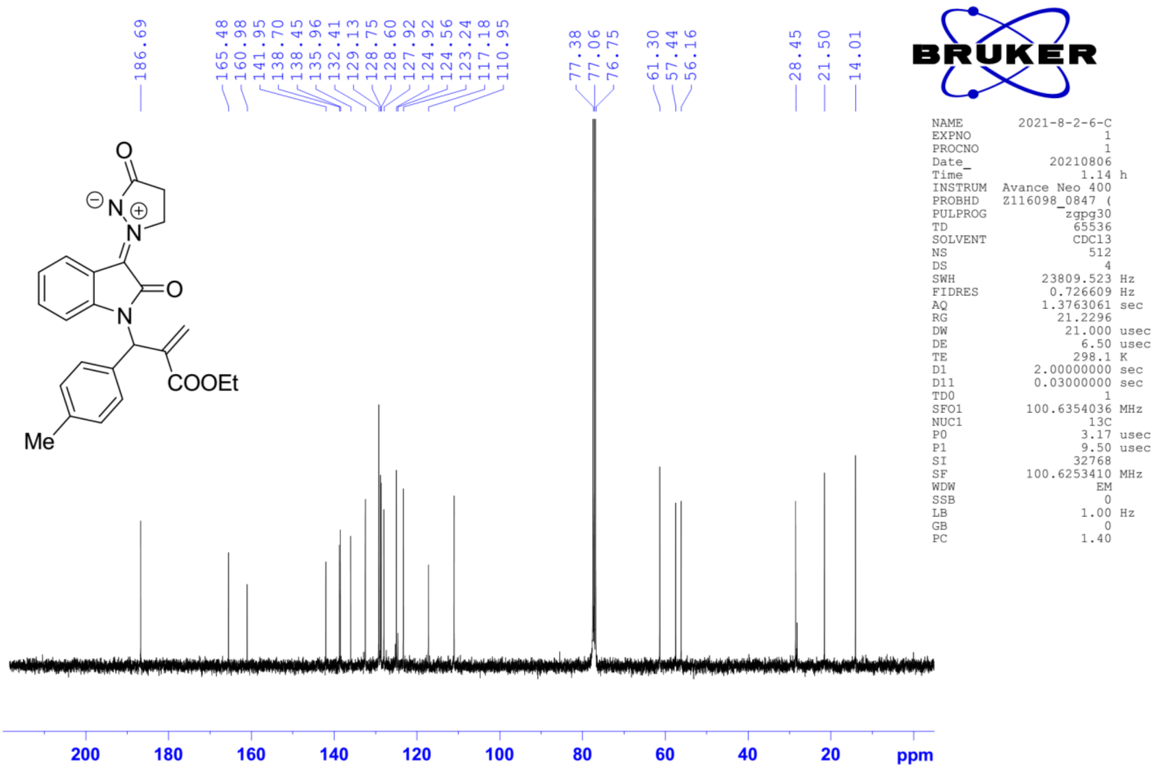

^{1}H and ^{13}C NMR Spectra for Compound **6e**

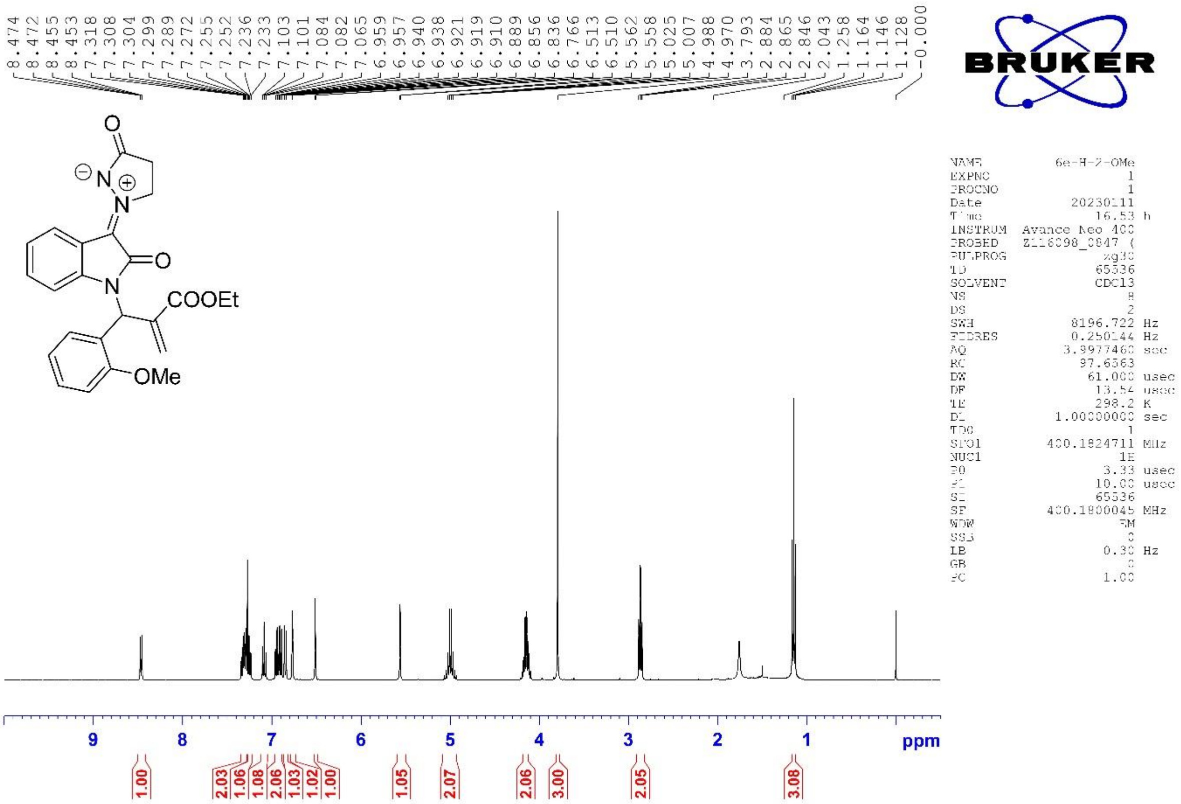

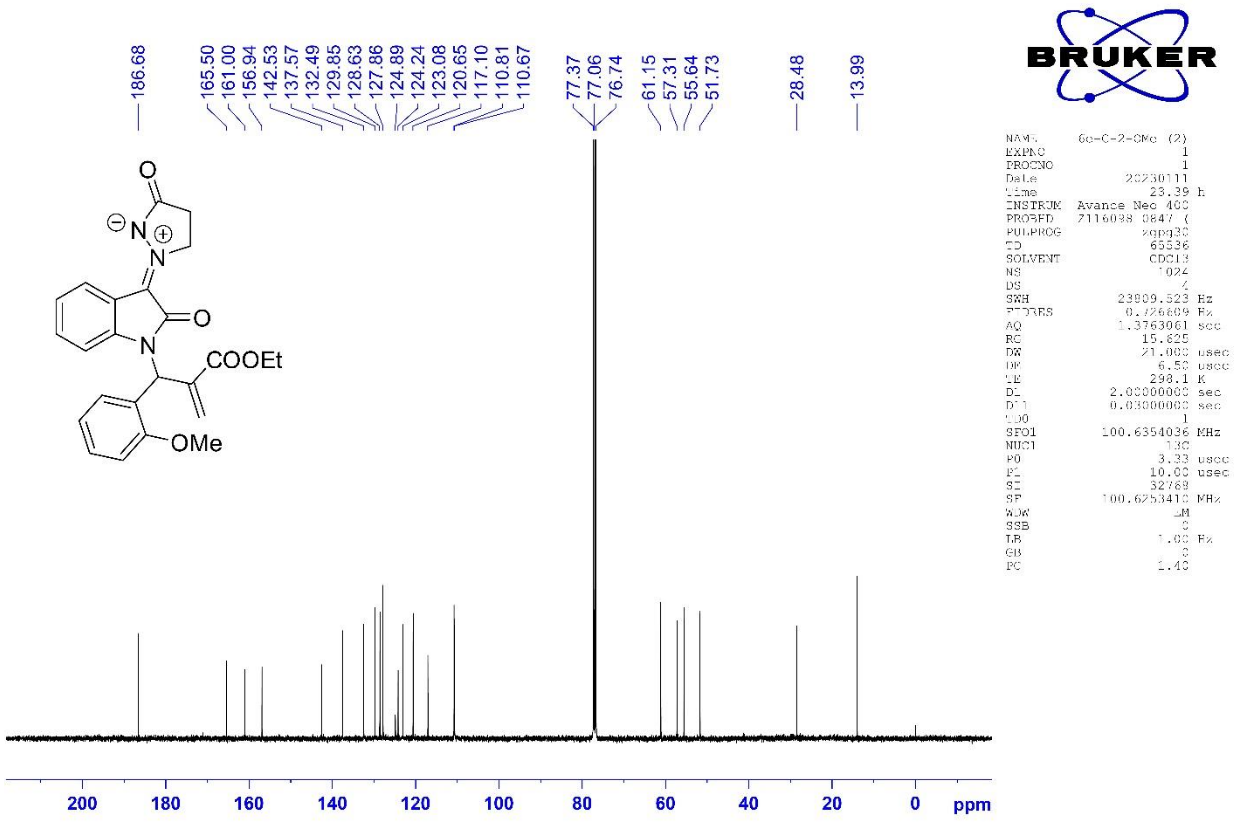

^{1}H and ^{13}C NMR Spectra for Compound **6f**

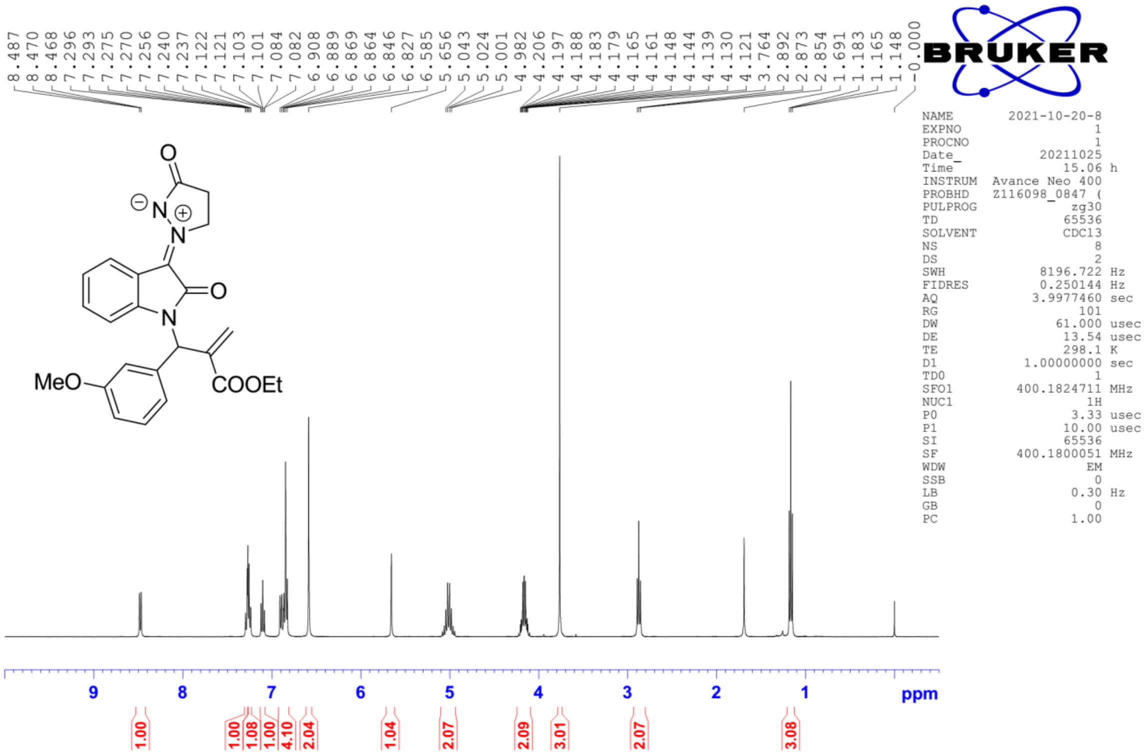

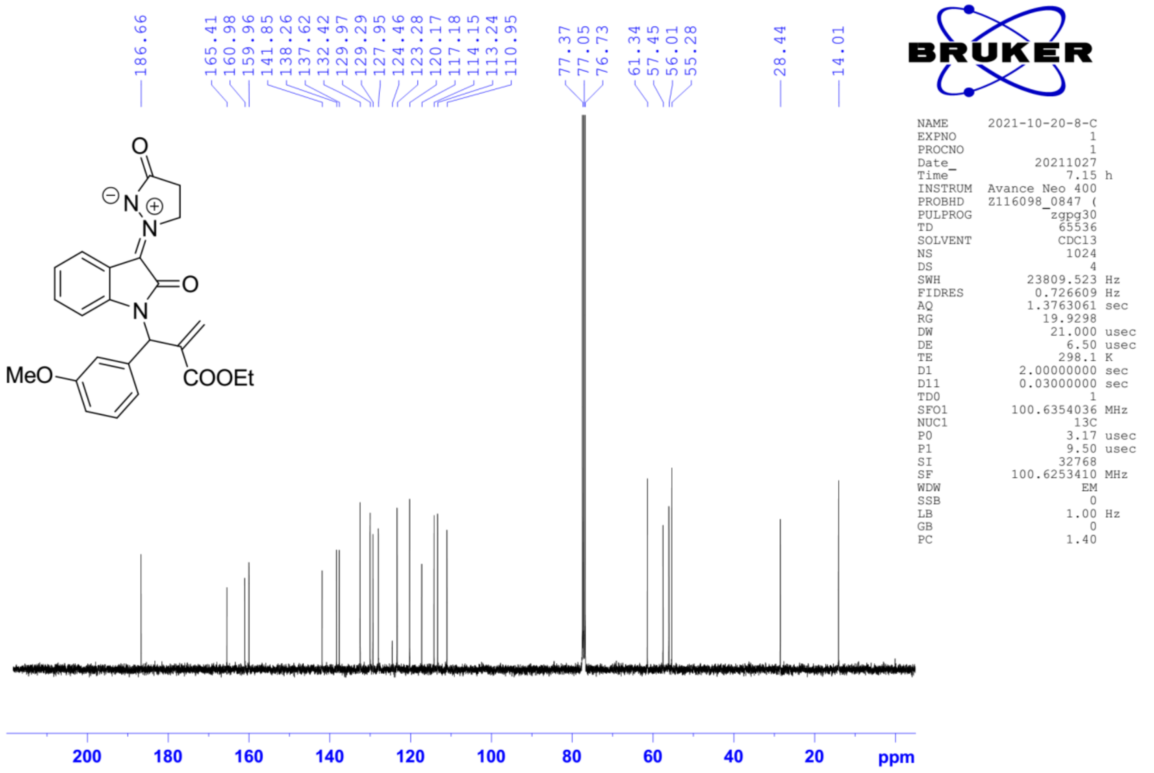

^{1}H NMR Spectra for Compound **6h** (mixed with an inseparable by-product)

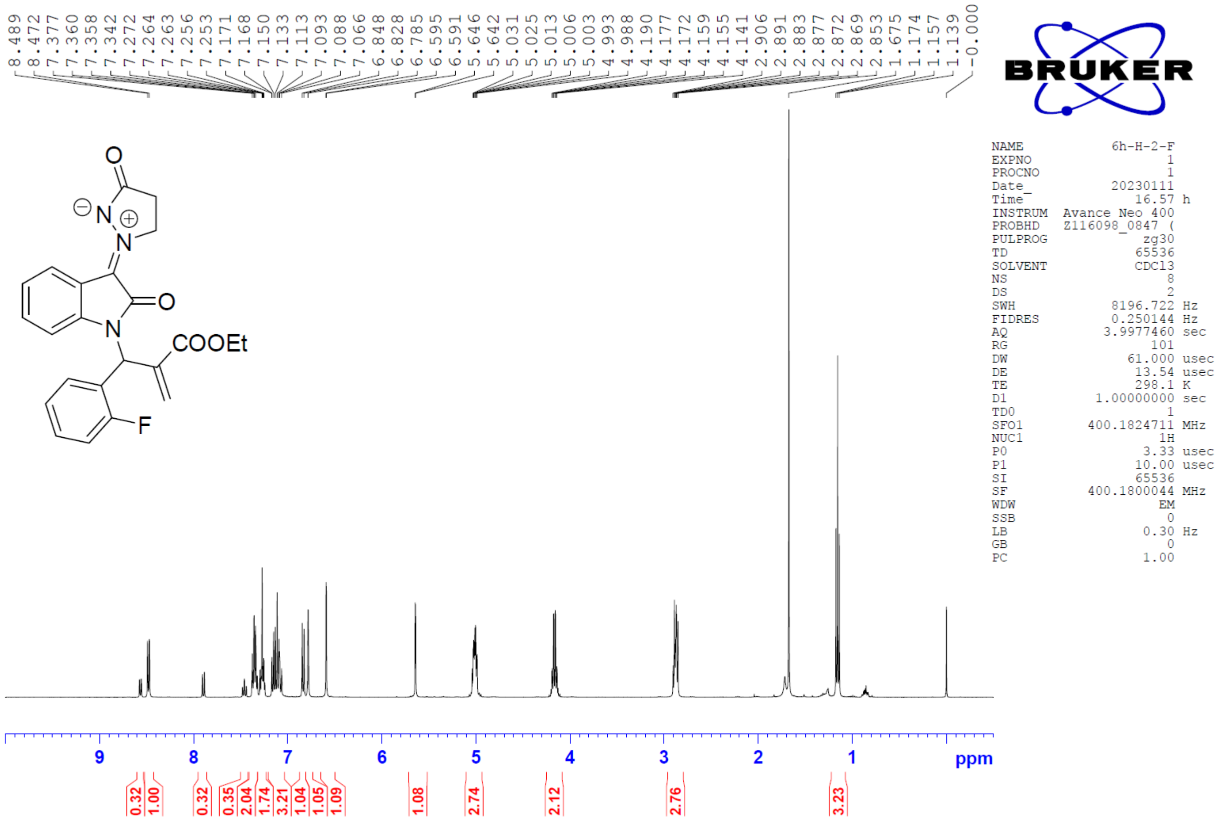

^{1}H, ^{13}C and ^{19}F NMR Spectra for Compound **6i**

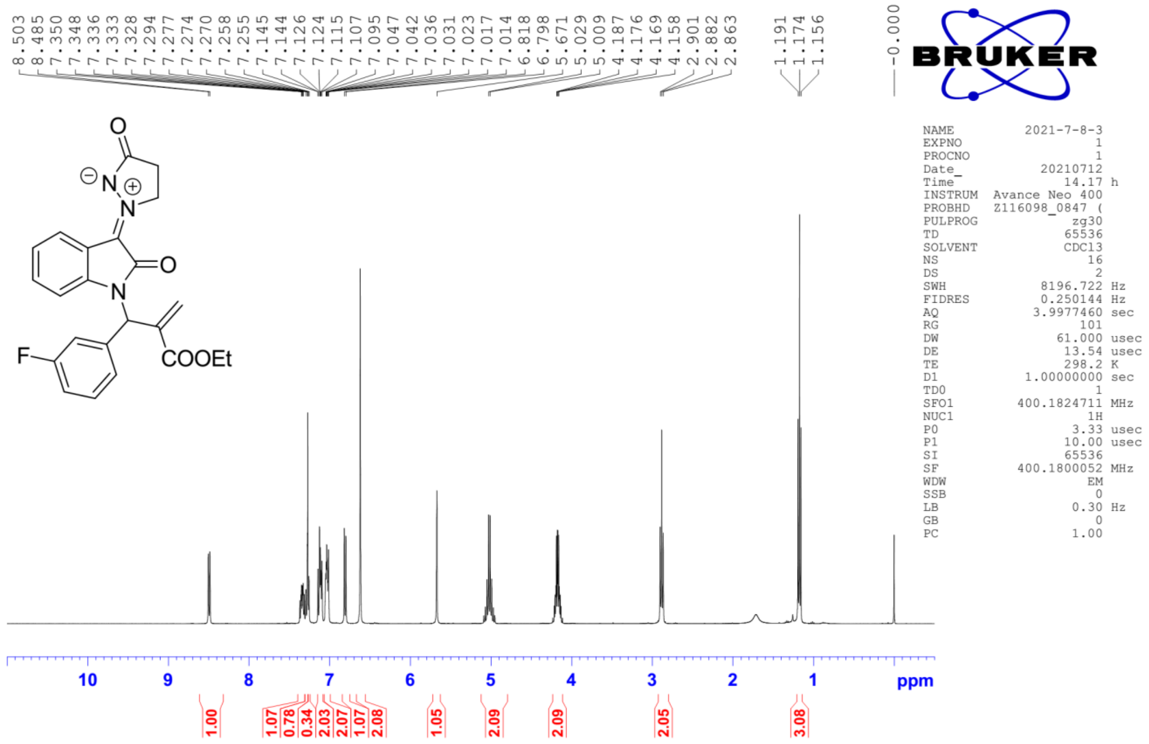

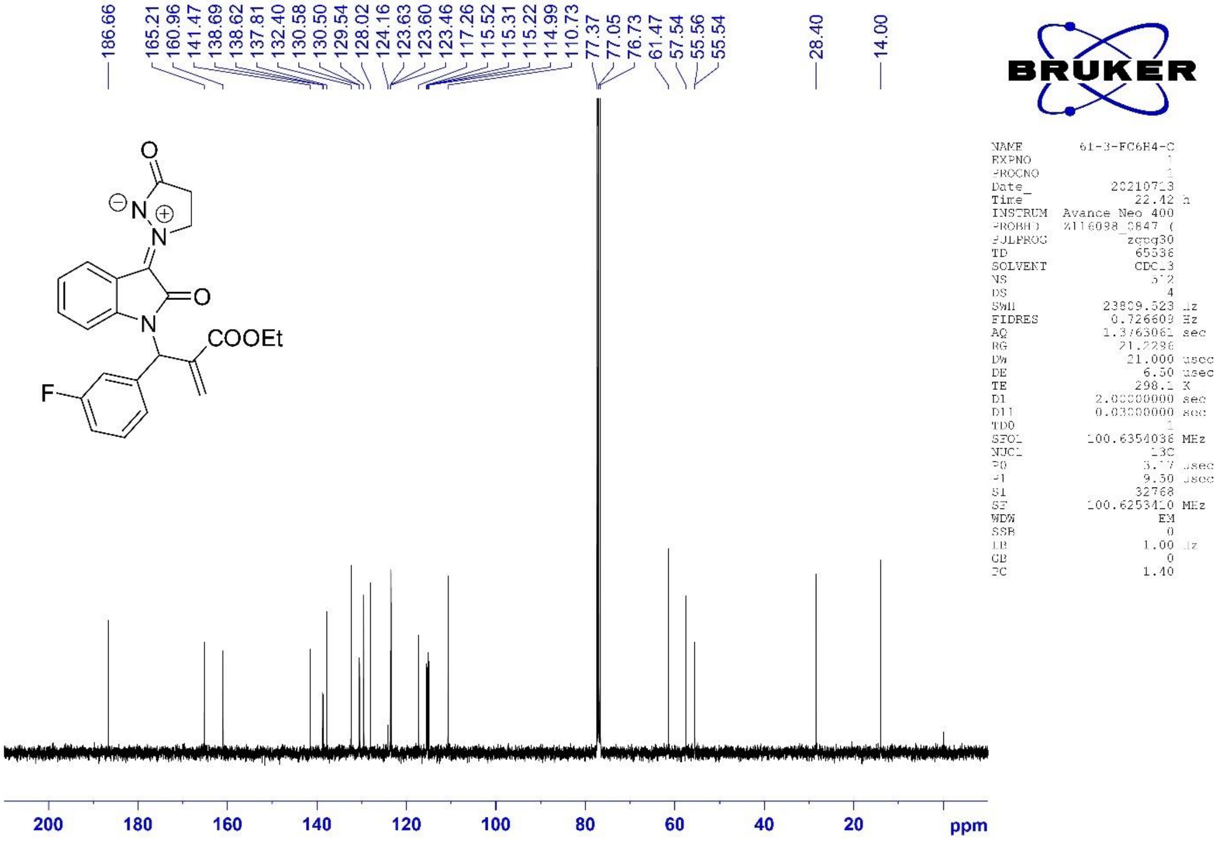

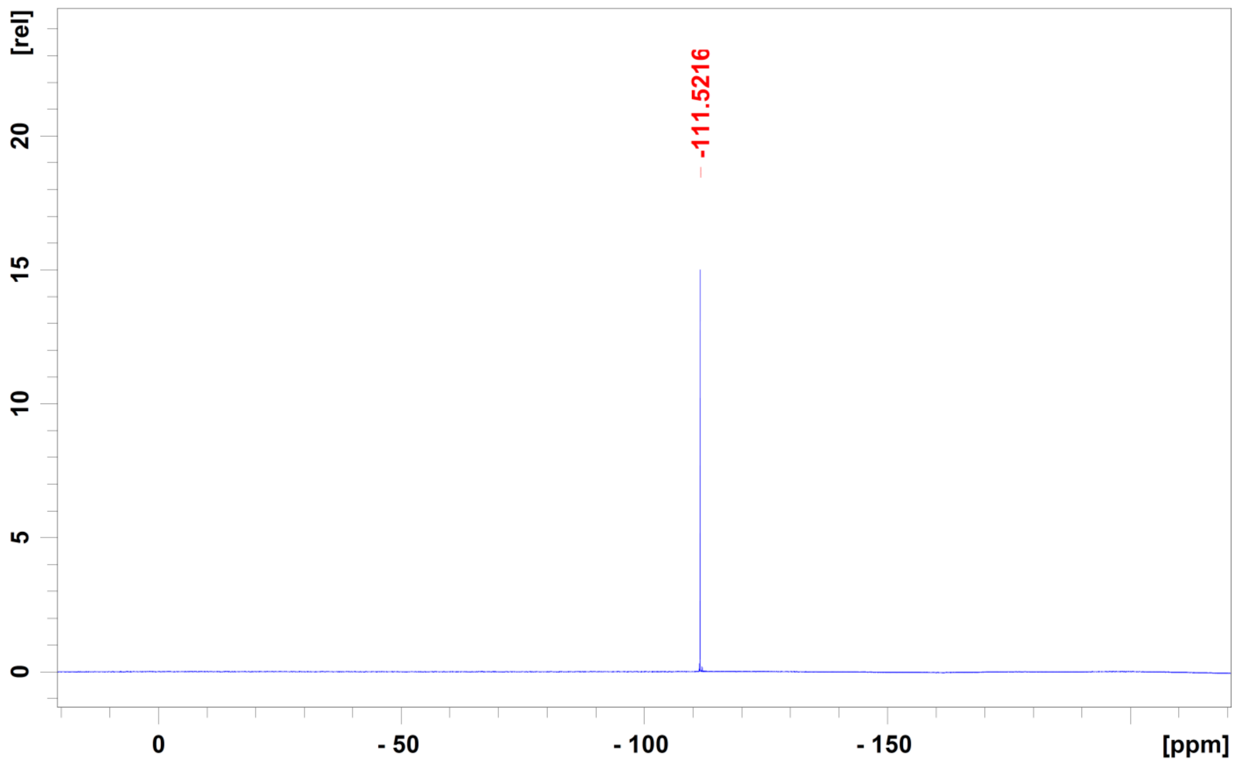

^{1}H NMR Spectra for Compound **6k** (mixed with an inseparable by-product)

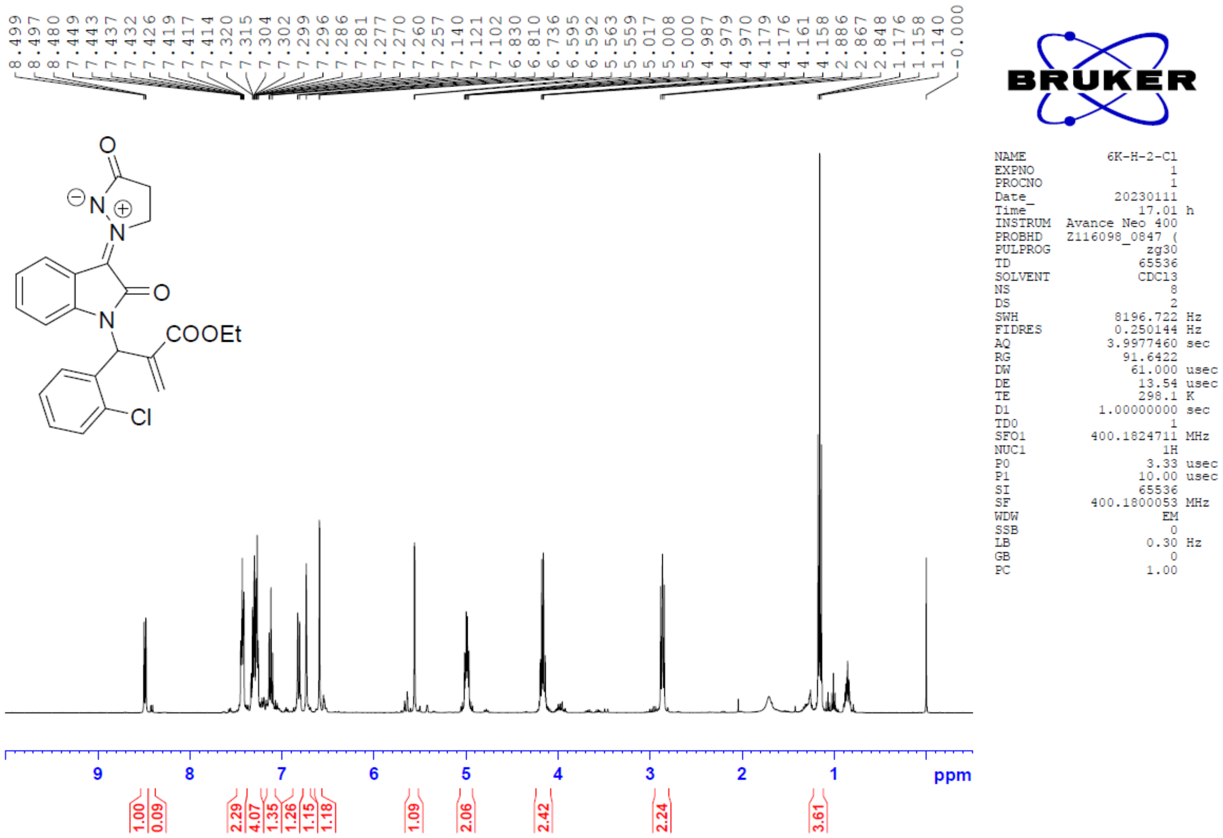

^{1}H and ^{13}C NMR Spectra for Compound **61**

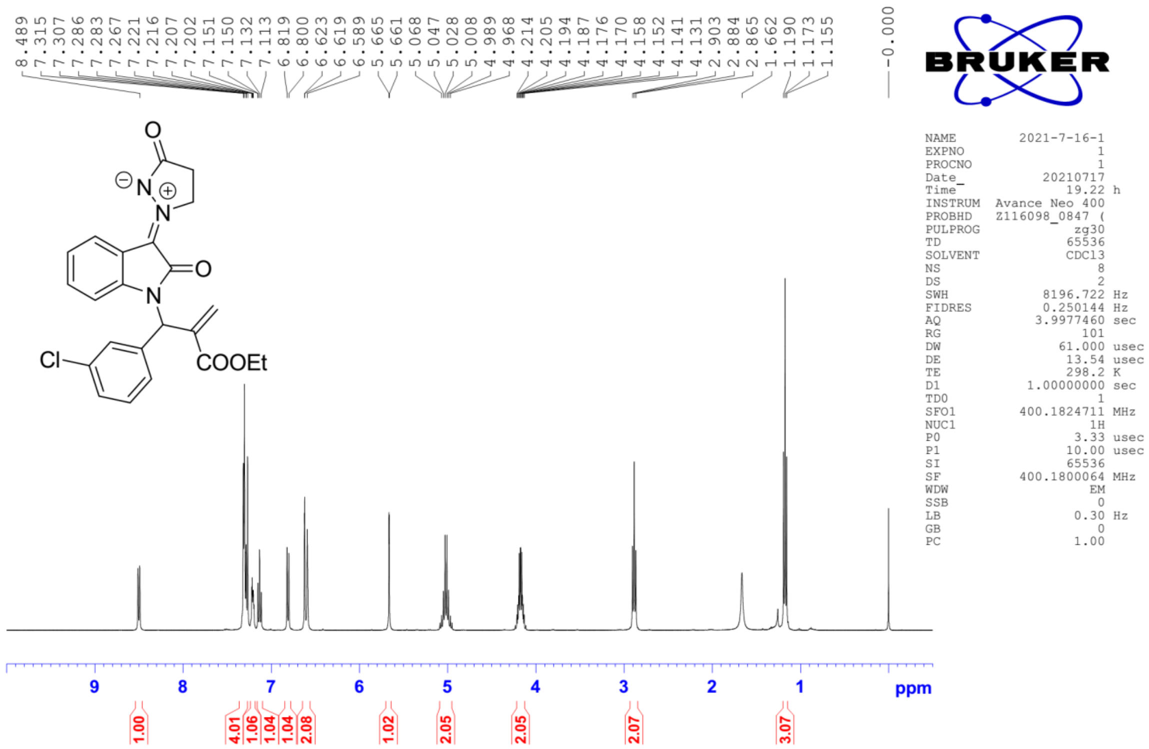

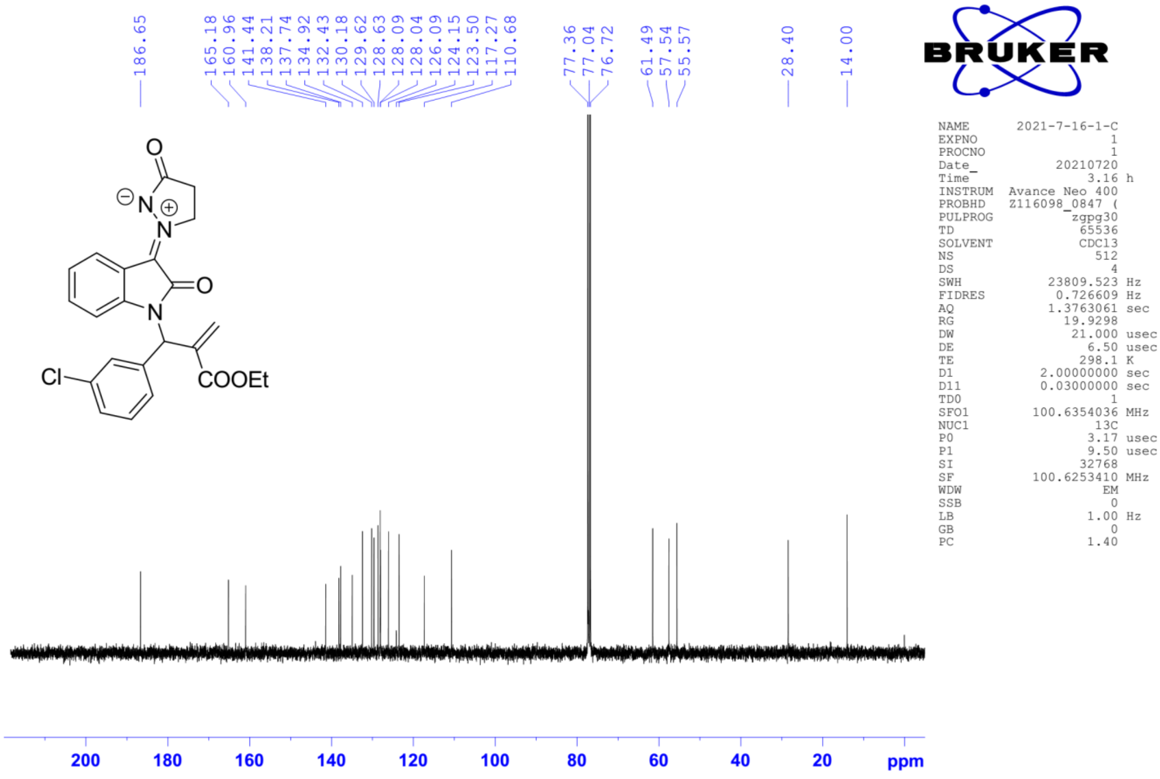

^{1}H and ^{13}C NMR Spectra for Compound **6m**

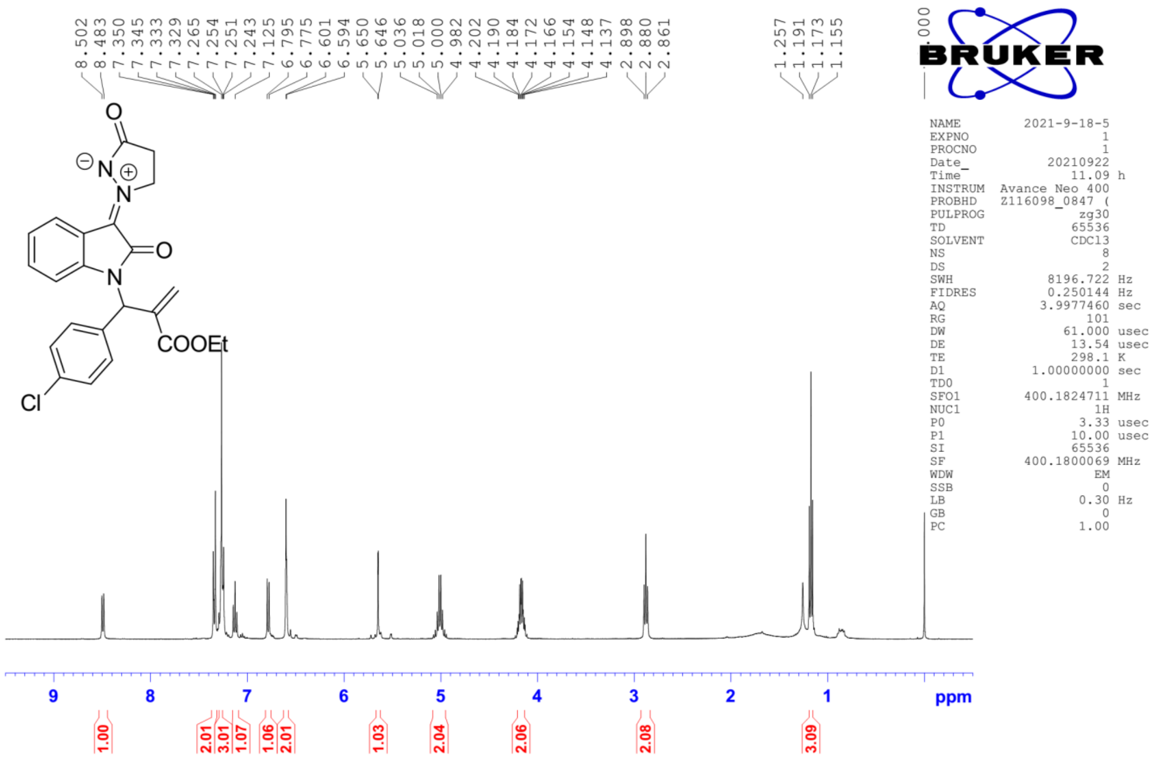

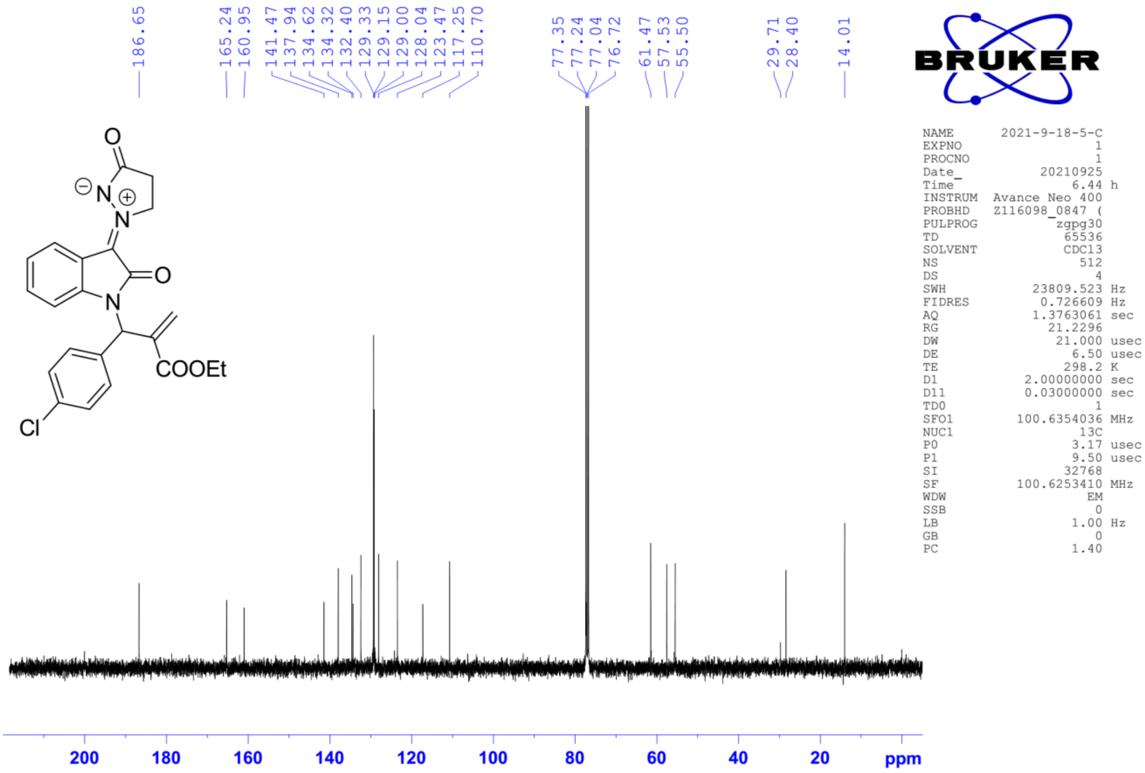

^{1}H and ^{13}C NMR Spectra for Compound **6o**

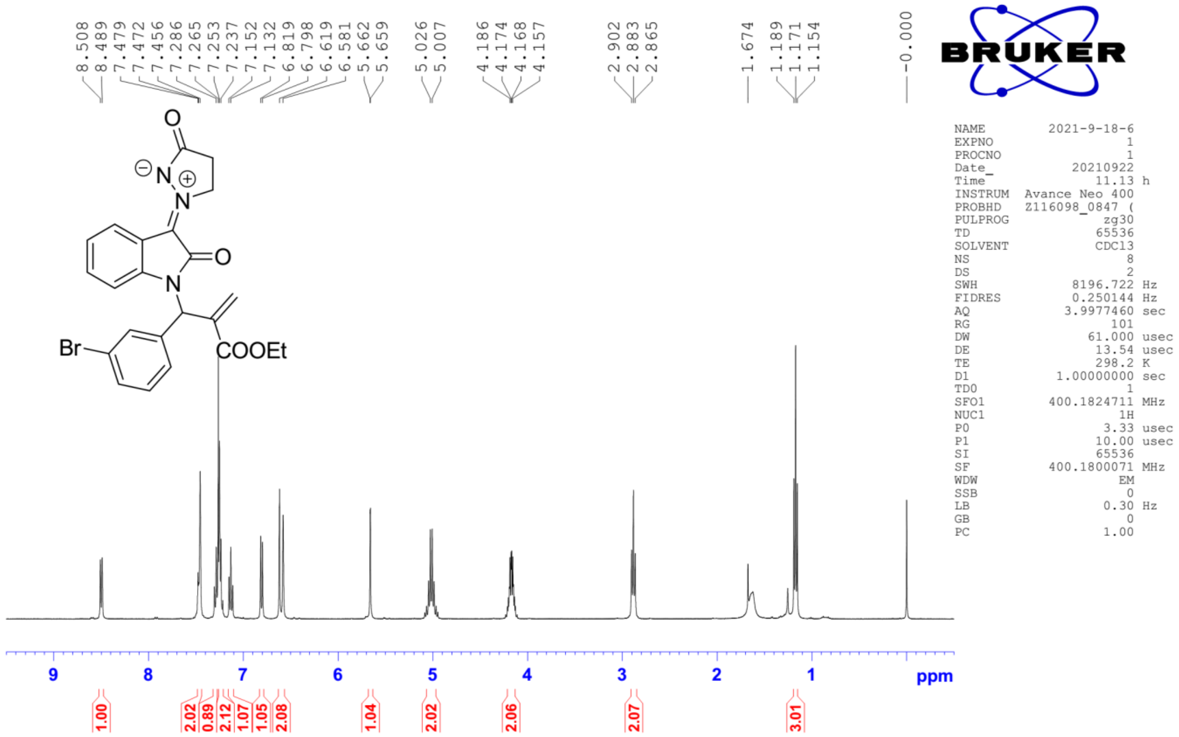

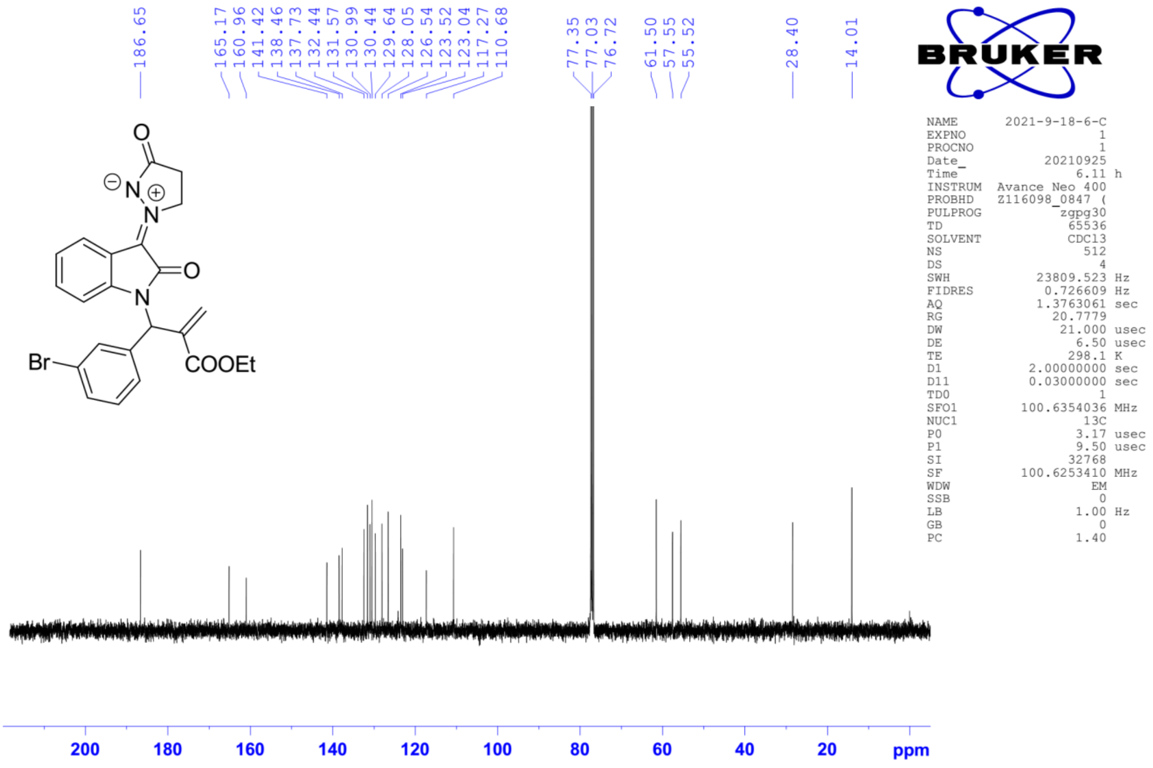

^{1}H and ^{13}C NMR Spectra for Compound **6p**

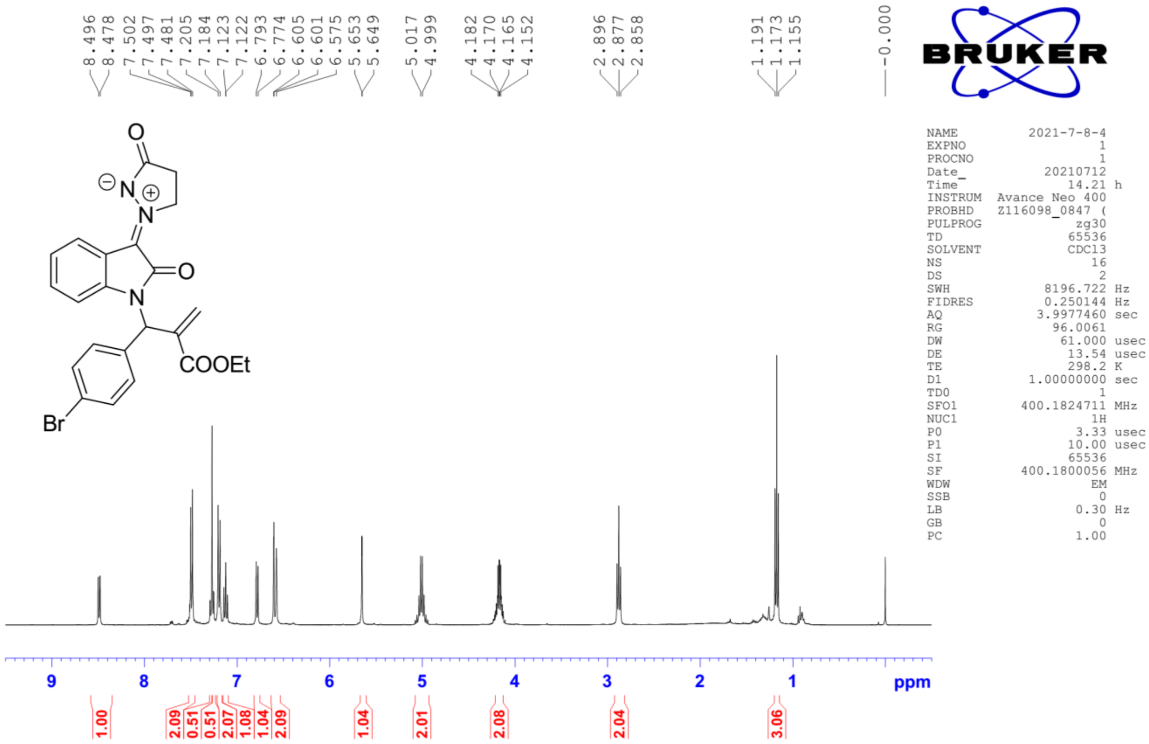

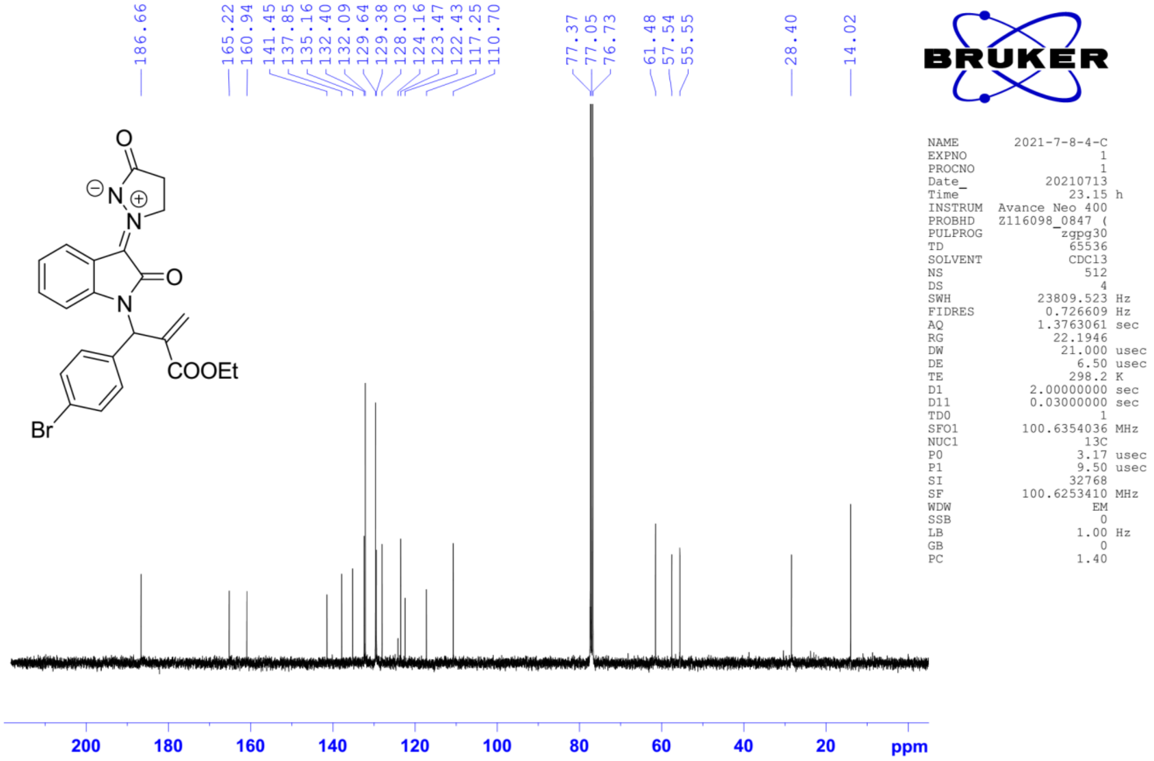

^{1}H and ^{13}C NMR Spectra for Compound **6r**

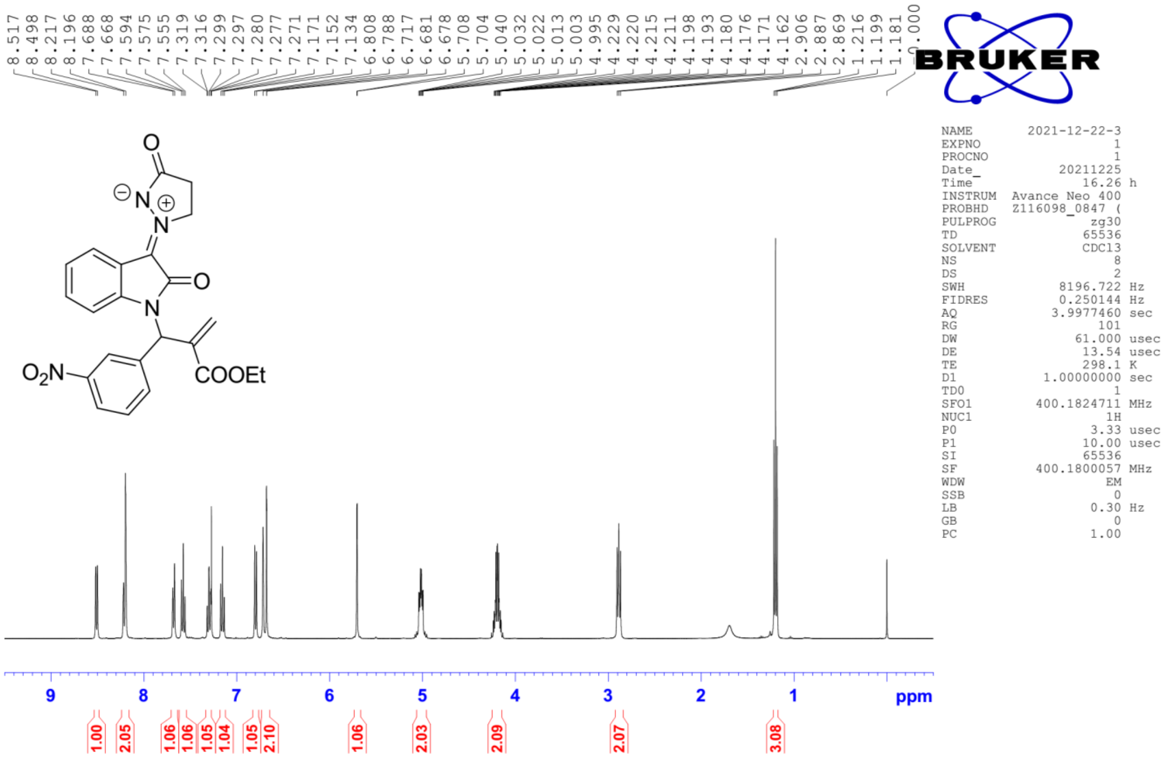

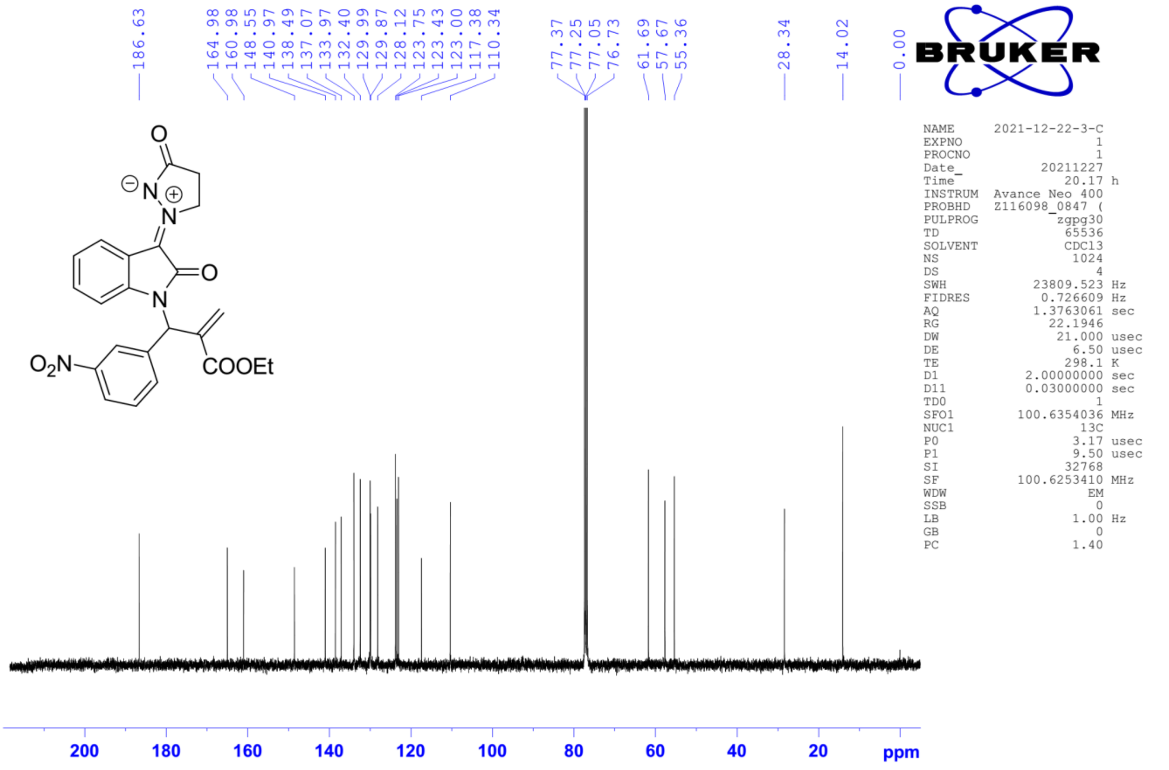

^{1}H and ^{13}C NMR Spectra for Compound **6s**

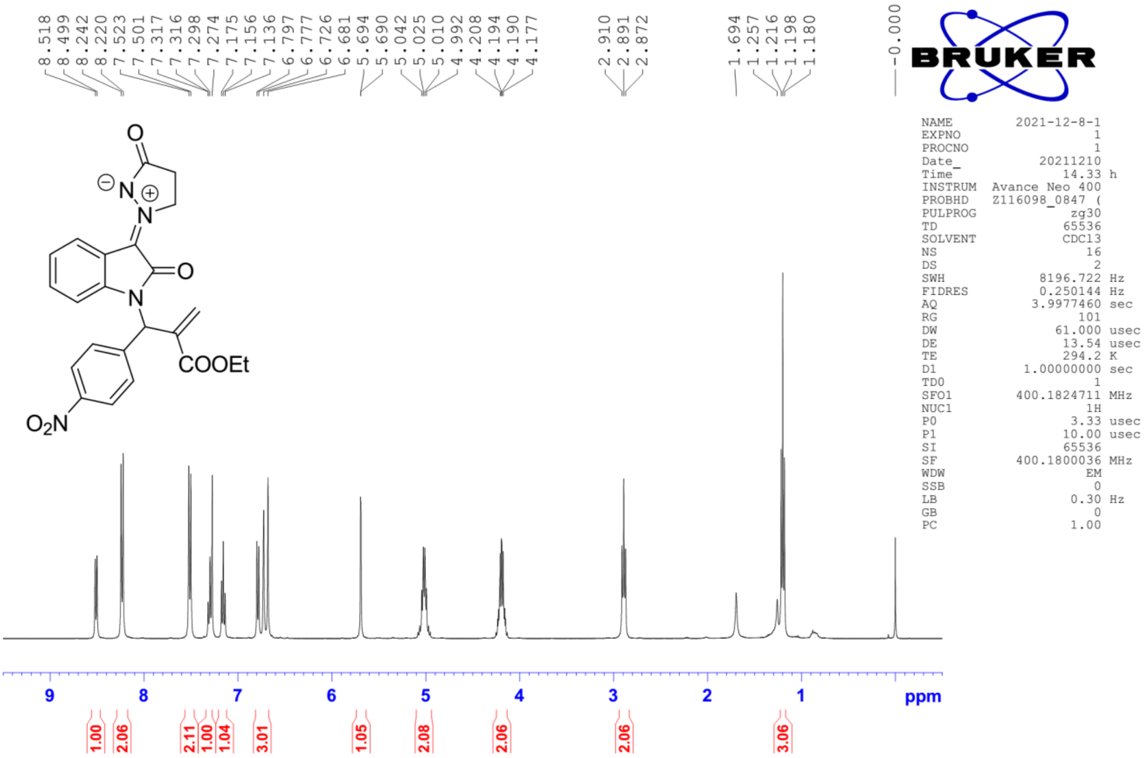

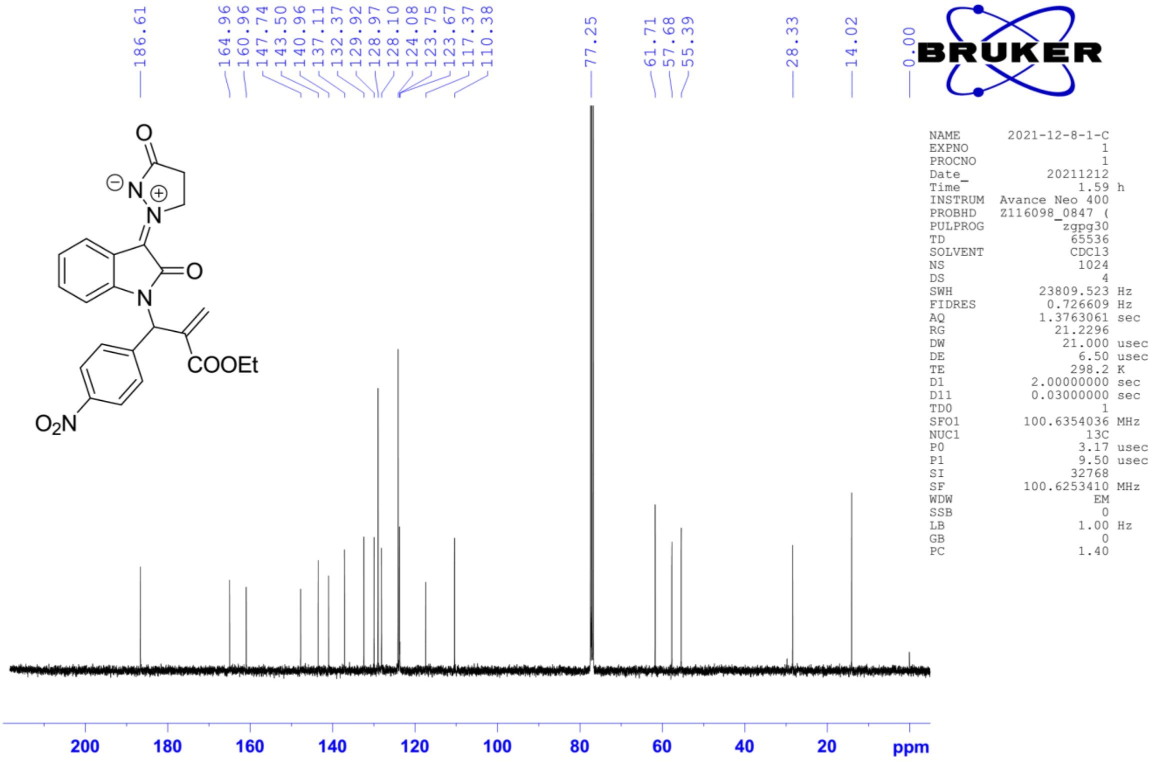

^{1}H and ^{13}C NMR Spectra for Compound **6’t**

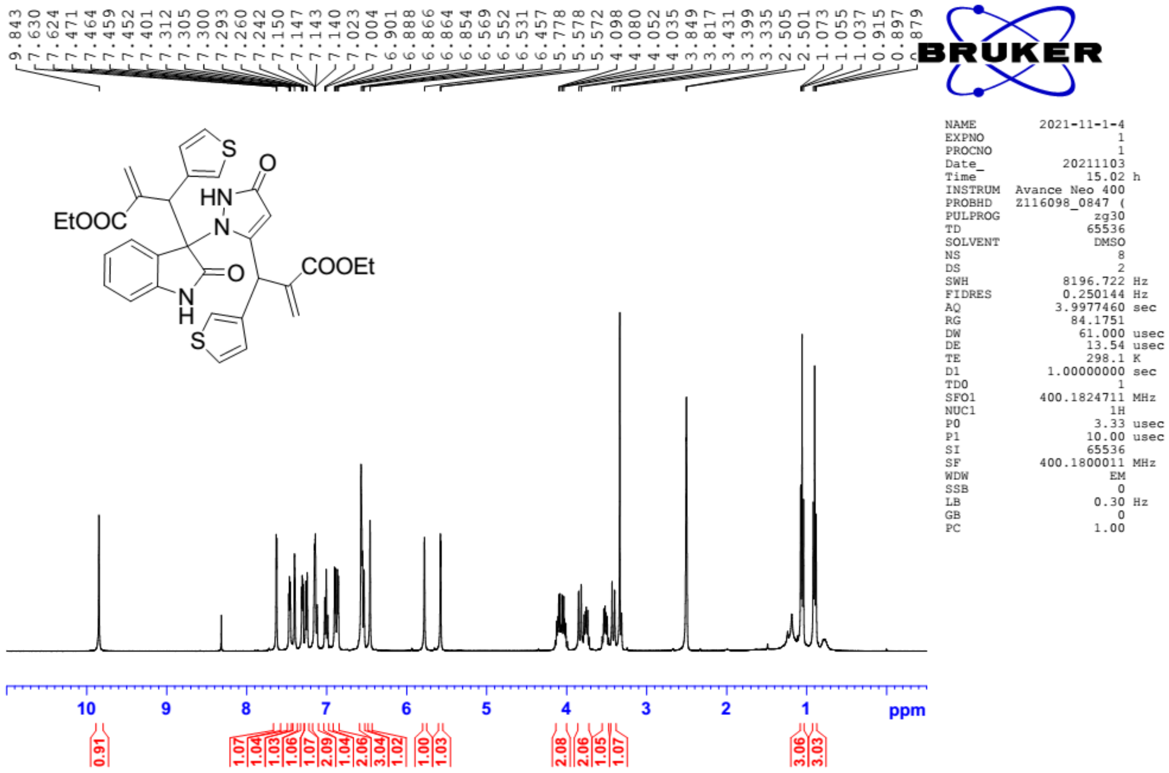

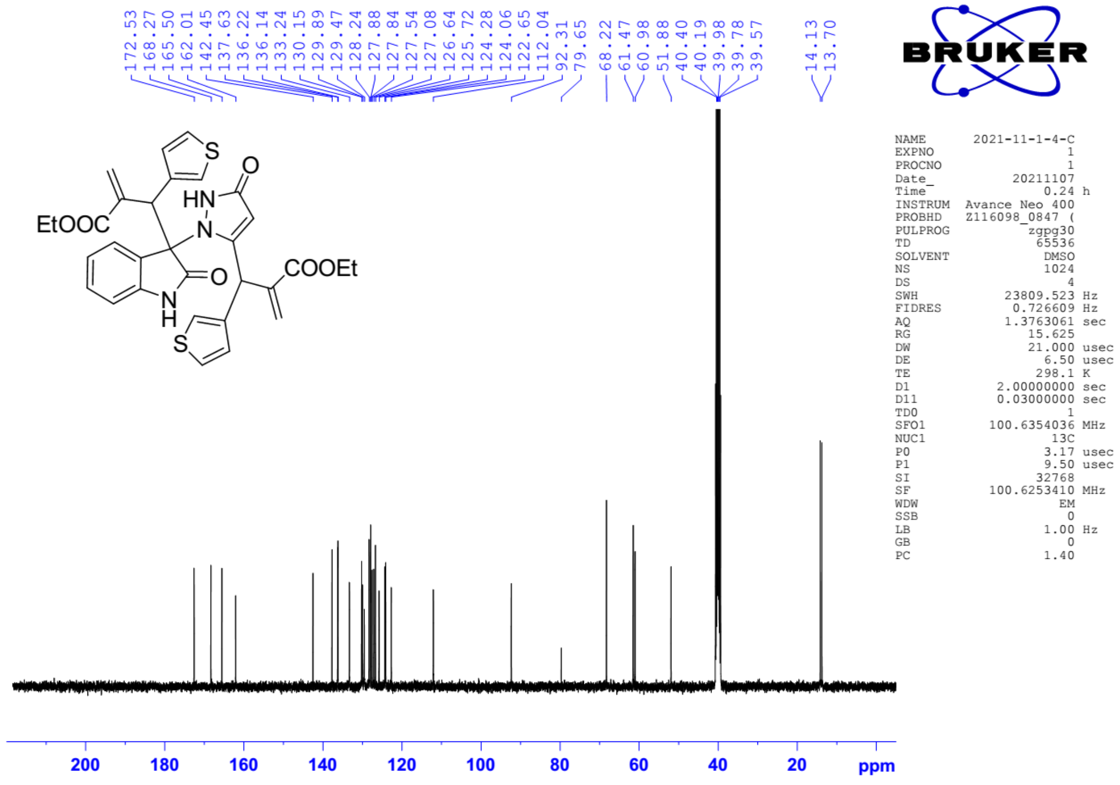

^{1}H and ^{13}C NMR Spectra for Compound **8b**

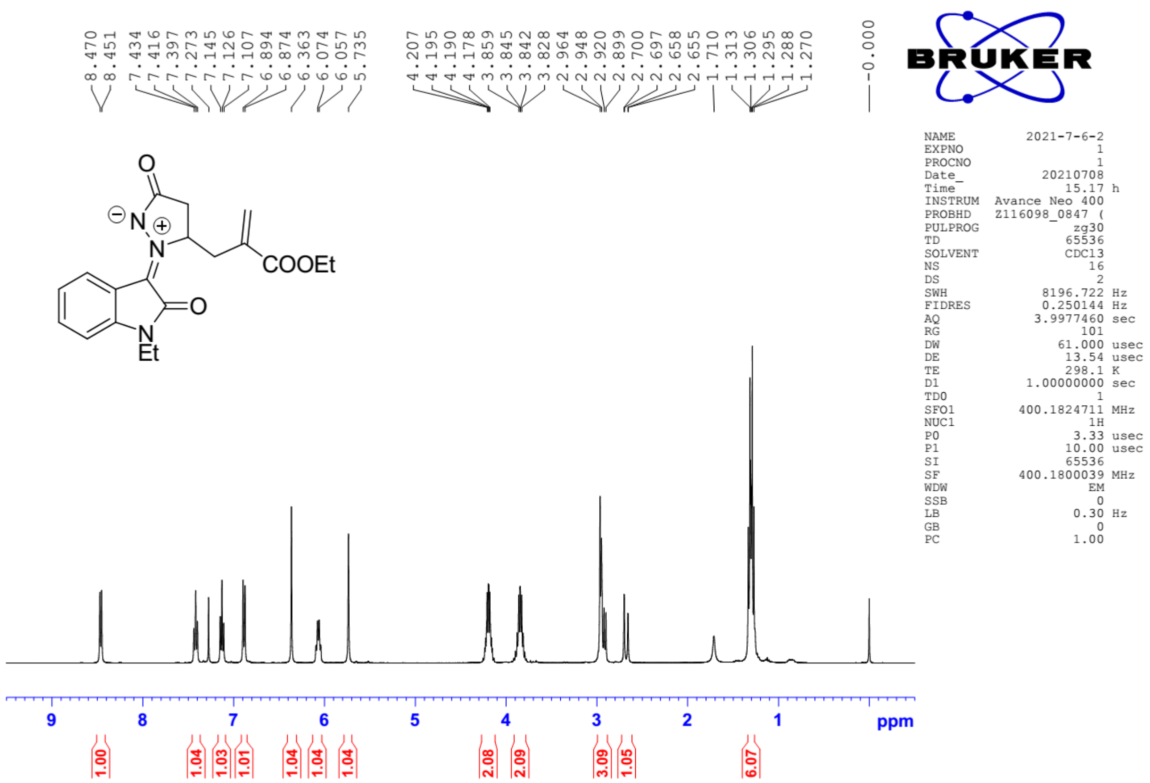

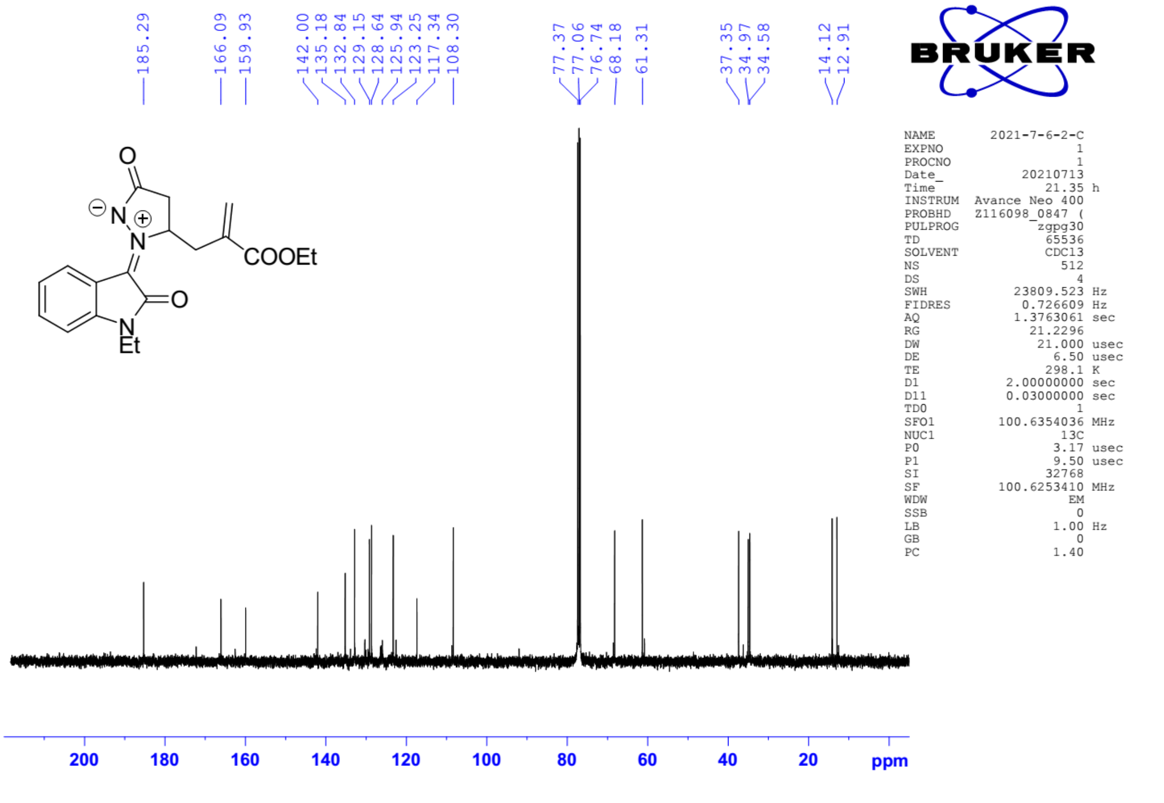

¹H NMR Spectra for Compound **8c** (a known compound)

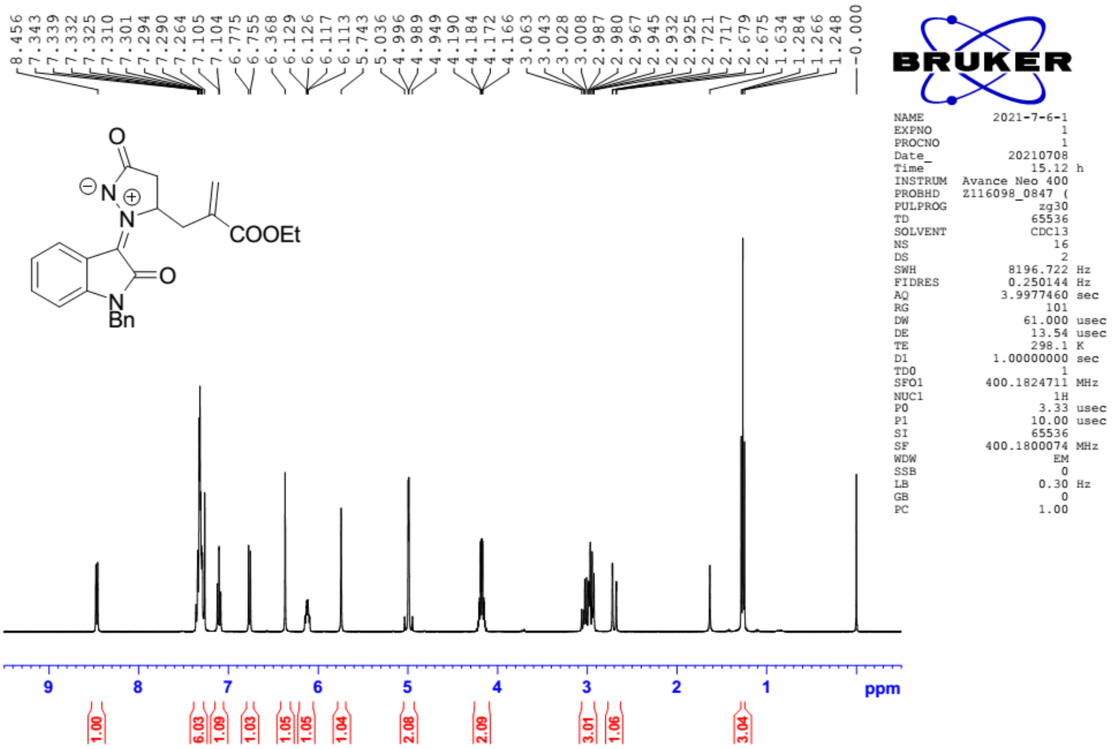

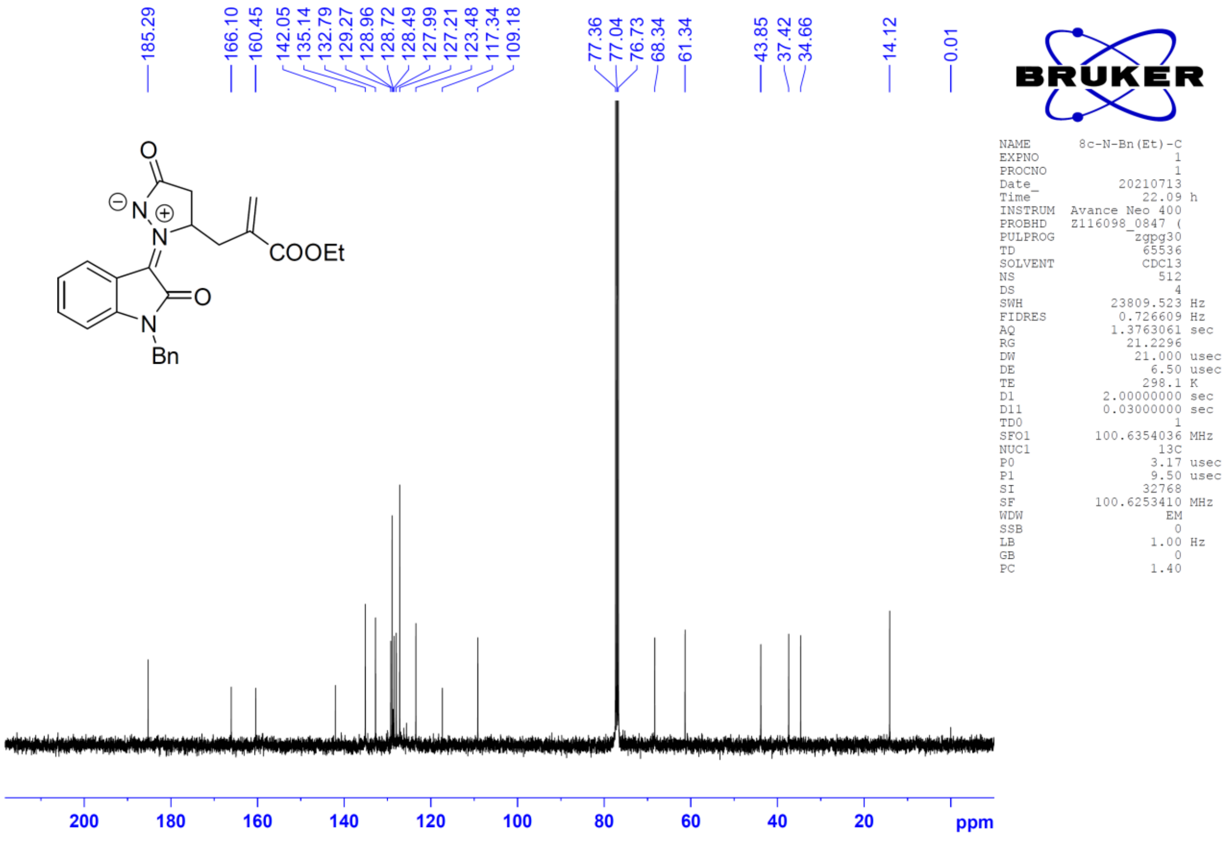

^{1}H and ^{13}C NMR Spectra for Compound **8d**

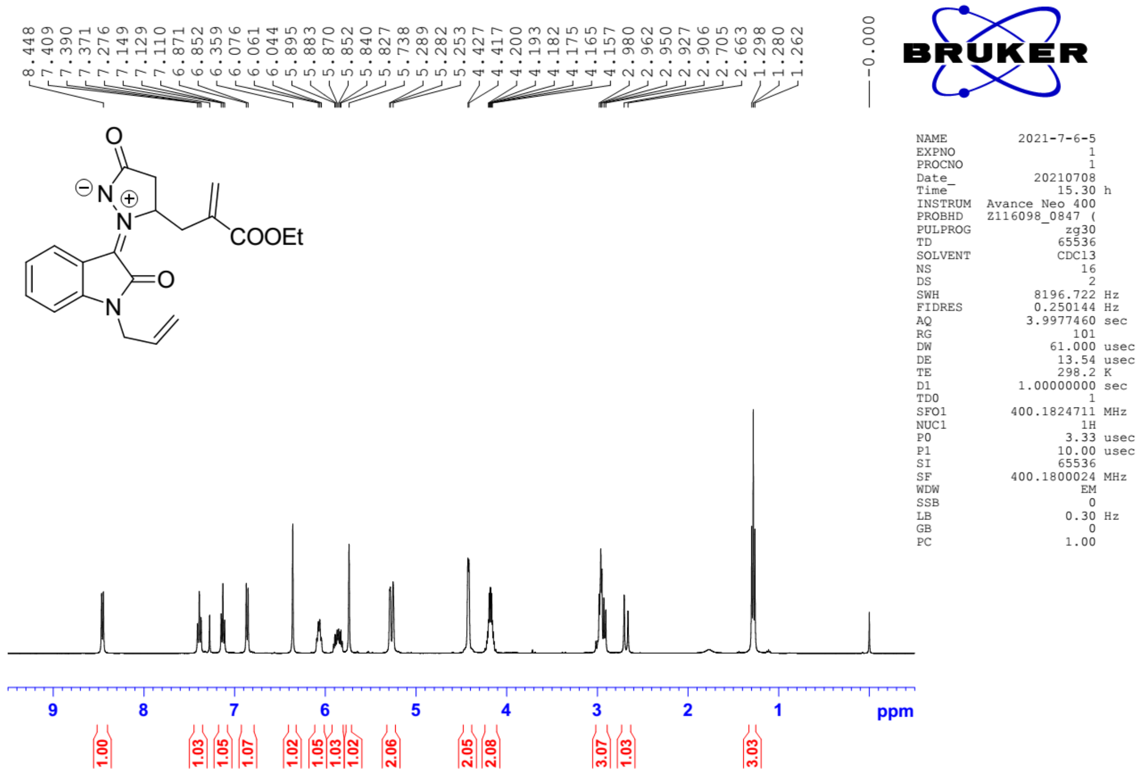

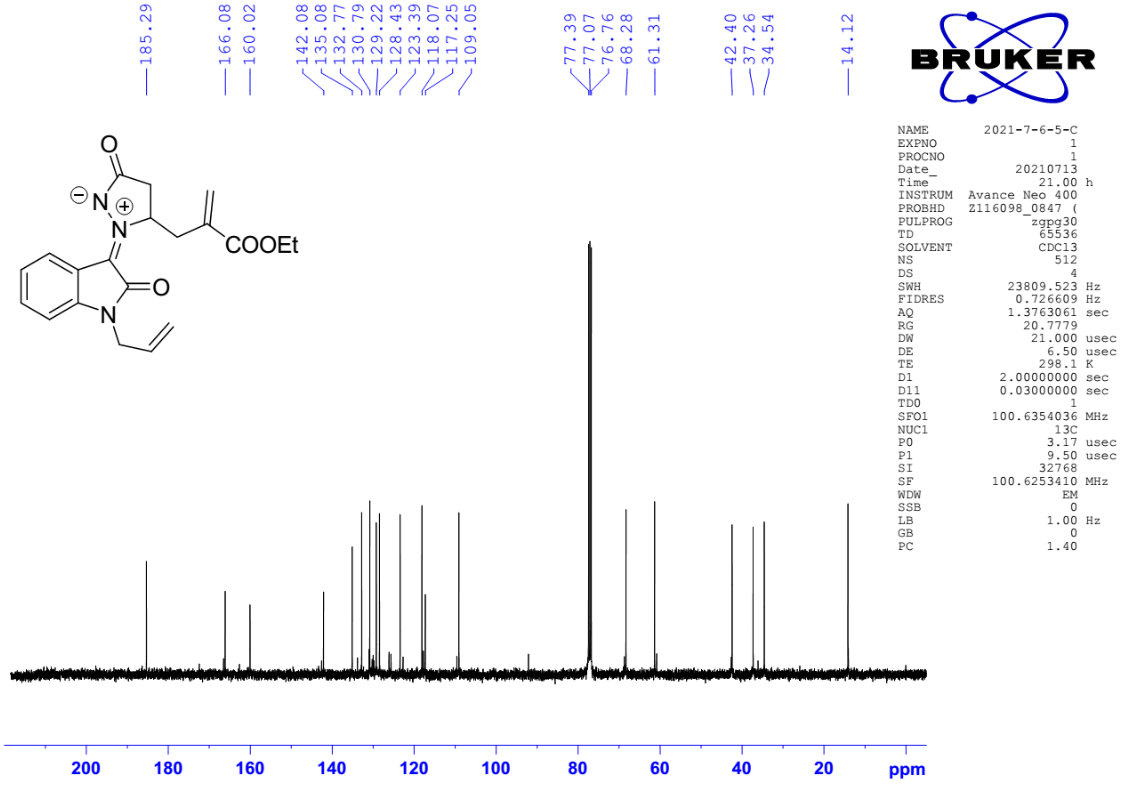

^{1}H and ^{13}C NMR Spectra for Compound **8e**

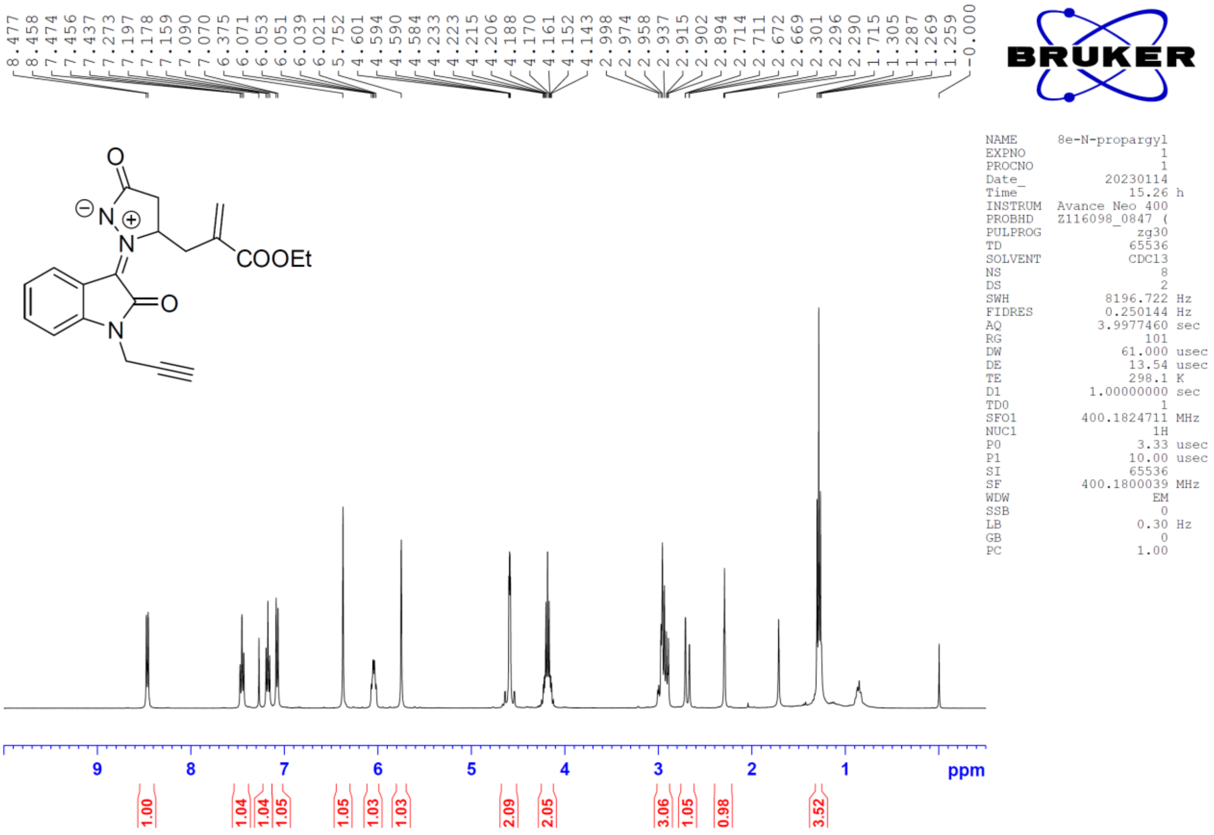

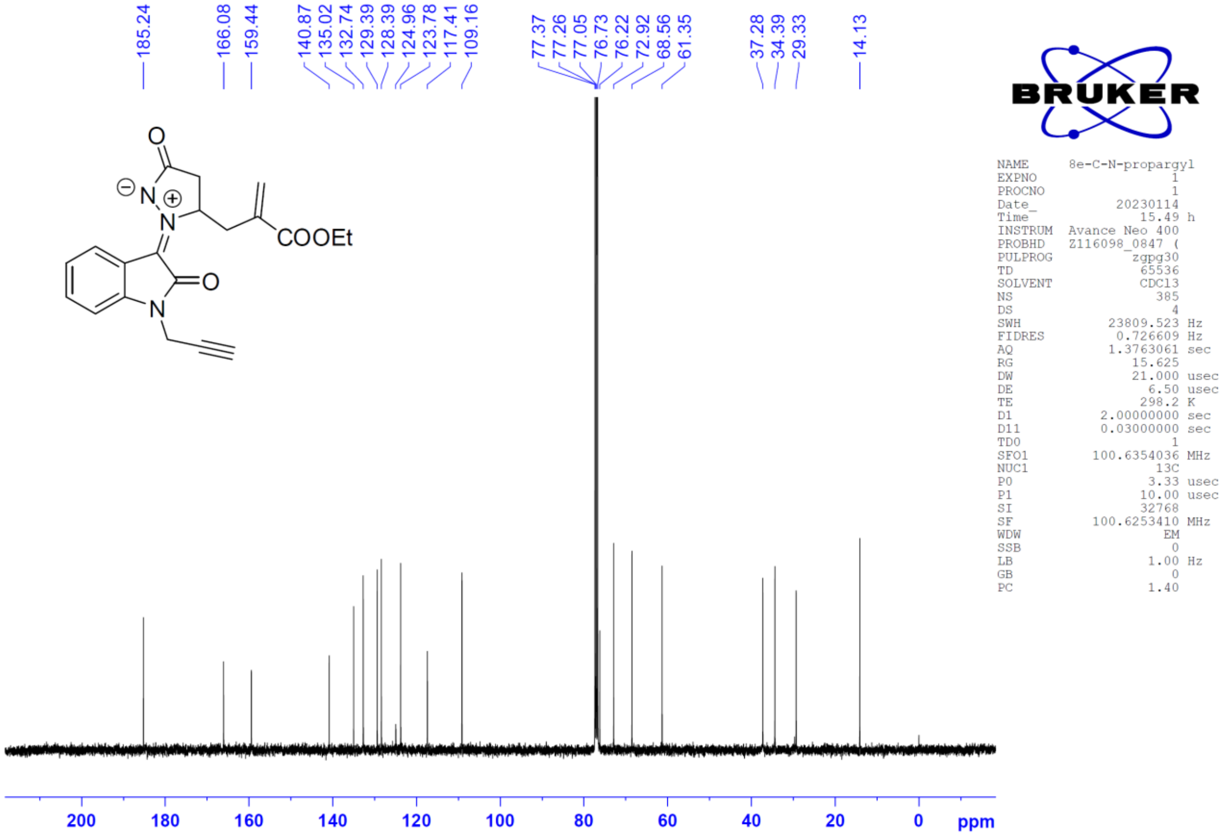

^{1}H and ^{13}C NMR Spectra for Compound **8f**

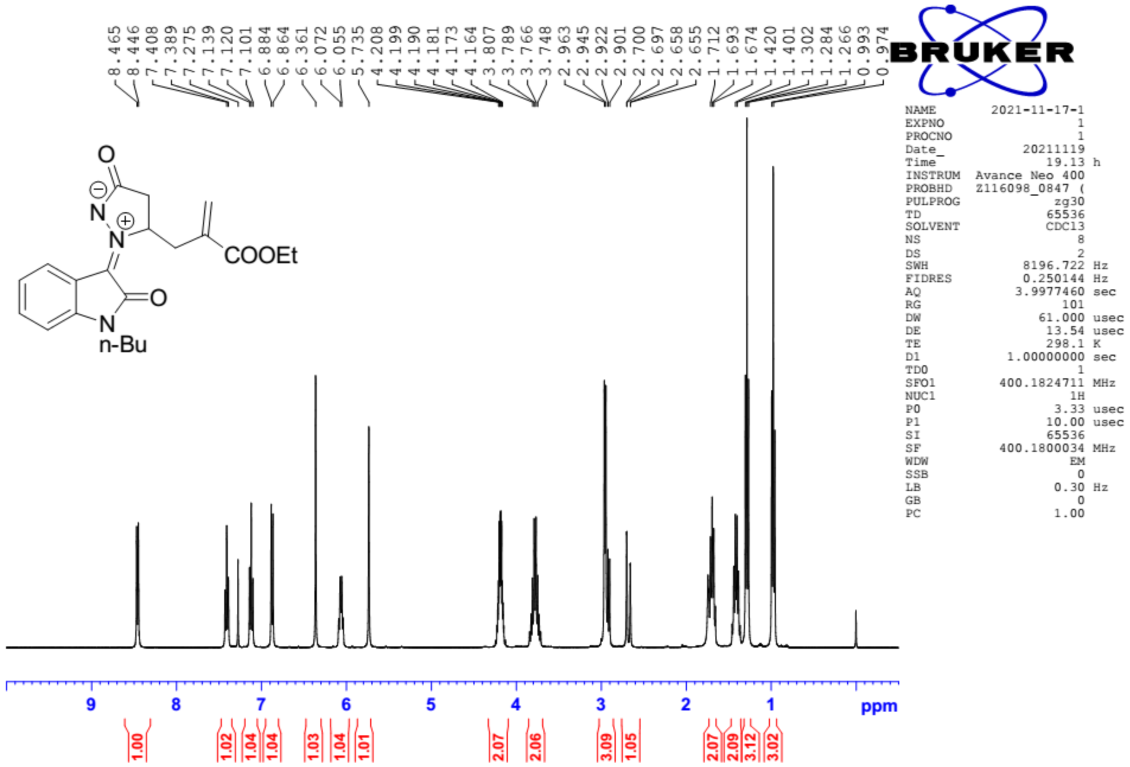

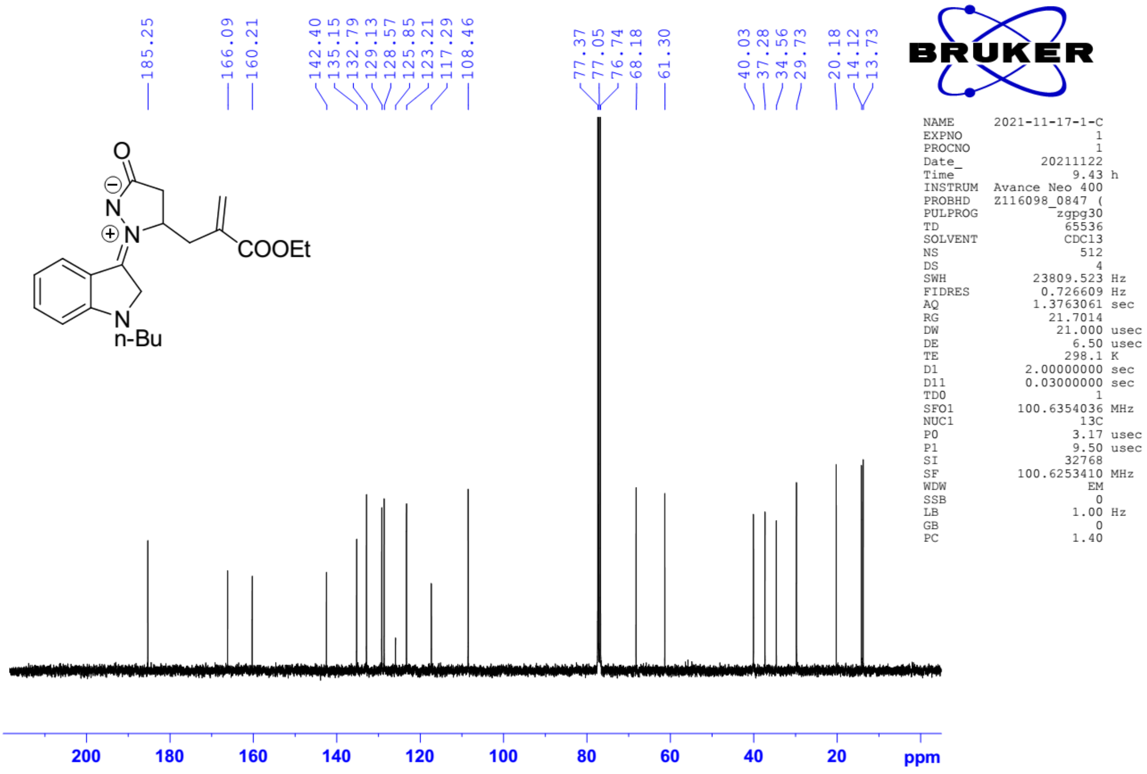

^{1}H and ^{13}C NMR Spectra for Compound **8g**

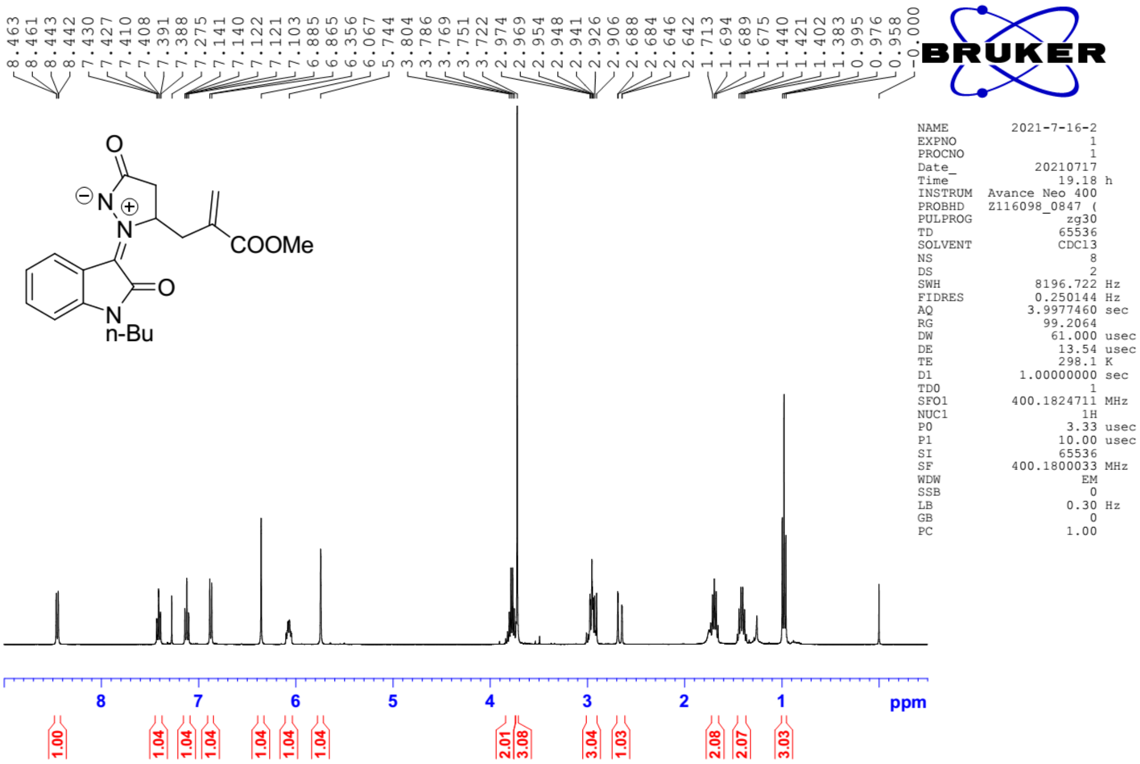

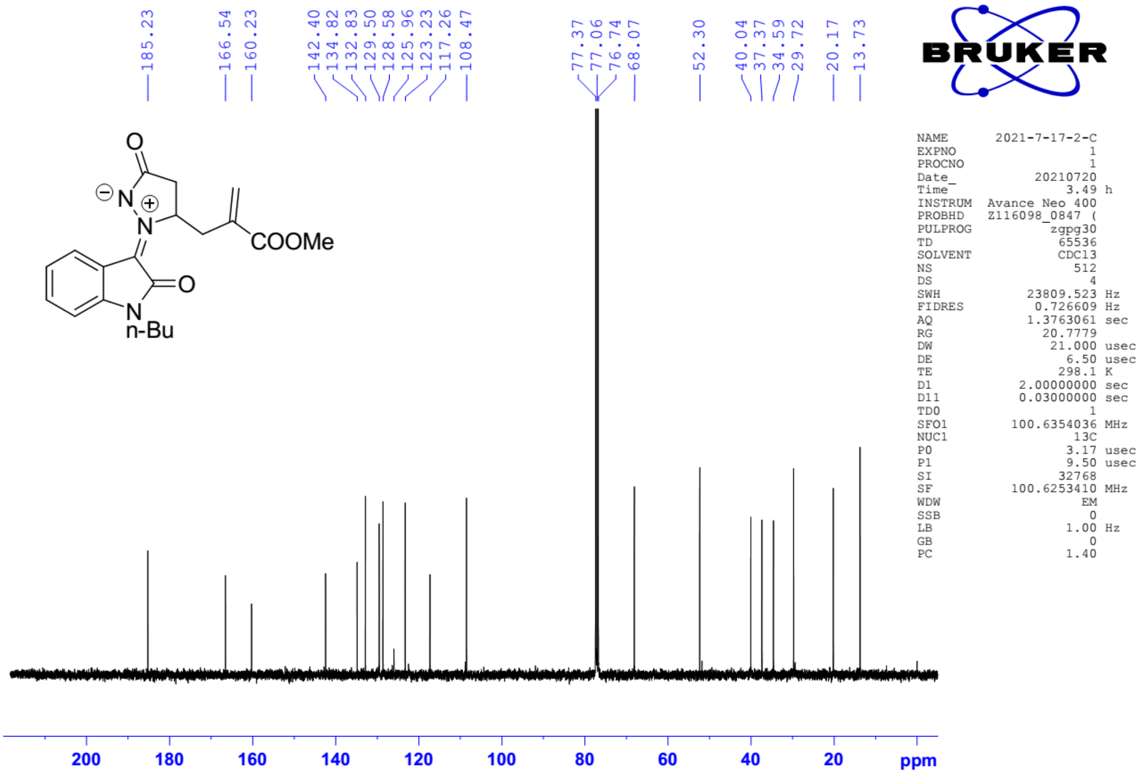

^{1}H and ^{13}C NMR Spectra for Compound **8h**

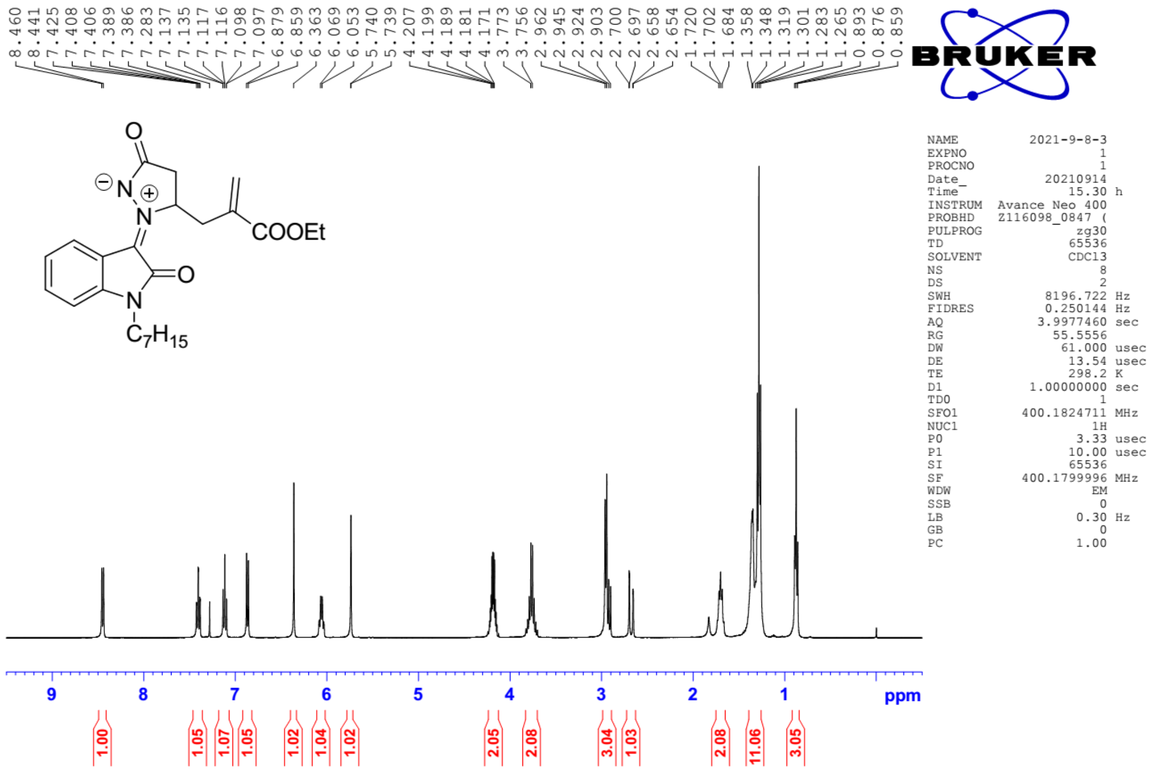

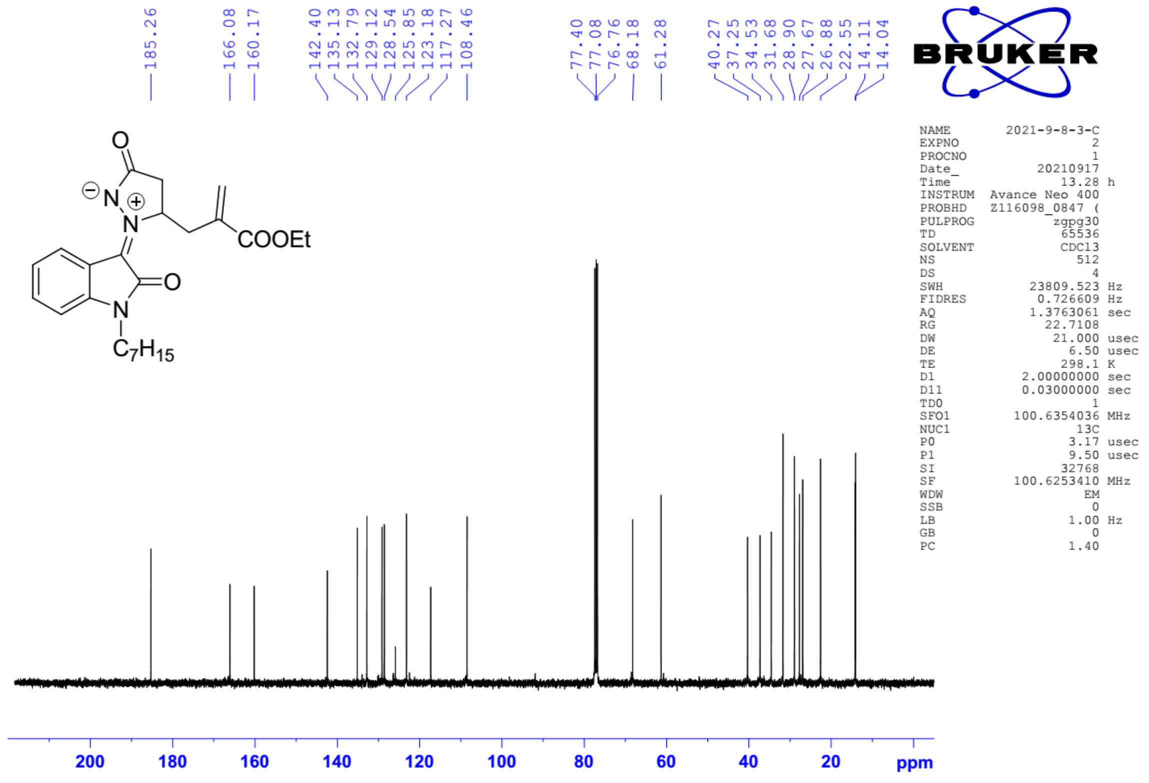

^{1}H and ^{13}C NMR Spectra for Compound **8i**

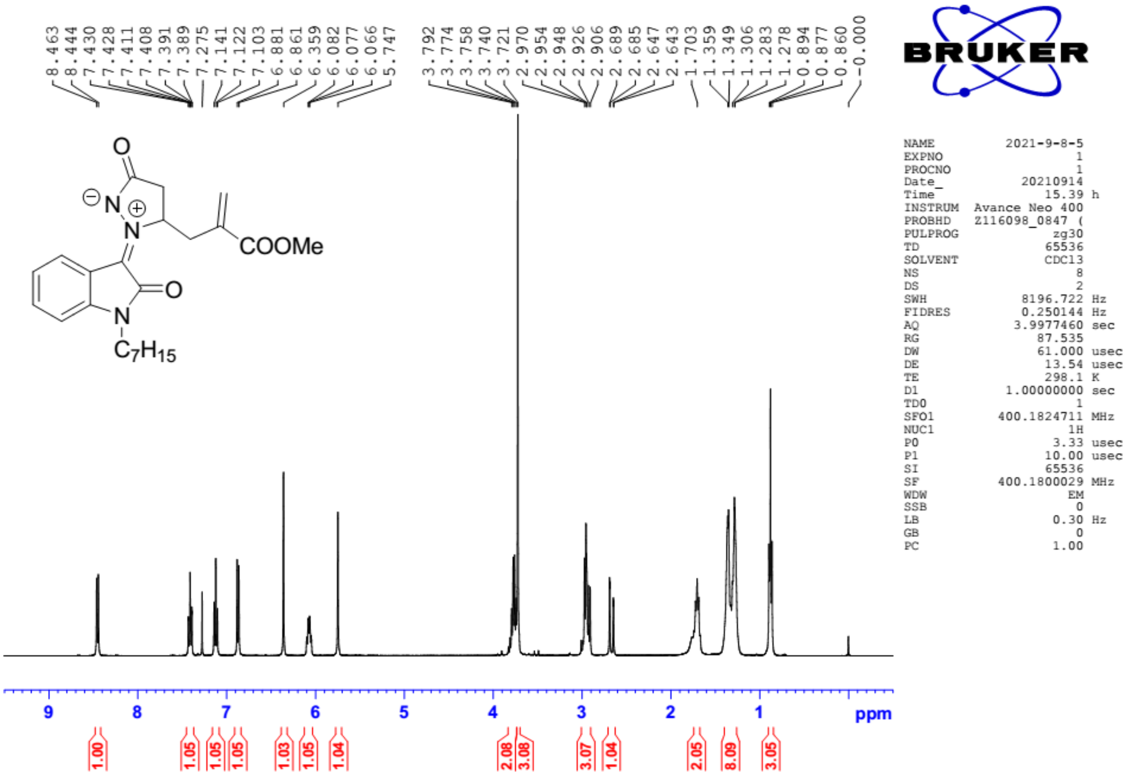

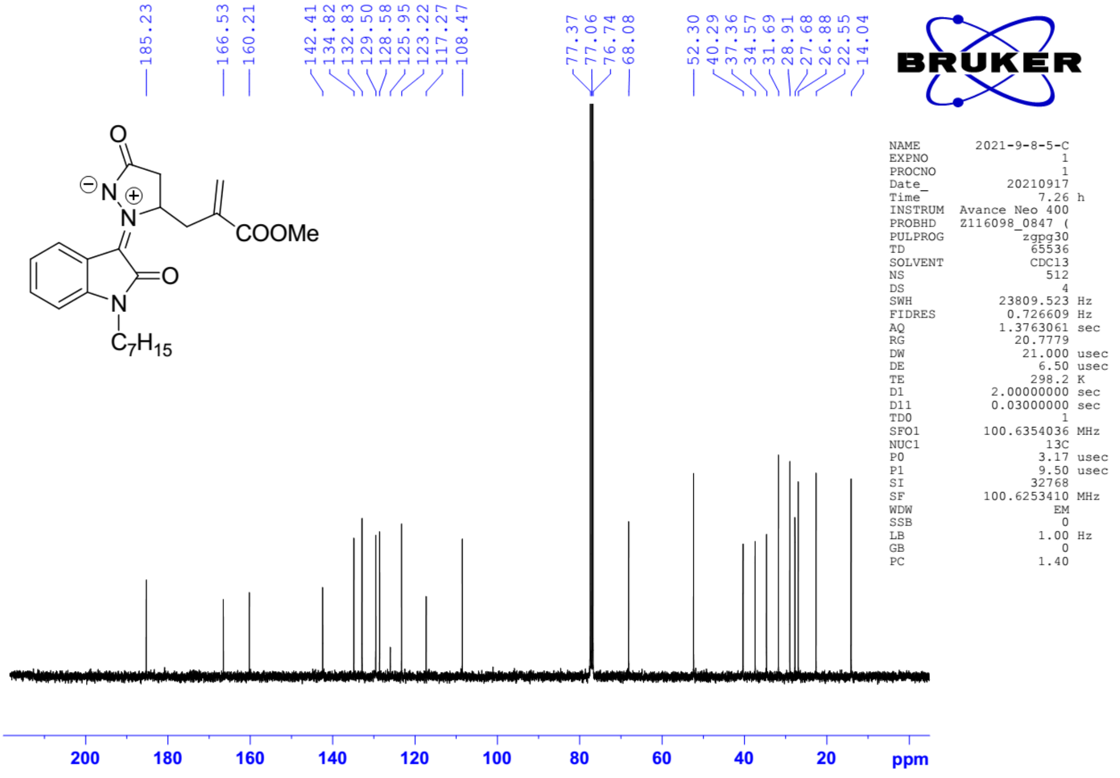

^{1}H and ^{13}C NMR Spectra for Compound **8j**

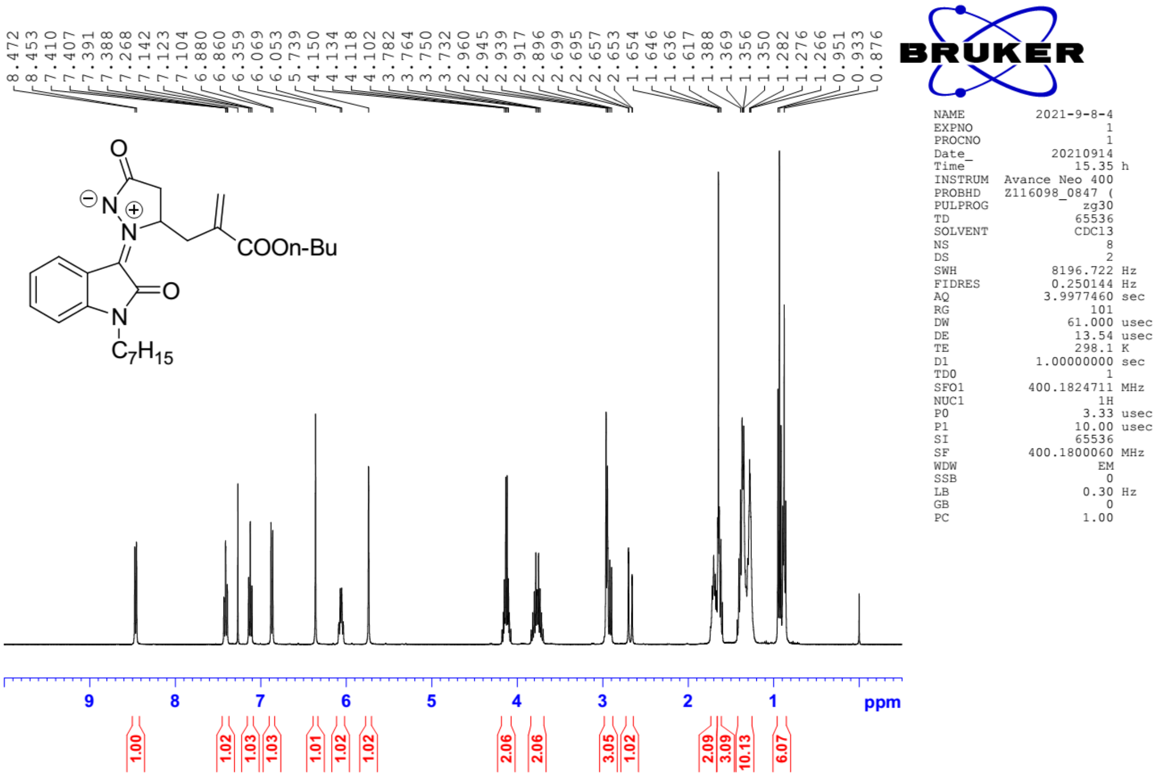

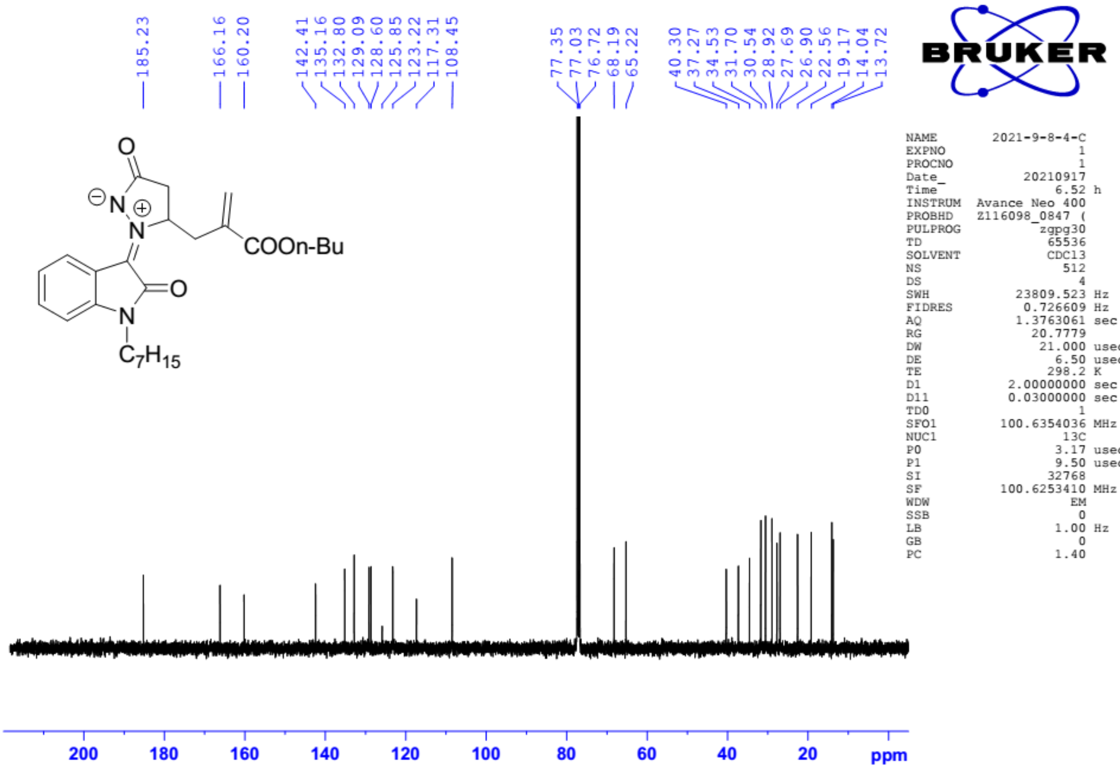

^{1}H and ^{13}C NMR Spectra for Compound **8k**

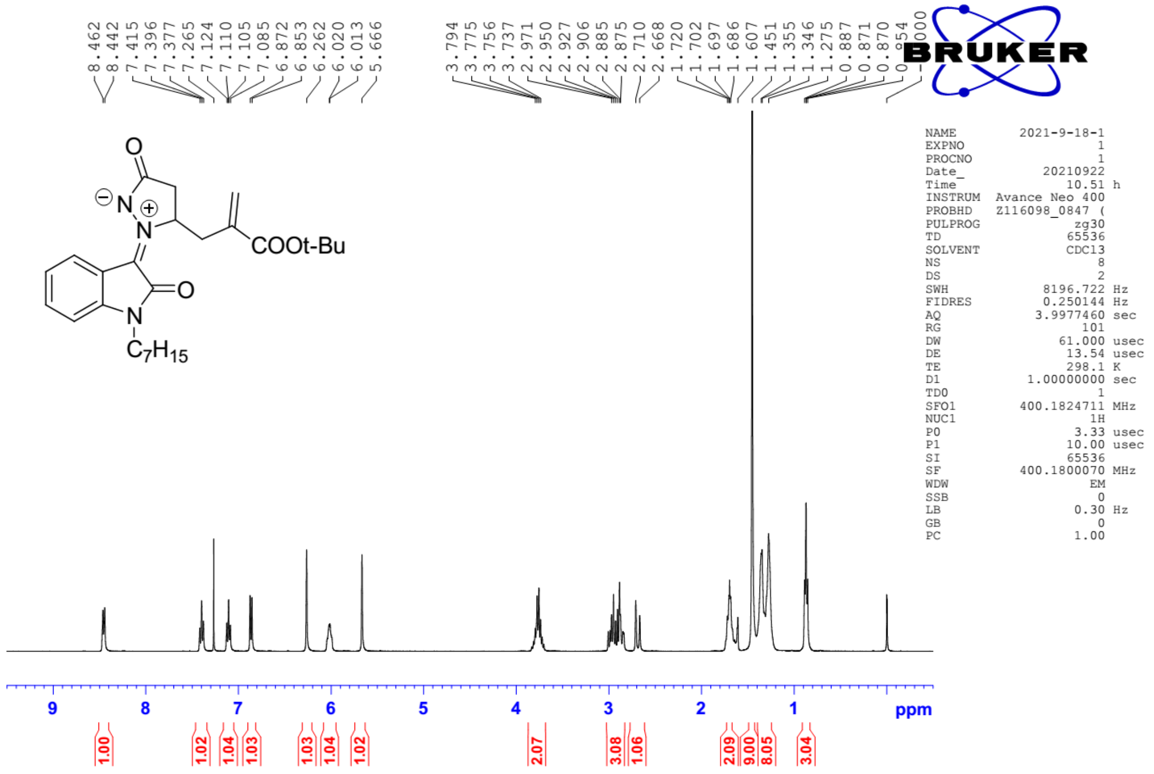

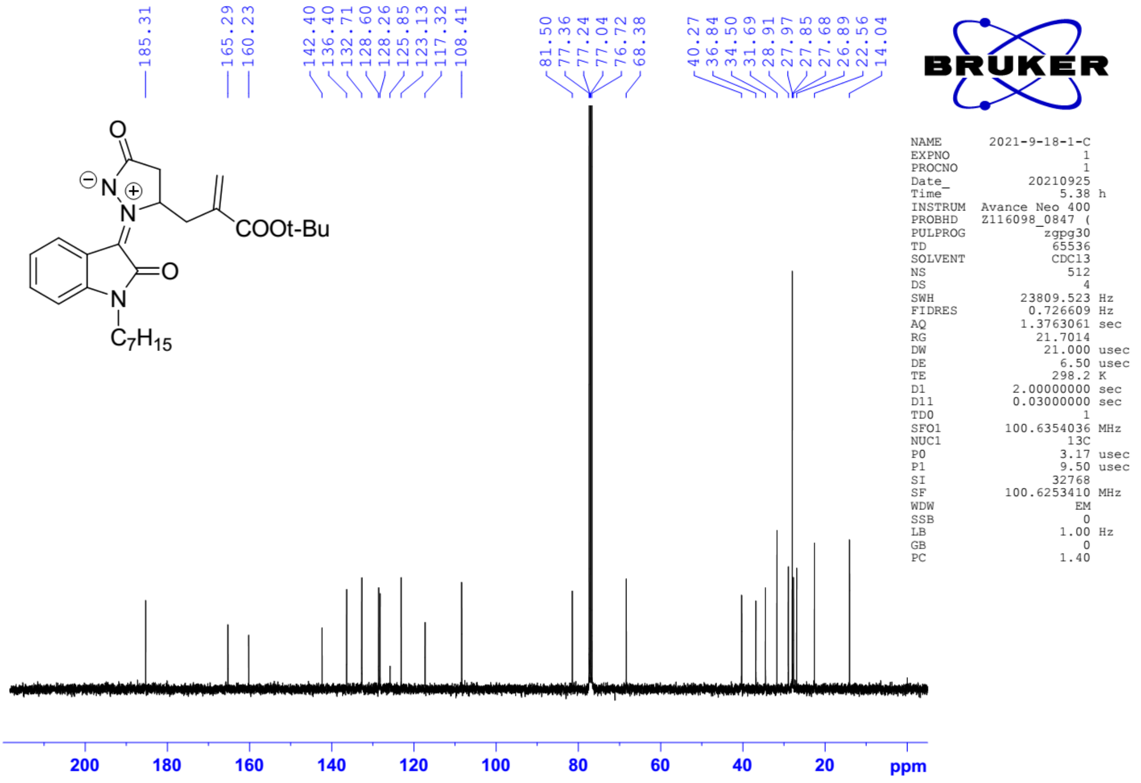

^{1}H and ^{13}C NMR Spectra for Compound **4a**

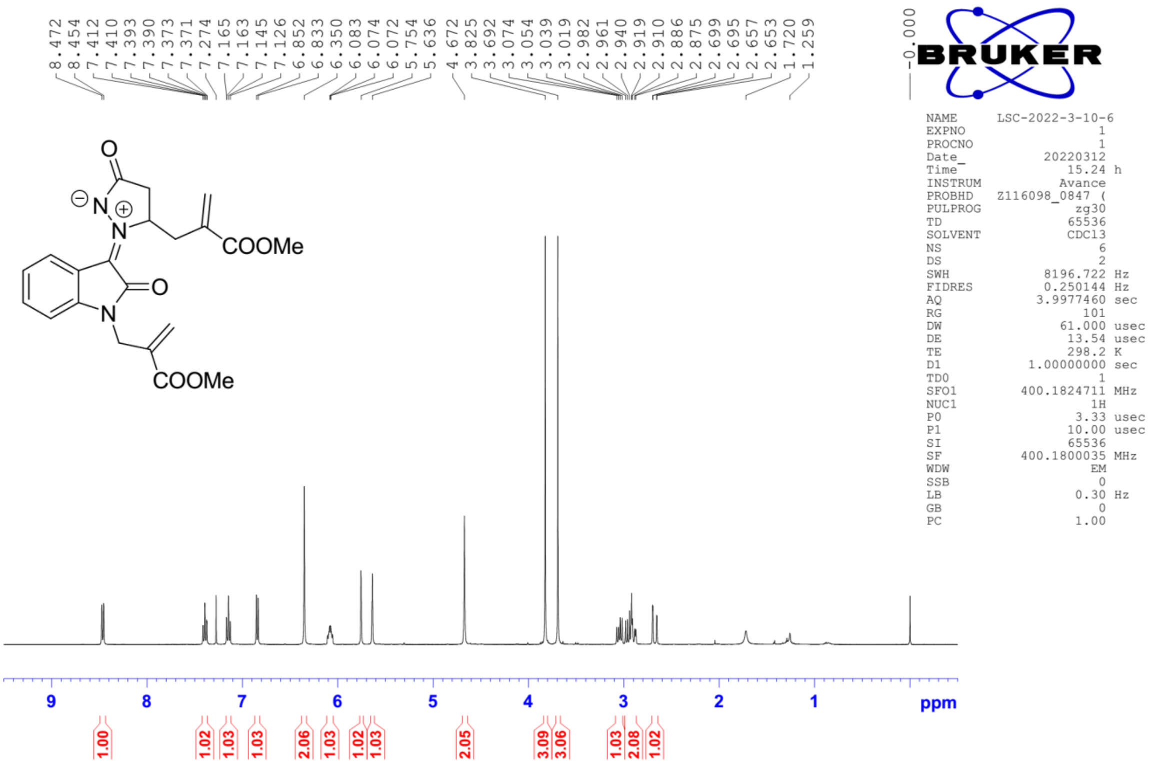

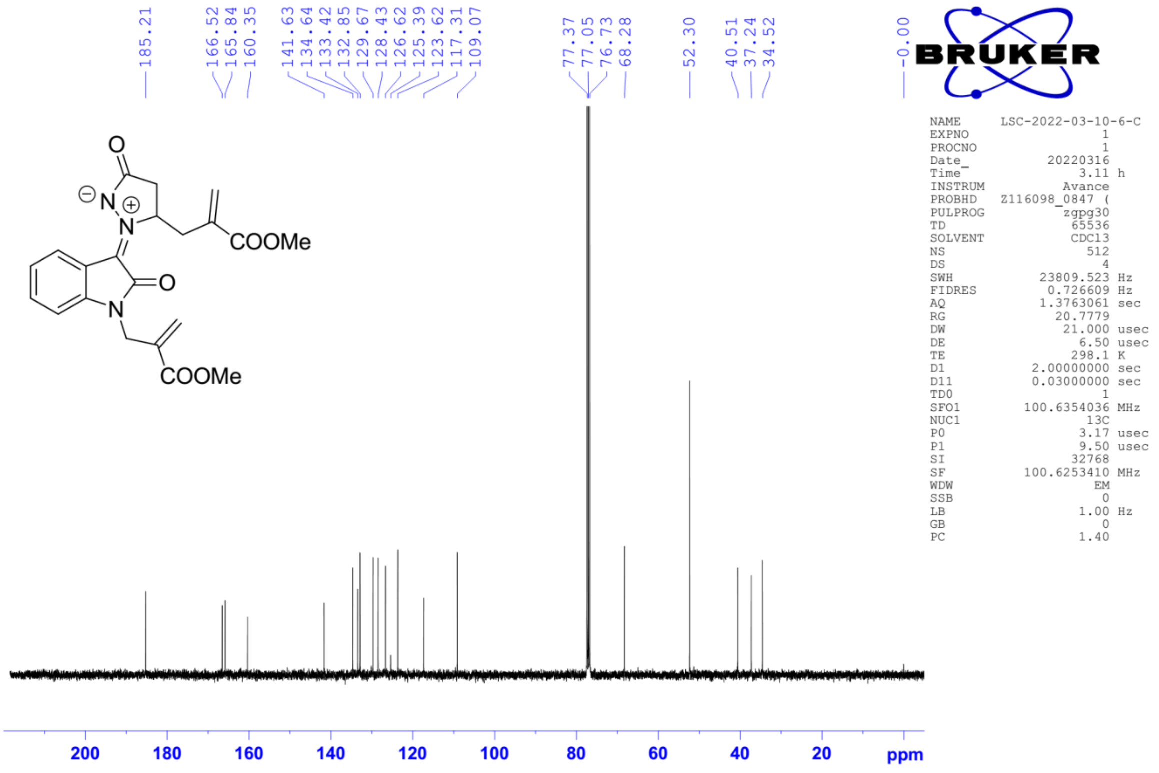

^{1}H and ^{13}C NMR Spectra for Compound **4b**

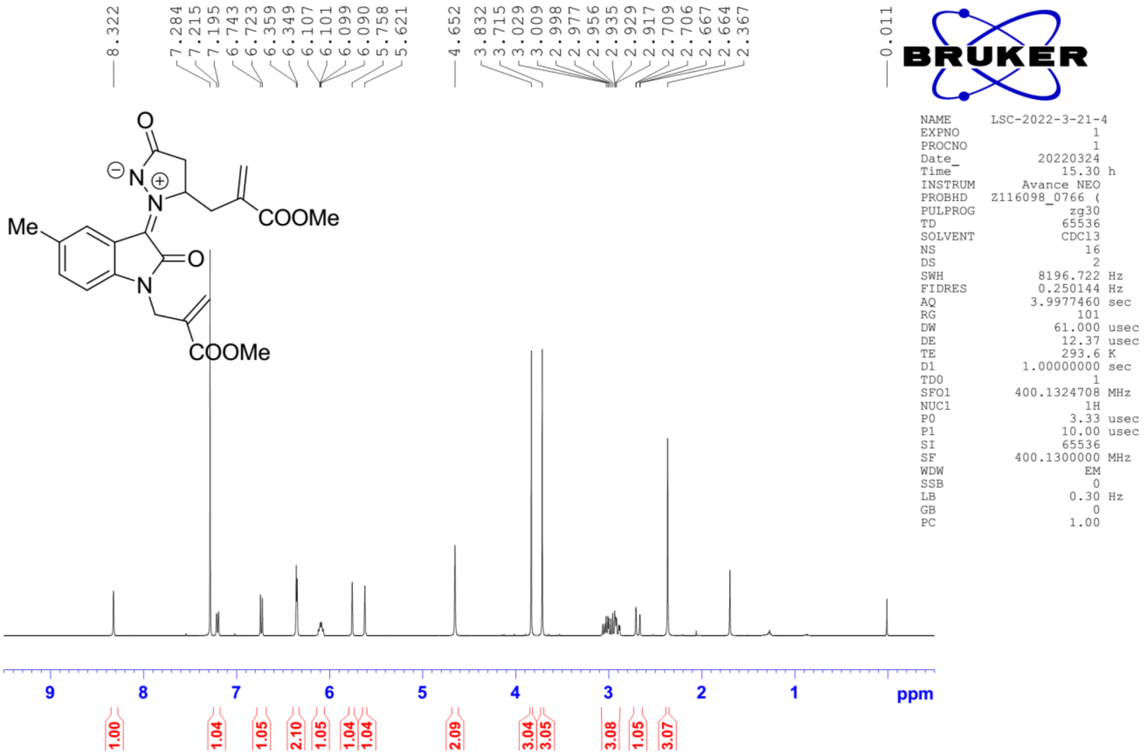

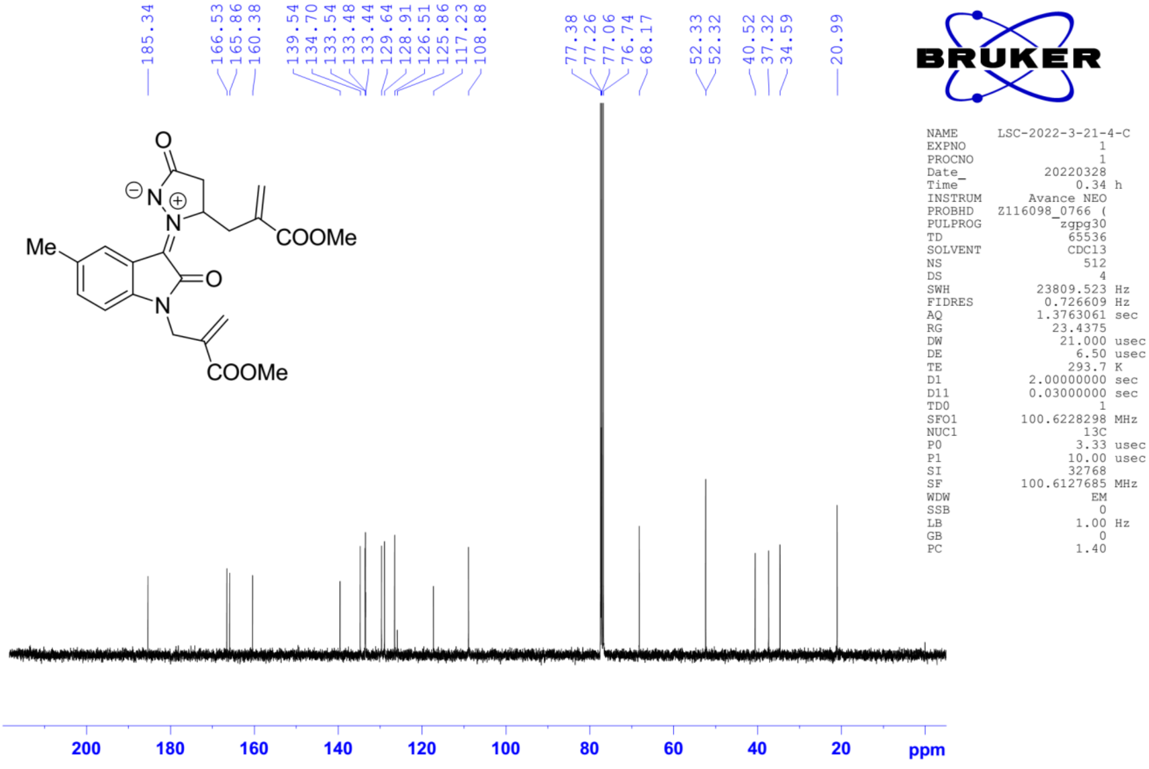

^{1}H and ^{13}C NMR Spectra for Compound **4c**

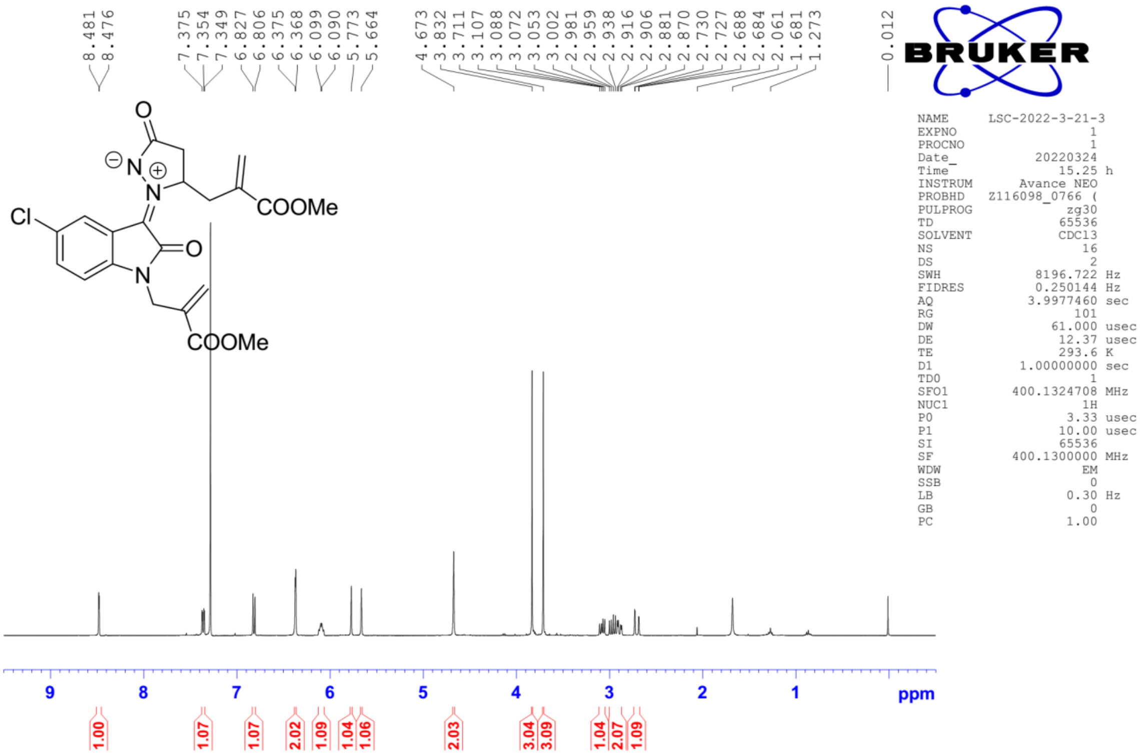

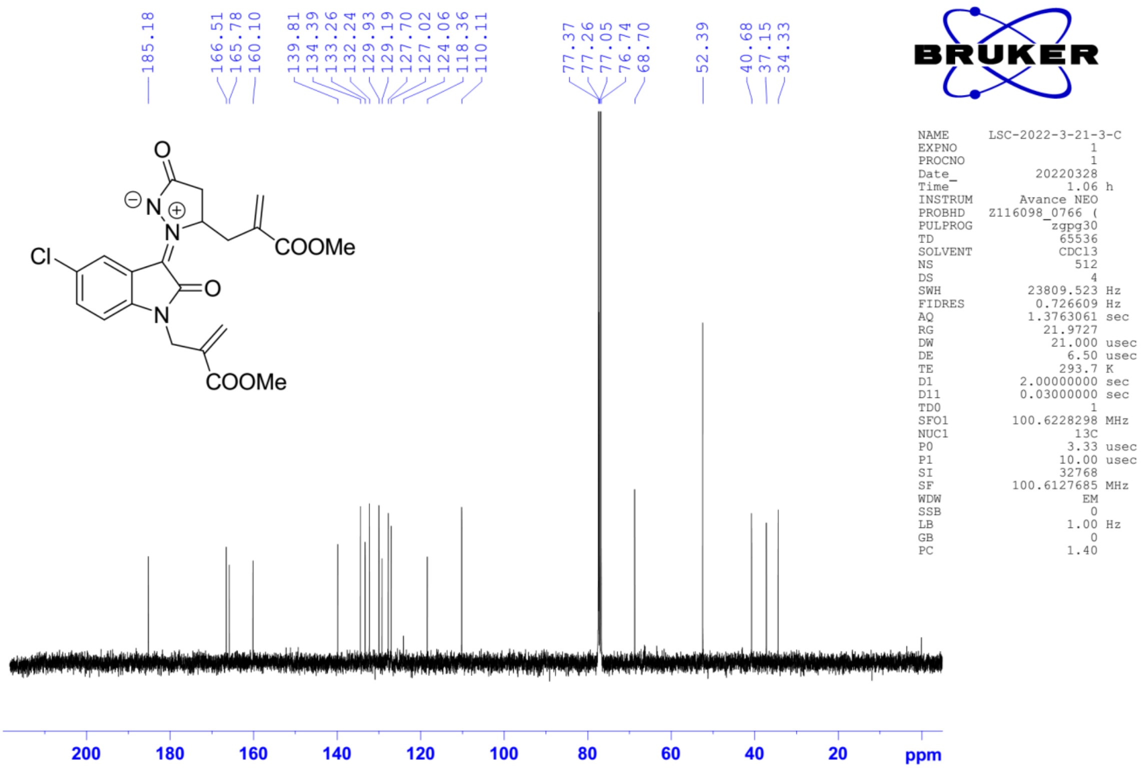

^{1}H and ^{13}C NMR Spectra for Compound **4d**

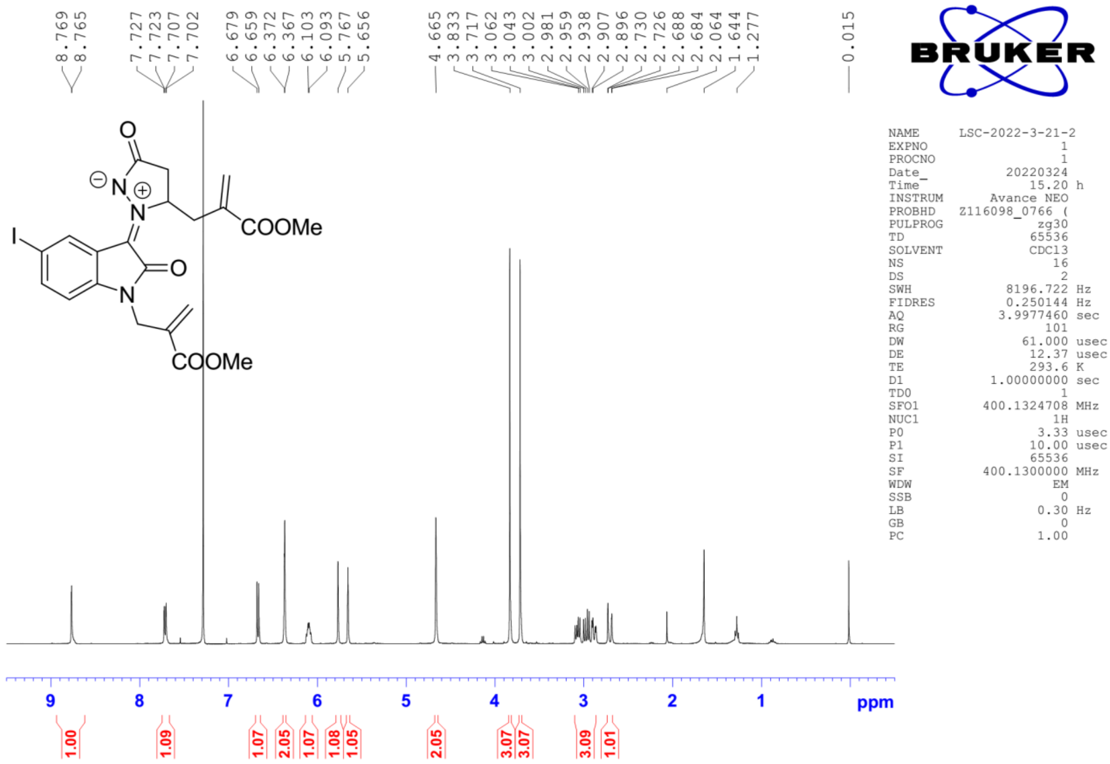

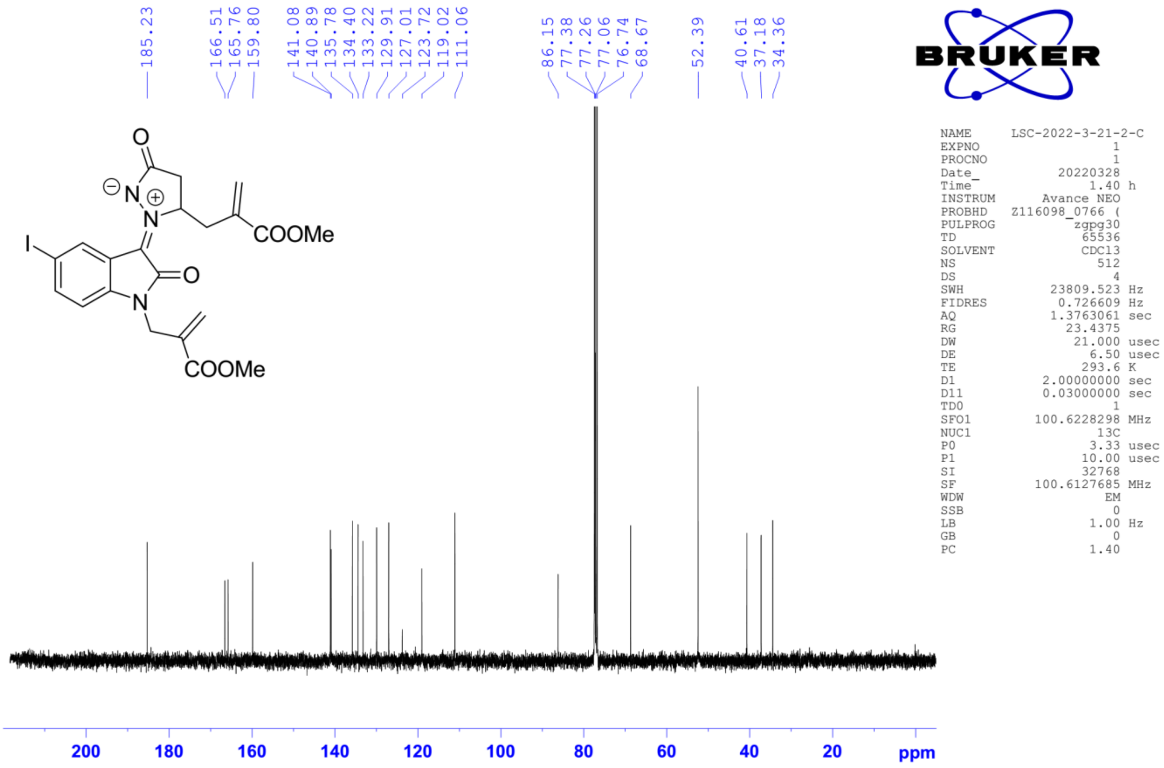

^{1}H and ^{13}C NMR Spectra for Compound **4e**

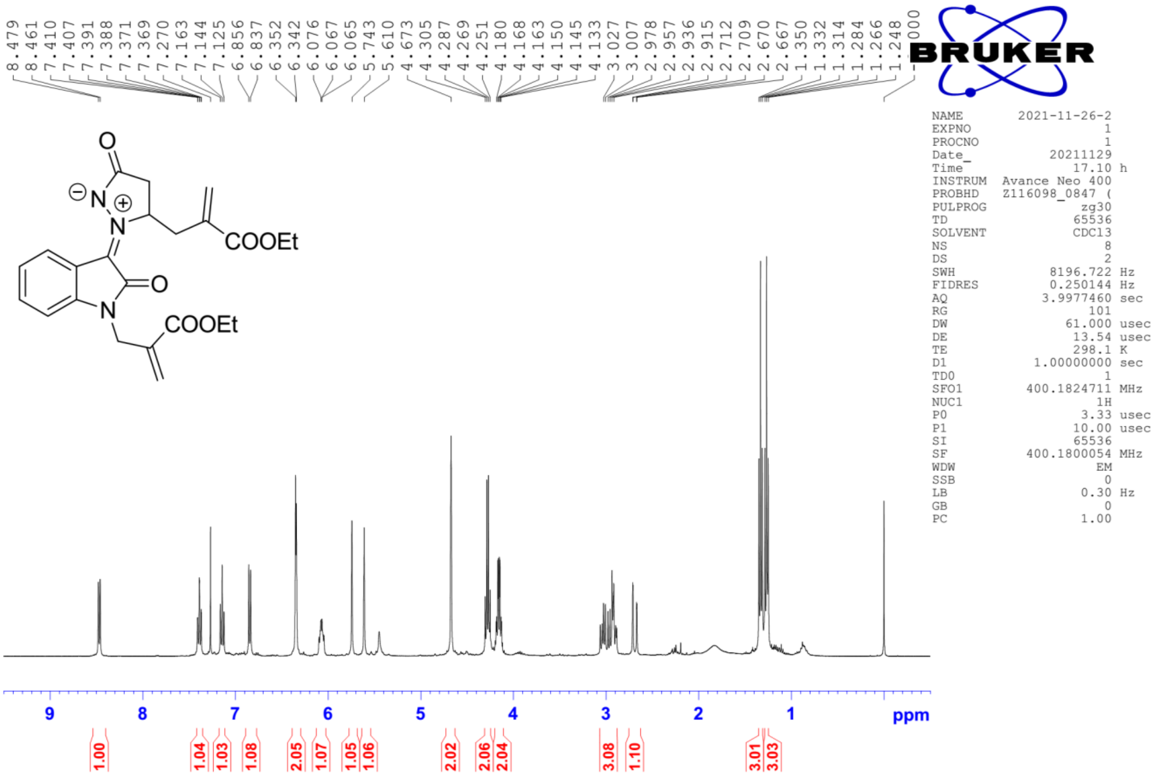

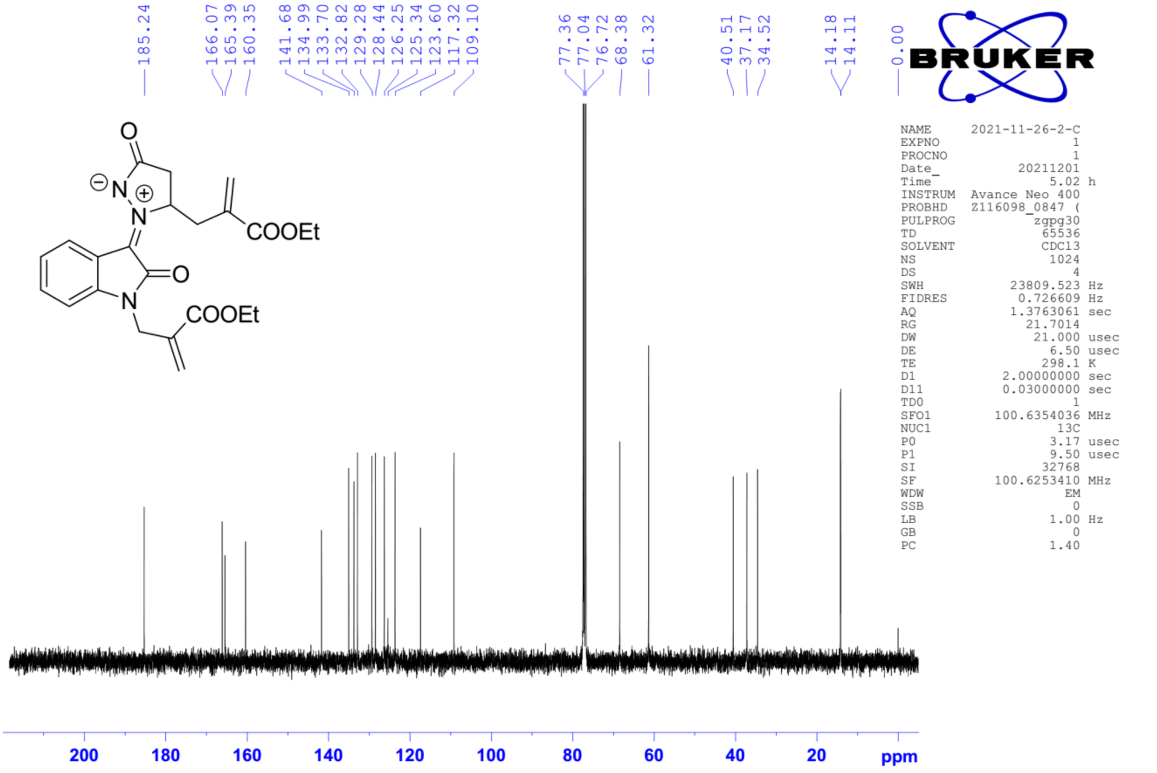

^{1}H and ^{13}C NMR Spectra for Compound **4f**

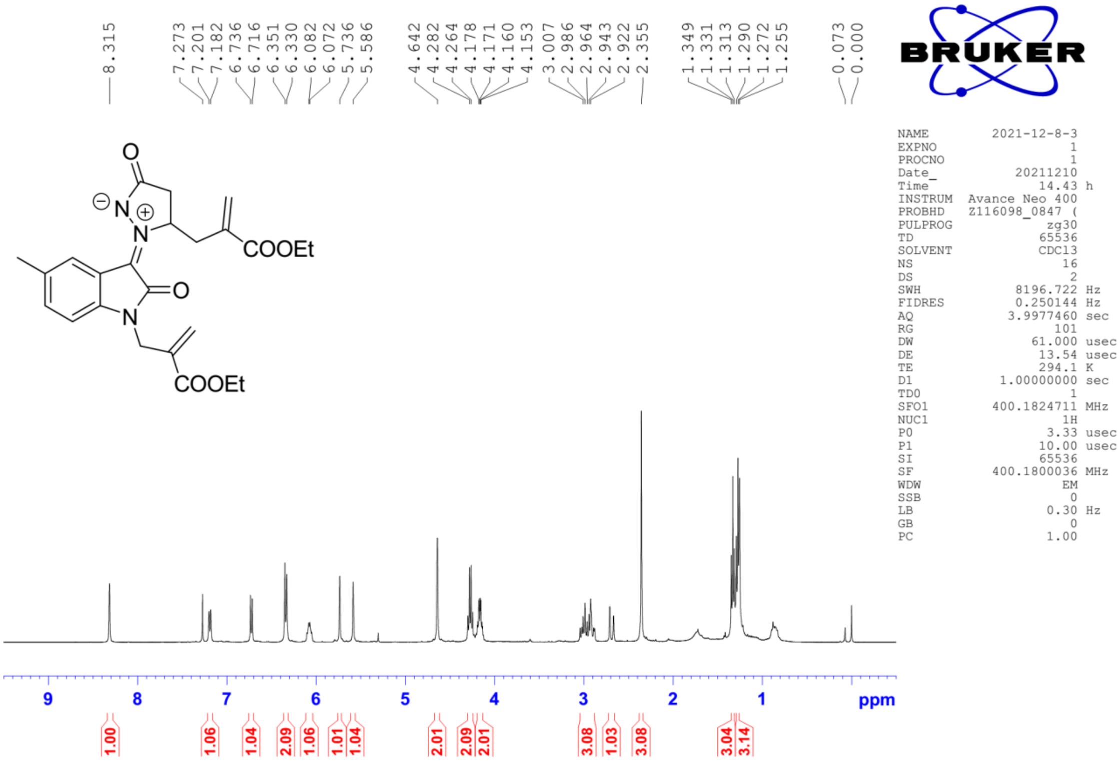

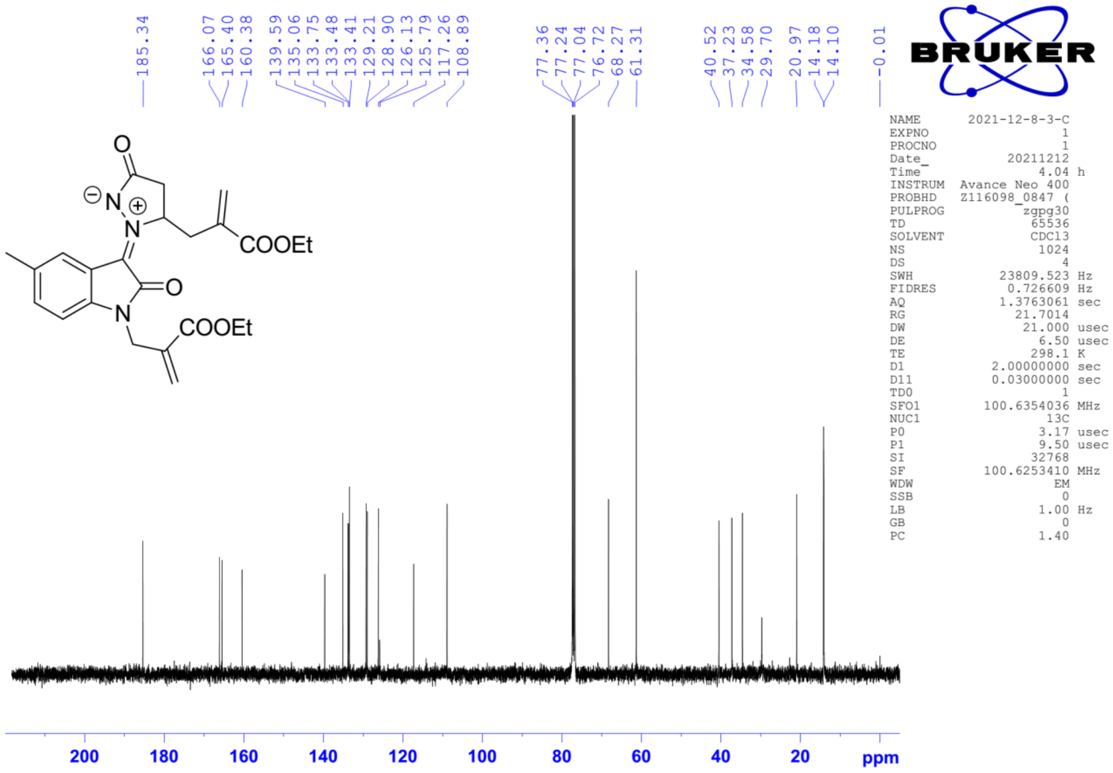

^{1}H, ^{13}C and ^{19}F NMR Spectra for Compound **4g**

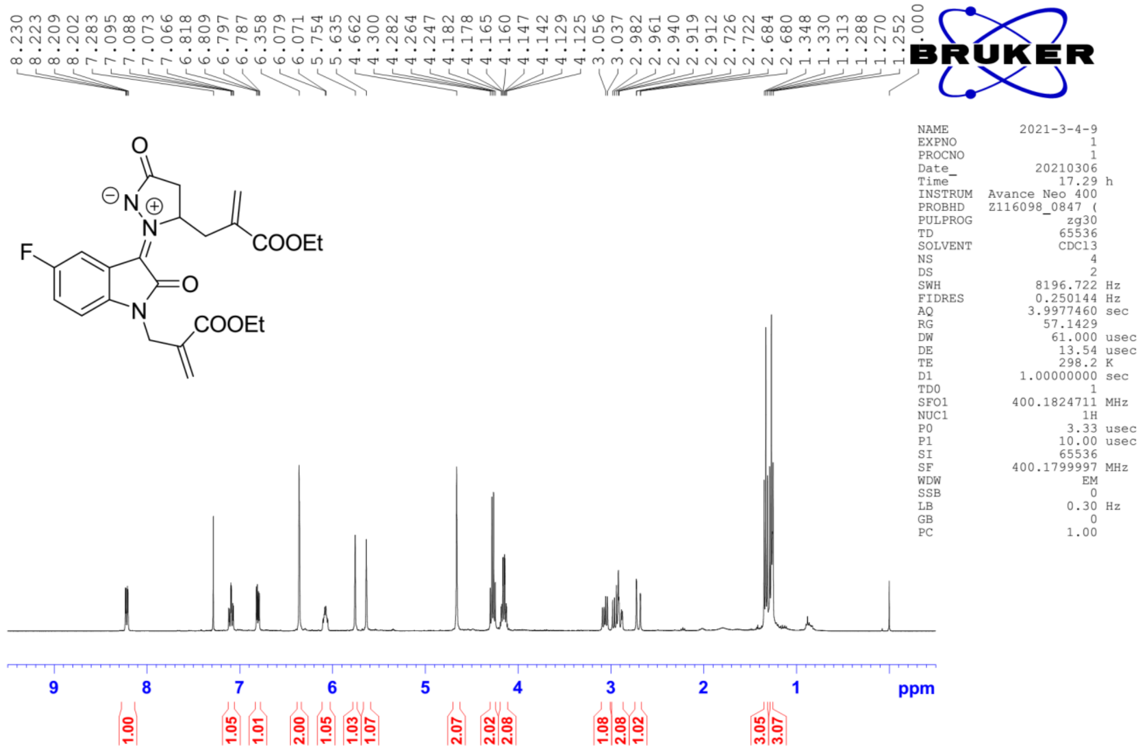

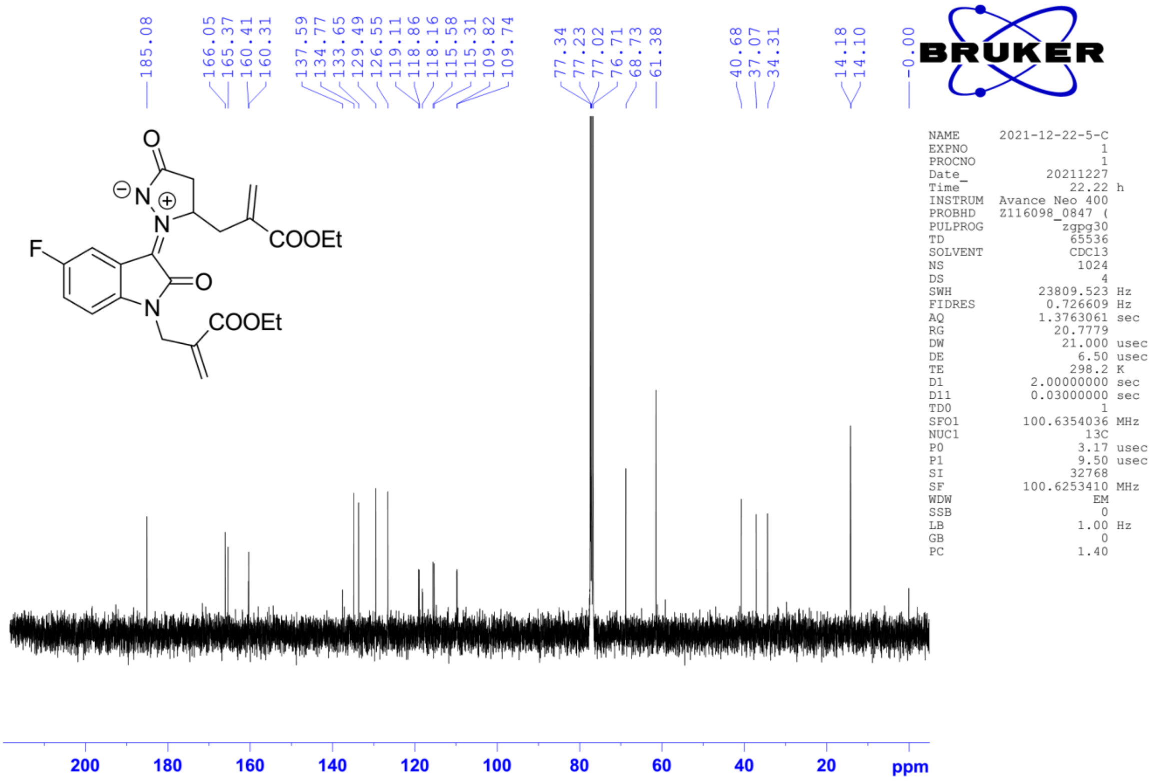

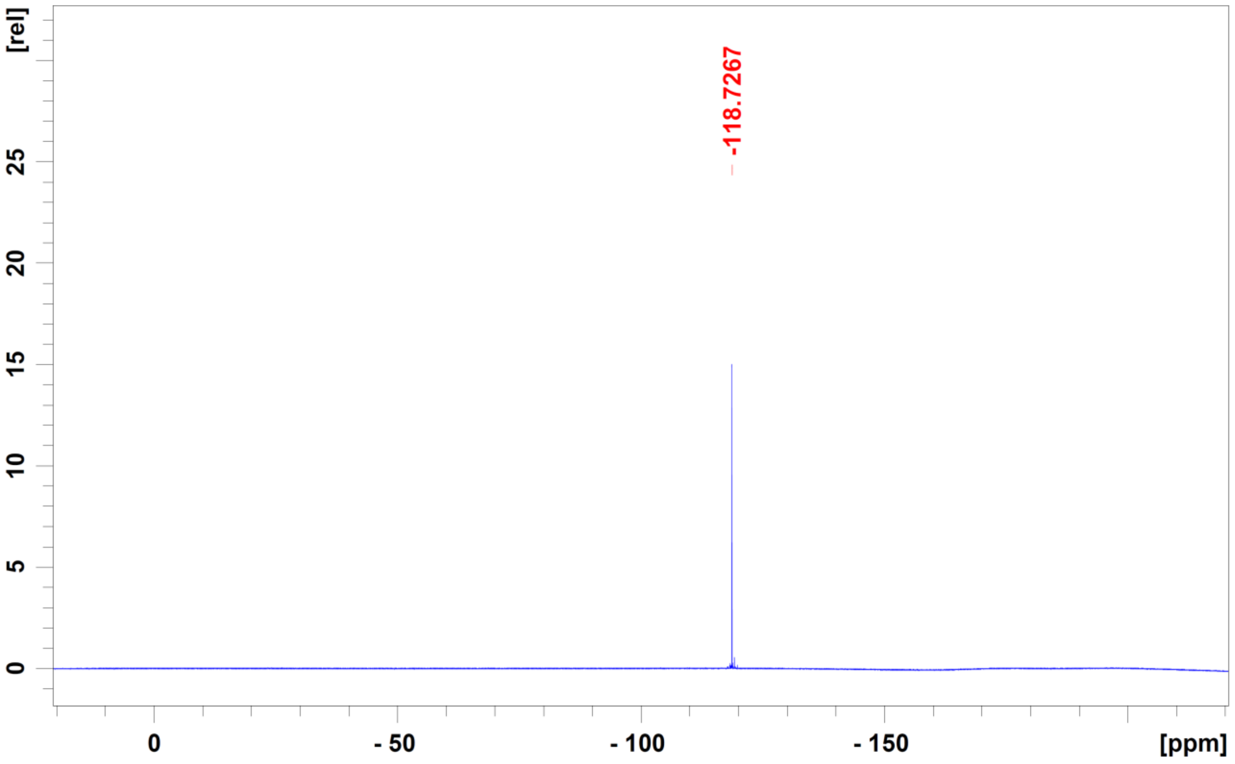

^{1}H and ^{13}C NMR Spectra for Compound **4h**

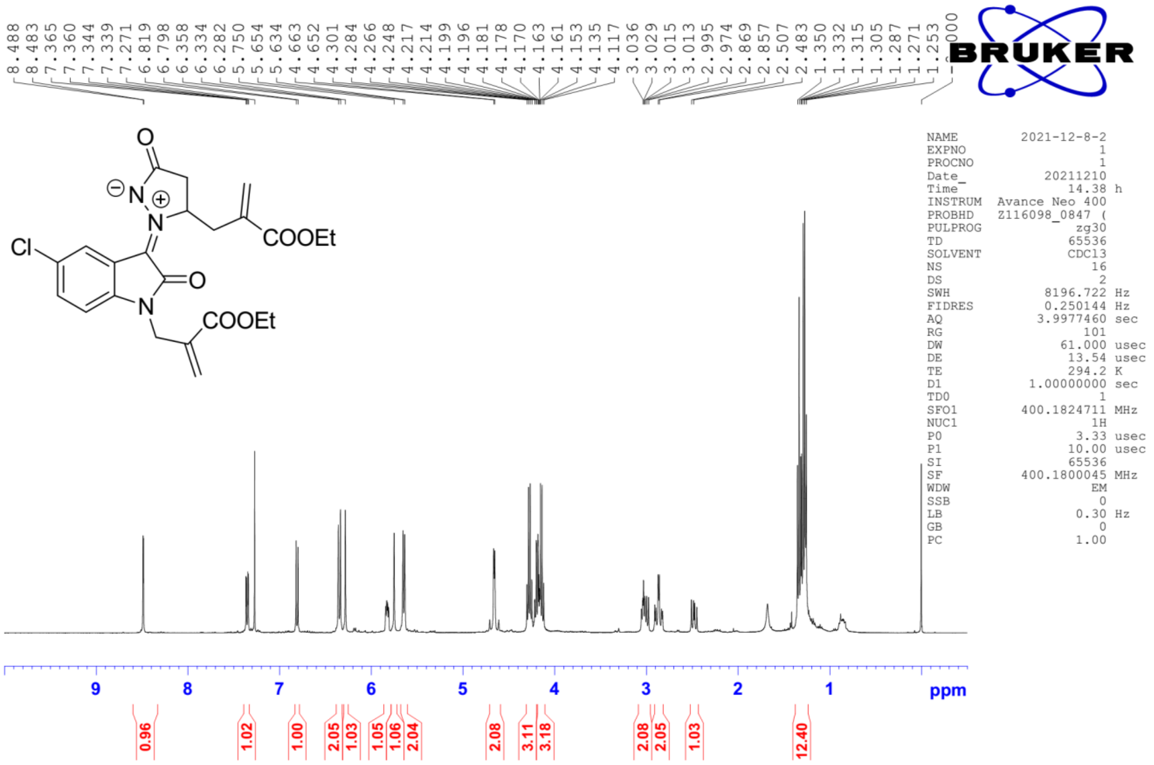

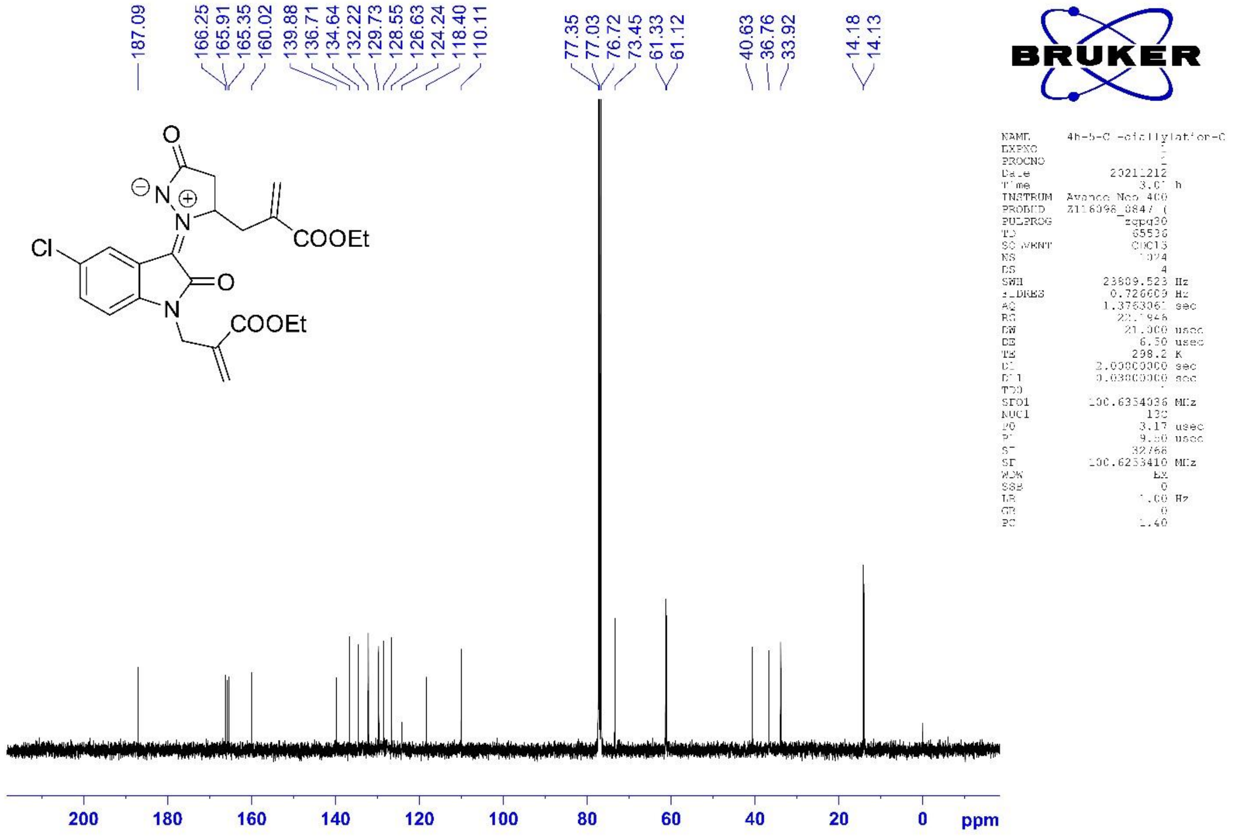

^{1}H NMR and ^{13}C NMR Spectra for Compound **4i**

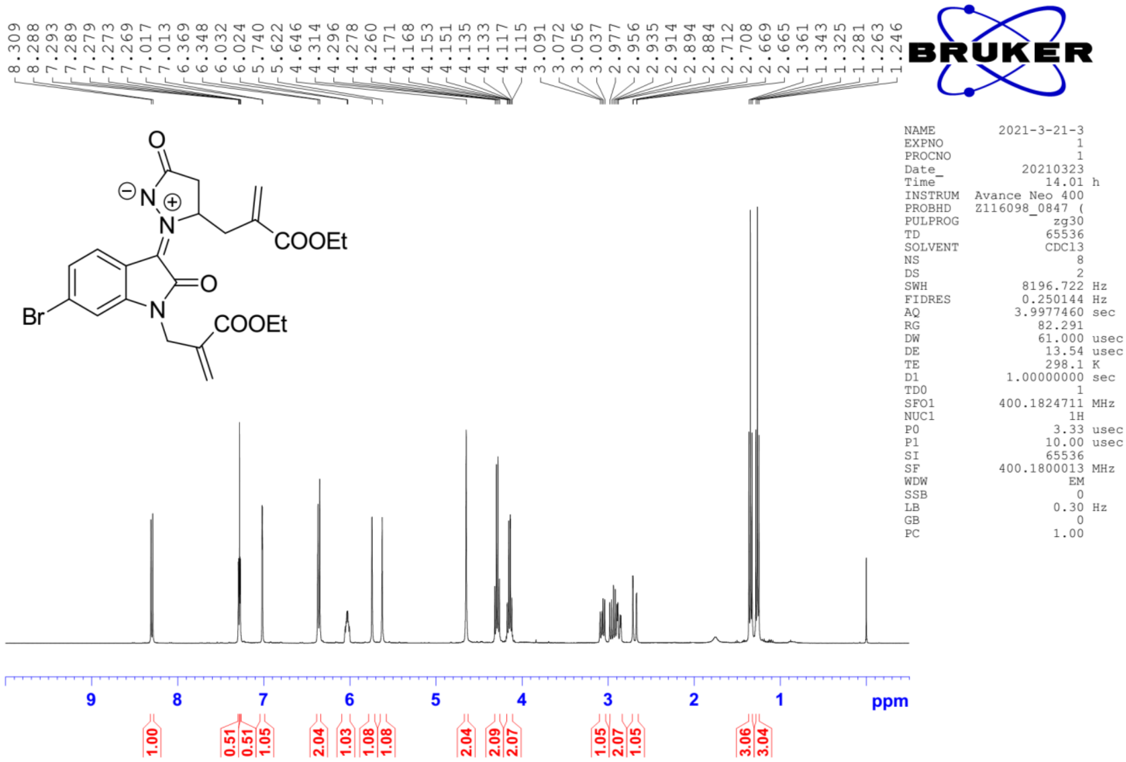

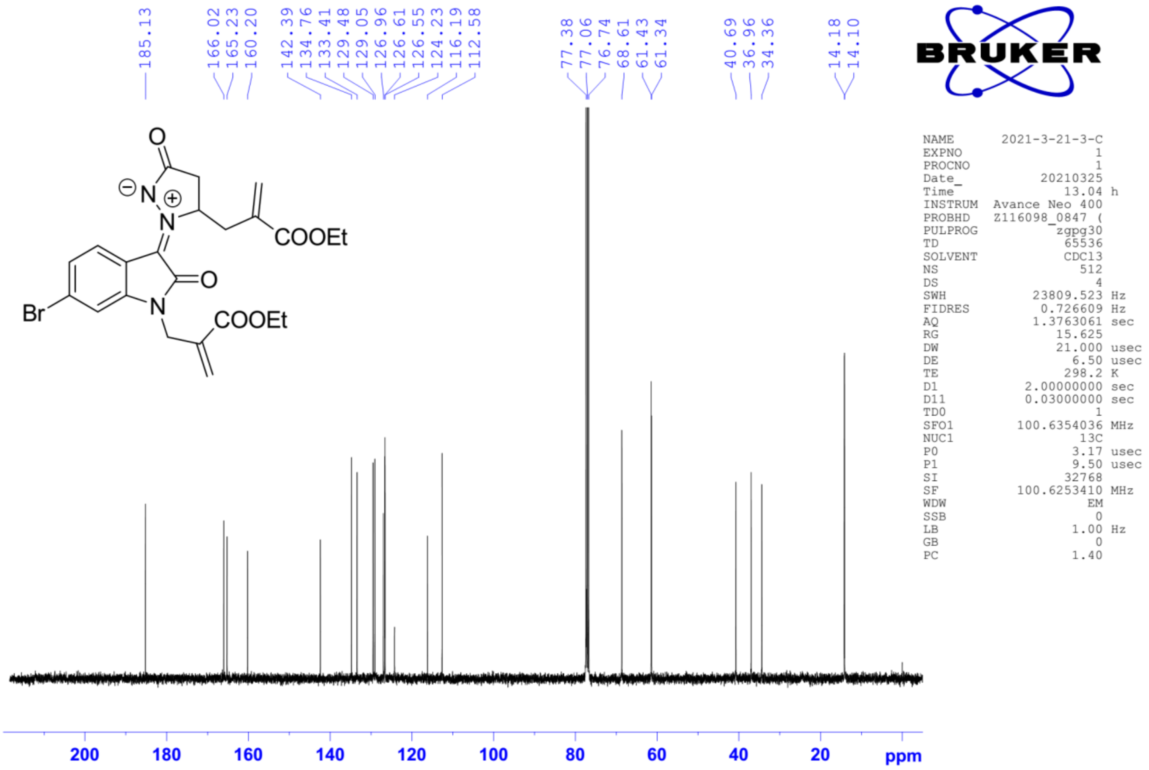

^{1}H and ^{13}C NMR Spectra for Compound **4j**

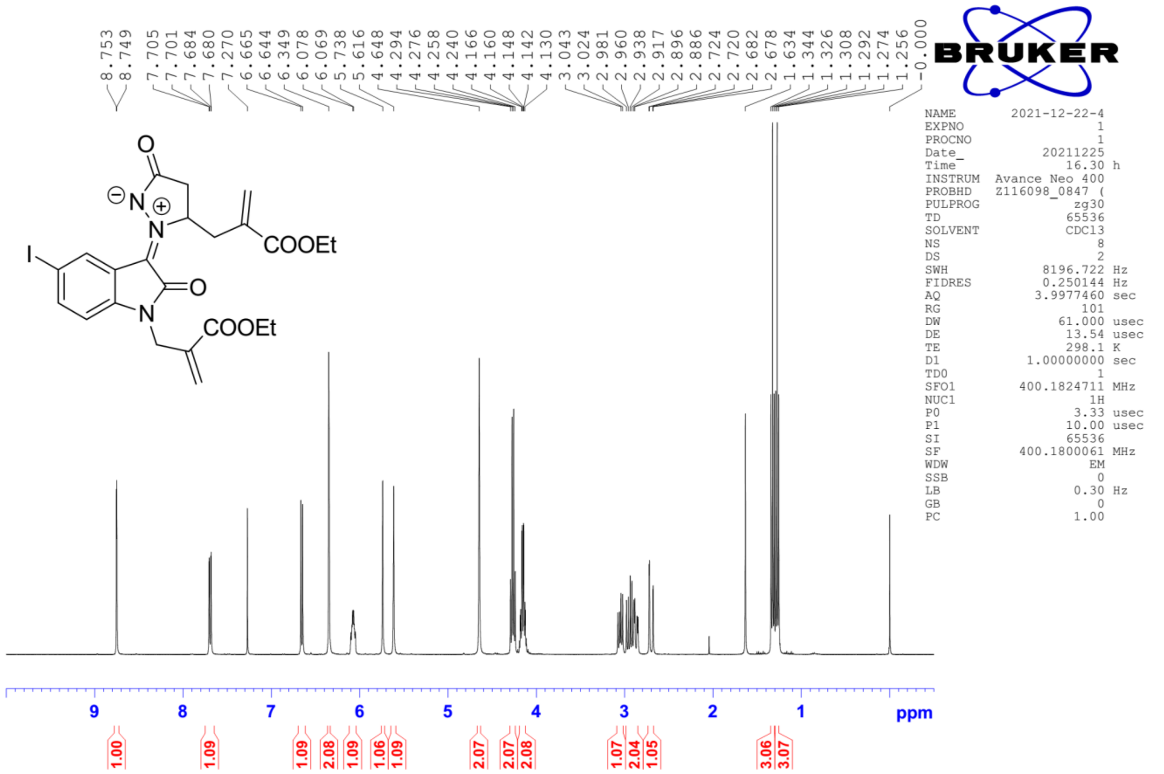

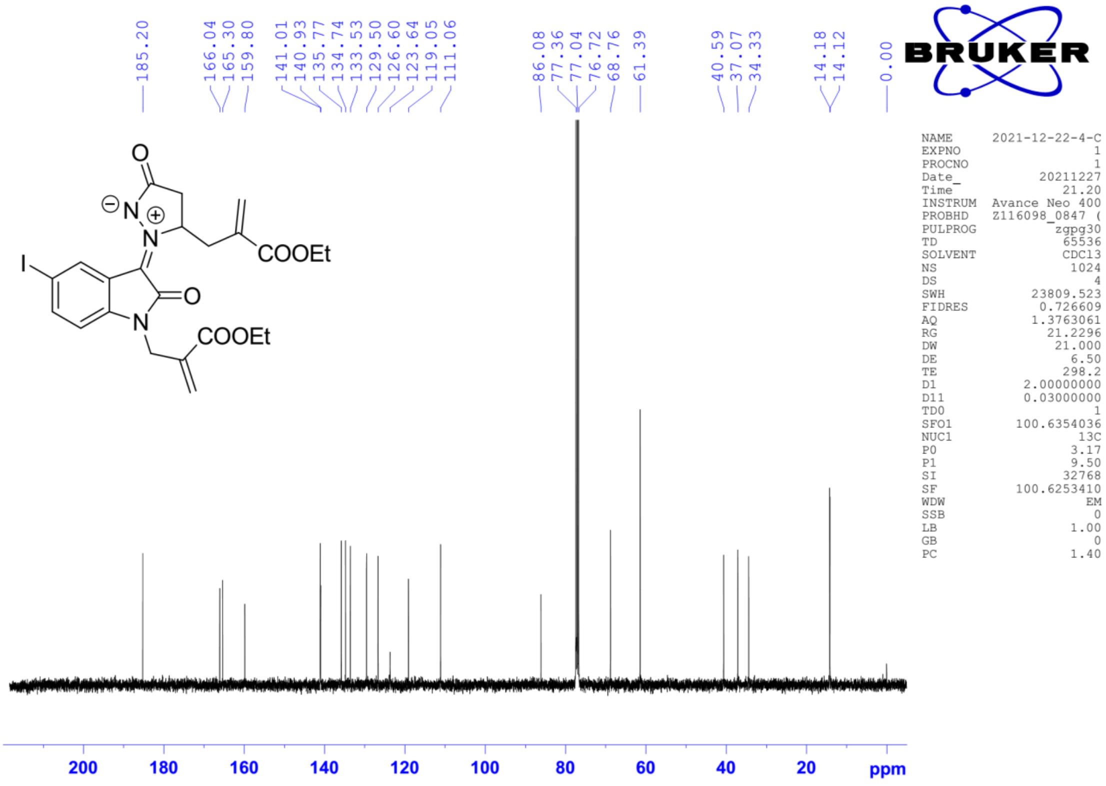

^{1}H and ^{13}C NMR Spectra for Compound **10**

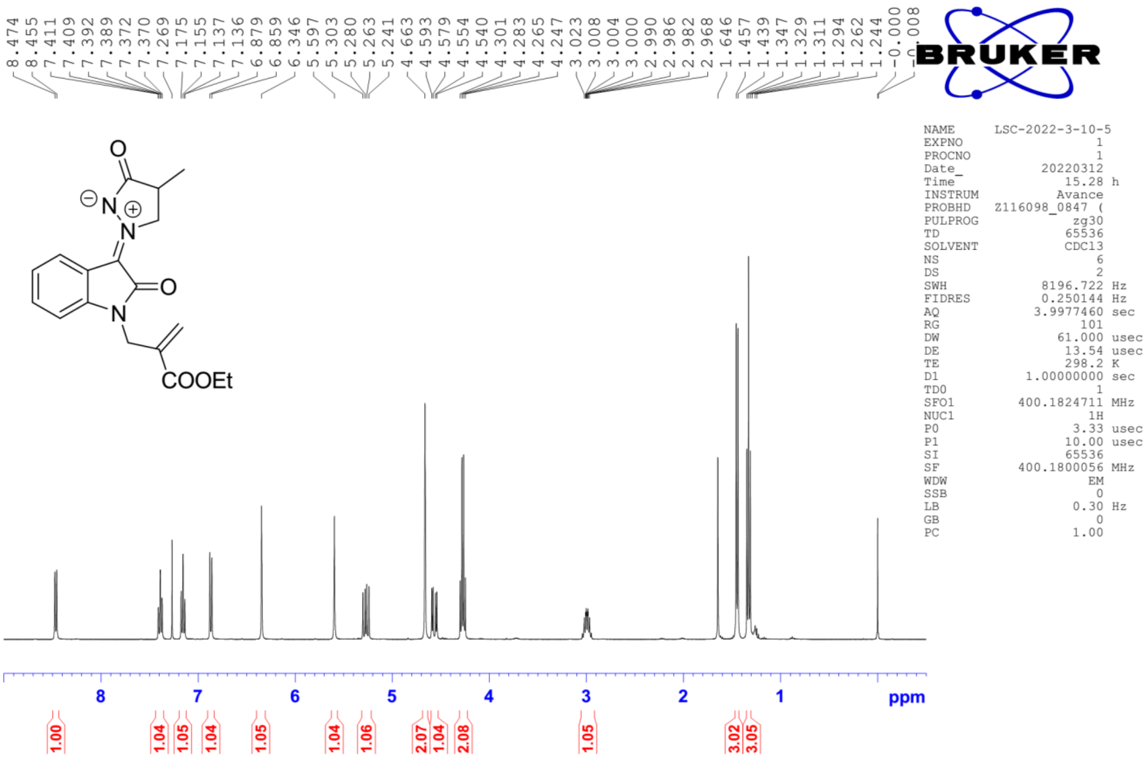

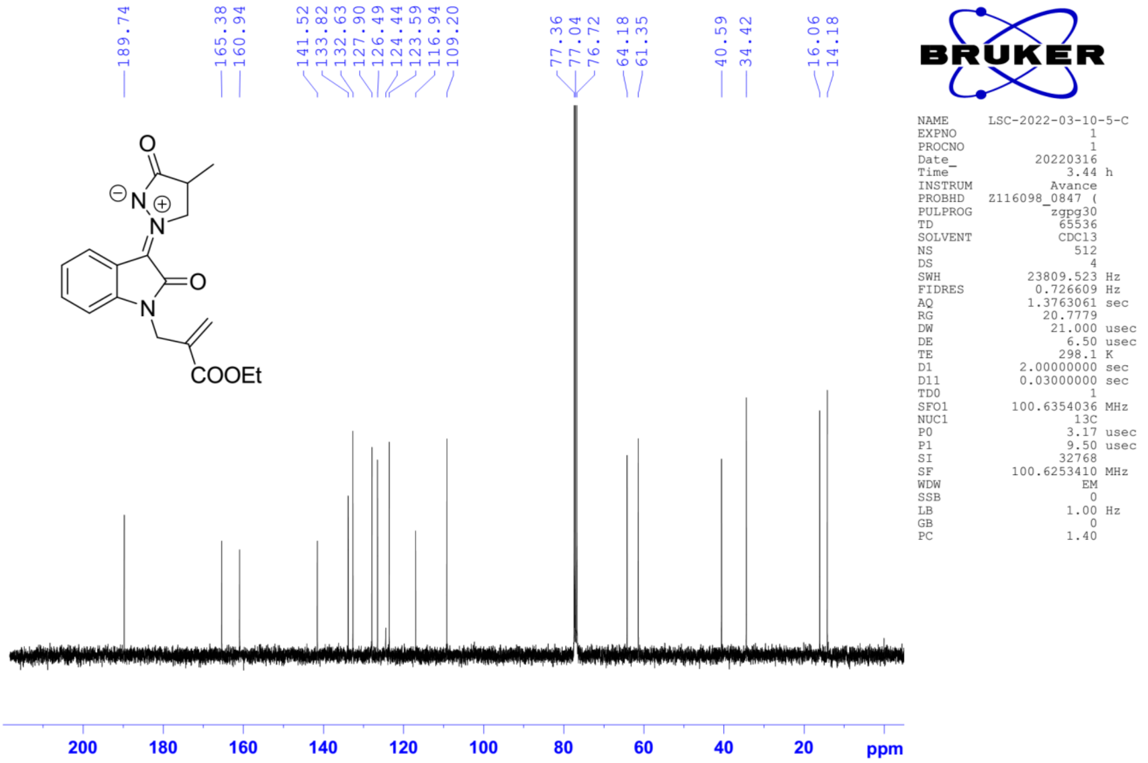

^{1}H NMR and ^{13}C NMR Spectra for Compound **12**

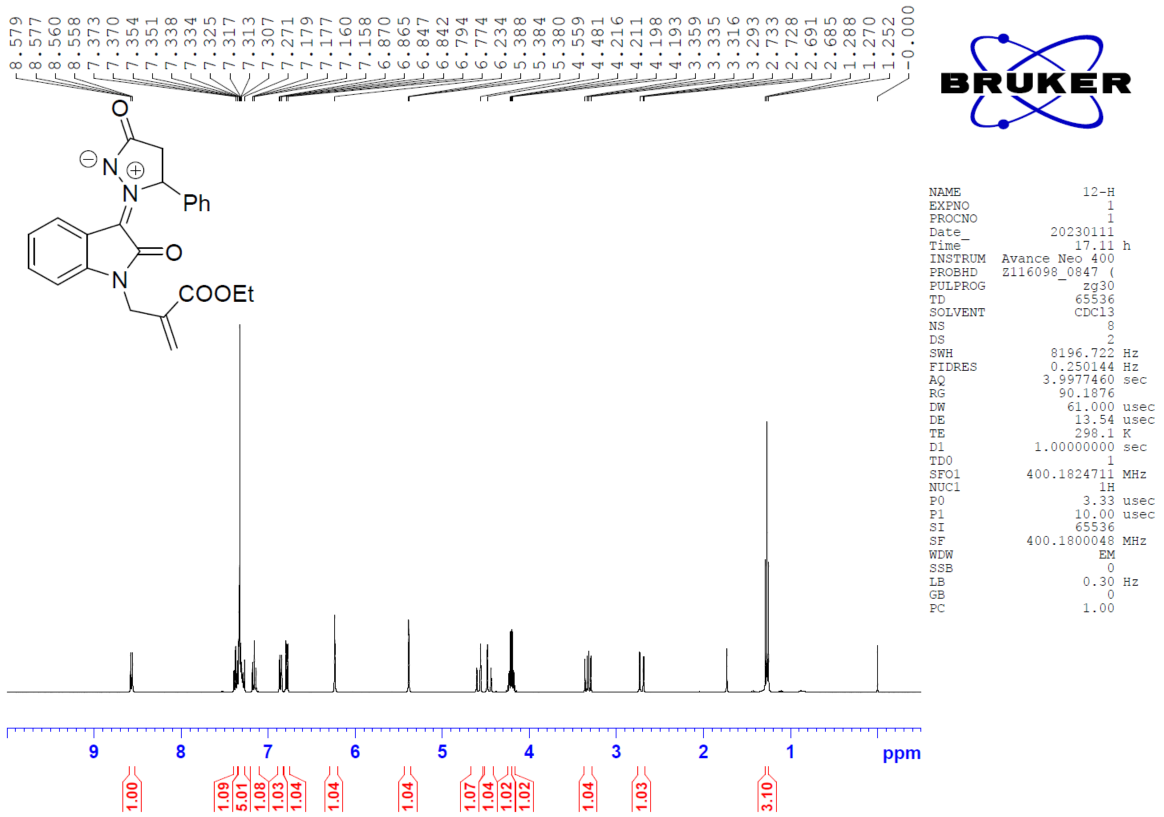

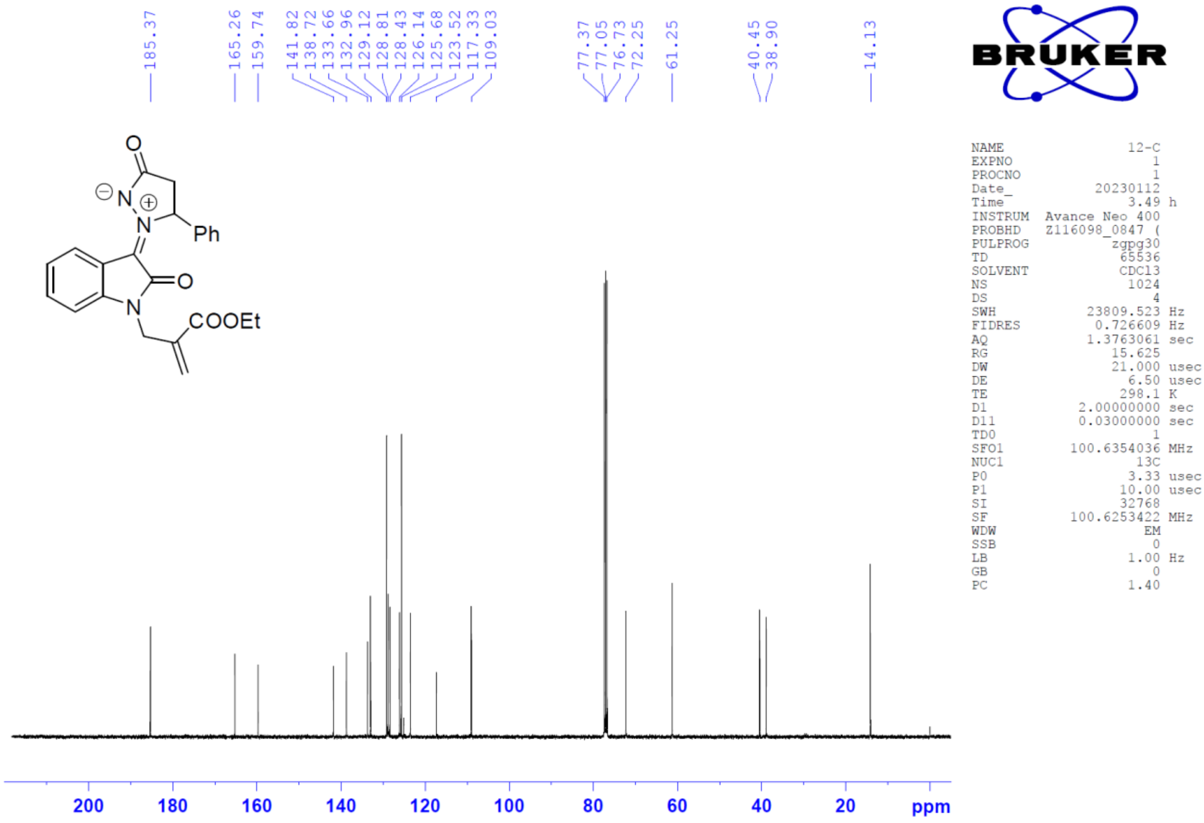

^{1}H and ^{13}C NMR Spectra for Compound **13**

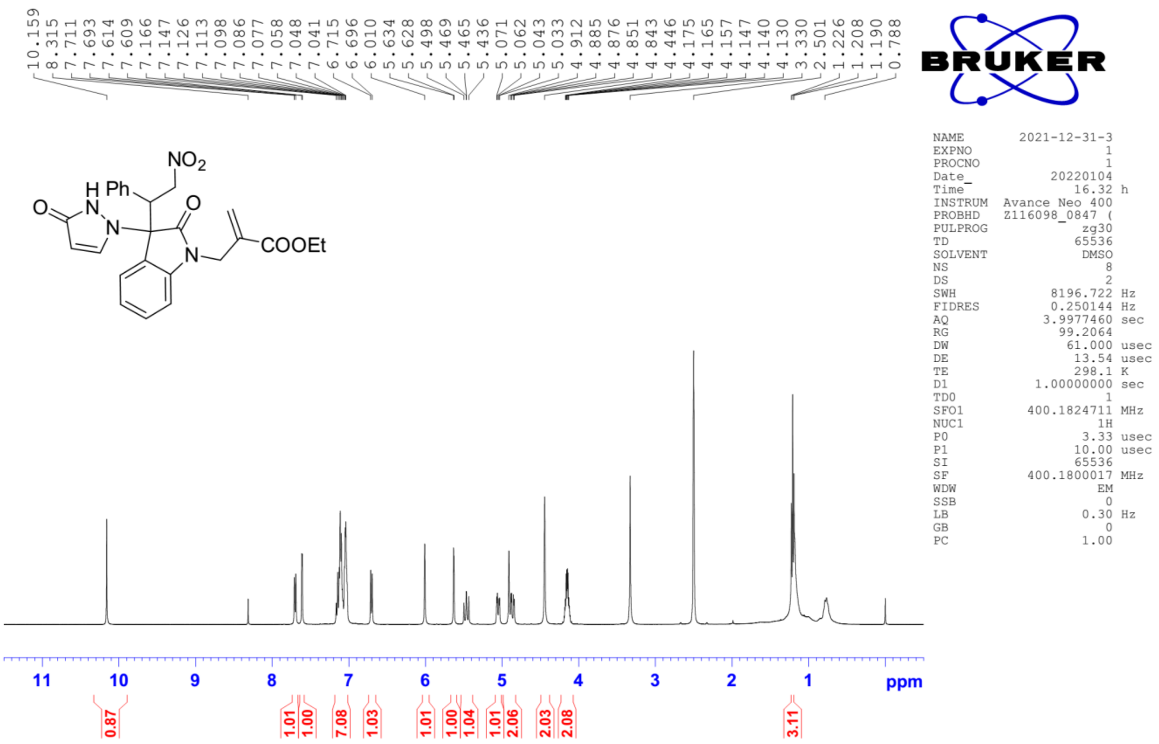

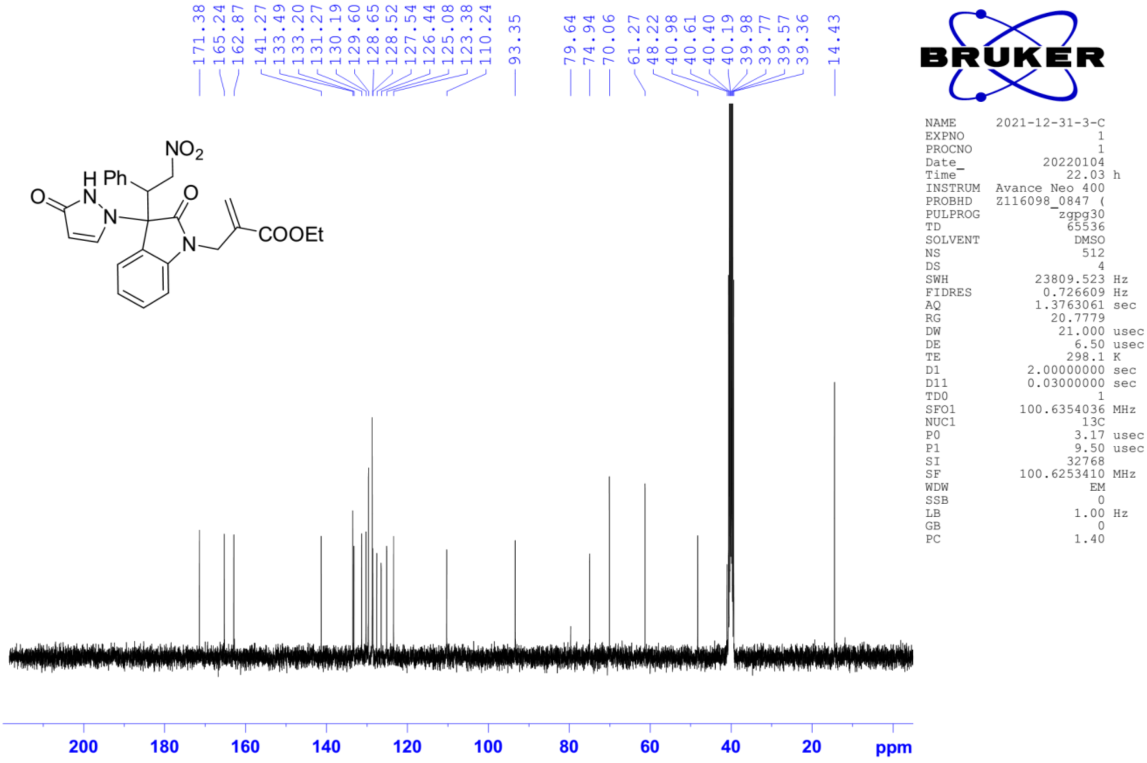

^{1}H and ^{13}C NMR Spectra for Compound **14**

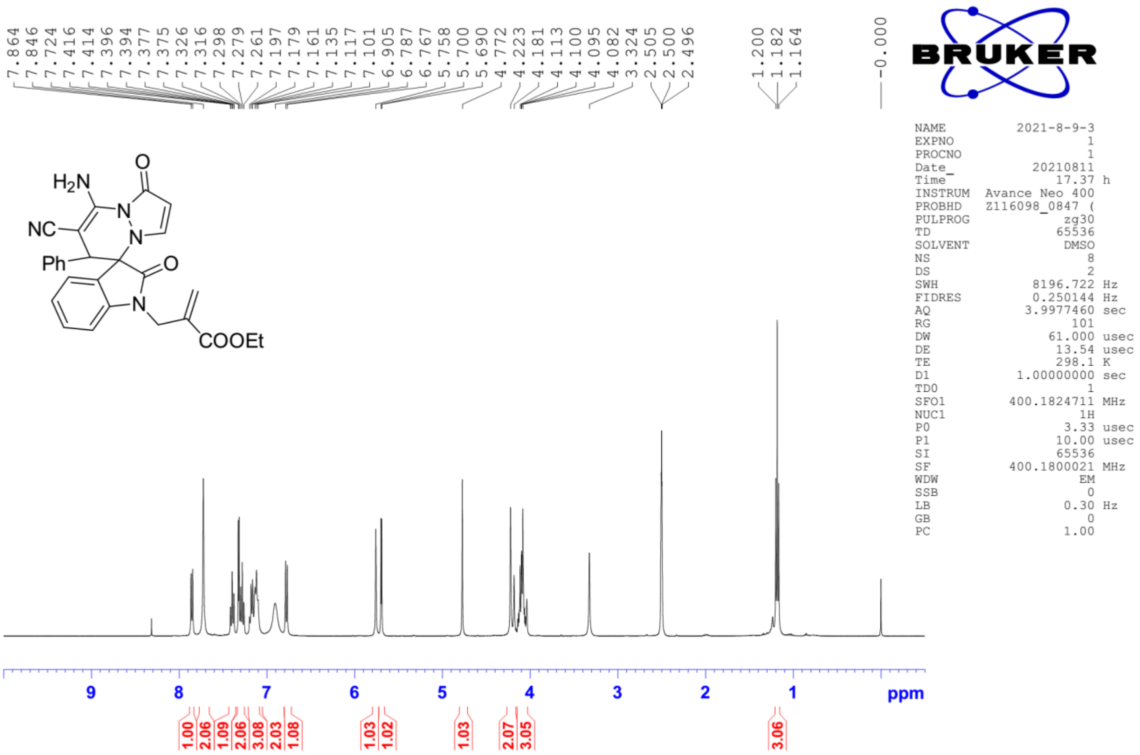

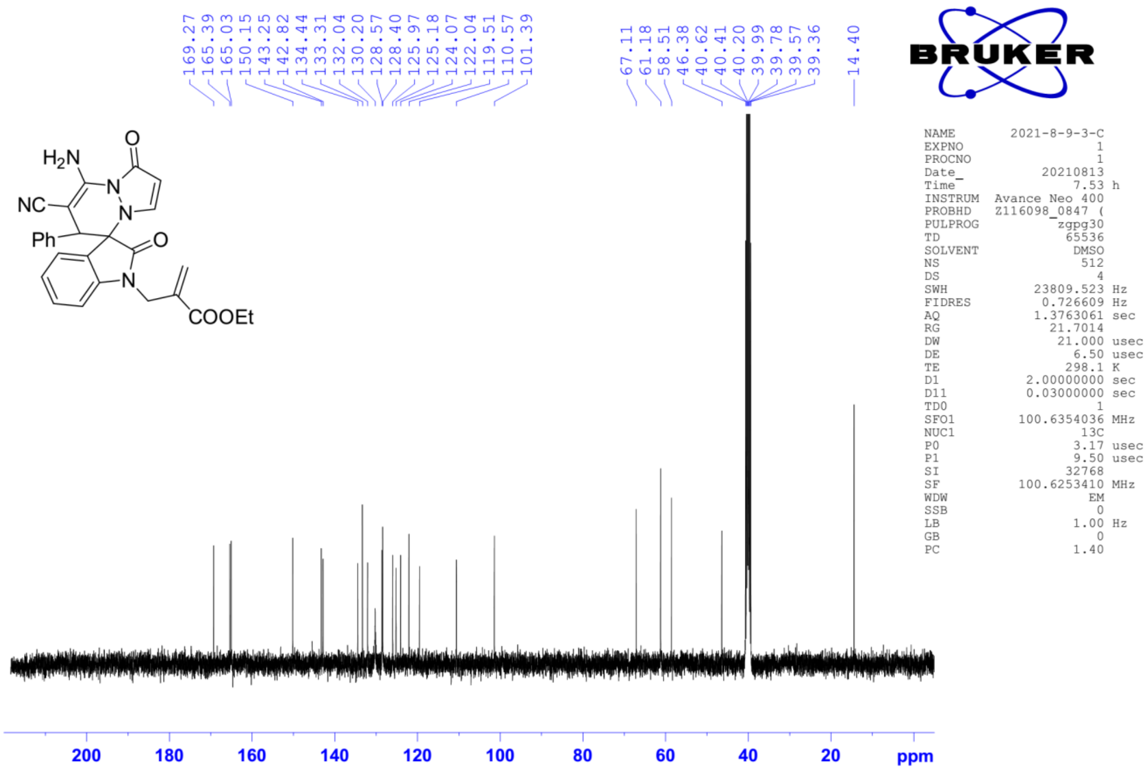

^{1}H and ^{13}C NMR Spectra for Compound **15**

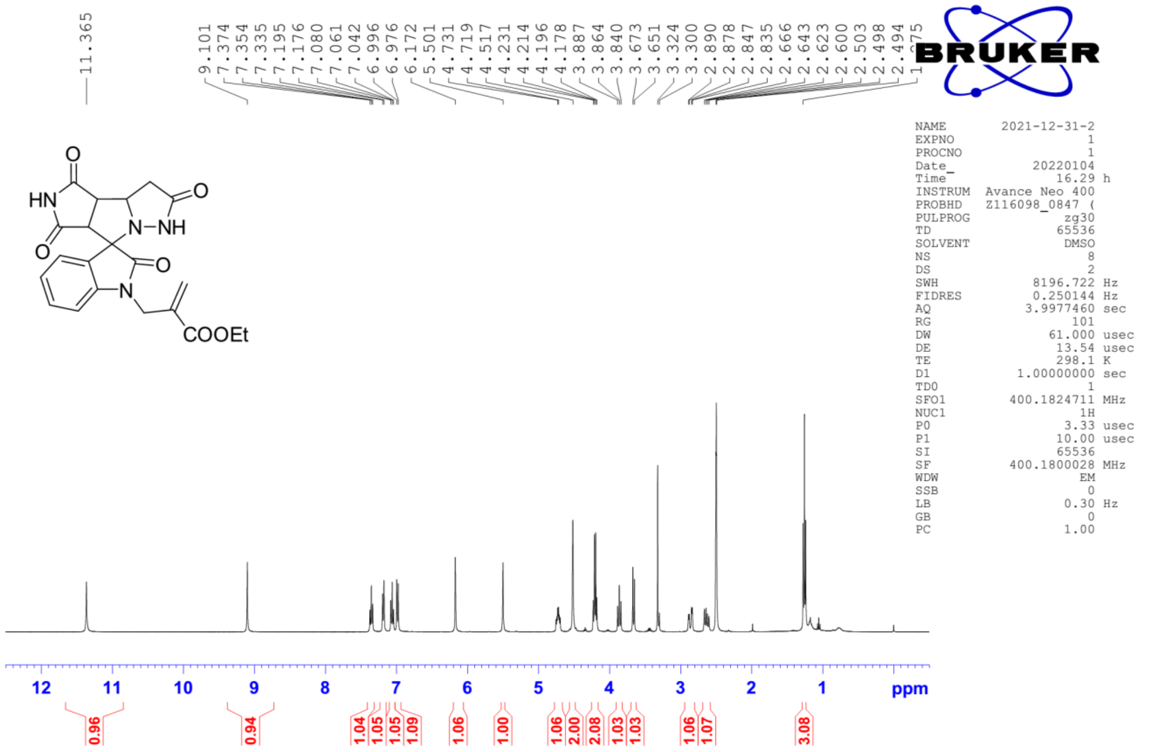

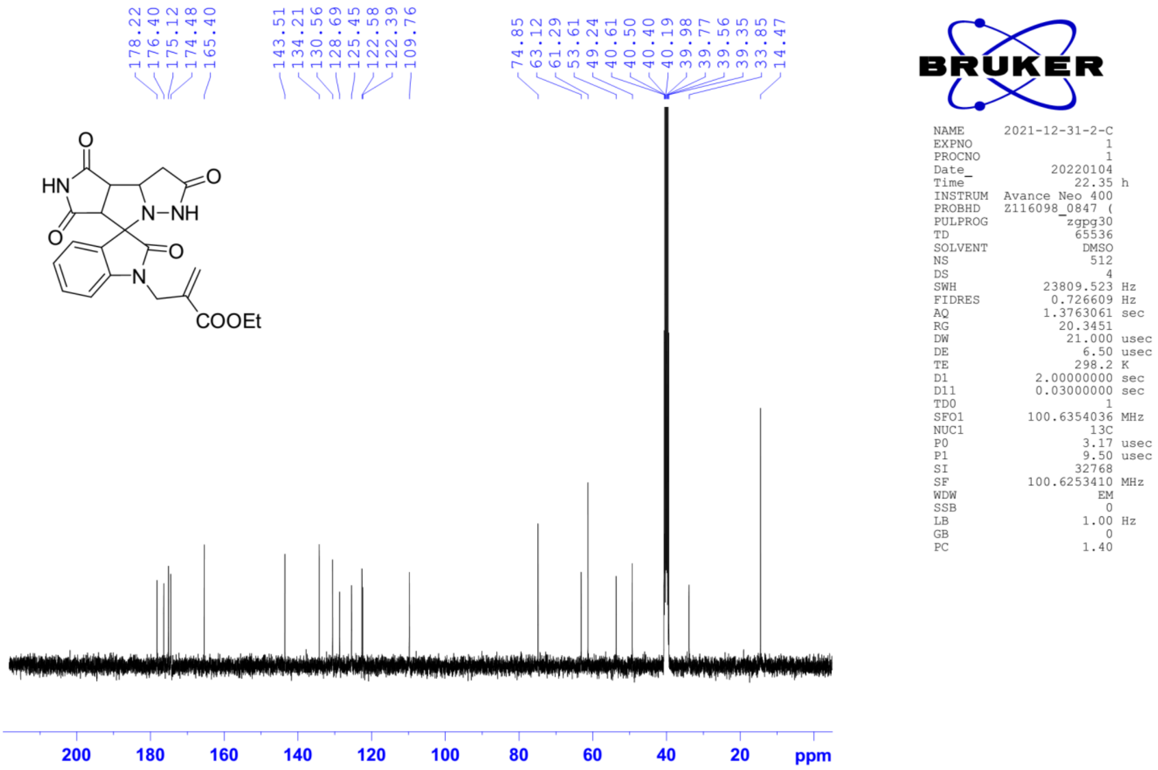

4. Copes of HRMS for all new compounds

HRMS for Compound **3a**

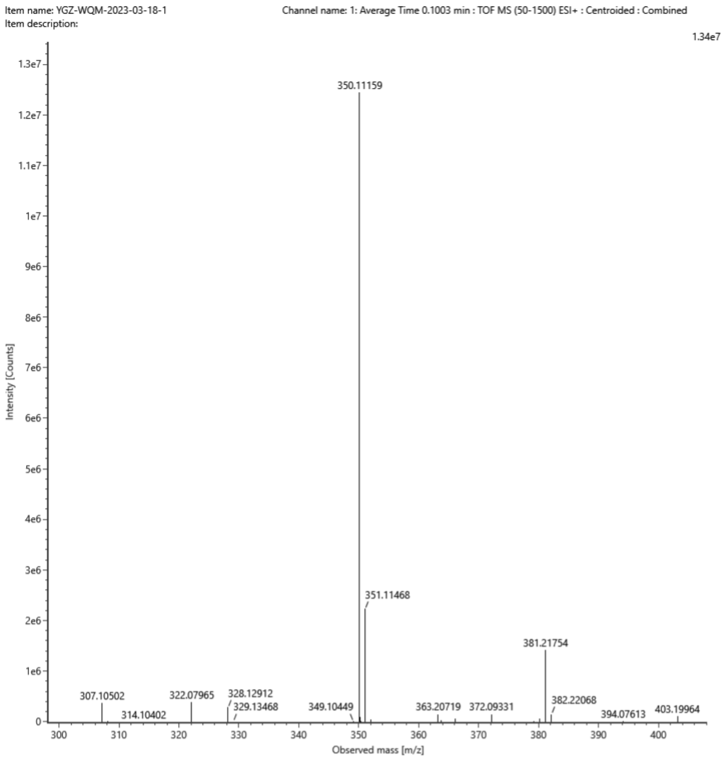

HRMS for Compound **3b**

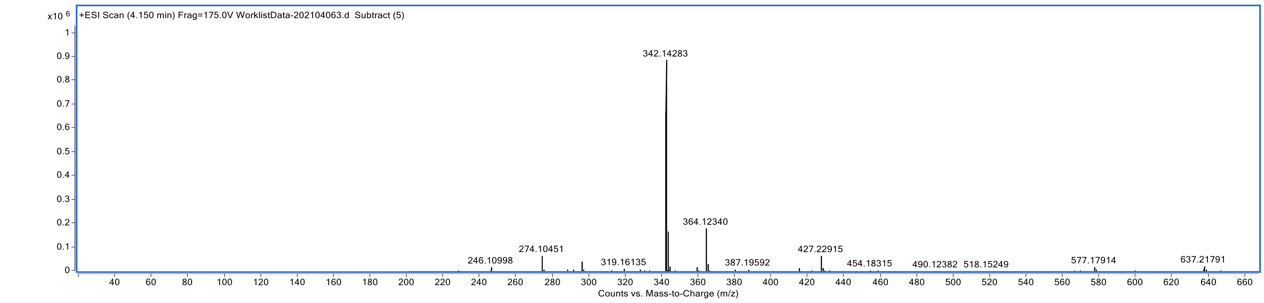

HRMS for Compound **3c**

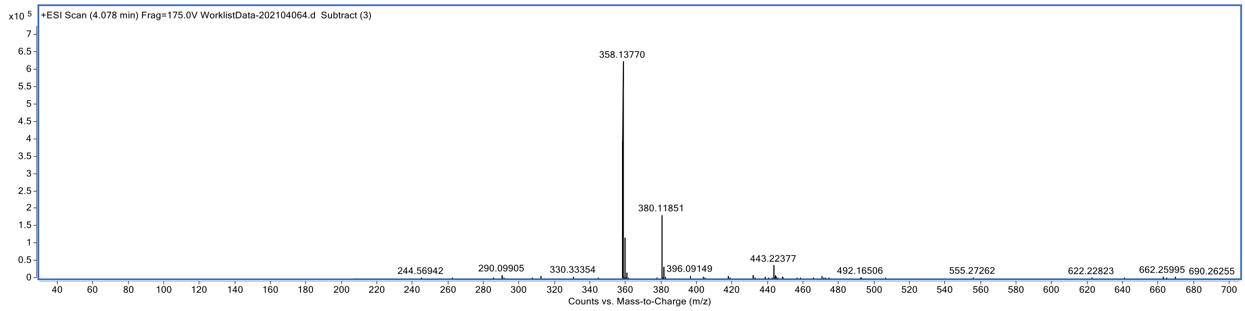

HRMS for Compound **3d**

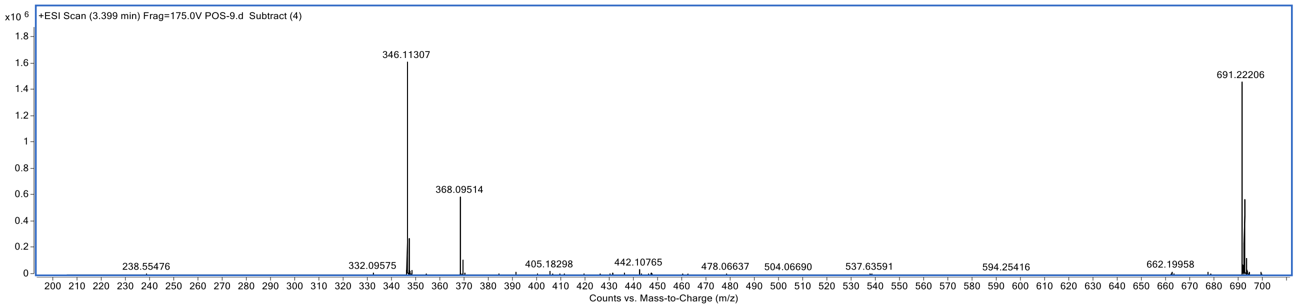

HRMS for Compound **3e**

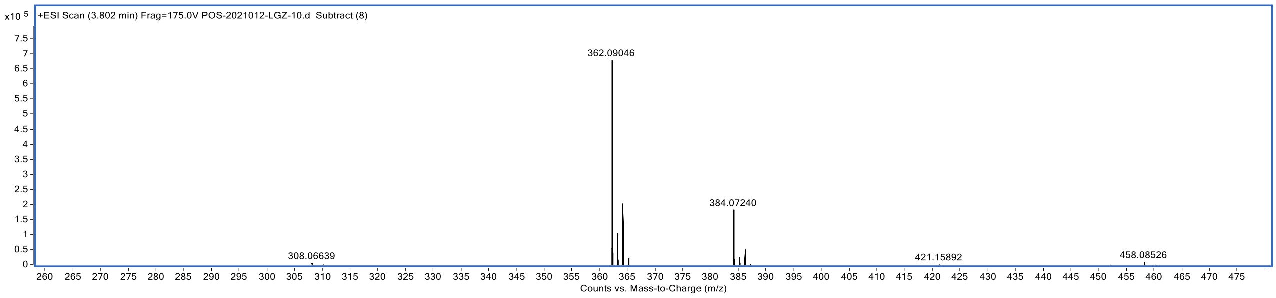

HRMS for Compound **3f**

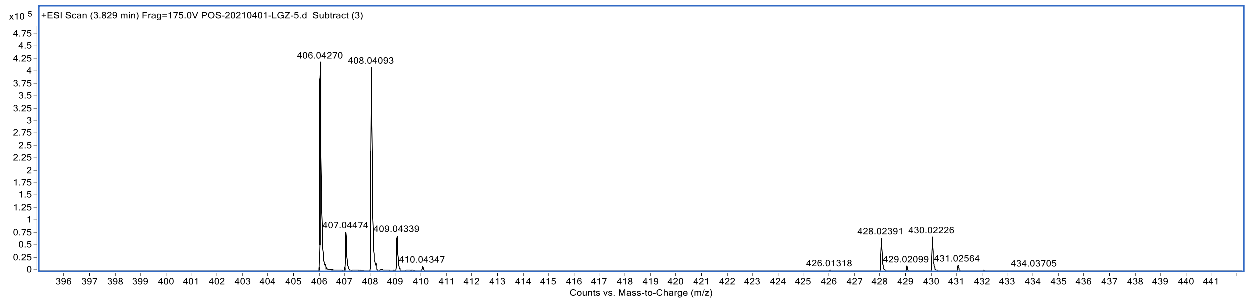

HRMS for Compound **3g**

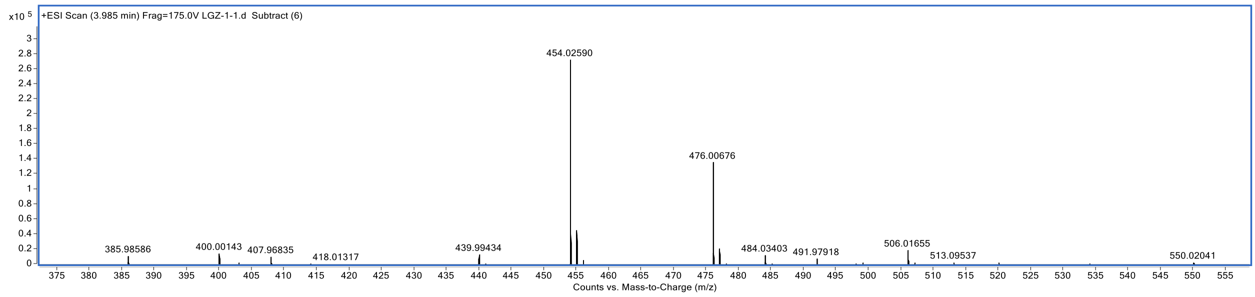

HRMS for Compound **3h**

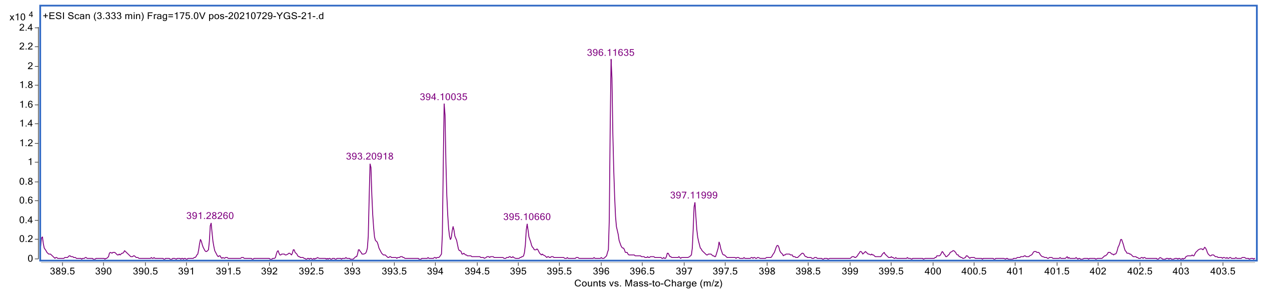

HRMS for Compound **3′i**

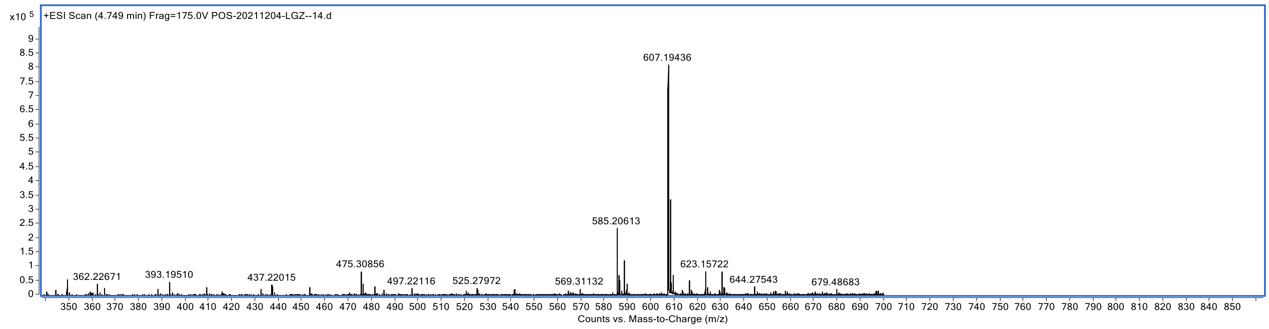

HRMS for Compound **3j**

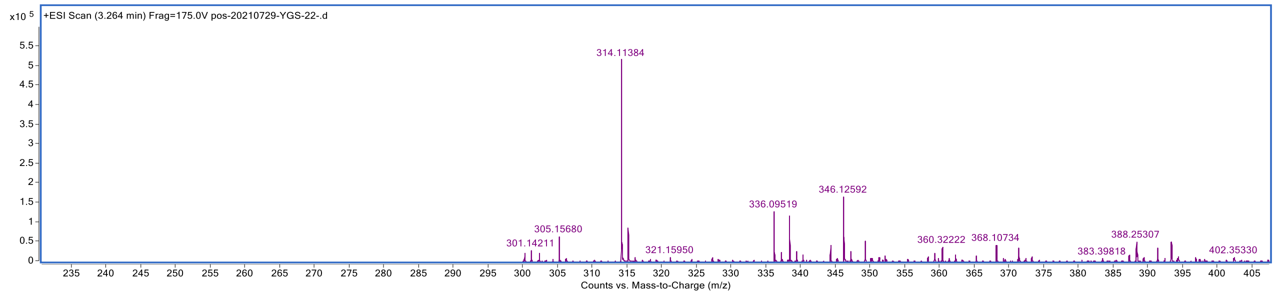

HRMS for Compound **3k**

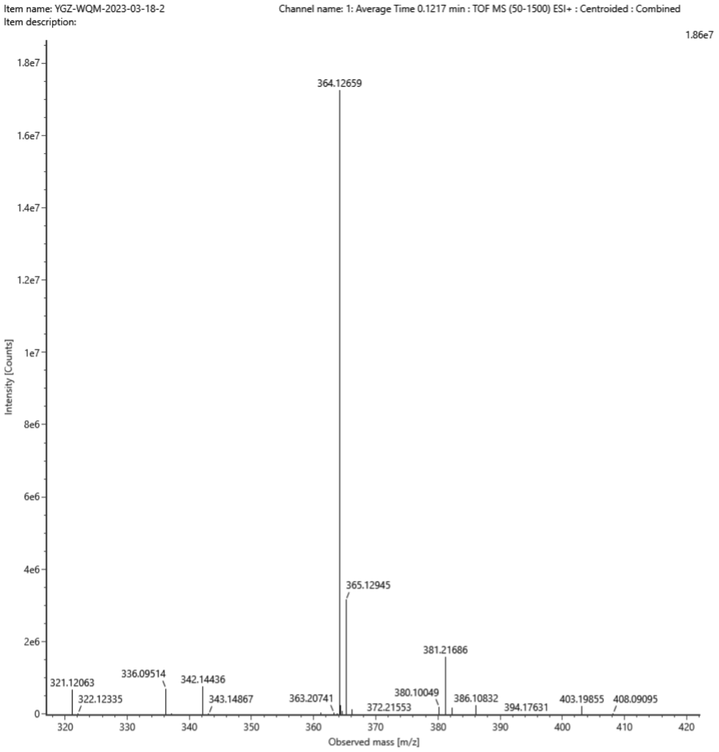

HRMS for Compound **3l**

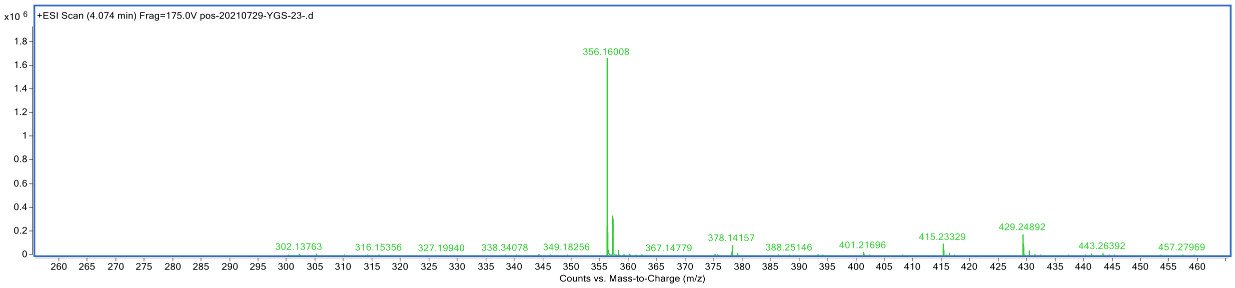

HRMS for Compound **3m**

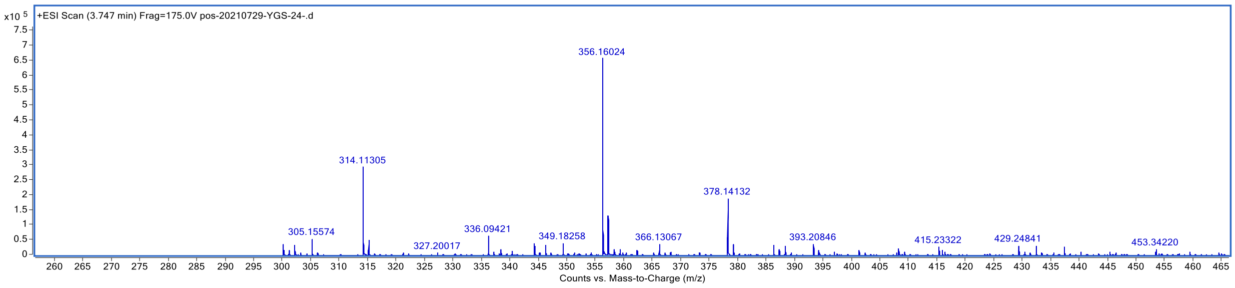

HRMS for Compound **6a**

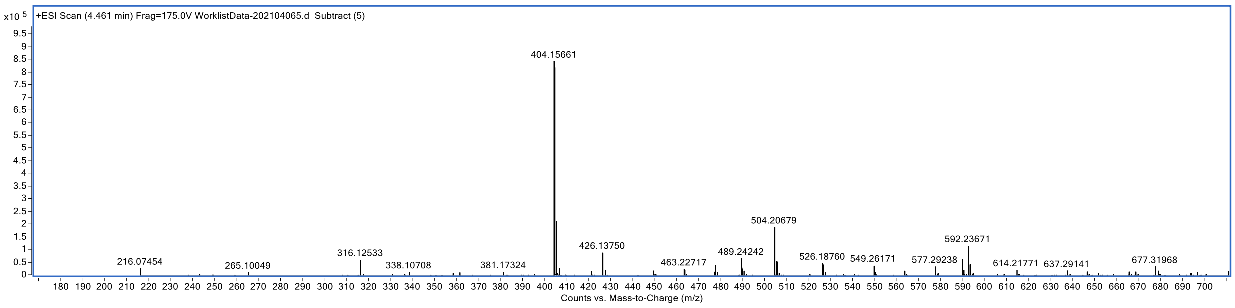

HRMS for Compound **6c**

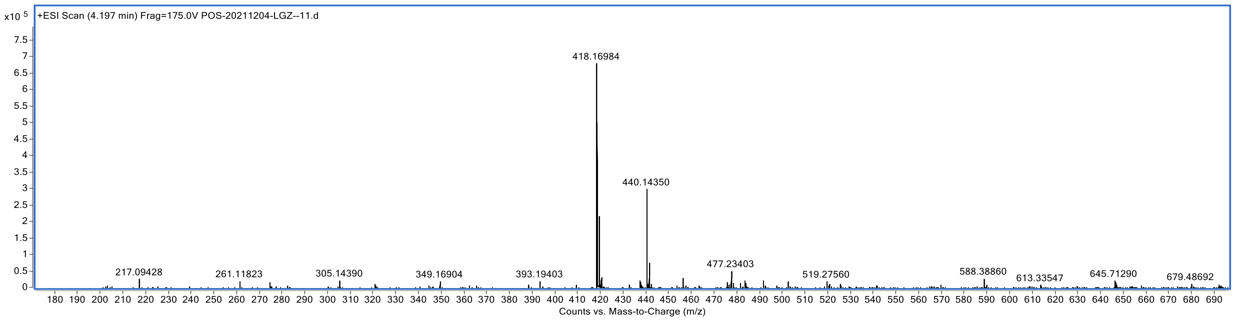

HRMS for Compound **6d**

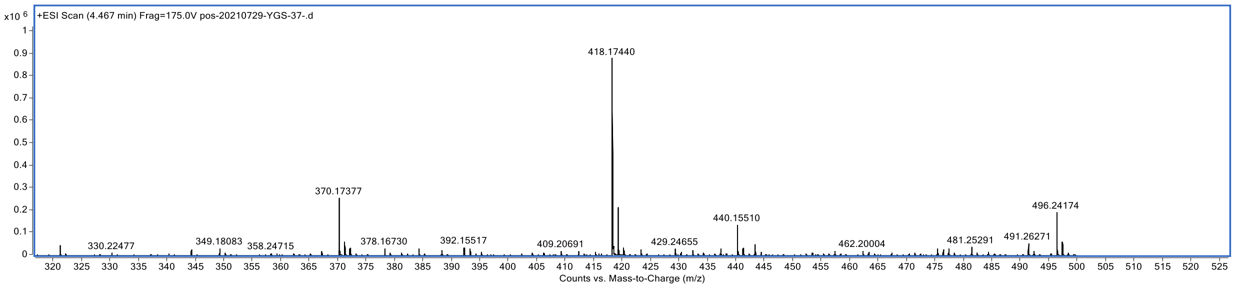

HRMS for Compound **6e**

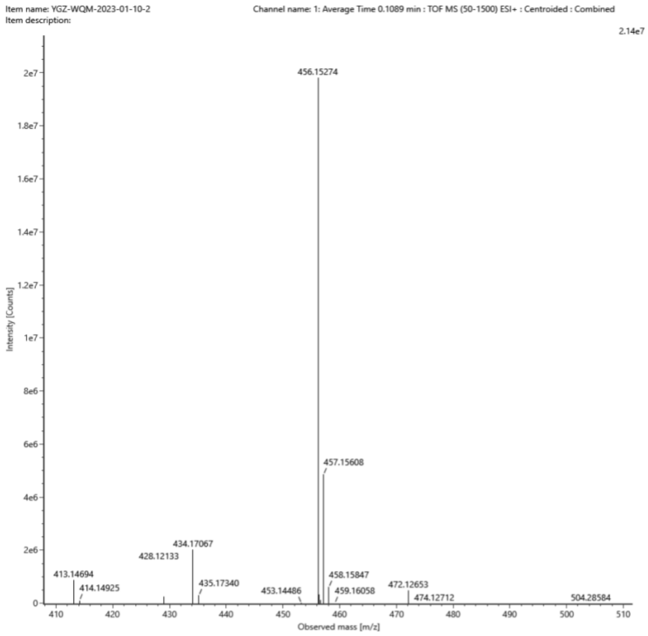

HRMS for Compound **6f**

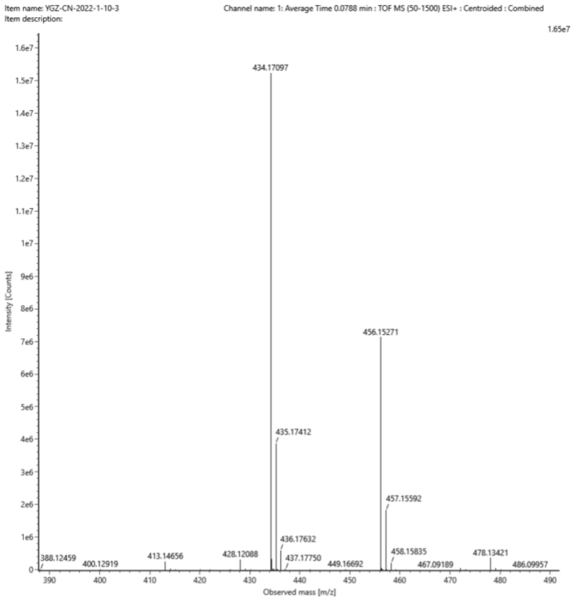

HRMS for Compound **6i**

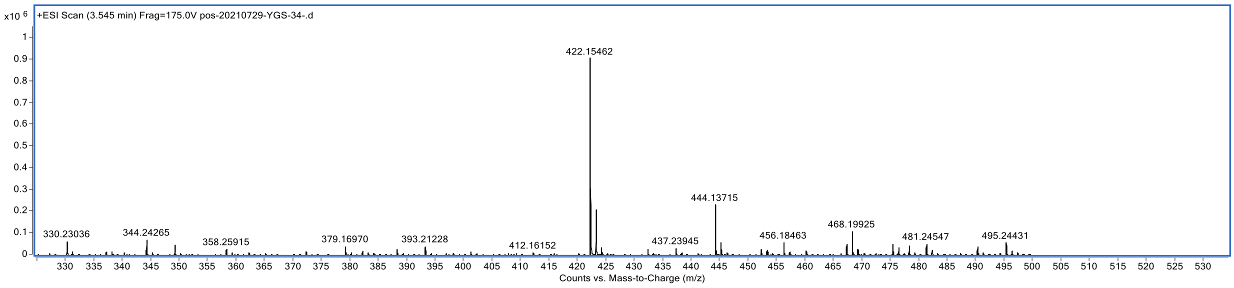

HRMS for Compound **6l**

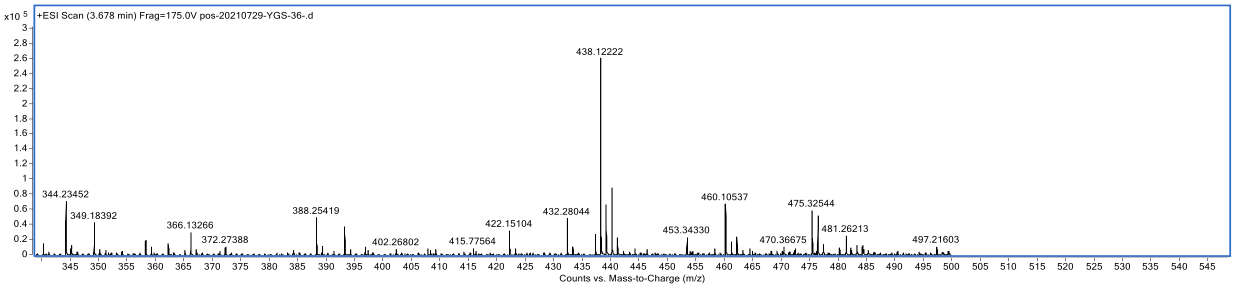

HRMS for Compound **6m**

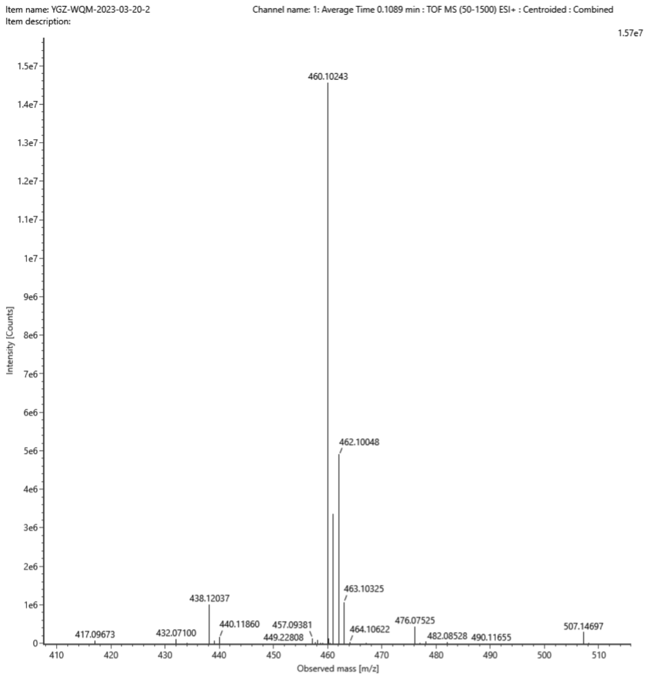

HRMS for Compound **6o**

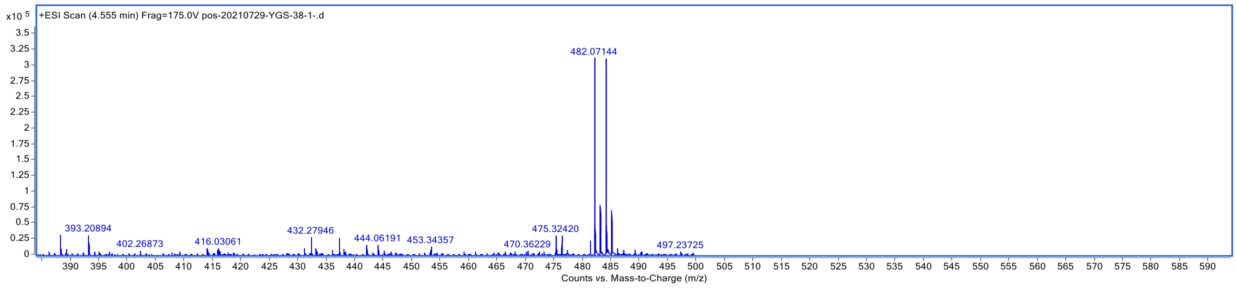

HRMS for Compound **6p**

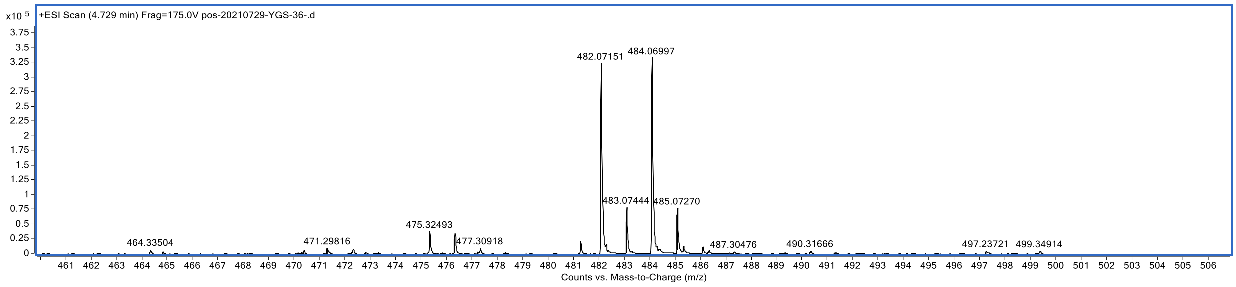

HRMS for Compound **6r**

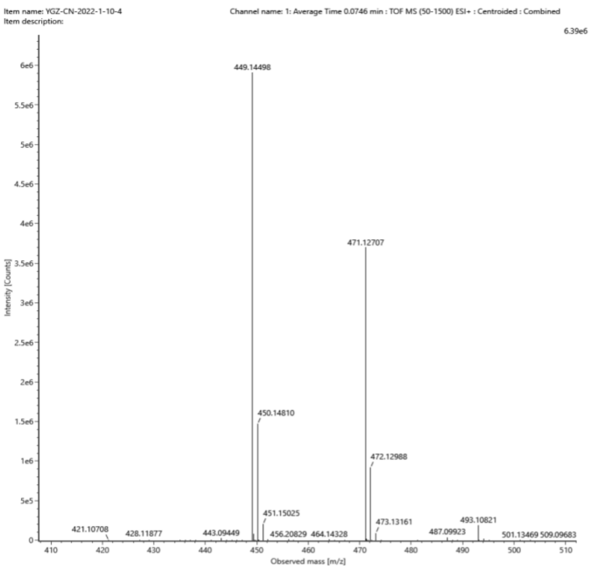

HRMS for Compound **6s**

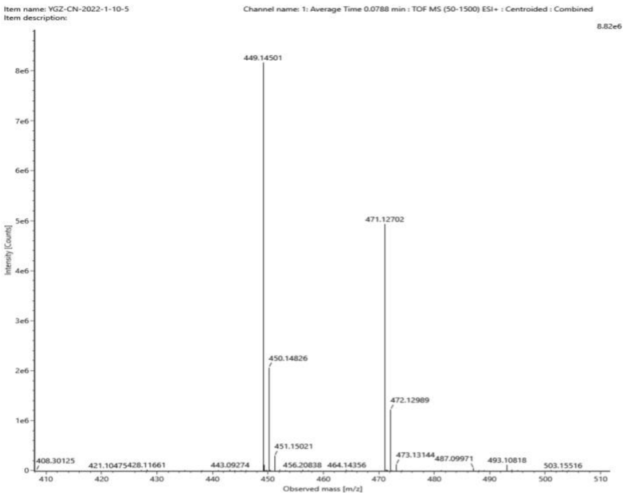

HRMS for Compound **6’t**

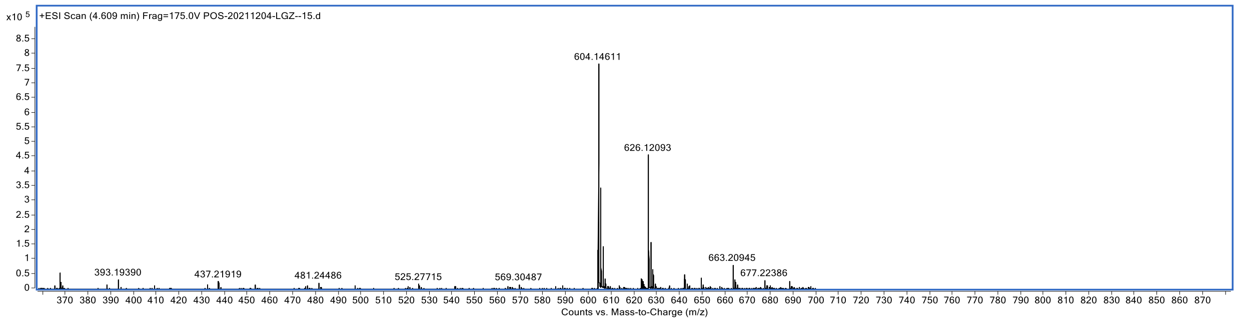

HRMS for Compound **8b**

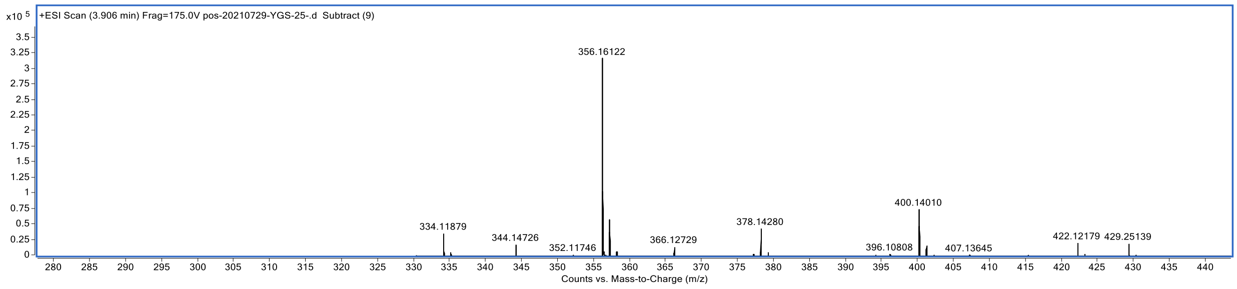

HRMS for Compound **8c**

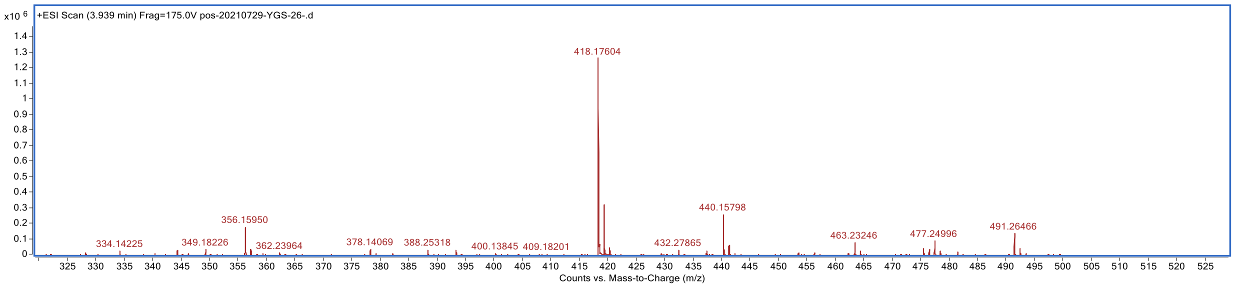

HRMS for Compound **8d**

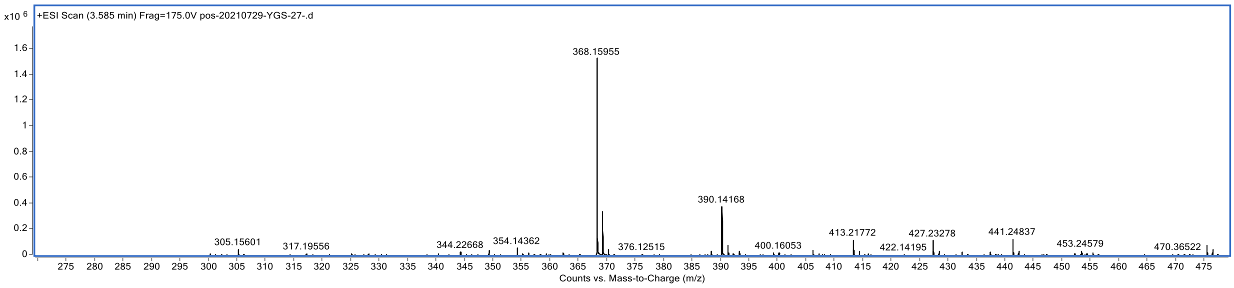

HRMS for Compound **8e**

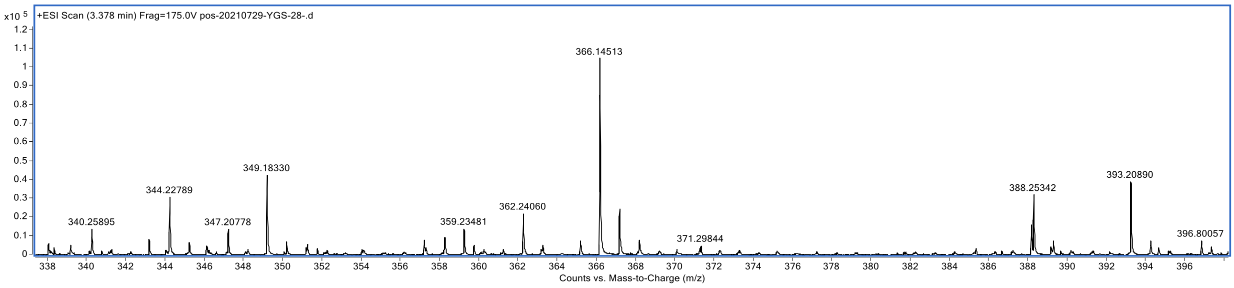

HRMS for Compound **8f**

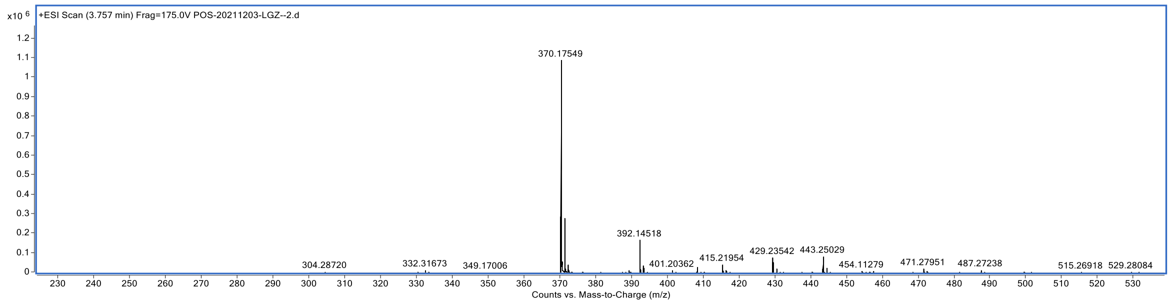

HRMS for Compound **8g**

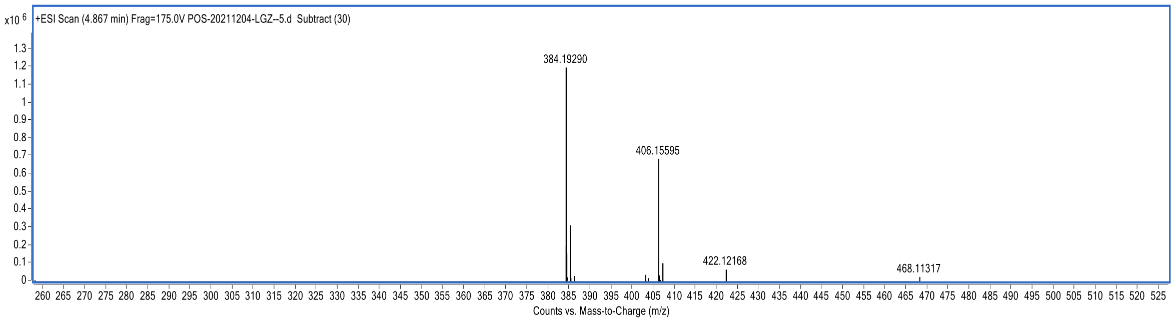

HRMS for Compound **8h**

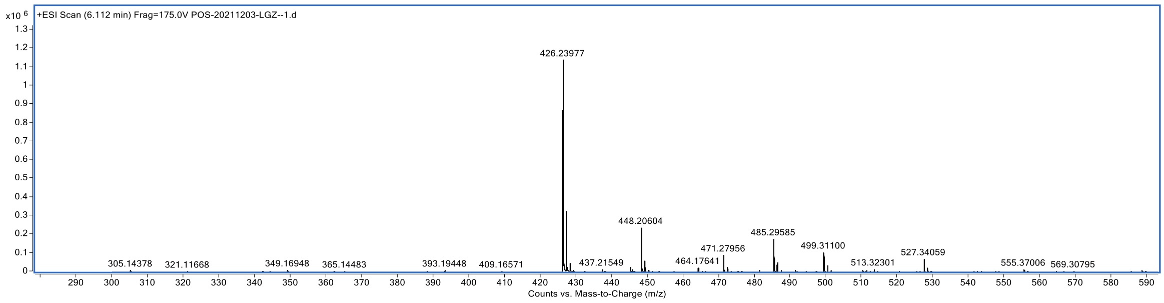

HRMS for Compound **8i**

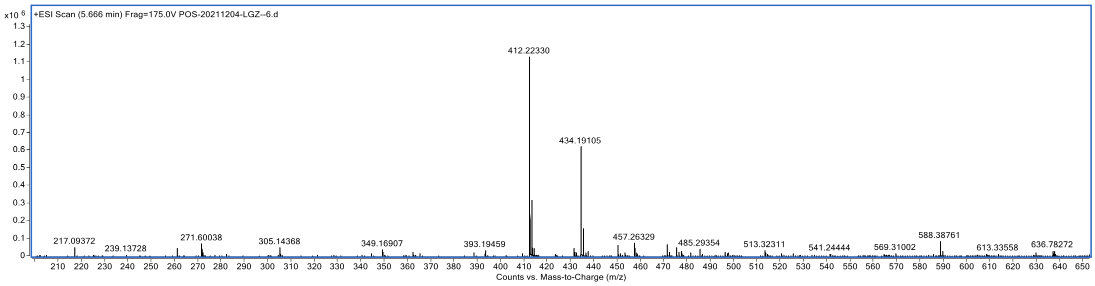

HRMS for Compound **8j**

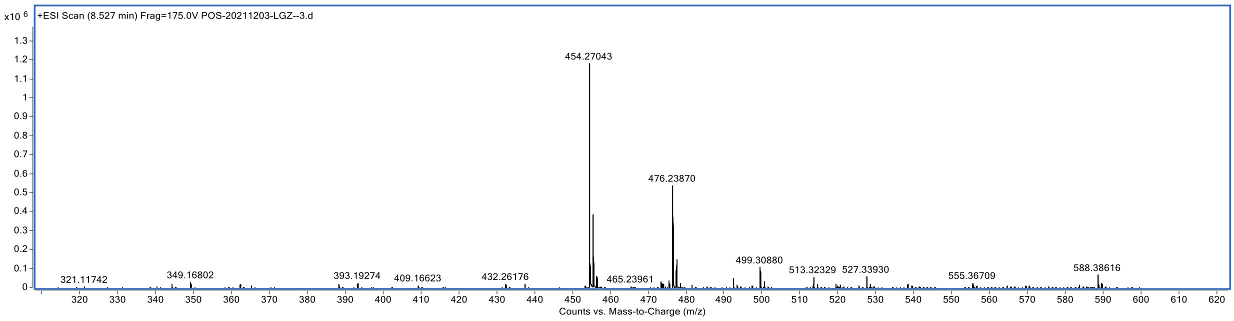

HRMS for Compound **8k**

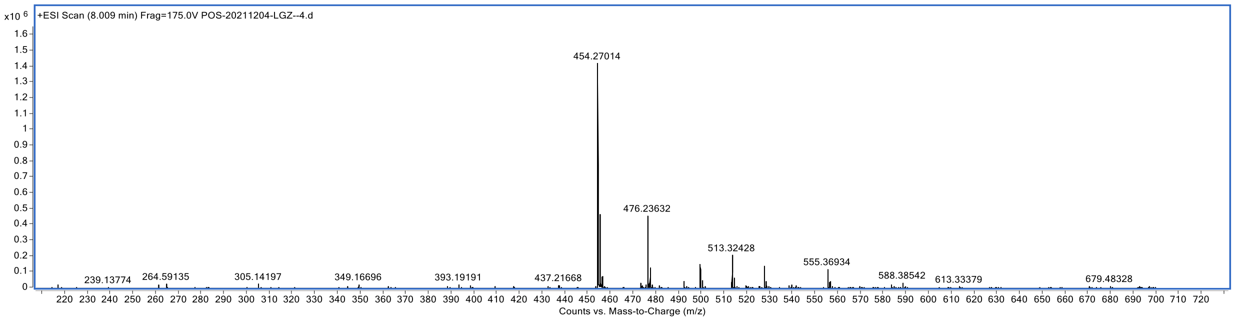

HRMS for Compound **4a**

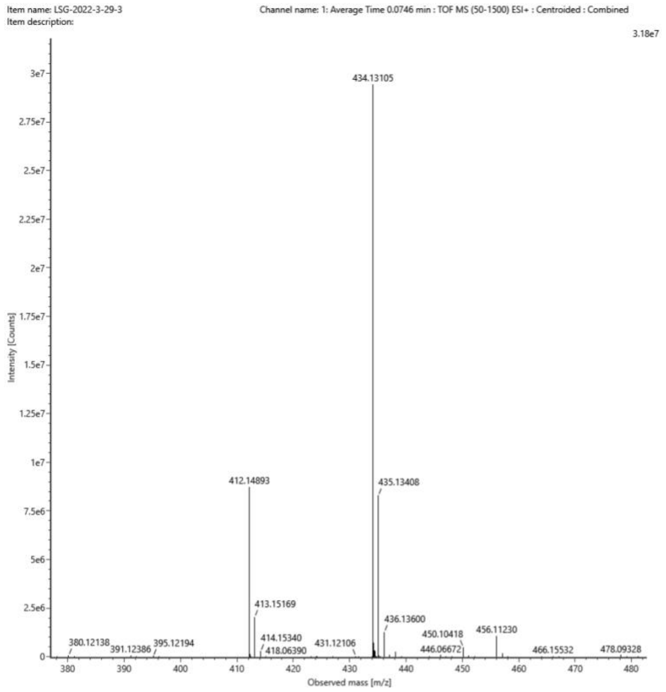

HRMS for Compound **4b**

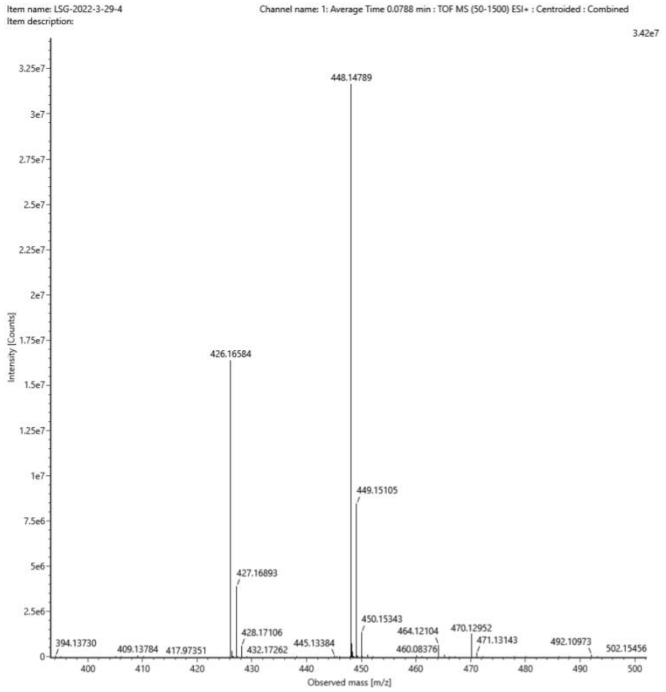

HRMS for Compound **4c**

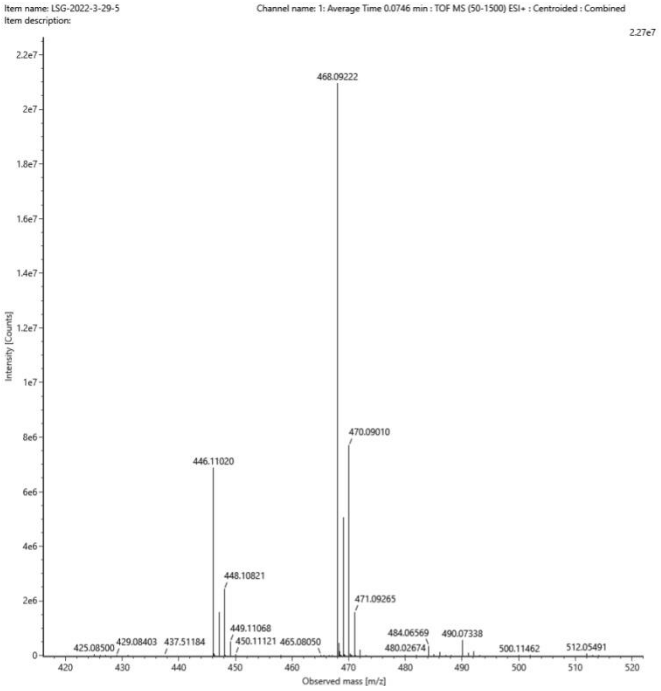

HRMS for Compound **4d**

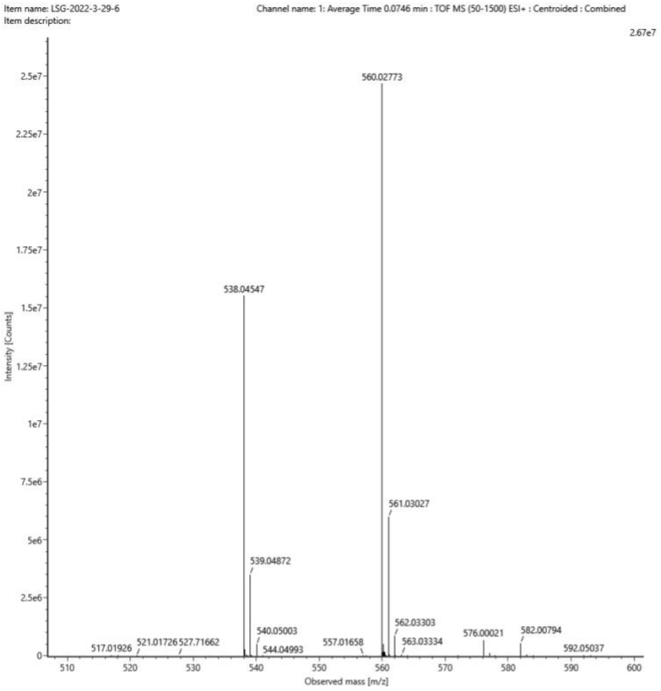

HRMS for Compound **4e**

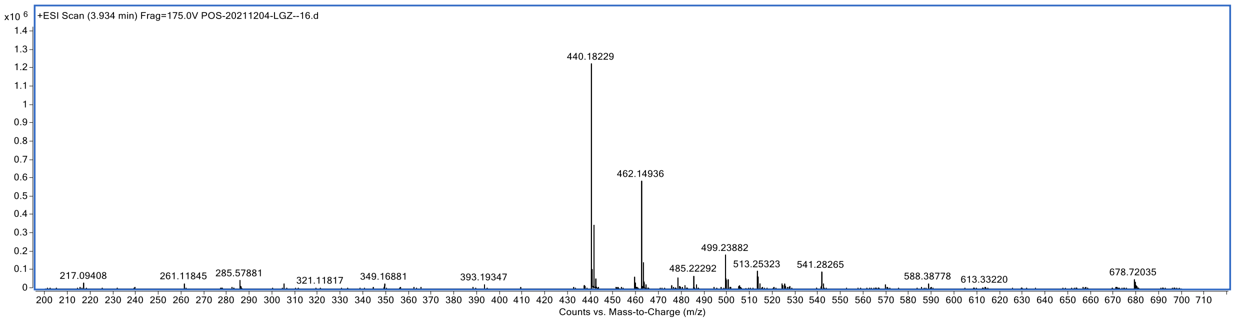

HRMS for Compound **4f**

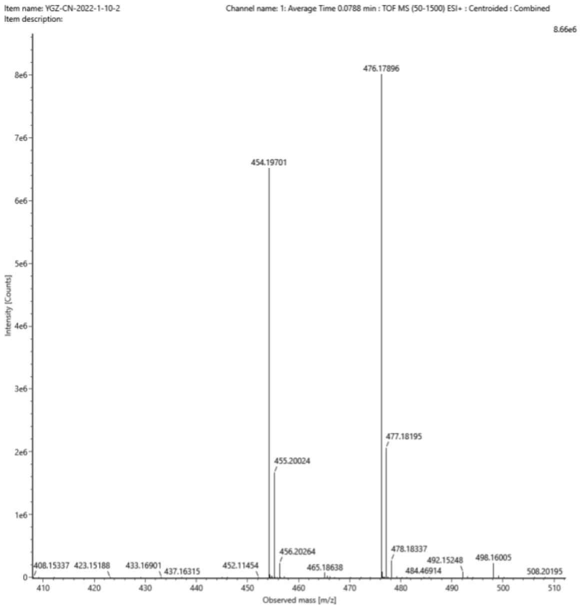

HRMS for Compound **4g**

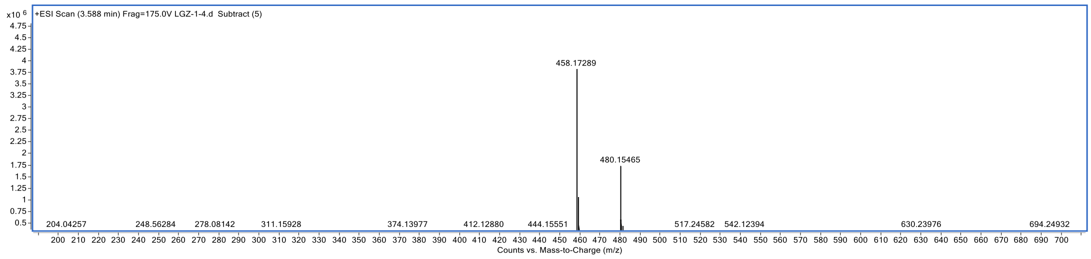

HRMS for Compound **4h**

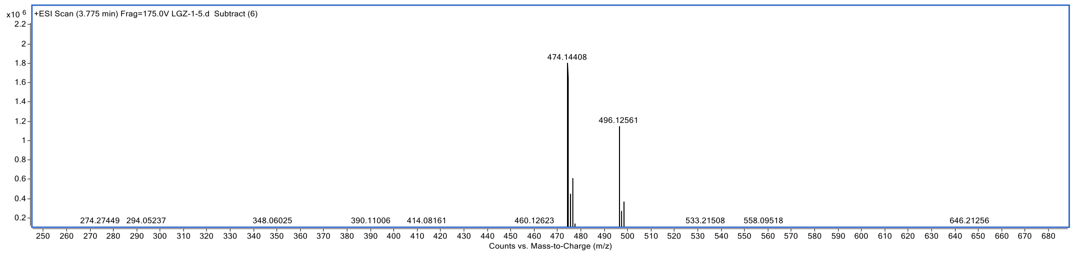

HRMS for Compound **4i**

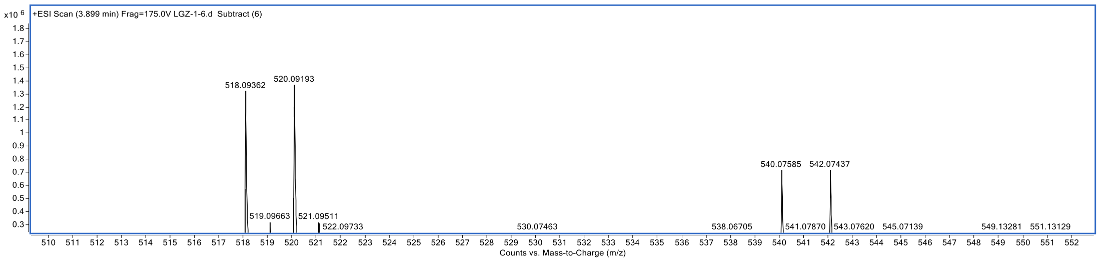

HRMS for Compound **4j**

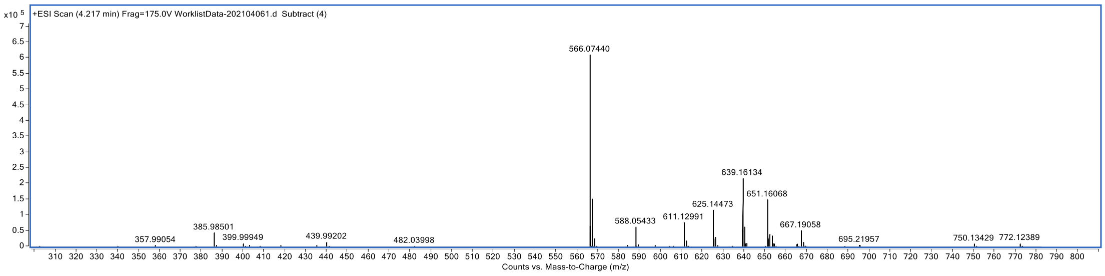

HRMS for Compound **10**

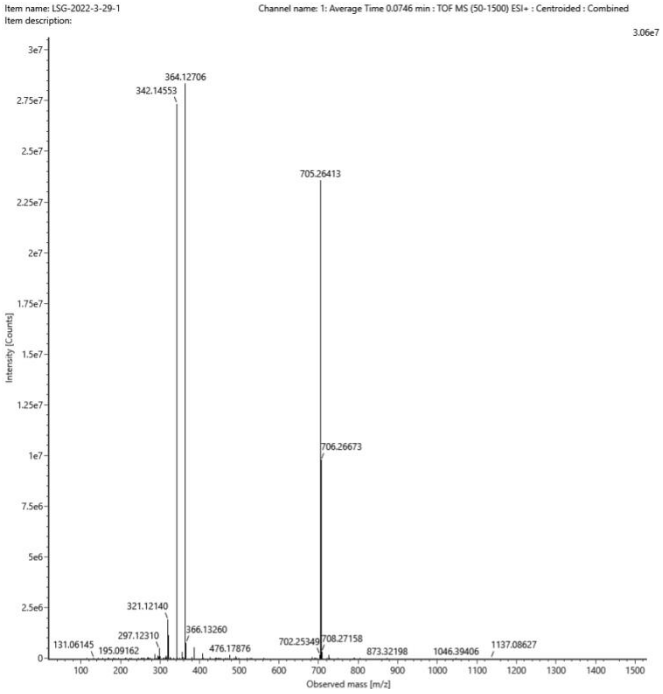

HRMS for Compound **12**

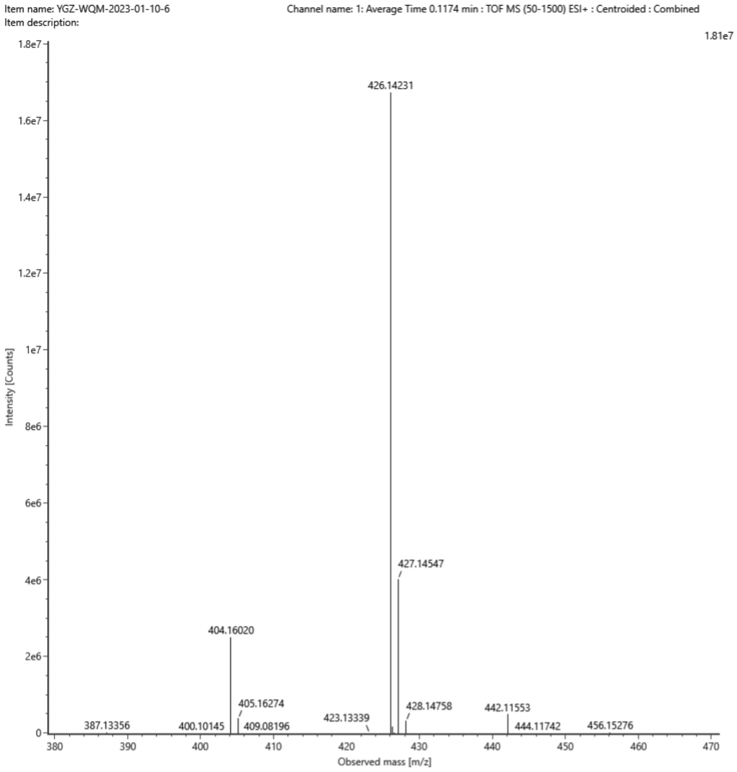

HRMS for Compound **13**

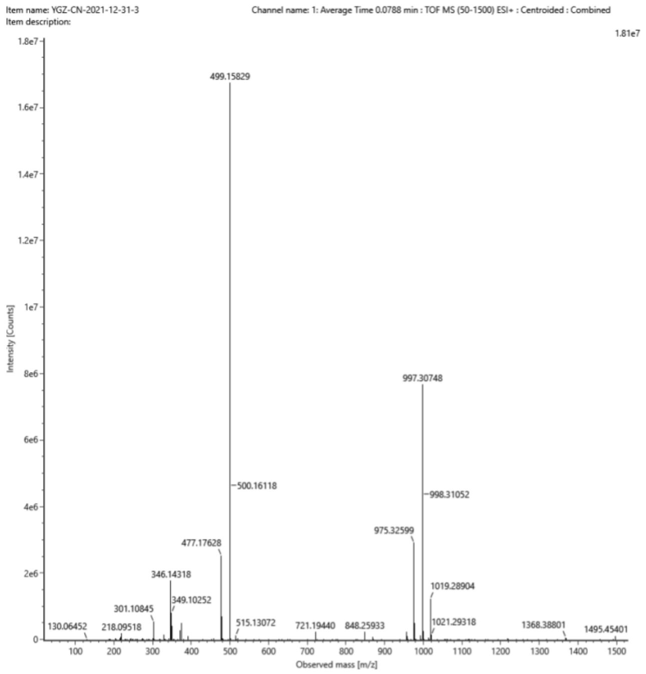

HRMS for Compound **14**

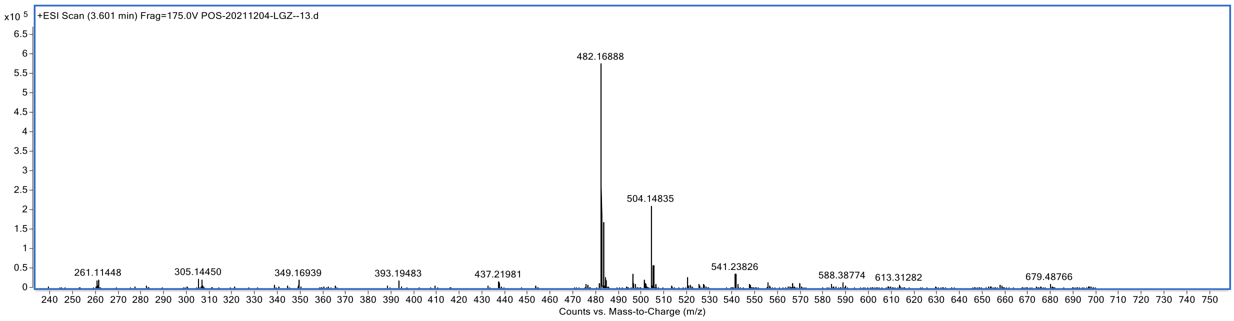

HRMS for Compound **15**

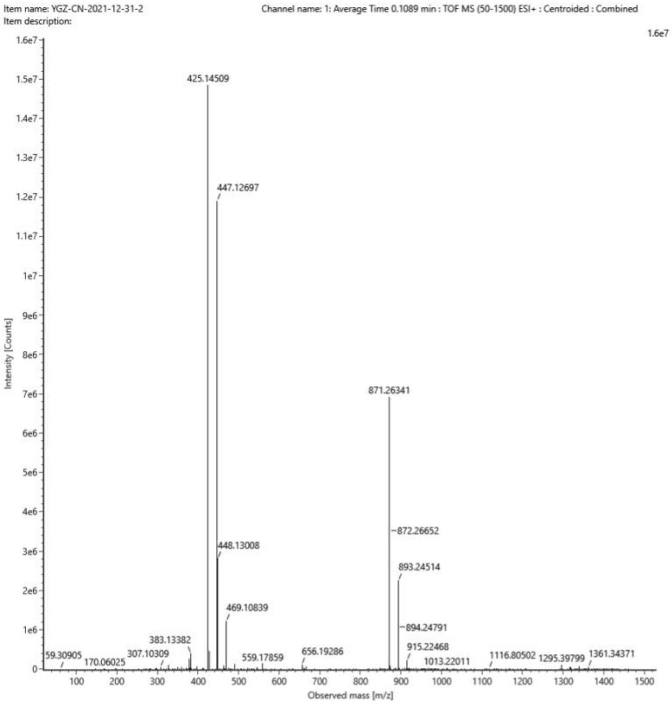

5. Copes of Data of X-ray crystal structure for 3′i (CCDC 2108657).

Datablock 111 - ellipsoid plot

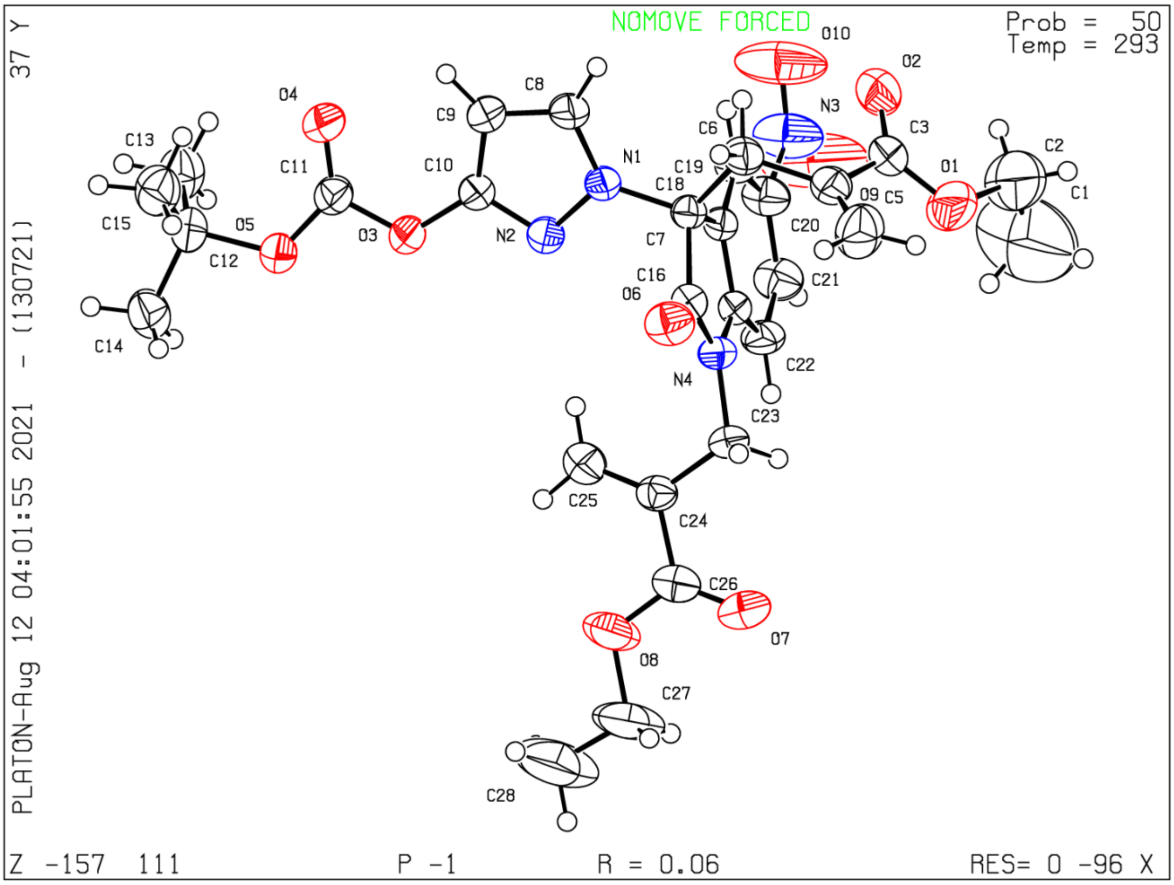

checkCIF/PLATON report

You have not supplied any structure factors. As a result the full set of tests cannot be run.

THIS REPORT IS FOR GUIDANCE ONLY. IF USED AS PART OF A REVIEW PROCEDURE FOR PUBLICATION, IT SHOULD NOT REPLACE THE EXPERTISE OF AN EXPERIENCED CRYSTALLOGRAPHIC REFEREE.

No syntax errors found. CIF dictionary Interpreting this report

Datablock: 111

```
Bond precision:   C-C = 0.0034 A          Wavelength=0.71073

Cell:            a=10.738(2)        b=10.755(2)       c=14.284(3)
                 alpha=67.993(6)    beta=88.754(8)    gamma=79.339(6)
Temperature:     293 K

                  Calculated                  Reported
Volume            1501.0(5)                   1501.0(5)
Space group       P -1                        P -1
Hall group        -P 1                        -P 1
Moiety formula    C28 H32 N4 O10              C28 H32 N4 O10
Sum formula       C28 H32 N4 O10              C28 H32 N4 O10
Mr                584.58                      584.57
Dx,g cm-3         1.293                       1.293
Z                 2                           2
Mu (mm-1)         0.099                       0.099
F000              616.0                       616.0
F000'             616.34
h,k,lmax          14,14,18                    14,14,18
Nref              7035                        6994
Tmin,Tmax         0.965,0.981                 0.708,0.746
Tmin'             0.965

Correction method= # Reported T Limits: Tmin=0.708 Tmax=0.746
AbsCorr = MULTI-SCAN

Data completeness= 0.994           Theta(max)= 27.728

R(reflections)= 0.0601( 4671)      wR2(reflections)= 0.1755( 6994)

S = 1.027                  Npar= 384
```

The following ALERTS were generated. Each ALERT has the format
test-name_ALERT_alert-type_alert-level.
Click on the hyperlinks for more details of the test.

checkCIF/PLATON report

You have not supplied any structure factors. As a result the full set of tests cannot be run.

THIS REPORT IS FOR GUIDANCE ONLY. IF USED AS PART OF A REVIEW PROCEDURE FOR PUBLICATION, IT SHOULD NOT REPLACE THE EXPERTISE OF AN EXPERIENCED CRYSTALLOGRAPHIC REFEREE.

No syntax errors found. CIF dictionary Interpreting this report

Datablock: 2_sq

Bond precision:	C-C = 0.0094 A		Wavelength=0.71073
Cell:	a=8.1785(14)	b=25.516(4)	c=12.111(2)
	alpha=90	beta=90	gamma=90
Temperature:	273 K		

	Calculated	Reported
Volume	2527.4(7)	2527.3(7)
Space group	P n a 21	P n a 21
Hall group	P 2c -2n	P 2c -2n
Moiety formula	C26 H20 Br N3 O3 [+ solvent]	C26 H20 Br N3 O3
Sum formula	C26 H20 Br N3 O3 [+ solvent]	C26 H20 Br N3 O3
Mr	502.35	502.36
Dx,g cm-3	1.320	1.320
Z	4	4
Mu (mm-1)	1.657	1.657
F000	1024.0	1024.0
F000'	1023.25	
h,k,lmax	10,33,15	10,33,15
Nref	5758[3016]	5674
Tmin,Tmax	0.667,0.806	0.410,0.746
Tmin'	0.654	

Correction method= # Reported T Limits: Tmin=0.410 Tmax=0.746
AbsCorr = MULTI-SCAN

Data completeness= 1.88/0.99 Theta(max)= 27.413

R(reflections)= 0.0573(3876) wR2(reflections)= 0.1471(5674)

S = 1.017 Npar= 298

Alert level B

```
PLAT220_ALERT_2_B NonSolvent   Resd 1   C   Ueq(max)/Ueq(min) Range        7.1 Ratio
```

Alert level C

```
PLAT220_ALERT_2_C NonSolvent   Resd 1   O   Ueq(max)/Ueq(min) Range        3.4 Ratio
PLAT222_ALERT_3_C NonSolvent Resd 1  H   Uiso(max)/Uiso(min) Range        7.4 Ratio
PLAT242_ALERT_2_C Low    'MainMol' Ueq as Compared to Neighbors of         N3 Check
PLAT242_ALERT_2_C Low    'MainMol' Ueq as Compared to Neighbors of         C2 Check
PLAT242_ALERT_2_C Low    'MainMol' Ueq as Compared to Neighbors of         C3 Check
PLAT242_ALERT_2_C Low    'MainMol' Ueq as Compared to Neighbors of        C26 Check
PLAT360_ALERT_2_C Short  C(sp3)-C(sp3) Bond  C1       - C2       .      1.34 Ang.
PLAT360_ALERT_2_C Short  C(sp3)-C(sp3) Bond  C27      - C28      .      1.38 Ang.
```

Alert level G

```
PLAT003_ALERT_2_G Number of Uiso or Uij Restrained non-H Atoms ...          3 Report
PLAT177_ALERT_4_G The CIF-Embedded .res File Contains DELU Records          1 Report
PLAT178_ALERT_4_G The CIF-Embedded .res File Contains SIMU Records          1 Report
PLAT199_ALERT_1_G Reported _cell_measurement_temperature ..... (K)        293 Check
PLAT200_ALERT_1_G Reported   _diffrn_ambient_temperature ..... (K)        293 Check
PLAT793_ALERT_4_G Model has Chirality at C7          (Centro SPGR)          R Verify
PLAT860_ALERT_3_G Number of Least-Squares Restraints ............          15 Note
PLAT941_ALERT_3_G Average HKL Measurement Multiplicity ..........         4.6 Low
```

```
  0 ALERT level A = Most likely a serious problem - resolve or explain
  1 ALERT level B = A potentially serious problem, consider carefully
  8 ALERT level C = Check. Ensure it is not caused by an omission or oversight
  8 ALERT level G = General information/check it is not something unexpected

  2 ALERT type 1 CIF construction/syntax error, inconsistent or missing data
  9 ALERT type 2 Indicator that the structure model may be wrong or deficient
  3 ALERT type 3 Indicator that the structure quality may be low
  3 ALERT type 4 Improvement, methodology, query or suggestion
  0 ALERT type 5 Informative message, check
```

It is advisable to attempt to resolve as many as possible of the alerts in all categories. Often the minor alerts point to easily fixed oversights, errors and omissions in your CIF or refinement strategy, so attention to these fine details can be worthwhile. In order to resolve some of the more serious problems it may be necessary to carry out additional measurements or structure refinements. However, the purpose of your study may justify the reported deviations and the more serious of these should normally be commented upon in the discussion or experimental section of a paper or in the "special_details" fields of the CIF. checkCIF was carefully designed to identify outliers and unusual parameters, but every test has its limitations and alerts that are not important in a particular case may appear. Conversely, the absence of alerts does not guarantee there are no aspects of the results needing attention. It is up to the individual to critically assess their own results and, if necessary, seek expert advice.

Publication of your CIF in IUCr journals

A basic structural check has been run on your CIF. These basic checks will be run on all CIFs submitted for publication in IUCr journals (*Acta Crystallographica*, *Journal of Applied Crystallography*, *Journal of Synchrotron Radiation*); however, if you intend to submit to *Acta Crystallographica Section C* or *E* or *IUCrData*, you should make sure that full publication checks are run on the final version of your CIF prior to submission.

Publication of your CIF in other journals

Please refer to the *Notes for Authors* of the relevant journal for any special instructions relating to CIF submission.

PLATON version of 13/07/2021; check.def file version of 13/07/2021
